# XIAP-mediated degradation of IFT88 disrupts HSC cilia to stimulate HSC activation and liver fibrosis

**DOI:** 10.1038/s44319-024-00092-y

**Published:** 2024-02-13

**Authors:** Renjie Hong, Yanjie Tan, Xiaoyu Tian, Zhenzhou Huang, Jiaying Wang, Hua Ni, Jia Yang, Weiwen Bu, Song Yang, Te Li, Fan Yu, Weilong Zhong, Tao Sun, Xiaohong Wang, Dengwen Li, Min Liu, Yunfan Yang, Jun Zhou

**Affiliations:** 1 Department of Genetics and Cell Biology, State Key Laboratory of Medicinal Chemical Biology, Haihe Laboratory of Cell Ecosystem, Tianjin Key Laboratory of Protein Science, College of Life Sciences, Nankai University, 300071 Tianjin, China; 2 Center for Cell Structure and Function, Collaborative Innovation Center of Cell Biology in Universities of Shandong, Shandong Provincial Key Laboratory of Animal Resistance Biology, College of Life Sciences, Shandong Normal University, 250014 Jinan, China; 3 Department of Gastroenterology and Hepatology, General Hospital, Tianjin Medical University, 300052 Tianjin, China; 4 State Key Laboratory of Medicinal Chemical Biology, College of Pharmacy, Nankai University, 300071 Tianjin, China; 5 Department of Pharmacology and Tianjin Key Laboratory of Inflammation Biology, School of Basic Medical Sciences, Tianjin Medical University, 300070 Tianjin, China; 6 Department of Cell Biology, School of Basic Medical Sciences, Cheeloo College of Medicine, Shandong University, 250012 Jinan, China

**Keywords:** Liver Fibrosis, Hepatic Stellate Cell, Cilium, Ubiquitination, Proteasomal Degradation, Cell Adhesion, Polarity & Cytoskeleton, Molecular Biology of Disease, Post-translational Modifications & Proteolysis

## Abstract

Activation of hepatic stellate cells (HSCs) plays a critical role in liver fibrosis. However, the molecular basis for HSC activation remains poorly understood. Herein, we demonstrate that primary cilia are present on quiescent HSCs but exhibit a significant loss upon HSC activation which correlates with decreased levels of the ciliary protein intraflagellar transport 88 (IFT88). *Ift88*-knockout mice are more susceptible to chronic carbon tetrachloride-induced liver fibrosis. Mechanistic studies show that the X-linked inhibitor of apoptosis (XIAP) functions as an E3 ubiquitin ligase for IFT88. Transforming growth factor-β (TGF-β), a profibrotic factor, enhances XIAP-mediated ubiquitination of IFT88, promoting its proteasomal degradation. Blocking XIAP-mediated IFT88 degradation ablates TGF-β-induced HSC activation and liver fibrosis. These findings reveal a previously unrecognized role for ciliary homeostasis in regulating HSC activation and identify the XIAP–IFT88 axis as a potential therapeutic target for liver fibrosis.

## Synopsis

Profibrotic stimuli disrupt primary cilia on quiescent hepatic stellate cells (HSCs) by inducing XIAP-mediated ubiquitination and proteasomal degradation of IFT88. This contributes to HSC activation and liver fibrosis.

- Primary cilia exist on multiple cell types in the liver, but only cilia present on HSCs disassemble during liver fibrosis.
- TGF-β enhances XIAP-mediated ubiquitination of IFT88, promoting its proteasomal degradation.
- Blocking XIAP-mediated IFT88 degradation ablates TGF-β-induced HSC activation.

## Abstract (web summary)

Profibrotic stimuli disrupt primary cilia on quiescent hepatic stellate cells (HSCs) by inducing XIAP-mediated ubiquitination and proteasomal degradation of IFT88. This contributes to HSC activation and liver fibrosis.

## Graphical abstract

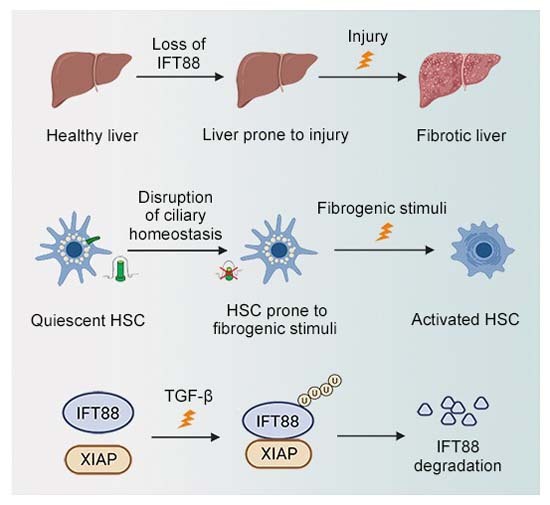

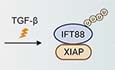

## Introduction

Liver fibrosis is characterized as excessive deposition of the extracellular matrix (ECM) in the liver, in response to chronic liver injury caused by various factors, such as alcohol, nonalcoholic steatohepatitis, drugs, nonalcoholic fatty liver disease, and cholestatic liver disease (Devaraj et al, 2022; Kisseleva and Brenner, 2021). The prolonged presence of liver fibrosis usually leads to cirrhosis and hepatocellular carcinoma, which are major causes of morbidity and mortality worldwide (Li et al, 2022a). Therefore, a deeper understanding of the molecular mechanisms of the liver fibrosis process may help develop better prevention and treatment methods and provide novel strategies for the management of liver cancer.

Activation of hepatic stellate cells (HSCs) plays an important role in promoting ECM protein accumulation during liver fibrosis (Arab et al, 2020). Liver injury allows the expression and release of various cytokines and growth factors to activate HSCs (Baghaei et al, 2022; Tsuchida and Friedman, 2017). Activated HSCs proliferate and produce large amounts of α-smooth muscle actin (α-SMA) and ECM, which eventually lead to liver fibrosis (Baghaei et al, 2022; Tsuchida and Friedman, 2017). Transforming growth factor-β (TGF-β) plays a key role in the conversion of quiescent HSCs into fibrotic myofibroblasts (Dewidar et al, 2019; Guan et al, 2021). Strategies aimed at disrupting the synthesis of TGF-β have been shown to significantly ameliorate liver fibrosis in experimental models (Hsu et al, 2019; Hung et al, 2022; Lu et al, 2018; Xi et al, 2021). Alcohol, drugs and viral infections have been shown to induce liver fibrosis because these stimuli cause repeated damage to hepatocytes, which stimulates HSC activation (Tsuchida and Friedman, 2017). In addition, chronic carbon tetrachloride (CCl_4_) is commonly used to induce hepatocyte injury and thus mimics the process of liver fibrosis, and CCl_4_-treated mice exhibit dramatic upregulation of TGF-β and activation of HSCs (Biagioli et al, 2022).

Primary cilia are microtubule-based organelles that extend from the cell surface and act as cellular antenna for signal sensing and transduction (Breslow and Holland, 2019; Nachury and Mick, 2019). Defects in the structure and function of primary cilia cause a range of diseases, collectively referred to as ciliopathies (Nishimura et al, 2019; Yu et al, 2016). Liver fibrosis is one of the most common manifestations of ciliopathies (Dillard et al, 2018; Gunay-Aygun, 2009; Van De Weghe et al, 2017). However, no cilia were present on hepatocytes, which comprise about 80% of the cells in the liver (Mansini et al, 2018). Bile duct epithelial cells (BECs), also known as cholangiocytes, represent the only cell type in the liver that has been reported to contain cilia, but the absence of cilia on BECs only cause biliary fibrosis in aged mice and does not affect the development of advanced fibrosis or cirrhosis during thioacetamide-induced chronic liver injury (Chen et al, 2022; Hong et al, 2023; Huang et al, 2006). Thus, the role of cilia in liver fibrosis needs further investigation. In addition, it remains to be investigated whether cilia are present on other cell types in the liver.

The formation and maintenance of cilia are regulated by a number of proteins including intraflagellar transport (IFT) proteins (Klena and Pigino, 2022; Nachury and Mick, 2019). Especially, IFT88 is a key ciliary protein, and *Ift88*-knockout mice are frequently used for the study of ciliary functions (Nishimura et al, 2019). In the present study, we report that primary cilia exist on multiple cell types in the liver, but only cilia present on HSCs disassemble during liver fibrosis. Our data reveal the downregulation of IFT88 and the disruption of ciliary homeostasis during HSC activation and liver fibrosis. Our data also show that TGF-β promotes the ubiquitination of IFT88 by X-linked inhibitor of apoptosis (XIAP), resulting in IFT88 degradation and ciliary disassembly. In addition, we demonstrate that XIAP-mediated downregulation of IFT88 in HSCs exacerbates liver fibrosis. These findings thus indicate that targeting the XIAP–IFT88 axis may represent a novel therapeutic strategy for the management of liver fibrosis.

## Results

### Disruption of ciliary homeostasis and downregulation of IFT88 during HSC activation and liver fibrosis

**Figure 1:**
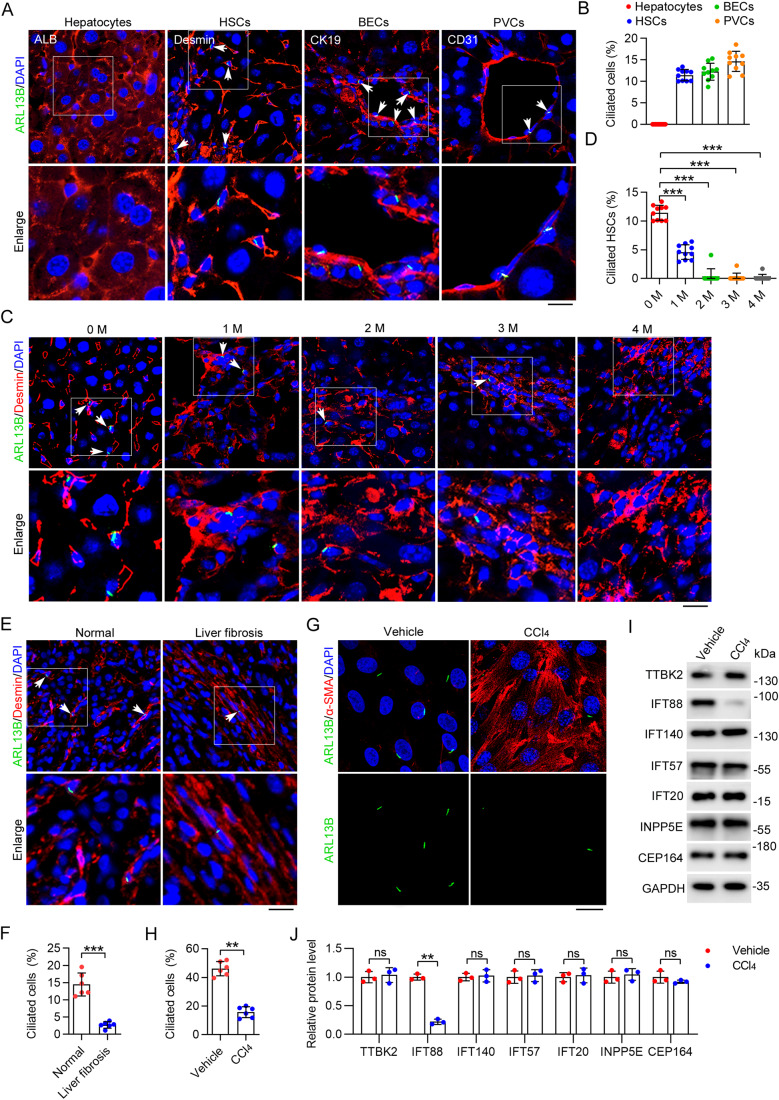
Primary cilia on HSCs disassemble during CCl_4_-induced liver fibrosis. (**A**) Immunofluorescence images of liver tissues from C57BL/6 mice stained with antibodies against ARL13B and ALB, desmin, CK19, or CD31. Nuclei were stained with DAPI (blue). Scale bar, 10 μm. (**B**) Quantification of the percentage of ciliated cells in hepatocytes, HSCs, BECs, and PVCs (*n* = 10 mice). To quantify the percentage of ciliated cells, >200 cells from six fields were analyzed for each mouse. (**C**, **D**) Immunofluorescence images (**C**) and quantification of the percentage of ciliated cells in HSCs (**D**) from CCl_4_-treated mice at the indicated time (*n*  = 10 mice). To quantify the percentage of ciliated cells (**D**), >200 cells from six fields were analyzed for each mouse. Scale bar, 10 μm. (**E**, **F**) Immunofluorescence images (**E**) and quantification of HSC cilia (**F**) of healthy and fibrotic human livers (*n* = 6 samples). To quantify the percentage of ciliated cells (**E**), >1000 cells from 10 sections were analyzed for each sample. Scale bar, 20 µm. (**G**, **H**) Immunofluorescence images (**G**) and quantification of the density of cilia (**H**) in primary mouse HSCs isolated from corn oil (vehicle) or CCl_4_-treated mice (*n*  =  6 independent experiments). To quantify the percentage of ciliated cells (**H**), >120 cells were analyzed for each experiment. Scale bar, 10 µm. (**I**, **J**) Immunoblotting (**I**) and quantification (**J**) of ciliary proteins in primary HSCs isolated from vehicle- or CCl_4_-treated mice for 2 months (*n* = 3 mice). Data information: Data are presented as mean ± SD. Statistical significance was determined by one-way ANOVA with post hoc tests (**D**) or unpaired two-tailed Student’s *t* test (**F**, **H**, **J**). ns not significant; **P < 0.01, ***P < 0.001. See also Figs. EV1 and EV2. Source data are available online for this figure.

To investigate the distribution of primary cilia in the liver, we stained the mouse liver tissue with antibodies against ADP ribosylation factor like GTPase 13B (ARL13B), a ciliary marker (Hor and Goh, 2019), and albumin (ALB, a marker of hepatocytes), desmin (a marker of HSCs), cytokeratin 19 (CK19, a marker of BECs), or cluster of differentiation 31 (CD31, a marker of hepatic portal vein cells (PVCs)). Consistent with previous studies, we observed the presence of primary cilia on BECs, whereas no cilia were detected on hepatocytes (Fig. 1A,B). Interestingly, we also observed the presence of primary cilia on HSCs and PVCs (Fig. 1A,B).

**Figure EV1:**

The mouse model of CCl_4_-induced liver fibrosis. (**A**, **B**) Immunofluorescence images (**A**) and quantification (**B**) of the α-SMA positive areas in the liver of mice treated with CCl_4_ for 0–4 months (*n* = 6 mice). Scale bar, 20 μm. (**C**, **D**) Representative images of Sirius red staining and H&E staining in the liver of C57BL6 mice treated with CCl_4_ for 2 months (**C**). The Image J software was used to quantify the collagen-positive areas (**D**), *n* = 6 mice). Scale bars for Sirius red staining and H&E staining, 200 μm. Scale bar for liver, 1 cm. (**E**, **F**) The activities of AST (**E**) and ALT (**F**) in the serum were analyzed in CCl_4_-treated mice for 2 months (*n* = 4 mice). Data information: Data are presented as mean ± SD. Statistical significance was determined by unpaired two-tailed Student’s *t* test. ns not significant; **P < 0.01, ***P < 0.001. Related to Fig. 1. Source data are available online for this figure.

**Figure EV2:**

Persistent presence of primary cilia on BECs and PVCs during CCl_4_-induced liver fibrosis. (**A**–**C**) Immunofluorescence images of primary cilia on hepatocytes (A), BECs (**B**), and PVCs (**C**) from CCl_4_-treated mice (*n*  =  10 mice). Scale bars, 10 μm. (**D**) Quantification of the percentage of ciliated cells in hepatocytes, PVCs, and BECs from mice described in (**A**–**C**) (*n*  =  10 mice). To quantify the percentage of ciliated cells (**D**), >200 cells from six fields were analyzed for each mouse. The same group of mice (*n* = 10 mice for each time point) were used for Fig. 1A–D and Fig. EV2. Data information: Data are presented as mean ± SD. Statistical significance was determined by one-way ANOVA. ns not significant. Related to Fig. 1. Source data are available online for this figure.

To analyze ciliary dynamics during the process of liver fibrosis, we used the CCl_4_-induced mouse model. The level of α-SMA increased gradually in the liver of mice injected with CCl_4_ (Fig. EV1A,B). Sirius red staining and hematoxylin and eosin (H&E) staining of the liver showed an apparent accumulation of collagen upon CCl_4_ treatment (Fig. EV1C,D). In addition, the activities of aspartate aminotransferase (AST) and alanine aminotransferase (ALT) in the serum were significantly increased in CCl_4_-treated mice (Fig. EV1E,F), indicating serious liver fibrosis.

By immunofluorescence microscopy, we found that only the number of cilia on HSCs was reduced during liver fibrosis (Fig. 1C,D), whereas the numbers of cilia on hepatocytes, BECs, and PVCs did not change (Fig. EV2). Consistent with these findings, immunofluorescence microscopy revealed a substantial reduction in the number of HSC cilia in patients with liver fibrosis compared to healthy individuals (Fig. 1E,F). Furthermore, we observed a significant decrease in the number of cilia in primary HSCs isolated from CCl_4_-treated mice in comparison to those from untreated mice (Fig. 1G,H). To explore the mechanism underlying the disruption of ciliary homeostasis in HSCs, we examined the levels of a number of key ciliary proteins, including tau tubulin kinase 2 (TTBK2), IFT88, IFT140, IFT57, IFT20, inositol polyphosphate-5-phosphatase E (INPP5E), and centrosomal protein 164 (CEP164), in primary HSCs isolated from mice treated with or without CCl_4_. The results revealed a significant decrease in the level of IFT88 in response to CCl_4_ treatment, whereas no other proteins showed significant alterations (Fig. 1I,J). These findings suggest a potential involvement of IFT88 downregulation in the disruption of HSC ciliary homeostasis during liver fibrosis.

### Ablation of HSC cilia exacerbates CCl_4_-induced liver fibrosis in mice

**Figure 2:**
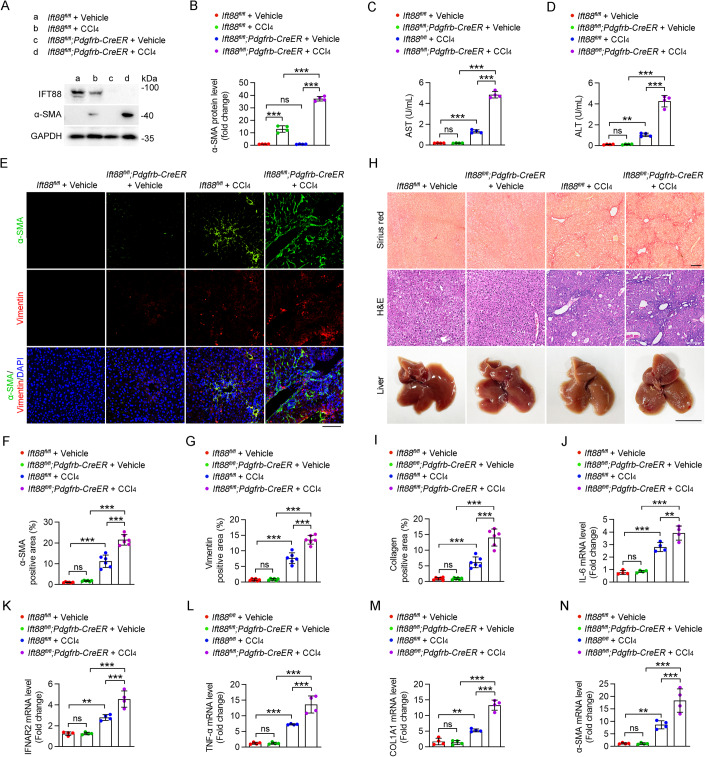
HSC-specific IFT88 deficiency exacerbates CCl_4_-induced liver fibrosis. (**A**, **B**) Immunoblotting (**A**) and quantification (**B**) of the levels of IFT88 and α-SMA in HSCs isolated from CCl_4_- or corn oil (vehicle)-treated *Ift88*^*fl/fl*^ and *Ift88*^*fl/fl*^*;Pdgfrb-CreER* mice for 1 month (*n* = 4 mice). (**C**, **D**) Examination of AST (**C**) and ALT (**D**) activities in the serum of *Ift88*^*fl/fl*^ and *Ift88*^*fl/f*^*;Pdgfrb-CreER* mice treated with CCl_4_ or vehicle for 1 month (*n* = 4 mice). (**E**–**G**) Immunofluorescence images (**E**) and quantification of the levels of α-SMA (**F**) and vimentin (**G**) in the liver of *Ift88*^*fl/fl*^ and *Ift88*^*fl/fl*^*;Pdgfrb-CreER* mice treated with CCl_4_ or vehicle for 1 month (*n* = 6 mice). Nuclei were stained with DAPI (blue). Scale bar, 100 μm. (**H**, **I**) CCl_4_-induced liver fibrosis in *Ift88*^*fl/fl*^ and *Ift88*^*fl/fl*^*;Pdgfrb-CreER* mice was examined with Sirius red staining and H&E staining (**H**), and the percentage of collagen-positive areas was quantified (**I**) (*n* = 6 mice). Scale bars for Sirius red staining and H&E staining, 200 μm. Scale bar for liver, 1 cm. (**J**–**N**) The mRNA levels of IL-6 (**J**), IFNAR2 (**K**), TNF-α (**L**), COL1A1 (**M**), and α-SMA (**N**) in liver tissues were measured by quantitative RT-PCR (*n* = 4 mice). Data information: Data are presented as mean ± SD. Statistical significance was determined by two-way ANOVA with post hoc tests. ns not significant; **P < 0.01, ***P < 0.001. See also Figs. EV3 and EV4. Source data are available online for this figure.

Next, we sought to determine the role of HSC cilia in the development of liver fibrosis. *Ift88*^*fl/fl*^ mice were crossed with the *Pdgfrb-Cre/ERT2* line to produce *Ift88*^*fl/fl*^*;Pdgfrb-Cre/ERT2* mice. By administrating tamoxifen to the *Ift88*^*fl/fl*^*;Pdgfrb-Cre/ERT2* mice, HSC-specific *Ift88*-knockout mice were achieved (Fig. 2A). Immunoblotting analysis indicated that the loss of IFT88 in HSCs remarkably increased the ability of CCl_4_ to induce α-SMA production (Fig. 2A,B). Furthermore, in response to CCl_4_ treatment, the AST and ALT activities in the serum of HSC-specific *Ift88*-knockout mice were elevated to a significantly higher level compared to *Ift88*^*fl/fl*^ mice (Fig. 2C,D). Consistently, immunofluorescence microscopy analysis of α-SMA and vimentin, as well as Sirius red staining of liver sections, both demonstrated that loss of IFT88 in HSCs significantly enhanced CCl_4_-induced liver fibrosis (Fig. 2E–I).

**Figure EV3:**

Whole-body deficiency in IFT88 exacerbates CCl_4_-induced liver fibrosis over a 2-month period. (**A**–**C**) Immunofluorescence images (**A**) and quantification of the levels of α-SMA (**B**) and vimentin (**C**) in the liver of *Ift88*^*fl/fl*^ and *Ift88*^*fl/fl*^*;Ubc-CreER* mice treated with CCl_4_ or corn oil (vehicle) for 2 months (*n* = 6 mice). Nuclei were stained with DAPI (blue). Scale bar, 50 μm. (**D**, **E**) CCl_4_-induced liver fibrosis in *Ift88*^*fl/fl*^ and *Ift88*^*fl/fl*^*;Ubc-CreER* mice was examined with Sirius red staining and H&E staining (**D**), and the percentage of collagen-positive areas was quantified (**E**) (*n* = 6 mice). Scale bars for Sirius red staining and H&E staining, 200 μm. Scale bar for liver, 1 cm. (**F**, **G**) Examination of the activities of AST (**F**) and ALT (**G**) in the serum of *Ift88*^*fl/fl*^ and *Ift88*^*fl/fl*^*;Ubc-CreER* mice (*n* = 4 mice). (**H**–**L**) The mRNA levels of IL-6 (H), IFNAR2 (**I**), TNF-α (**J**), COL1A1 (**K**), and α-SMA (**L**) in liver tissues were measured by quantitative RT-PCR (*n* = 4 mice). (**M**, **N**) Immunoblotting (**M**) and quantification (**N**) of the levels of IFT88 and α-SMA in the liver of *Ift88*^*fl/fl*^ and *Ift88*^*fl/fl*^*;Ubc-CreER* mice treated with CCl_4_ or vehicle (*n* = 4 mice). Data information: Data are presented as mean ± SD. Statistical significance was determined by two-way ANOVA with post hoc tests. ns not significant; *P < 0.05, **P < 0.01, ***P < 0.001. Related to Fig. 2. Source data are available online for this figure.

**Figure EV4:**

Whole-body deficiency in IFT88 exacerbates CCl_4_-induced liver fibrosis over a 4-month period. (**A**–**C**) Immunofluorescence images (**A**) and quantification of α-SMA (**B**) and vimentin (**C**) in the liver of *Ift88*^*fl/fl*^ and *Ift88*^*fl/fl*^*;Ubc-CreER* mice treated with CCl_4_ or corn oil (vehicle) for 4 months (*n* = 6 mice). Scale bar, 50 μm. (**D**, **E**) CCl_4_-induced liver fibrosis in *Ift88*^*fl/fl*^ and *Ift88*^*fl/fl*^*;Ubc-CreER* mice for 4 months was evaluated with Sirius red staining and H&E staining (**D**). The Image J software was used for the quantification of collagen-positive areas (**E**) (*n* = 6 mice). Scale bars for Sirius red staining and H&E staining, 200 μm. Scale bar for liver, 1 cm. (**F**, **G**) The activities of AST (**F**) and ALT (**G**) in the serum were analyzed in *Ift88*^*fl/fl*^ and *Ift88*^*fl/fl*^*;Ubc-CreER* mice (*n* = 4 mice). (**H**–**L**) The mRNA levels of IL-6 (**H**), IFNAR2 (**I**), TNF-α (**J**), COL1A1 (**K**), and α-SMA (**L**) in liver tissues were measured by quantitative RT-PCR (*n* = 4 mice). Data information: Data are presented as mean ± SD. Statistical significance was determined by two-way ANOVA with post hoc tests. ns, not significant; *P < 0.05, **P < 0.01, ***P < 0.001. Related to Fig. 2. Source data are available online for this figure.

Quantitative RT-PCR analysis was performed to determine the mRNA levels of interleukin-6 (IL-6), interferon α and β receptor subunit 2 (IFNAR2), and tumor necrosis factor-α (TNF-α), collagen type 1 α1 chain (COL1A1) and α-SMA. The results showed that upon CCl_4_ treatment, HSC-specific *Ift88*-knockout mice exhibited a greater increase in the mRNA levels of all profibrotic factors in comparison to *Ift88*^*fl/fl*^ mice (Fig. 2J–N). Similar results were observed in an inducible whole-body *Ift88*-knockout mouse model, *Ift88*^*fl/fl*^*;Ubc-Cre/ERT2* (Figs. EV3 and EV4). Taken together, these results demonstrate the essential role of HSC cilia in restricting the progression of liver fibrosis.

### IFT88 undergoes proteasome-dependent degradation during HSC activation

**Figure 3:**
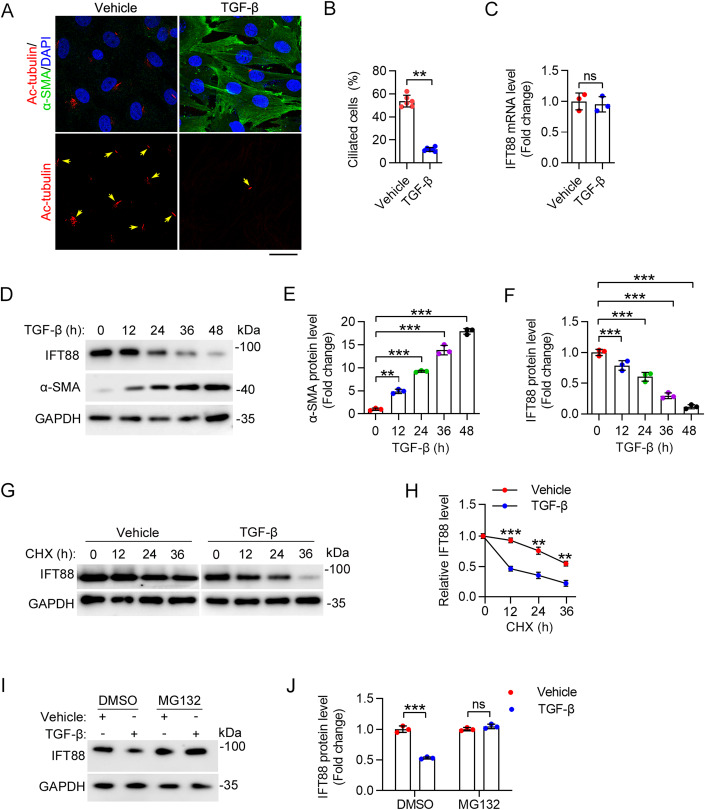
TGF-β promotes IFT88 degradation during primary HSC activation. (**A**, **B**) Immunofluorescence images (**A**) and quantification of cilia (**B**) in primary mouse HSCs treated with or without TGF-β for 24 h (*n* = 6 independent experiments). To quantify the percentage of ciliated cells (**B**), >120 cells were analyzed for each experiment. Scale bar, 10 µm. (**C**) HSCs were treated as described in (**A**). IFT88 mRNA level was measured by quantitative RT-PCR (*n* = 3 independent experiments). (**D**–**F**) Immunoblotting (**D**) of IFT88 and α-SMA in primary mouse HSCs treated with TGF-β, and the levels of IFT88 (**E**) and α-SMA (**F**) were quantified by densitometry (*n* = 3 independent experiments). (**G**, **H**) The effect of TGF-β on the half-life of IFT88 in primary mouse HSCs treated with CHX (20 mg/mL) was examined by immunoblotting (**G**), and the protein half-life curves were obtained (**H**) (*n* = 3 independent experiments). (**I**, **J**) Primary mouse HSCs were treated with TGF-β for 24 h and then treated with MG132 (5 mM) for 12 h. The levels of IFT88 and GAPDH were examined by immunoblotting (**I**), and the level of IFT88 was determined by densitometry (**J**) (*n* = 3 independent experiments). Data information: Data are presented as mean ± SD. Statistical significance was determined by one-way ANOVA with post hoc tests (**E**, **F**) or unpaired two-tailed Student’s *t* test (**B**, **C**, **H**, **J**). ns not significant; **P < 0.01, ***P < 0.001. See also Fig. EV5. Source data are available online for this figure.

**Figure EV5:**
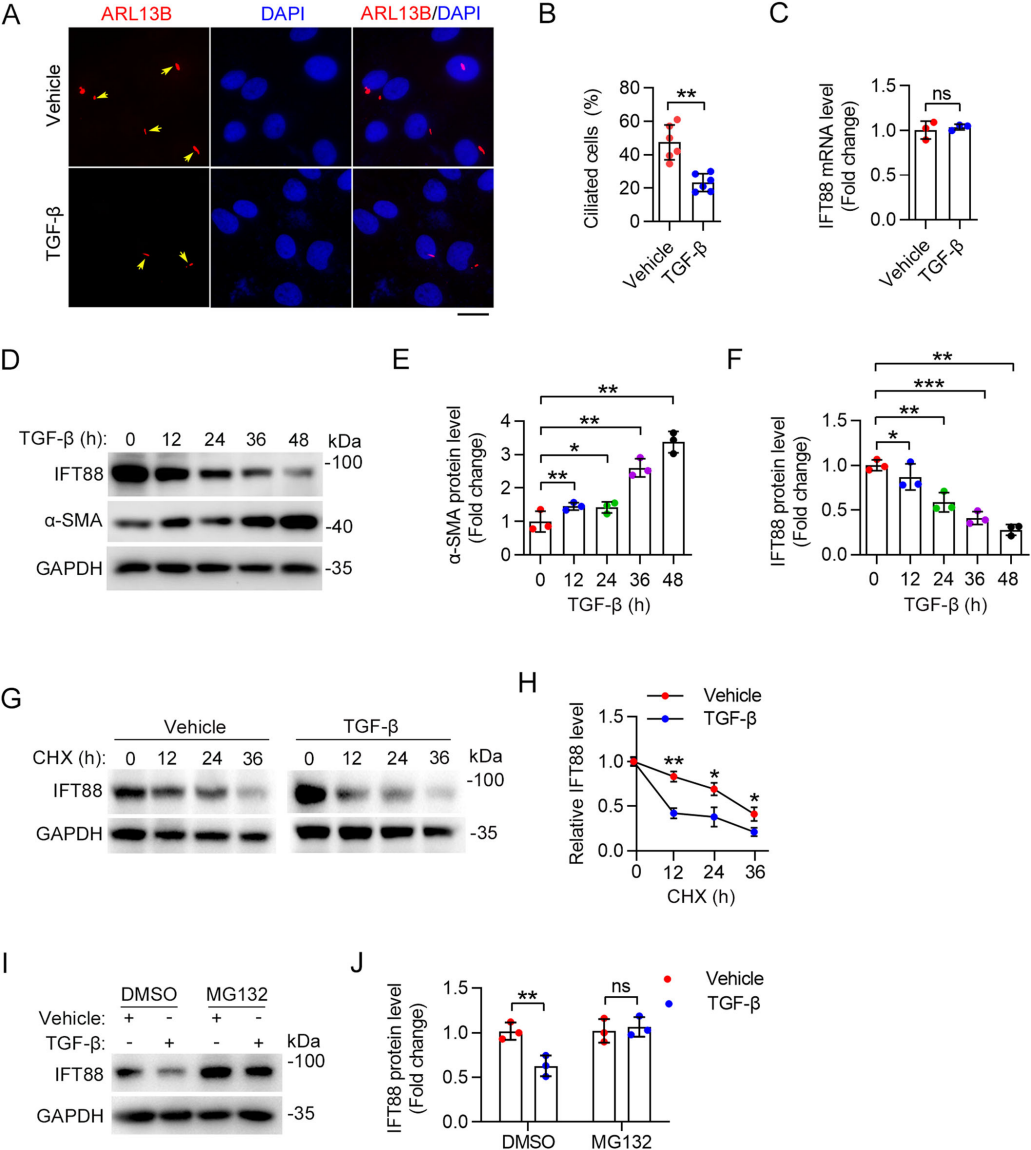
TGF-β promotes IFT88 degradation during LX-2 cell activation. (**A**, **B**) Immunofluorescence images (**A**) and quantification of the density of cilia (**B**) in LX-2 cells treated with TGF-β for 24 h (*n*  =  6 independent experiments). To quantify the percentage of ciliated cells (**B**), >140 cells were analyzed for each experiment. Scale bar, 10 µm. (**C**) IFT88 mRNA expression was measured by quantitative RT-PCR after TGF-β treatment for 24 h in LX-2 cells (*n* = 3 independent experiments). (**D**–**F**) Immunoblotting (**D**) of IFT88 and α-SMA in LX-2 cells treated with TGF-β, and the levels of IFT88 (**E**) and α-SMA (**F**) were quantified by densitometry (*n* = 3 independent experiments). (**G**, **H**) The effect of TGF-β on the half-life of IFT88 in LX-2 cells treated with CHX (20 mg/mL) was examined by immunoblotting (**G**), and the protein half-life curves were obtained (**H**) (*n* = 3 independent experiments). (**I**, **J**) LX-2 cells were treated with TGF-β for 24 h and then treated with MG132 (5 mM) for 12 h. The levels of IFT88 and GAPDH were examined by immunoblotting (**I**), and the level of IFT88 was determined by densitometry (**J**) (*n* = 3 independent experiments). Data information: Data are presented as mean ± SD. Statistical significance was determined by one-way ANOVA with post hoc tests (**E**, **F**) or unpaired two-tailed Student’s *t* test (**B**, **C**, **H**, **J**). ns not significant; *P < 0.05, **P < 0.01, ***P < 0.001. Related to Fig. 3. Source data are available online for this figure.

We then studied the molecular mechanism underlying the downregulation of IFT88 upon activation of HSCs. Immunofluorescence microscopy revealed a dramatic loss of cilia upon TGF-β-induced activation of primary mouse HSCs (Fig. 3A,B). Quantitative RT-PCR analysis revealed that the IFT88 mRNA level in primary HSCs was not affected by TGF-β treatment (Fig. 3C). Immunoblotting revealed that TGF-β treatment increased the level of α-SMA in primary mouse HSCs in a time-dependent manner, confirming HSC activation (Fig. 3D,E). Consistent with the results obtained in CCl_4_-treated mice, the IFT88 protein level was gradually decreased during TGF-β-induced primary HSC activation (Fig. 3D–F). To determine whether the IFT88 protein stability is affected by TGF-β treatment, we blocked de novo protein synthesis using cycloheximide (CHX) in primary HSCs. By immunoblotting, we found that the half-life of IFT88 was much shorter after TGF-β treatment (Fig. 3G,H). In addition, the inhibitory effect of TGF-β on the IFT88 protein level could be reversed by the treatment of cells with the proteasome inhibitor MG132 (Fig. 3I,J). Similar results were also observed in human LX-2 cell lines (Fig. EV5). These results indicate that IFT88 undergoes proteasome-dependent degradation during HSC activation.

### IFT88 interacts with the E3 ubiquitin ligase XIAP

**Figure 4:**
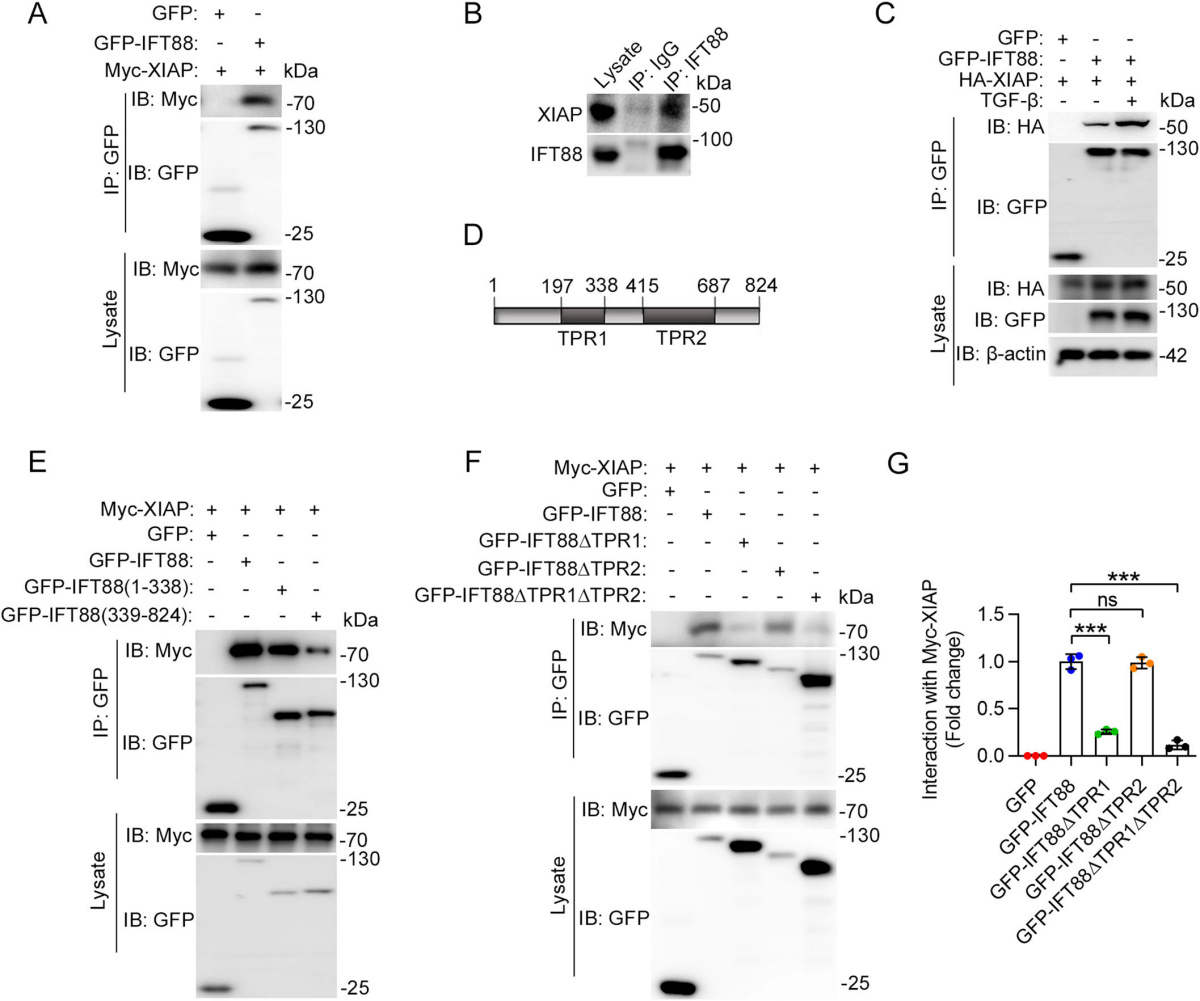
TGF-β promotes the interaction between IFT88 and XIAP. (**A**) Immunoprecipitation and immunoblotting showing the interaction between GFP-IFT88 and Myc-XIAP in LX-2 cells. (**B**) Immunoprecipitation and immunoblotting showing the interaction between endogenous IFT88 and XIAP. (**C**) Immunoprecipitation and immunoblotting showing that the interaction between IFT88 and XIAP was increased by TGF-β treatment for 24 h. (**D**) Schematic diagram of IFT88 showing the TPR1 and TPR2 domains. (**E**) Identification of the domains mediating the interaction between XIAP and IFT88 in cells transfected with Myc-XIAP and different truncated forms of IFT88 tagged with GFP. (**F**, **G**) Immunoprecipitation and immunoblotting (**F**) and quantification (**G**) showing that the TPR1 domain of IFT88 is critical for its interaction with XIAP in LX-2 cells (*n* = 3 independent experiments). Data information: Data are presented as mean ± SD. Statistical significance was determined by one-way ANOVA with post hoc tests. ns not significant; ***P < 0.001. See also Appendix Figs. S1 and  S2. Source data are available online for this figure.

To further study the mechanism underlying IFT88 degradation, we sought to identify proteins that interact with IFT88. GFP-tagged IFT88 was overexpressed in LX-2 cells and immunoprecipitated with a GFP antibody, and IFT88-interacting proteins were identified by mass spectrometry (Appendix Fig. S1A). Two E3 ubiquitin ligases, XIAP and HUW domain containing E3 ubiquitin ligase 1 (HUWE1), were identified in the IFT88 immunoprecipitate, and XIAP exhibited the highest score among these proteins (Appendix Fig. S1B). To confirm the interaction between IFT88 and XIAP, we performed immunoprecipitation using LX-2 cells. We found that Myc-XIAP interacted with GFP-IFT88 (Fig. 4A). Similar results were also observed in HEK293 human embryonic kidney epithelial cells (Appendix Fig. S2A). Immunoprecipitation also revealed an interaction between endogenous XIAP and IFT88 in LX-2 cells (Fig. 4B). Furthermore, the interaction between IFT88 and XIAP was enhanced by TGF-β treatment (Fig. 4C).

We then used a series of truncation mutants of IFT88 to determine the domains that mediate its interaction with XIAP. IFT88 has two tetratricopeptide repeat (TPR) motifs, i.e., TPR1 (amino acids 197–338) and TPR2 (amino acids 415–687) (Fig. 4D). Immunoprecipitation revealed that the TPR1-containing amino-terminal fragment (amino acids 1–338) had a stronger interaction with XIAP than the TPR2-containing carboxyl-terminal fragment (amino acids 339–824) (Fig. 4D,E). Moreover, the interaction between XIAP and IFT88 was significantly reduced by the removal of TPR1 (ΔTPR1) or both TPR1 and TPR2 (ΔTPR1ΔTPR2), but not TPR2 (ΔTPR2) (Fig. 4F,G). Similar results were also observed in HEK293 cells (Appendix Fig. S2B,C). These results suggest that the TPR1 domain of IFT88 is critical for its interaction with XIAP.

### XIAP mediates IFT88 ubiquitination in a TGF-β-responsive manner

**Figure 5:**
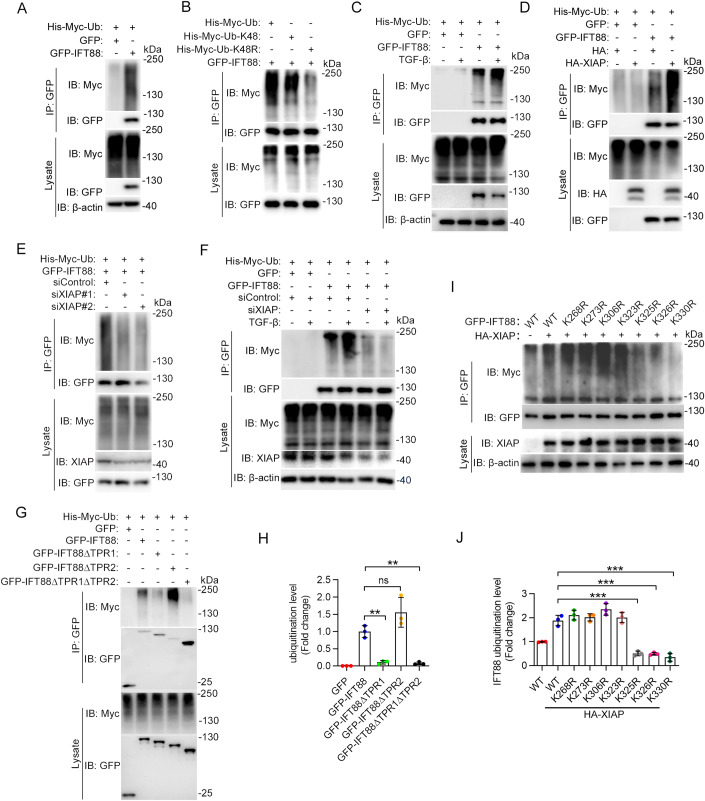
TGF-β promotes XIAP-mediated ubiquitination of IFT88. (**A**) Immunoprecipitation and immunoblotting to examine IFT88 ubiquitination in LX-2 cells transfected with GFP-vector or GFP-IFT88. (**B**) Examination of IFT88 ubiquitination in HEK293 cells transfected with His-Myc-ubiquitin (Ub), His-Myc-ubiquitin-K48, or His-Myc-ubiquitin-K48R. (**C**) Examination of IFT88 ubiquitination in LX-2 cells with or without TGF-β treatment. (**D**) Analysis of IFT88 ubiquitination in LX-2 cells with or without HA-XIAP overexpression. (**E**) Examination of IFT88 ubiquitination in LX-2 cells transfected with control or XIAP siRNAs. (**F**) Analysis of IFT88 ubiquitination in LX-2 cells transfected with His-Myc-ubiquitin and GFP-IFT88, together with control or XIAP siRNAs, and treated with or without TGF-β. (**G**, **H**) Immunoblotting (**G**) and densitometric quantification (**H**) of the domains responsible for IFT88 ubiquitination by XIAP (*n* = 3 independent experiments). HEK293 cells were transfected with various truncated forms of GFP-IFT88. (**I**, **J**) HEK293 cells were transfected with GFP-IFT88 or the indicated lysine-to-arginine mutants together with HA-XIAP. Immunoprecipitation and immunoblotting were then performed (**I**), and the level of IFT88 ubiquitination was quantified (**J**) (*n* = 3 independent experiments). Data information: Data are presented as mean ± SD. Statistical significance was determined by one-way ANOVA with post hoc tests. ns not significant; **P < 0.01, ***P < 0.001. See also Appendix Fig. S3. Source data are available online for this figure.

We then sought to study whether XIAP mediates IFT88 ubiquitination. Immunoblotting revealed the presence of IFT88 polyubiquitination in LX-2 cells (Fig. 5A). To investigate the linkage specificity for IFT88 ubiquitination, we used a couple of ubiquitin mutants. The lysine 48 (K48)-linked ubiquitin chain is well known to play a major role in mediating the proteasomal degradation of proteins (Pla-Prats and Thoma, 2022). Thus, we used the K48 mutant of ubiquitin, which contains only a single lysine residue (lysine 48) with all of the other lysines mutated to arginines, and the K48R mutant, in which only lysine 48 is mutated to arginine (Ran et al, 2020). We found that the K48R mutant of ubiquitin significantly inhibited IFT88 ubiquitination, whereas the K48 mutant showed an effect similar to wild-type ubiquitin (Fig. 5B), indicating that IFT88 undergoes K48-linked polyubiquitination. We further found that the ubiquitination of IFT88 was significantly increased by TGF-β treatment (Fig. 5C).

By immunoprecipitation and immunoblotting, we found that the level of IFT88 ubiquitination was increased by XIAP overexpression and decreased by siRNA-mediated knockdown of XIAP expression in LX-2 cells (Fig. 5D,E). Similar results were observed in HEK293 cells (Appendix Fig. S3A,B), suggesting that XIAP functions as an E3 ubiquitin ligase for IFT88. In addition, we found that the ability of TGF-β to increase IFT88 ubiquitination was largely abolished by XIAP siRNAs (Fig. 5F), suggesting a specific effect of TGF-β on XIAP-mediated IFT88 ubiquitination. We further found that the TPR1 domain of IFT88 was required for its ubiquitination by XIAP (Fig. 5G,H). This result is consistent with our finding that the TPR1 domain mediates the interaction of IFT88 with XIAP. To identify the XIAP-specific ubiquitination sites in IFT88, we individually mutated the seven lysine residues in the TPR1 domain to arginines. Compared to wild-type IFT88, the K325R, K326R, and K330R mutants exhibited a significantly decreased ubiquitination (Fig. 5I,J). This result suggests that K325, K326, and K330 are the major residues ubiquitinated by XIAP.

### Blocking XIAP-mediated IFT88 ubiquitination suppresses HSC activation

**Figure 6:**
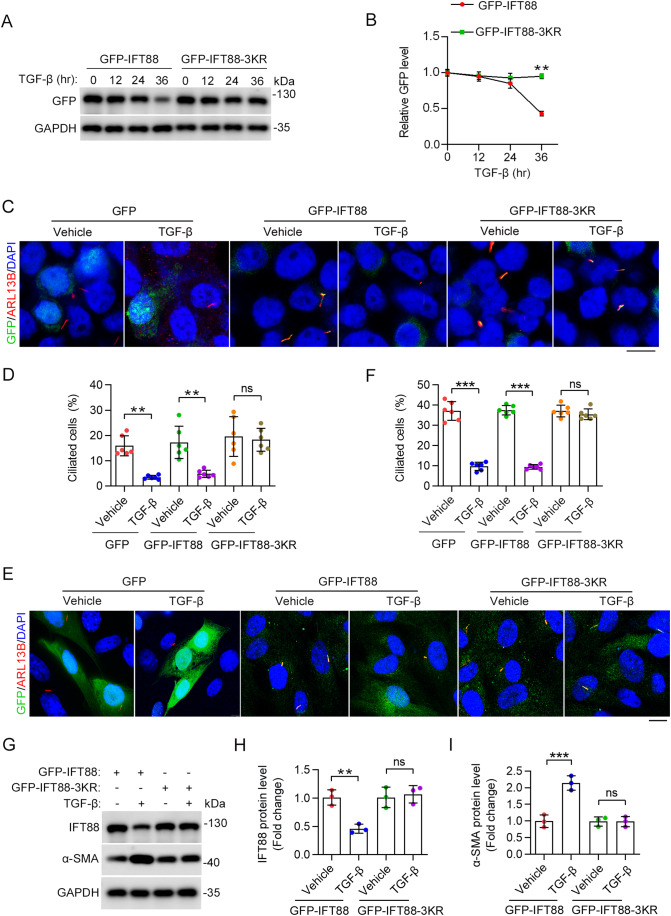
Effect of XIAP-mediated IFT88 ubiquitination on TGF-β-induced HSC activation. (**A**, **B**) Immunoblotting (**A**) and quantification (**B**) of GFP-IFT88 levels in LX-2 cells expressing GFP-IFT88 or GFP-IFT88-3KR (*n* = 3 independent experiments). Cells were exposed to TGF-β for the indicated time. (**C**, **D**) Immunofluorescence images (**C**) and quantification of the percentage of ciliated cells (**D**) in LX-2 cells transfected with the indicated plasmids with or without TGF-β treatment for 24 h (*n* = 6 independent experiments). To quantify the percentage of ciliated cells (**D**), >140 cells were analyzed for each experiment. Scale bar, 15 µm. (**E**, **F**) Immunofluorescence images (**E**) and quantification of the percentage of ciliated cells (**F**) in primary mouse HSCs overexpressed with indicated proteins and treated with or without TGF-β for 24 h (*n* = 6 independent experiments). To quantify the percentage of ciliated cells (**F**), >140 cells were analyzed for each experiment. Scale bar, 15 µm. (**G**–**I**) Immunoblotting (**G**) and quantification of the levels of IFT88 (**H**) and α-SMA (**I**) in LX-2 cells transfected with plasmids encoding GFP-IFT88 or GFP-IFT88-3KR for 48 h (*n* = 3 independent experiments). Data information: Data are presented as mean ± SD. Statistical significance was determined by unpaired two-tailed Student’s *t* test (**B**) or two-way ANOVA with post hoc tests (**D**, **F**, **H**, **I**). ns not significant; **P < 0.01, ***P < 0.001. Source data are available online for this figure.

We next evaluated whether IFT88 ubiquitination at K325, K326, and K330 has a functional relevance to HSC activation. We constructed a 3KR mutant of IFT88, in which K325, K326, and K330 are mutated to arginines. We found that this mutant was resistant to TGF-β-induced degradation compared to wild-type IFT88 (Fig. 6A,B). In addition, the 3KR mutant, but not wild-type IFT88, was able to restore TGF-β-induced ciliary defects (Fig. 6C–F). Collectively, these results suggest that XIAP-mediated ubiquitination of IFT88 at K325, K326, and K330 promotes its proteasomal degradation, leading to the disruption of ciliary homeostasis.

To investigate a potential role for XIAP-mediated ubiquitination of IFT88 in the activation of HSCs, we examined the effects of wild-type IFT88 and the 3KR mutant on TGF-β-induced α-SMA expression in LX-2 cells. By immunoblotting, we found that the IFT88-3KR mutant significantly prevented the induction of α-SMA expression by TGF-β treatment (Fig. 6G–I). This result thus suggests that XIAP-mediated IFT88 ubiquitination and proteasomal degradation contribute to HSC activation during liver fibrosis.

## Discussion

Liver fibrosis is a converging point for more serious hepatic diseases, such as cirrhosis and hepatocellular carcinoma (Li et al, 2022a). Interestingly, liver fibrosis is also a common complication observed in several ciliopathies, such as polycystic kidney disease, Meckel syndrome, nephronophthisis, and Joubert syndrome, and is manifested in several cilium-defective animal models, indicating a potential role for cilia in the process of liver fibrosis (Dillard et al, 2018; Stayner et al, 2017; Van De Weghe et al, 2017). However, the molecular basis for liver fibrosis is not fully understood, and the major ciliary proteins contributing to the fibrogenic process remain to be identified. In the present study, we reveal a disruption of ciliary homeostasis during HSC activation and liver fibrosis, and uncover a significant decrease of a key ciliary factor, IFT88, upon profibrotic stimulation. Moreover, we identify XIAP as an E3 ubiquitin ligase for IFT88 ubiquitination and proteasomal degradation, and establish a critical function for the XIAP–IFT88 axis in HSC activation and liver fibrosis.

BECs have been demonstrated previously to contain primary cilia, which extend from the cell membrane to the bile duct lumen (Frassetto et al, 2018; Masyuk et al, 2008; Ogawa et al, 2021). These cilia act as mechanic, osmotic, and chemical sensors in intrahepatic BECs and are associated with increased BEC proliferation within liver cysts (Chen et al, 2022; Mansini et al, 2019). Defects in ciliary structures or functions are associated with a number of liver diseases (Devane et al, 2022; Masyuk et al, 2022; Shaheen et al, 2020). However, there is evidence that ablating BEC cilia does not promote liver fibrosis (Chen et al, 2022). In this study, we demonstrate that primary cilia are also present on PVCs and HSCs in the healthy liver. During HSC activation and liver fibrosis, the cilia on HSCs disassemble, whereas those on PVCs and BECs are stable, indicating that disruption of ciliary homeostasis in HSCs is specifically associated with liver fibrosis. Importantly, ablating cilia by depleting IFT88 sensitizes mice to CCl_4_-induced liver fibrosis. Therefore, we speculate that the primary cilia present on HSCs may play an important role in inhibiting liver fibrosis. It is important to note that the above evidence does not rule out the possibility that the loss of cilia in other cell types could also contribute to liver fibrosis. Future research utilizing additional animal models, such as methionine- and choline-deficient (MCD) diet and bile duct ligation, will help determine whether disruption of primary cilia represents a universal mechanism of HSC activation across different types of liver fibrosis.

HSCs reside in the perisinusoidal space and exhibit a dormant phenotype under physiological conditions (Lua et al, 2016). HSCs are activated to become myofibroblasts in response to liver injury or inflammatory cytokines (Devaraj et al, 2022; Tsuchida and Friedman, 2017). Proliferating myofibroblasts are the major source of ECM molecules, such as type I and III collagens, that constitute the pathological fibrous tissue. Thus, activation of HSCs is regarded as a key event in liver fibrosis (Tsuchida and Friedman, 2017). Our study shows that HSCs with ablated cilia produce more α-SMA and ECMs, promoting liver fibrosis. Primary cilia are known to sense and transmit extracellular signals to regulate various cellular processes important for tissue development and homeostasis (Nachury and Mick, 2019; Nishimura et al, 2019). The sensory function of cilia is dependent on the coordinated regulation of specific receptors and associated signal molecules localized in cilia (Nachury and Mick, 2019). For example, the hedgehog (Hh) pathway is a typical cilium-dependent pathway that regulates a variety of biological processes (Cruz et al, 2022; Schmidt et al, 2022). Especially, the Hh pathway plays an important role in maintaining the proliferative, non-differentiated state of hepatocytes and its dysregulation has been implicated in liver fibrosis (Davey et al, 2014; Machado and Diehl, 2018). Whether the disruption of ciliary homeostasis in HSCs participates in liver fibrosis through the Hh pathway thus merits further investigation.

IFT88 is a key ciliary protein essential for mammalian development (Bowie and Goetz, 2020; Li et al, 2022b). However, little is known about the molecular mechanisms that regulate the IFT88 level. This study shows that IFT88 is selectively downregulated during liver fibrosis, contributing to the specific loss of cilia on HSCs. Our data also reveal that XIAP binds and ubiquitinates IFT88, resulting in its proteasomal degradation. XIAP is known to promote tumor growth and progression by inhibiting cell death pathways via its E3 ubiquitin ligase activity (Jost and Vucic, 2020). We show that XIAP catalyzes the K48-linked polyubiquitination of IFT88 at K325, K326, and K330. Mutation of these three lysines to arginines can prevent ciliary loss and HSC activation during liver fibrosis, implying a potential for blocking IFT88 ubiquitination in the intervention of the fibrogenic process. Given that multiple anti-XIAP drugs have been developed for the treatment of various diseases (Jost and Vucic, 2020), further studies are warranted to examine their therapeutic potential in liver fibrosis.

## Methods

### Animals

HSC-specific or whole-body *Ift88*-knockout mice were generated by intraperitoneal injection of 6-week-old *Ift88*^*fl/fl*^*;Pdgfrb-Cre/ERT2* or *Ift88*^*fl/fl*^*;Ubc-Cre/ERT2* mice with 100 μL of 20 mg/mL tamoxifen daily for 5 consecutive days as described previously (Yang et al, 2021). To induce liver fibrosis, the mice were intraperitoneally injected with CCl_4_ (1.0 mL/kg body weight, 1:4 dissolved in corn oil) twice a week. All applicable institutional and/or national guidelines for the care and use of animals were followed. The use of mice was approved by the Animal Care and Use Committee of Nankai University (2019-SYDWLL-000001).

### Clinical samples

All human liver tissues were obtained from the tissue bank of Tianjin Medical University General Hospital. The use of clinical samples was approved by the Medical Ethics Committee of Tianjin Medical University General Hospital, in accordance with the relevant provisions of the Ethical Review of Biomedical Research Involving Human Beings of the National Health Commission.

### Reagents

CCl_4_ (C805329) and corn oil (C805618) were purchased from Macklin. The H&E staining kit (G1120) and Sirius red staining kit (G1471) were purchased from Solarbio. TGF-β (100-21) was from PeproTech. CHX (HY12320) was from MedChemExpress. MG132 (M7449) was from Sigma-Aldrich. The primary antibodies used in this study are as follows: rabbit anti-α-SMA (Abcam, ab5694; IF 1:500), rabbit anti-vimentin (Abcam, ab92547; IF 1:500), mouse anti-IFT88 (Proteintech, 13967-1-AP; IB 1:1000, IP 1:1000), mouse anti-ALB (Proteintech, 66051-1-lg; IF 1:1000), mouse anti-Desmin (Abcam, ab6322; IF 1:1000), mouse anti-CK19 (Abcam, ab254186; IF 1:1000), rabbit anti-CD31 (Abcam, ab28364; IF 1:1000), mouse anti-ARL13B (Proteintech, 17711-1-AP; IF 1:1000), rabbit anti-TTBK2 (Proteintech, 15072-1-AP; IB 1:1000), rabbit anti-IFT140 (Proteintech, 17460-1-AP; IB 1:3000), rabbit anti-IFT57 (Proteintech, 11083-1-AP; IB 1:1000), rabbit anti-IFT20 (Proteintech, 13615-1-AP; IB 1:800), rabbit anti-INPP5E (Proteintech, 17797-1-AP; IB 1:1000), rabbit anti-CEP164 (Proteintech, 22227-1-AP; IB 1:500), mouse anti-XIAP (Proteintech, 10037-1-lg; IB 1:1000), rabbit anti-GAPDH (Abways, AB0036; IB 1:2000), rabbit anti-β-actin (Abways, AB0035; IB 1:2000), rabbit anti-GFP (Abways, AB0045; IF 1:2,000; IB 1:1000), mouse anti-Myc tag (AC3966-2MG, Sigma-Aldrich; IB 1:1000), mouse anti-HA tag (Abways, AB0004; IB 1:1000), mouse anti-Flag (Abways, AB0008; IB 1:1000), and mouse anti-His (Abways, AB0002; IB 1:1000). Alexa Fluor 488- and 568-conjugated secondary antibodies were purchased from Life Technologies.

### Cell culture and transfection

Primary mouse HSCs were isolated from normal or fibrotic livers (for the CCl_4_ model, all isolations from the CCl_4_ model were done 2 days after the last injection of CCl_4_) using pronase-collagenase perfusion as previously described (Mederacke et al, 2015). Primary mouse HSC, LX-2 (ATCC), and HEK293 cells (ATCC) were cultured in DMEM supplemented with 10% FBS and 1% penicillin/streptomycin, and cultured at 37 °C with 5% CO_2_. To induce cilium formation, primary mouse HSC or LX-2 cells were cultured in serum-free medium for 48 h. Plasmids and siRNAs were transfected into cells using lipofectamine 3000 and lipofectamine RNAiMAX (Thermo Fisher Scientific), respectively. The mammalian expression plasmids for XIAP and IFT88 were constructed by PCR, and the plasmids for ubiquitin were described previously (Ran et al, 2020). Control siRNA (5′-CGUACGCGGAAUACUUCGA-3′), IFT88 siRNAs (#1: 5′-CGAAGCACUUAACACUUA-3′; #2: 5′-CUGAAACUUCACGCAAUCC-3′), and XIAP siRNAs (#1: 5′-AAGUGGUAGUCCUGUUUCAGC-3′; #2: 5′-GUGCUGGACUCUACUACACUU-3) were synthesized by RiboBio.

### Histopathology and immunofluorescence microscopy

For histopathology, human or mouse liver samples were fixed with 4% paraformaldehyde for 24 h, dehydrated with 30% sucrose for 24 h, and then cryo-embedded in Tissue-Tek OCT (Sakura) on dry-ice slabs. Frozen sections were cut into 8 µm sections and subjected to H&E or Sirius red staining as described previously (Ran et al, 2022). For immunofluorescence staining, mouse liver tissues were fixed in 4% paraformaldehyde at 4 °C overnight and then cryo-embedded in Tissue-Tek OCT on dry-ice slabs. Frozen sections were cut into 8 µm slices on a Leica CM1950 cryostat and mounted on Superfrost Plus slides (Thermo Fisher Scientific). The slides were fixed again with 4% paraformaldehyde for 20 min and permeabilized with 0.5% Triton X-100 in PBS for 20 min. The tissues were then blocked with 4% bovine serum albumin (BSA) for 1 h, incubated overnight with primary antibodies at 4 °C, and stained with secondary antibodies and DAPI (Sigma-Aldrich). For cultured cells, cells grown on glass coverslips were fixed with 4% paraformaldehyde for 20 min, washed with PBS, and blocked with 4% BSA at room temperature for 2 h. Cells were then incubated with primary antibodies for 2 h, followed by incubation with secondary antibodies for 1 h and stained with DAPI for 10 min as described (Zamith-Miranda et al, 2021). The length of cilia and the percentage of ciliated cells were measured with Image J (National Institutes of Health).

### Measurement of ALT and AST activities

The activities of ALT and AST in the serum were determined using the ALT activity kit (Solarbio, BC1555) and the AST activity kit (Solarbio, BC1565) following the manufacturer’s instructions.

### Immunoprecipitation and immunoblotting

The whole cell lysates containing a mixture of protease inhibitors (Thermo Fisher Scientific) were collected and centrifuged at 10,000× *g* for 10 min at 4 °C. Then, the lysates were incubated with agarose beads coated with primary antibodies at 4 °C for 4 h. The beads were washed four times and fractionated by SDS-PAGE followed by immunoblotting. For immunoblotting, the protein sample was separated by SDS-PAGE and transferred onto polyvinylidene difluoride membranes and then blocked with 5% fat-free milk at room temperature for 2 h and incubated with primary antibody overnight at 4 °C. After washing five times, the membrane was incubated with secondary antibodies at room temperature for 1 h. Protein signals were detected using immunoblotting detection reagents (Thermo Fisher Scientific). The experiment was repeated for at least three times, and the intensity of immunoblots was measured by densitometry using the Image J software (National Institutes of Health).

### Mass spectrometry

Mass spectrometry was performed by PTM Bio-Lab. The peptides were subjected to the NSI source followed by tandem mass spectrometry (MS/MS) in Q ExactiveTM Plus (Thermo) coupled online to the UPLC. The resulting MS/MS data were processed using Proteome Discoverer 1.3 tandem mass spectra. Peptide confidence was set to high, and peptide ion score was set to >20.

### Quantitative RT-PCR

Total RNA was extracted from the mouse liver and cultured cells according to the manufacturer’s instructions, and 1 µg RNA was reverse transcribed by the SuperScript III kit. Quantitative RT-PCR was then performed with the Power SYBR Green PCR Master Mix Kit (Applied Biosystems). GAPDH was used as the loading control, and the results were calculated with the 2^-ΔΔCt^ method. The primers used for qPCR are listed in Table EV1.

### Statistical analysis

All data were obtained from at least three independent experiments unless indicated otherwise. GraphPad Prism 8.0 (GraphPad Software) was used for statistical analysis. The significant difference between the two groups was analyzed using the unpaired two-tailed Student’s *t* test. One-way or two-way analysis of variance (ANOVA) was used for comparison between multiple conditions. Results are expressed as mean ± SD. Levels of statistical significance are indicated as follows: ns: not significant; *P  <  0.05; **P  <  0.01; ***P  <  0.001.

## Supplementary information

Appendix

Source Data Fig. 1

Source Data Fig. 2

Source Data Fig. 3

Source Data Fig. 4

Source Data Fig. 5

Source Data Fig. 6

Figure EV Source Data

Table EV1

Peer Review File

Expanded View Figures

## Data Availability

This study has not generated data that requires deposition in a public repository.
