# Supplementary material for: XIAP-mediated degradation of IFT88 disrupts HSC cilia to stimulate HSC activation and liver fibrosis
Source: EMBO Rep. 2024 Feb 13;25(3):12. doi: 10.1038/s44319-024-00092-y (PMC10933415; doi:10.1038/s44319-024-00092-y)
Supplement: Supplementary file 1 — Appendix [file 44319_2024_92_MOESM1_ESM.pdf]

Table of Content

Appendix Figure S1	Mass spectrometric identification of IFT88-interacting proteins.	Page 2
Appendix Figure S2	IFT88 interacts with the E3 ubiquitin ligase XIAP in HEK293 cells.	Page 3
Appendix Figure S3	XIAP mediates IFT88 ubiquitination in HEK293 cells.	Page 4

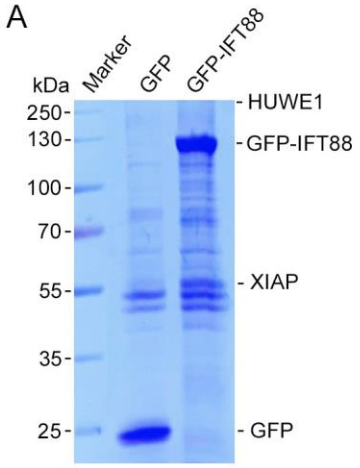

B

Protein	Score	Coverage	Unique peptides
**XIAP**	**212.03**	**17.30**	**7**
HUWE1	38.21	0.30	1

Appendix Figure S1. Mass spectrometric identification of IFT88-interacting proteins.

A. Coomassie blue staining of proteins immunoprecipitated from LX-2 cells with anti-GFP antibody.

B. E3 ubiquitin ligases identified by mass spectrometry as IFT88-interacting proteins.

Related to Fig 4.

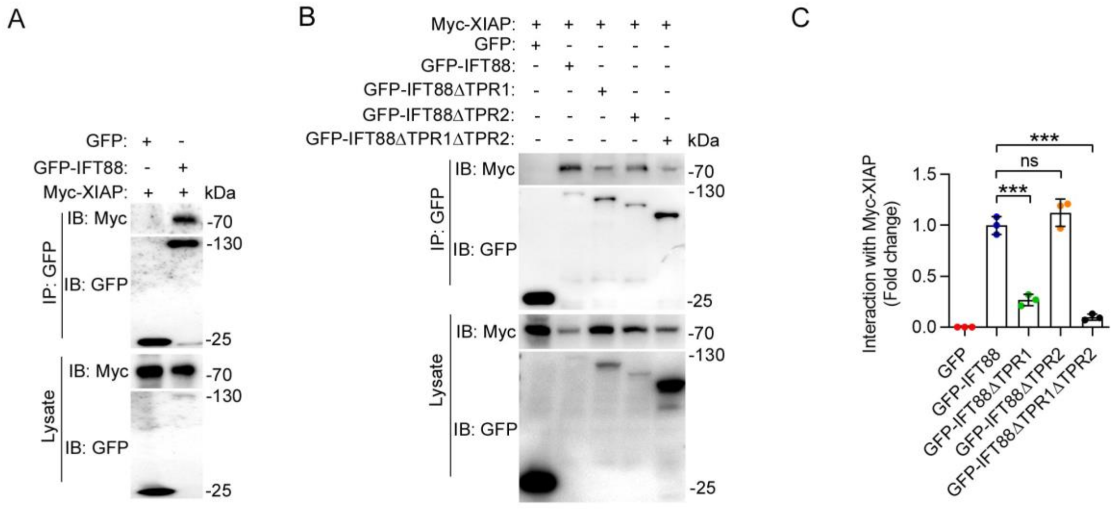

Appendix Figure S2. IFT88 interacts with the E3 ubiquitin ligase XIAP in HEK293 cells.

A. Analysis of IFT88 ubiquitination in HEK293 cells with or without HA-XIAP overexpression.

B, C. Examination of IFT88 ubiquitination in HEK293 cells transfected with control or XIAP siRNAs.

Data information: Data are presented as mean ± SD. Statistical significance was determined by one-way ANOVA with post-hoc tests. ns, not significant; ***$p < 0.001$. Related to Fig 4.

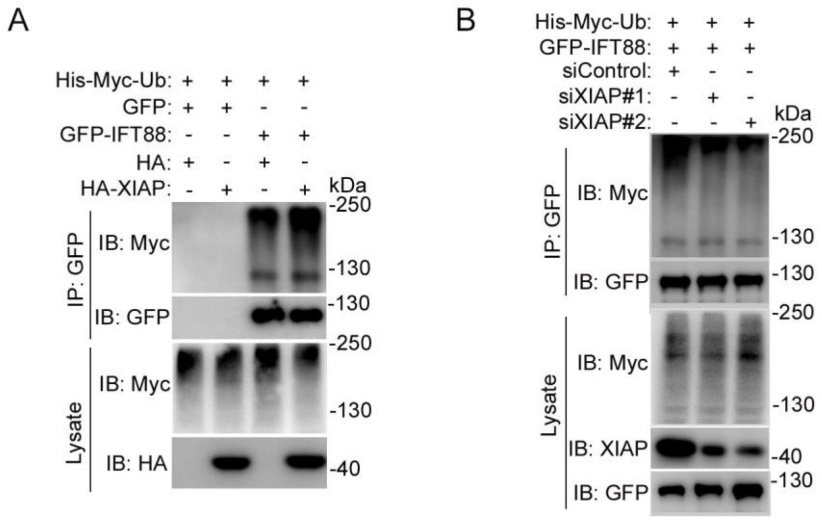

Appendix Figure S3. XIAP mediates IFT88 ubiquitination in HEK293 cells.
A. Analysis of IFT88 ubiquitination in HEK293 cells with or without HA-XIAP overexpression.
B. Examination of IFT88 ubiquitination in HEK293 cells transfected with control or XIAP siRNAs.
Related to Fig 5.
