# Supplementary figures and images for: XIAP-mediated degradation of IFT88 disrupts HSC cilia to stimulate HSC activation and liver fibrosis (part 1 of 2)
Source: EMBO Rep. 2024 Feb 13;25(3):12. doi: 10.1038/s44319-024-00092-y (PMC10933415; doi:10.1038/s44319-024-00092-y)

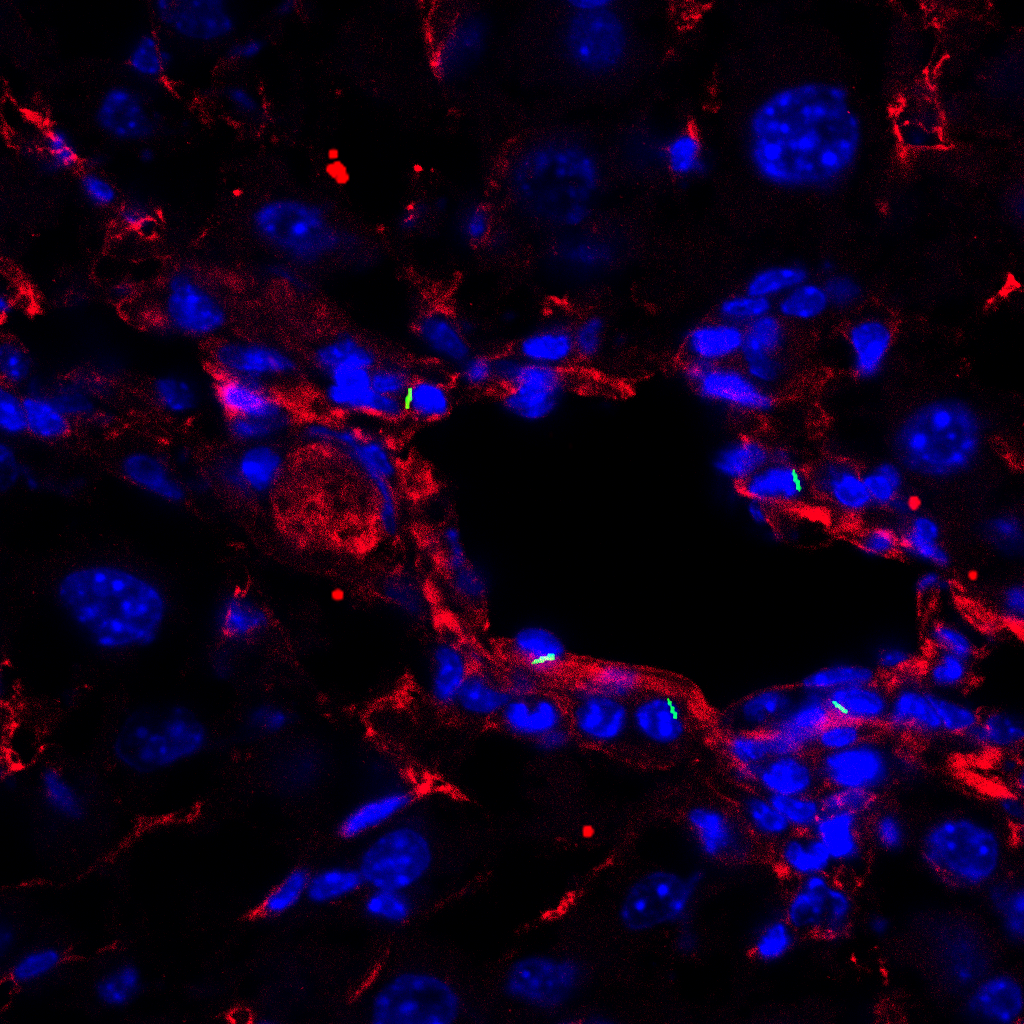

Supplement: Supplementary file 2 — Source Data Fig. 1 [file 44319_2024_92_MOESM2_ESM.zip › Figure 1A/Figure 1A BECs.tif]

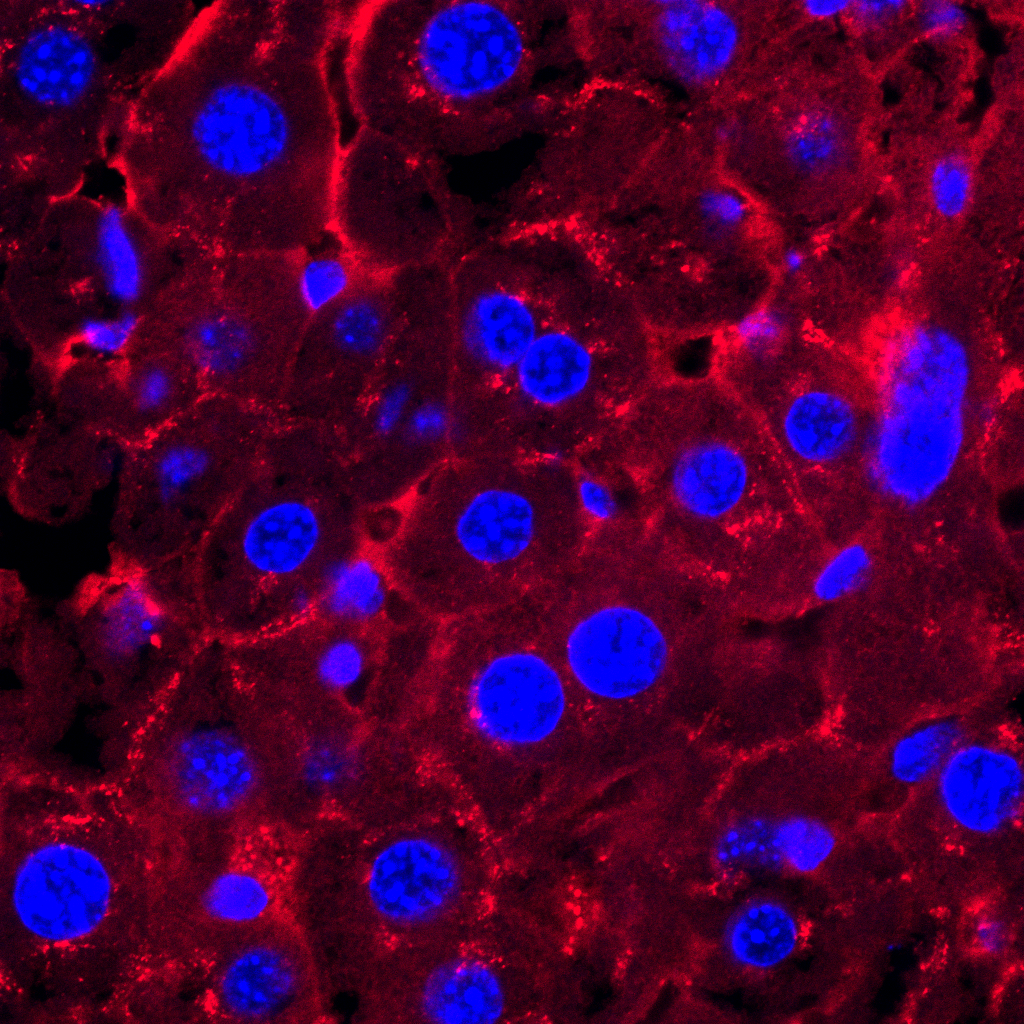

Supplement: Supplementary file 2 — Source Data Fig. 1 [file 44319_2024_92_MOESM2_ESM.zip › Figure 1A/Figure 1A hepatocytes .tif]

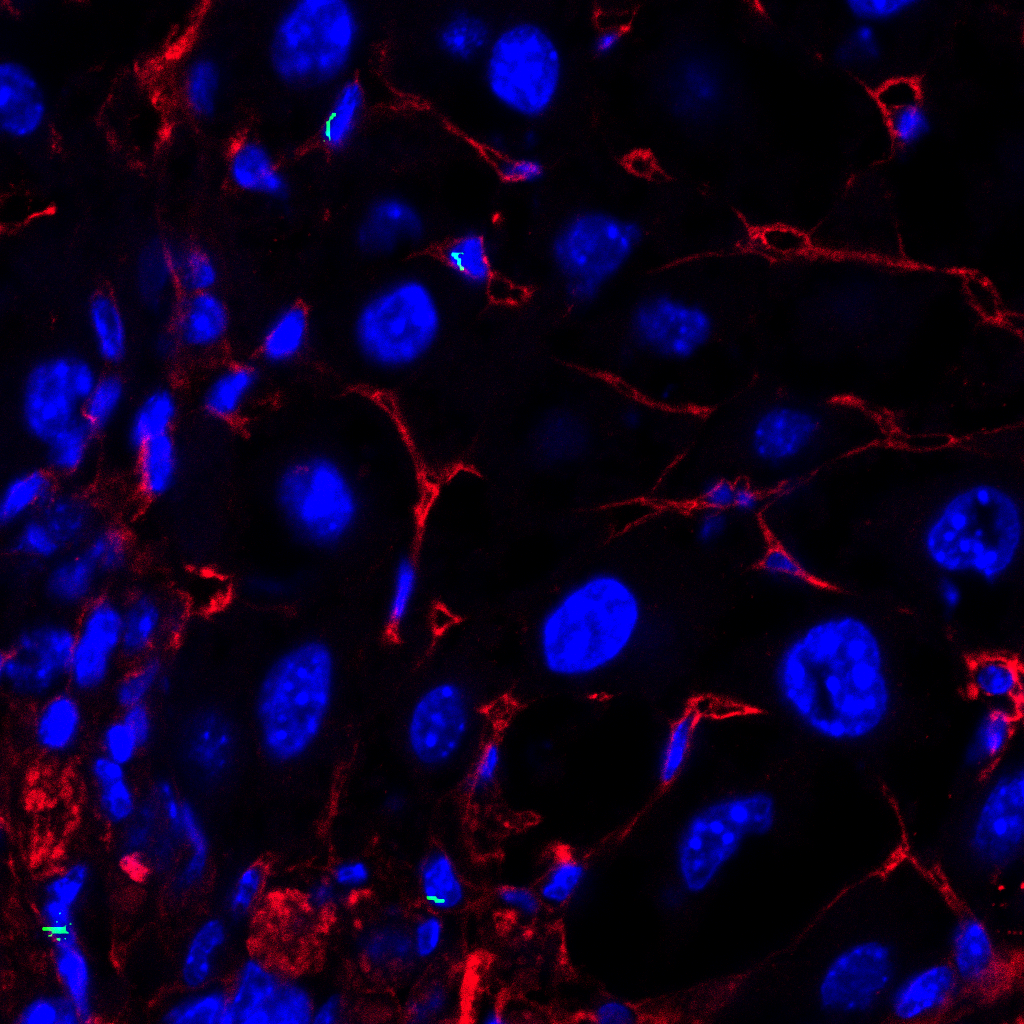

Supplement: Supplementary file 2 — Source Data Fig. 1 [file 44319_2024_92_MOESM2_ESM.zip › Figure 1A/Figure 1A HSCs.tif]

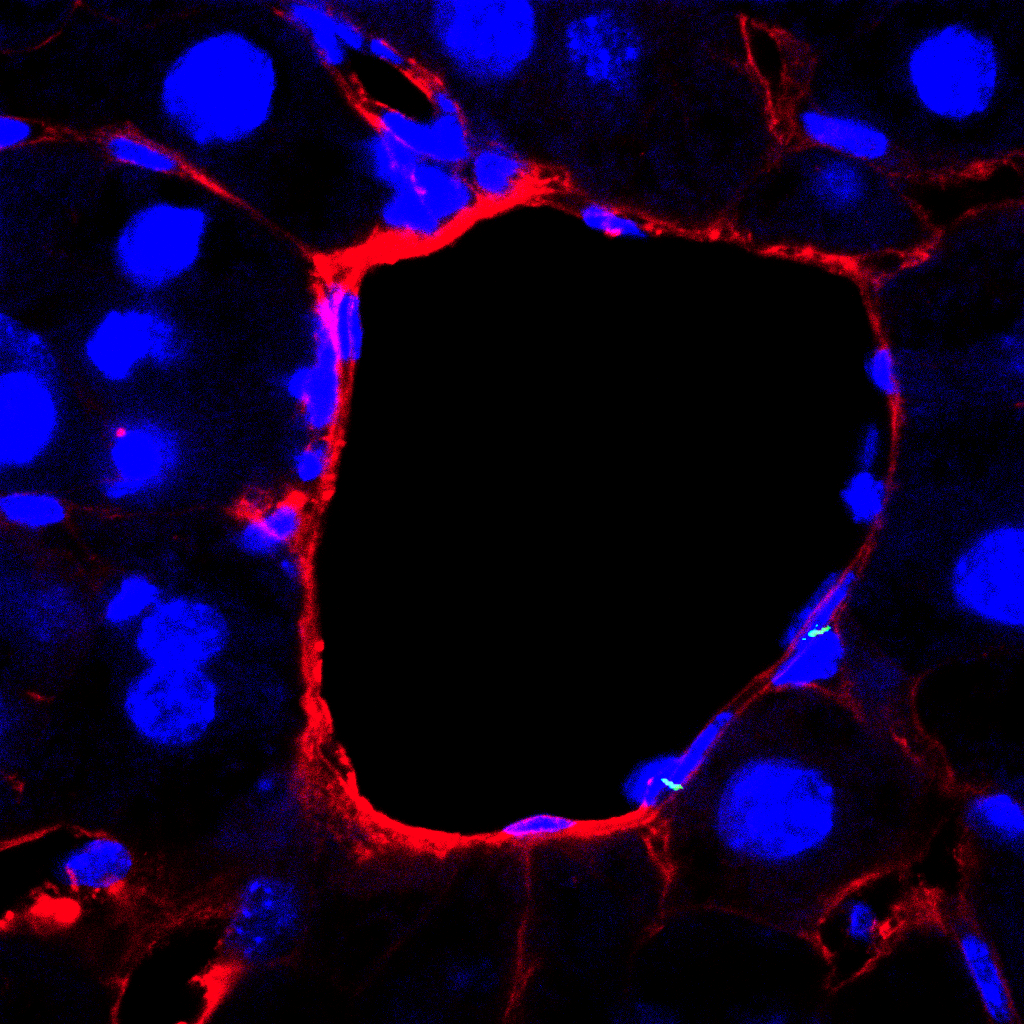

Supplement: Supplementary file 2 — Source Data Fig. 1 [file 44319_2024_92_MOESM2_ESM.zip › Figure 1A/Figure 1A PVCs.tif]

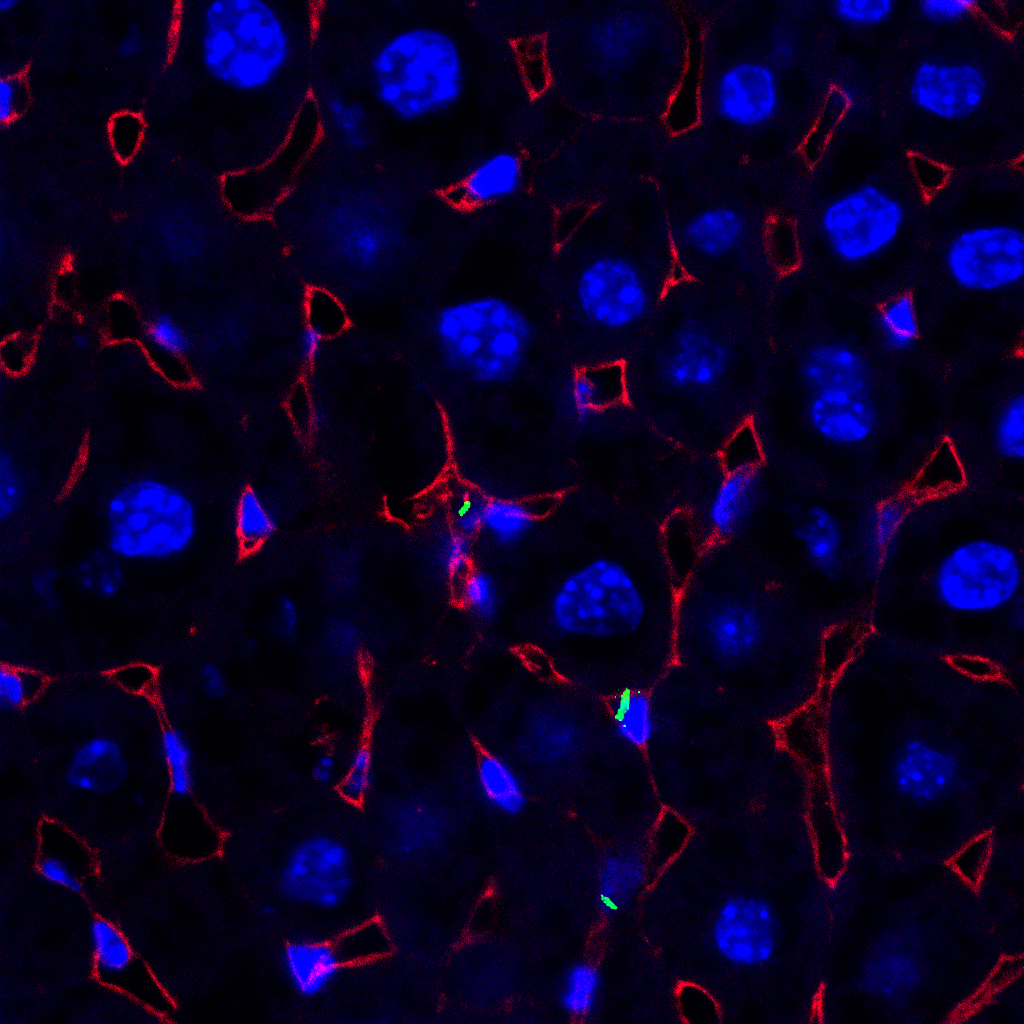

Supplement: Supplementary file 2 — Source Data Fig. 1 [file 44319_2024_92_MOESM2_ESM.zip › Figure 1C/Figure 1C 0M.tif]

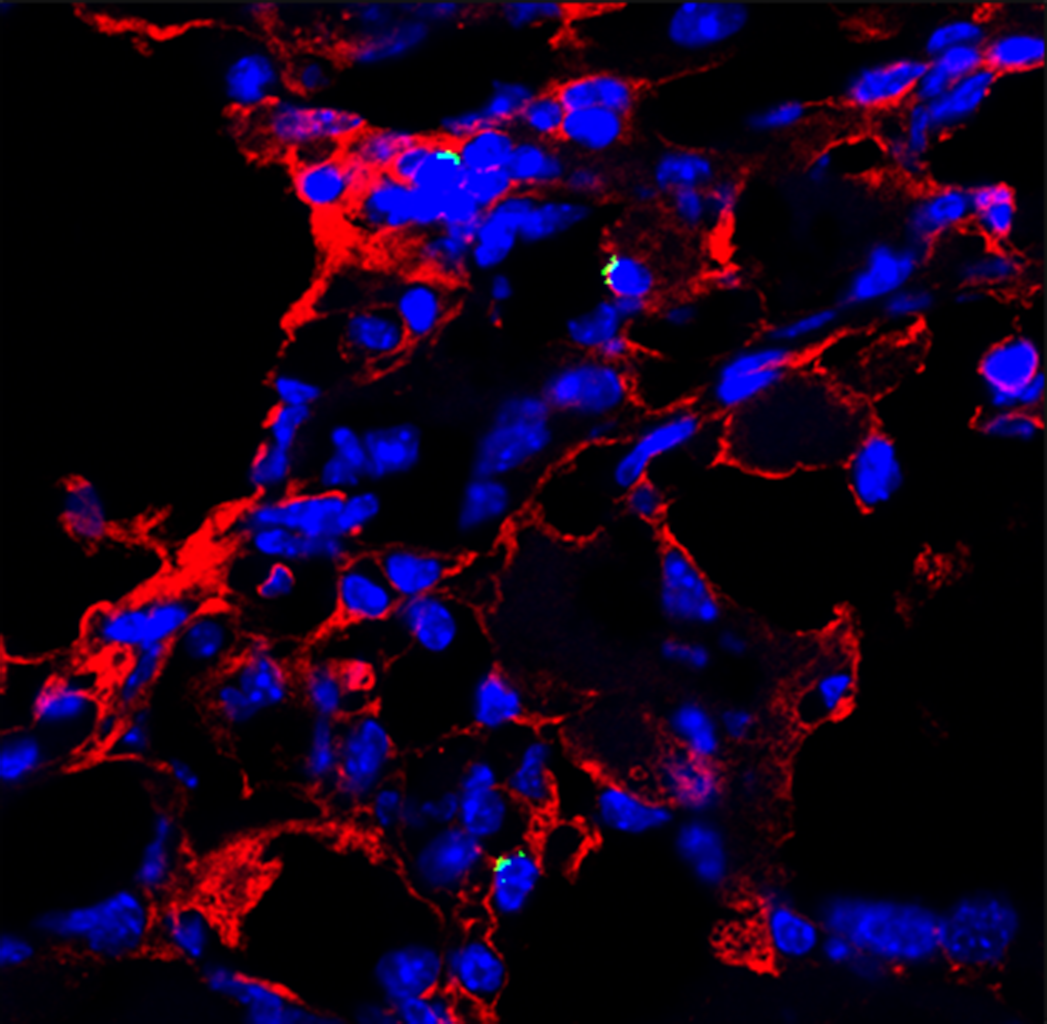

Supplement: Supplementary file 2 — Source Data Fig. 1 [file 44319_2024_92_MOESM2_ESM.zip › Figure 1C/Figure 1C 1M.tif]

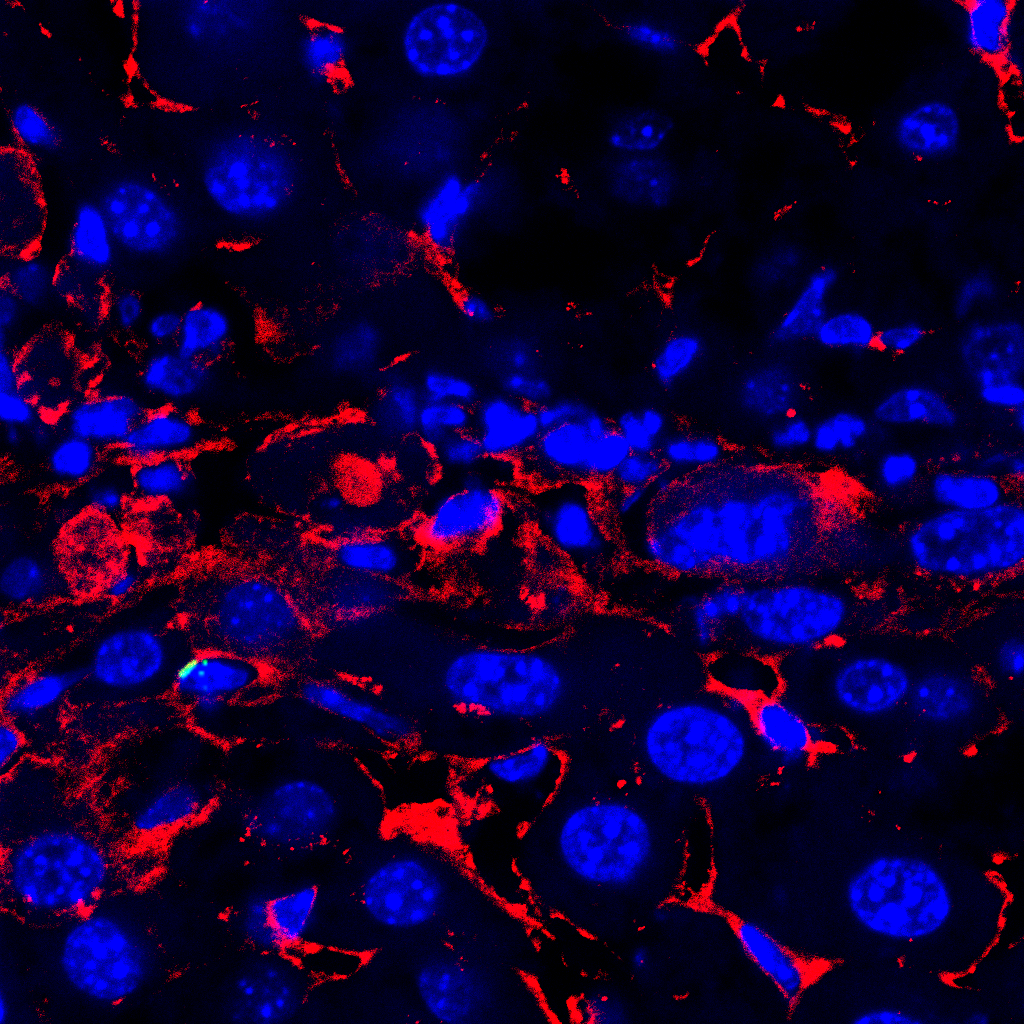

Supplement: Supplementary file 2 — Source Data Fig. 1 [file 44319_2024_92_MOESM2_ESM.zip › Figure 1C/Figure 1C 2M.tif]

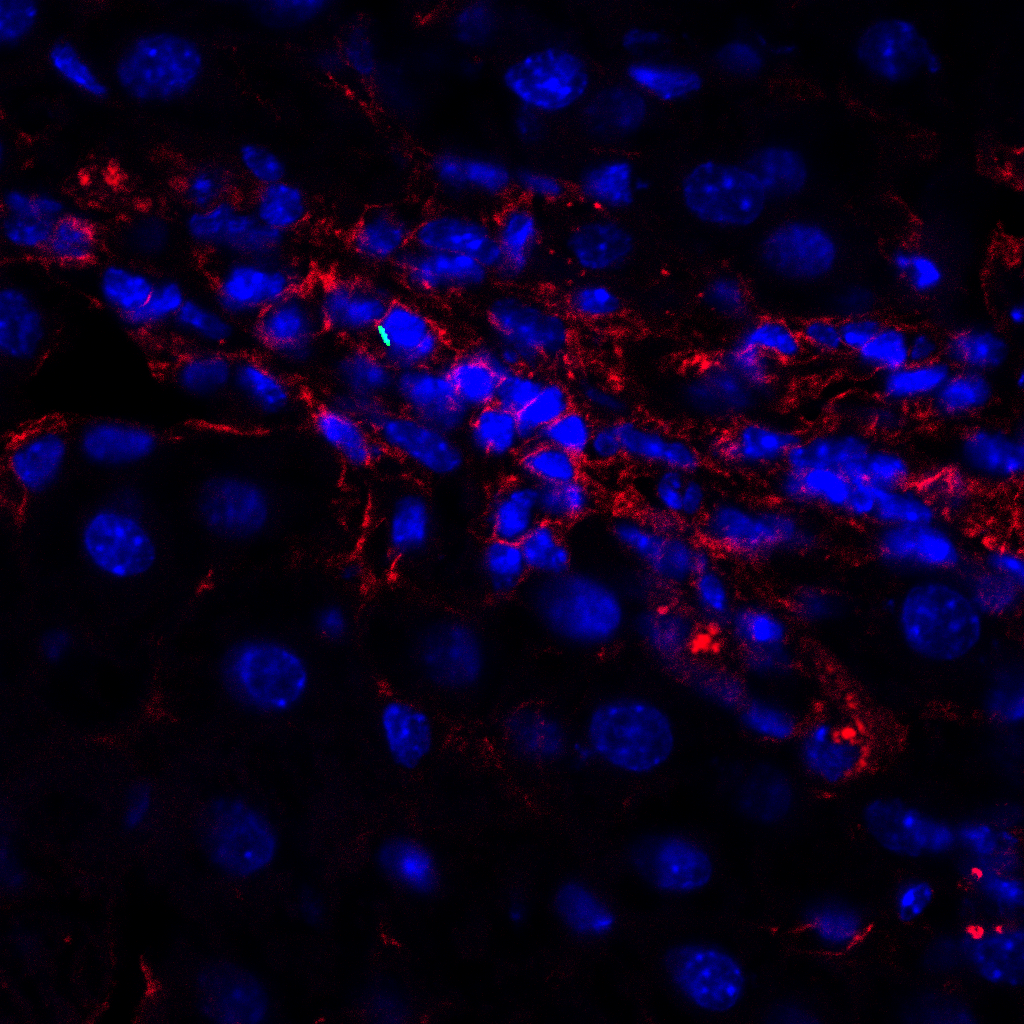

Supplement: Supplementary file 2 — Source Data Fig. 1 [file 44319_2024_92_MOESM2_ESM.zip › Figure 1C/Figure 1C 3M.tif]

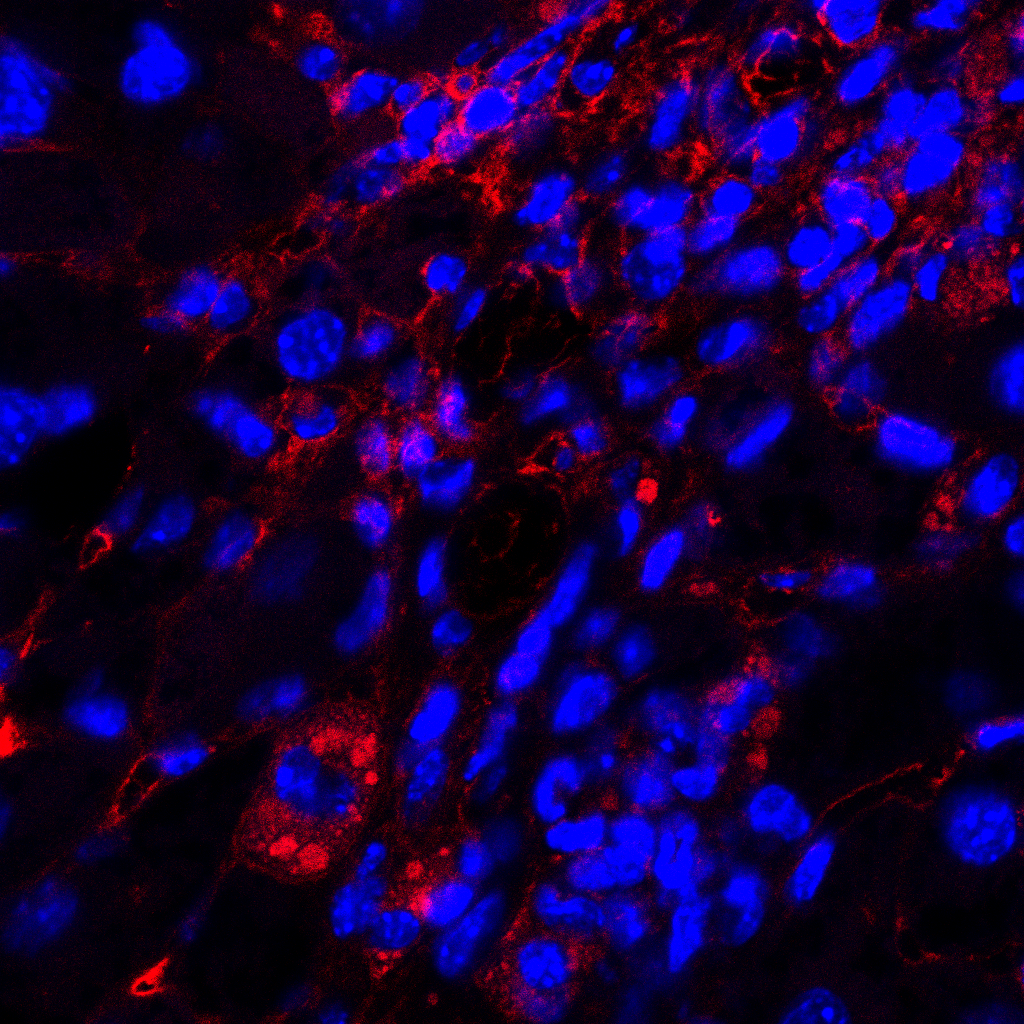

Supplement: Supplementary file 2 — Source Data Fig. 1 [file 44319_2024_92_MOESM2_ESM.zip › Figure 1C/Figure 1C 4M.tif]

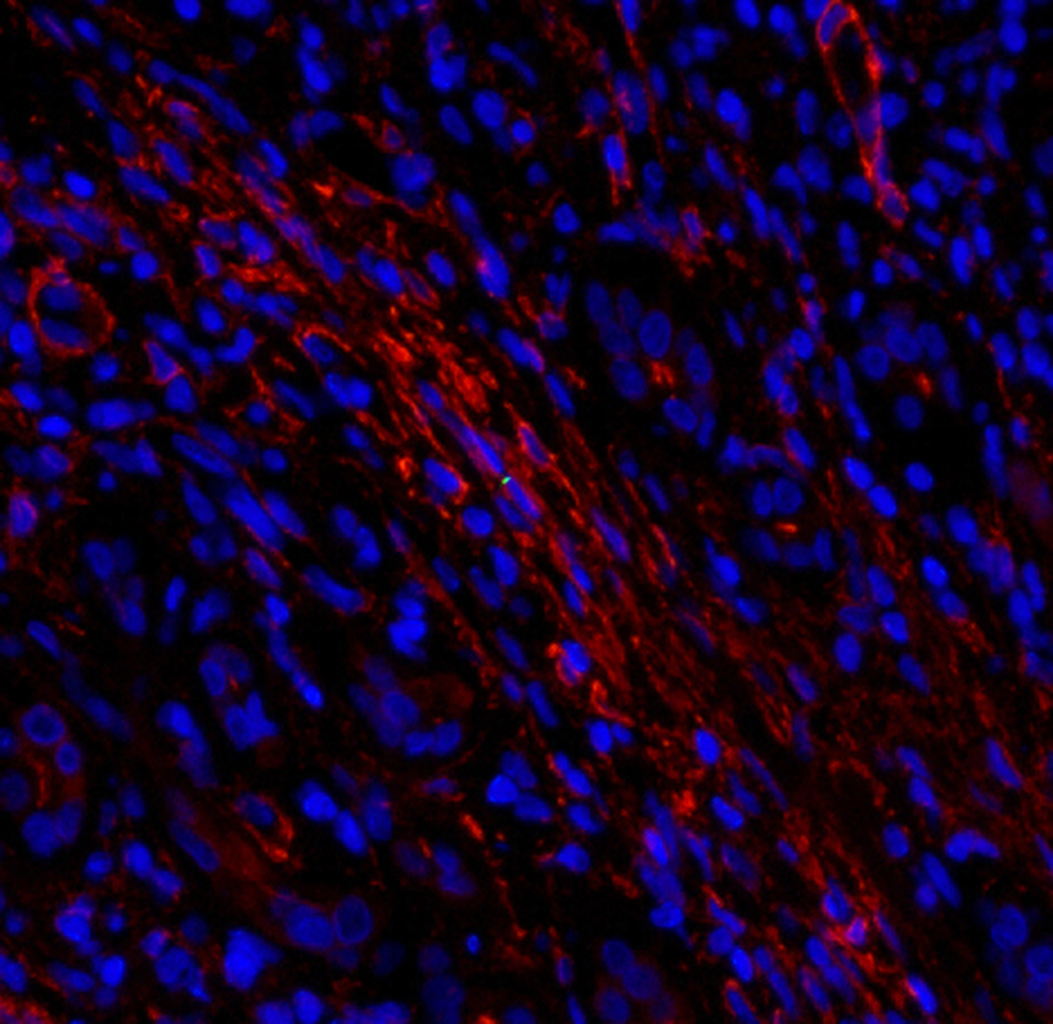

Supplement: Supplementary file 2 — Source Data Fig. 1 [file 44319_2024_92_MOESM2_ESM.zip › Figure 1E/Figure 1E liver fibrosis.tif]

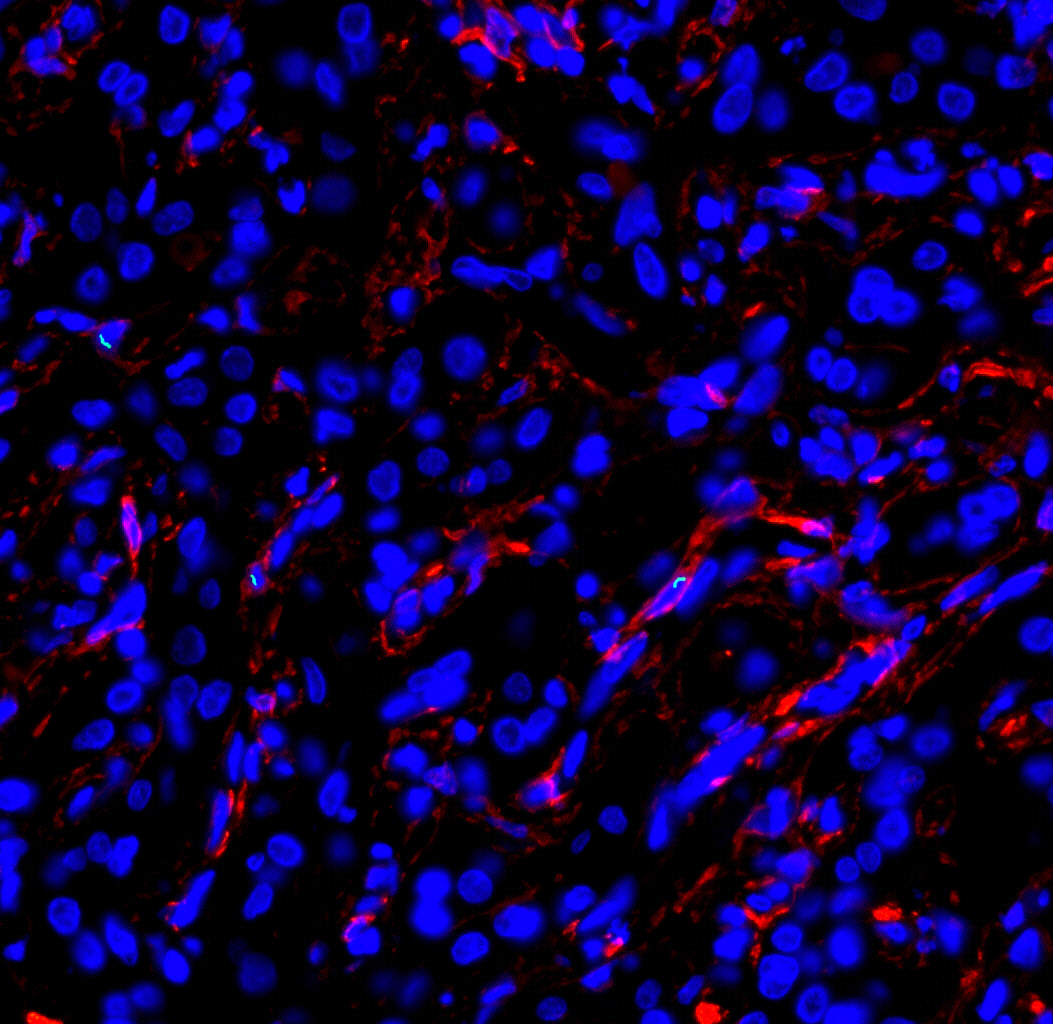

Supplement: Supplementary file 2 — Source Data Fig. 1 [file 44319_2024_92_MOESM2_ESM.zip › Figure 1E/Figure 1E Normal.tif]

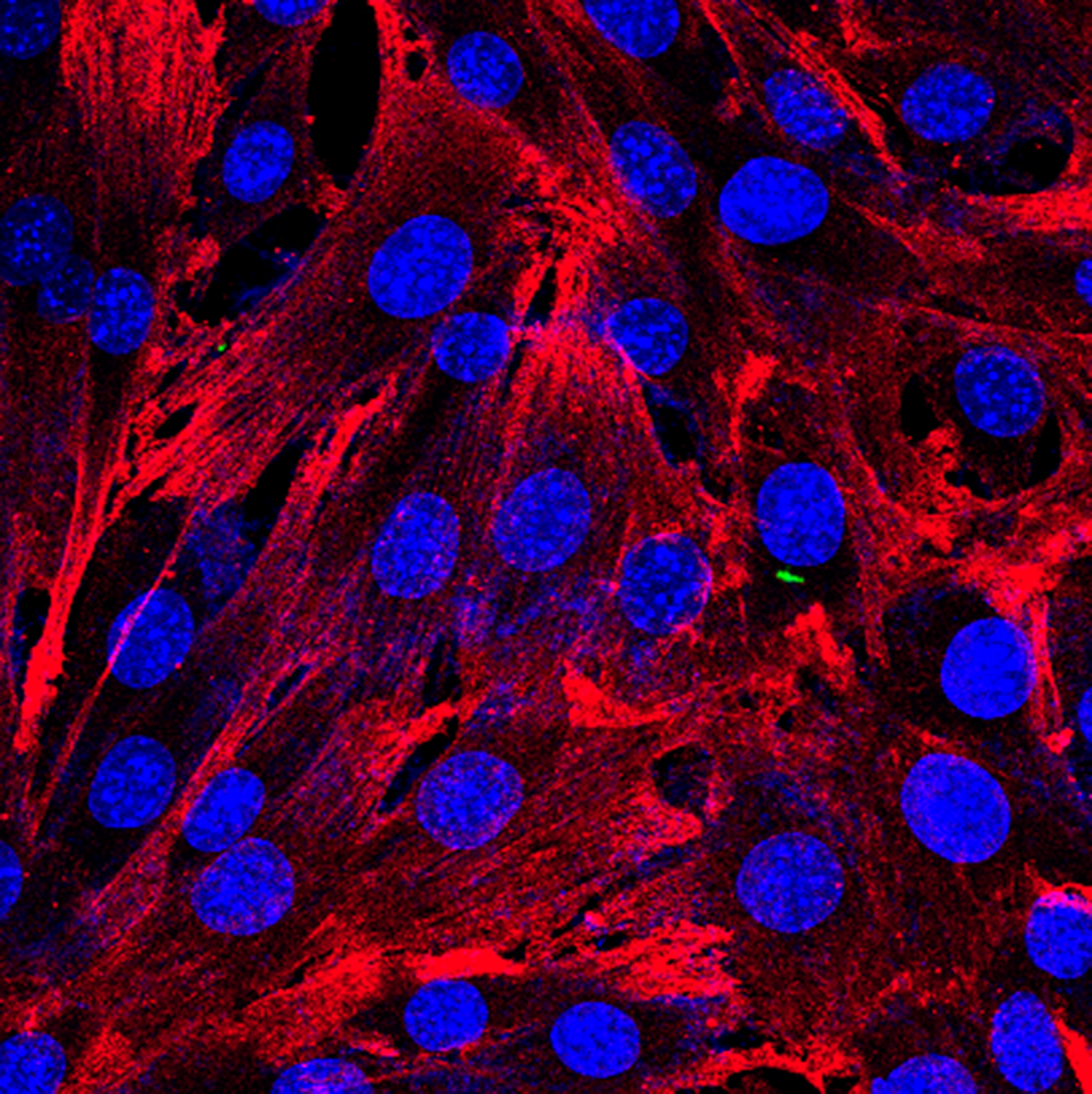

Supplement: Supplementary file 2 — Source Data Fig. 1 [file 44319_2024_92_MOESM2_ESM.zip › Figure 1G/Figure 1G CCl4.tif]

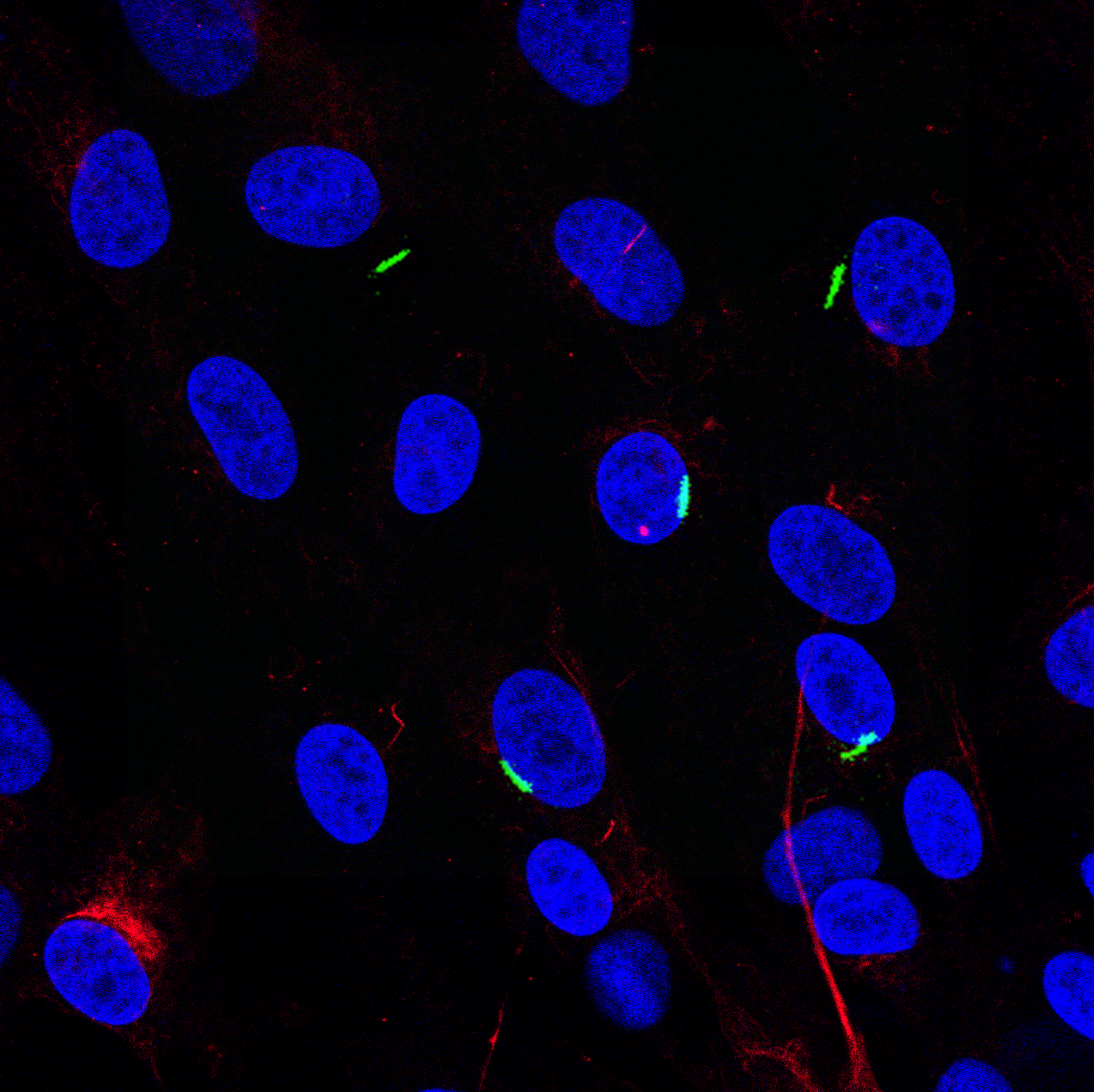

Supplement: Supplementary file 2 — Source Data Fig. 1 [file 44319_2024_92_MOESM2_ESM.zip › Figure 1G/Figure 1G Vehicle.tif]

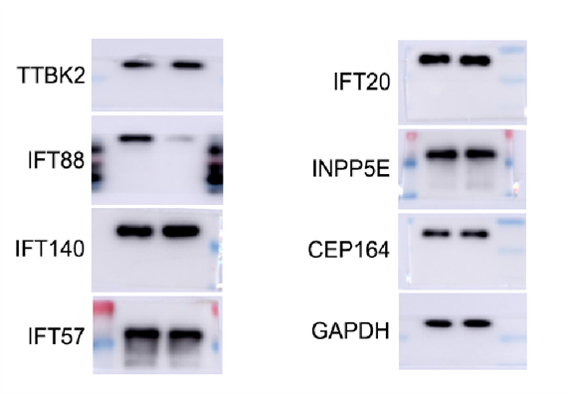

Supplement: Supplementary file 2 — Source Data Fig. 1 [file 44319_2024_92_MOESM2_ESM.zip › Figure 1I/Figure 1I.tif]

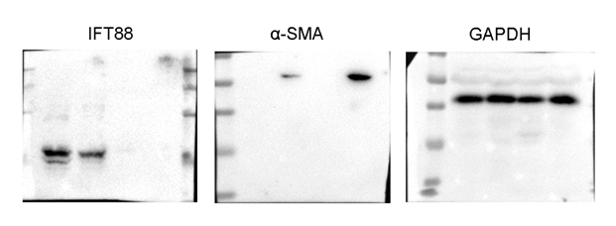

Supplement: Supplementary file 3 — Source Data Fig. 2 [file 44319_2024_92_MOESM3_ESM.zip › Figure 2A/Figure 2A.tif]

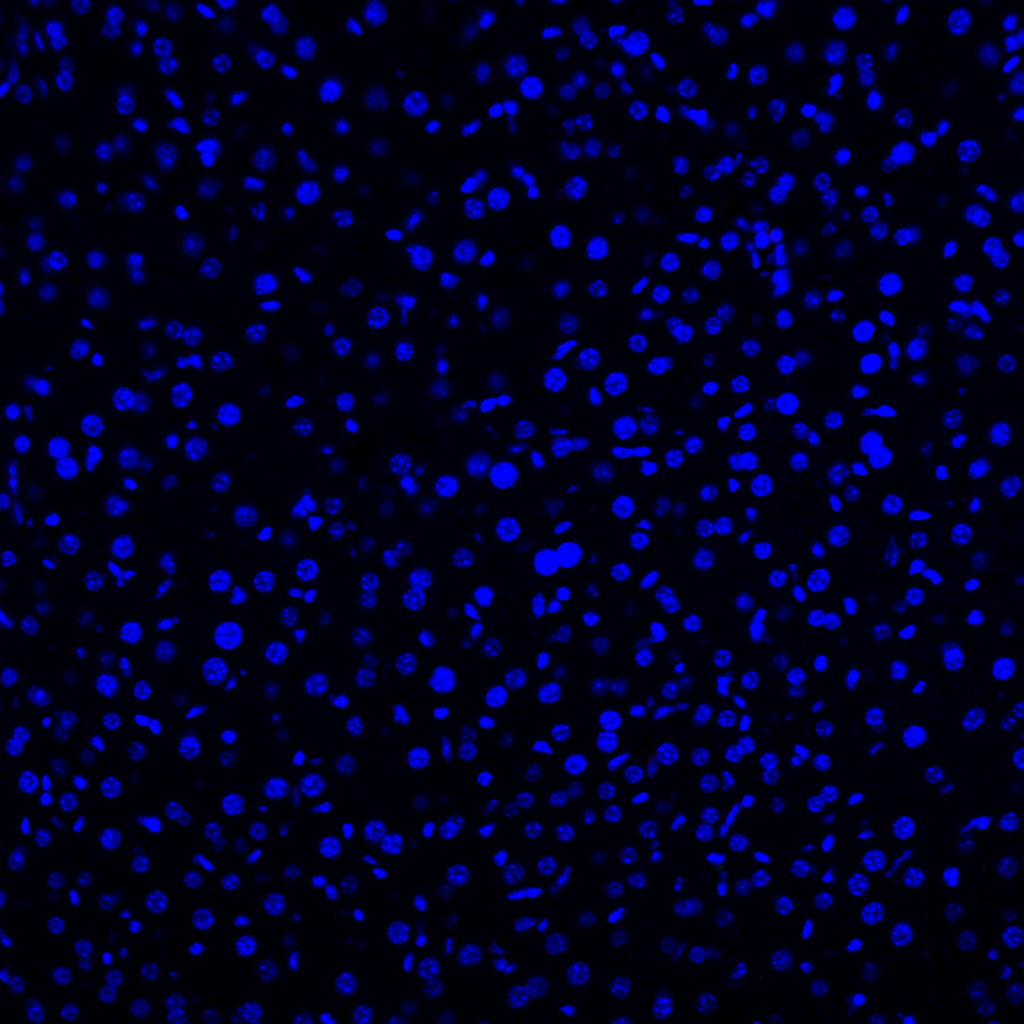

Supplement: Supplementary file 3 — Source Data Fig. 2 [file 44319_2024_92_MOESM3_ESM.zip › Figure 2E/Figure 2E 1.tif]

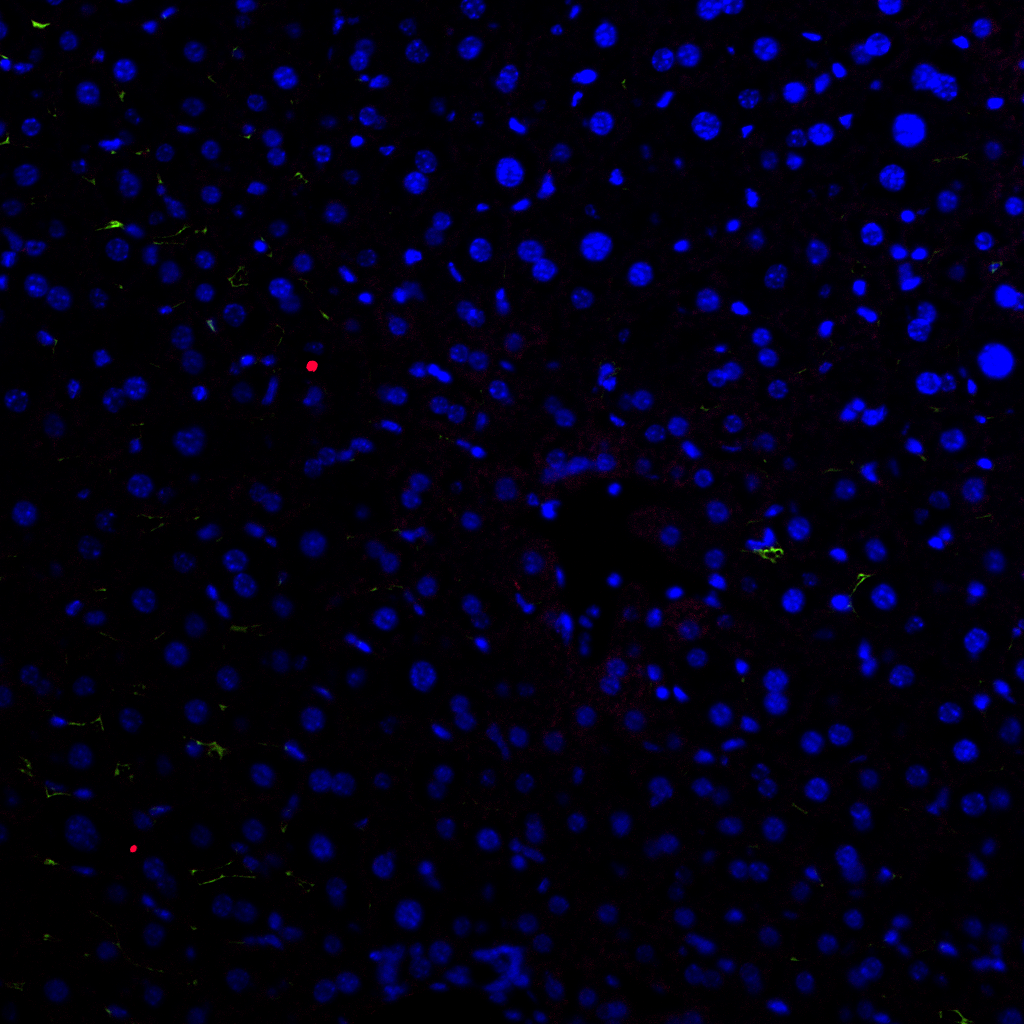

Supplement: Supplementary file 3 — Source Data Fig. 2 [file 44319_2024_92_MOESM3_ESM.zip › Figure 2E/Figure 2E 2.tif]

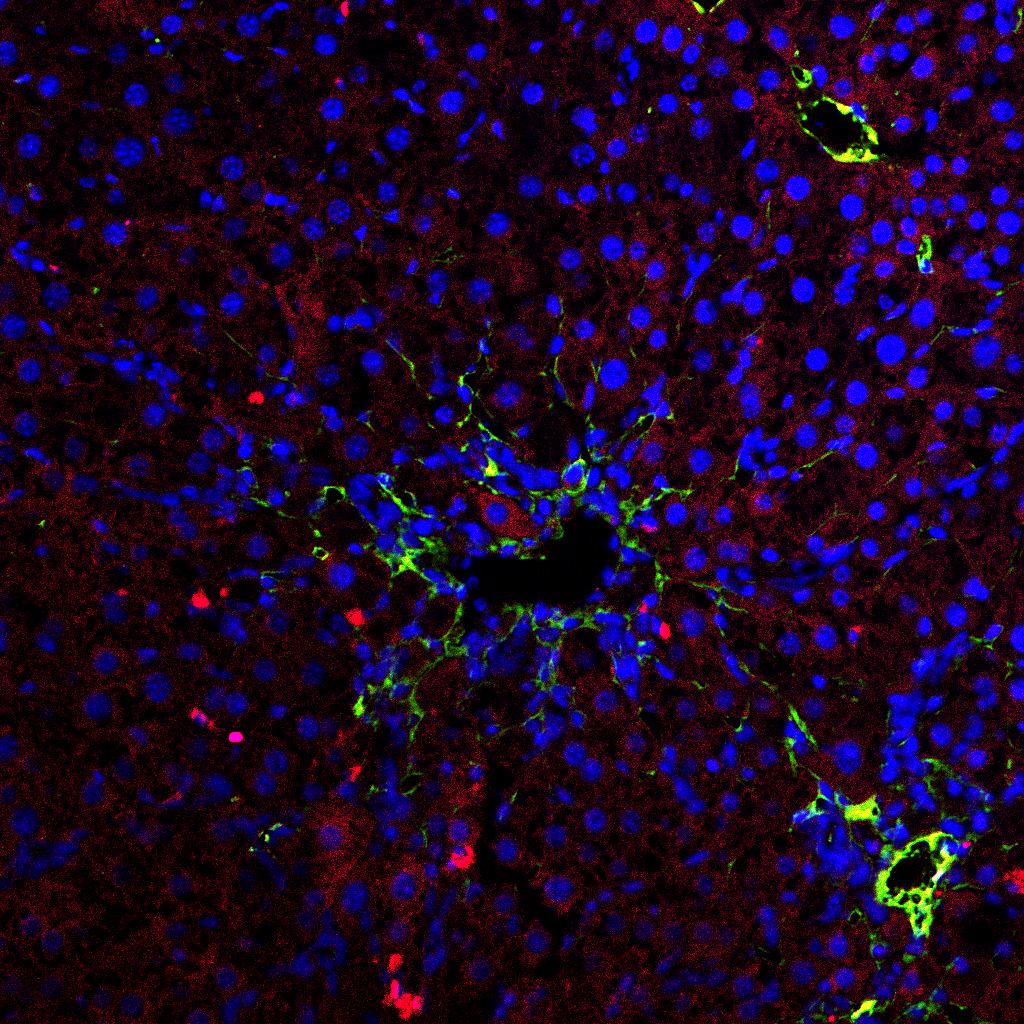

Supplement: Supplementary file 3 — Source Data Fig. 2 [file 44319_2024_92_MOESM3_ESM.zip › Figure 2E/Figure 2E 3.tif]

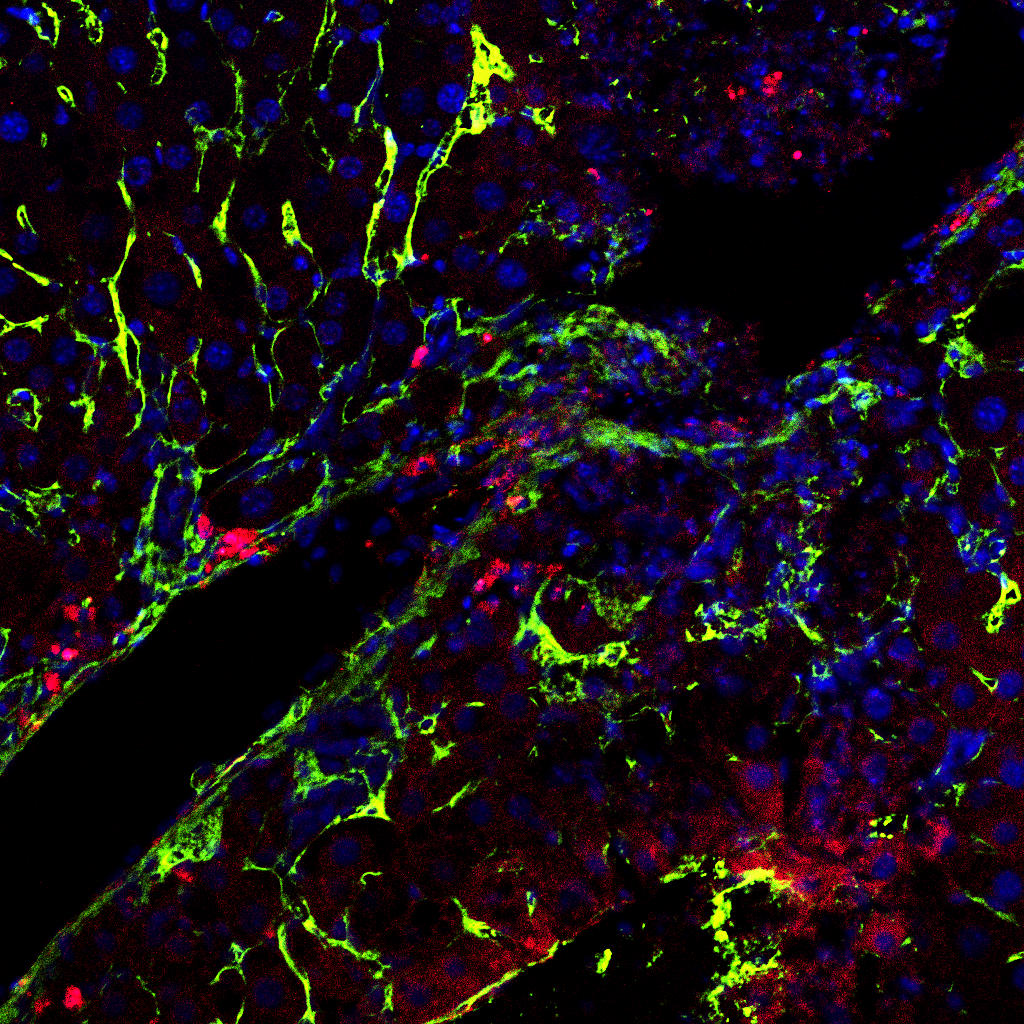

Supplement: Supplementary file 3 — Source Data Fig. 2 [file 44319_2024_92_MOESM3_ESM.zip › Figure 2E/Figure 2E 4.tif]

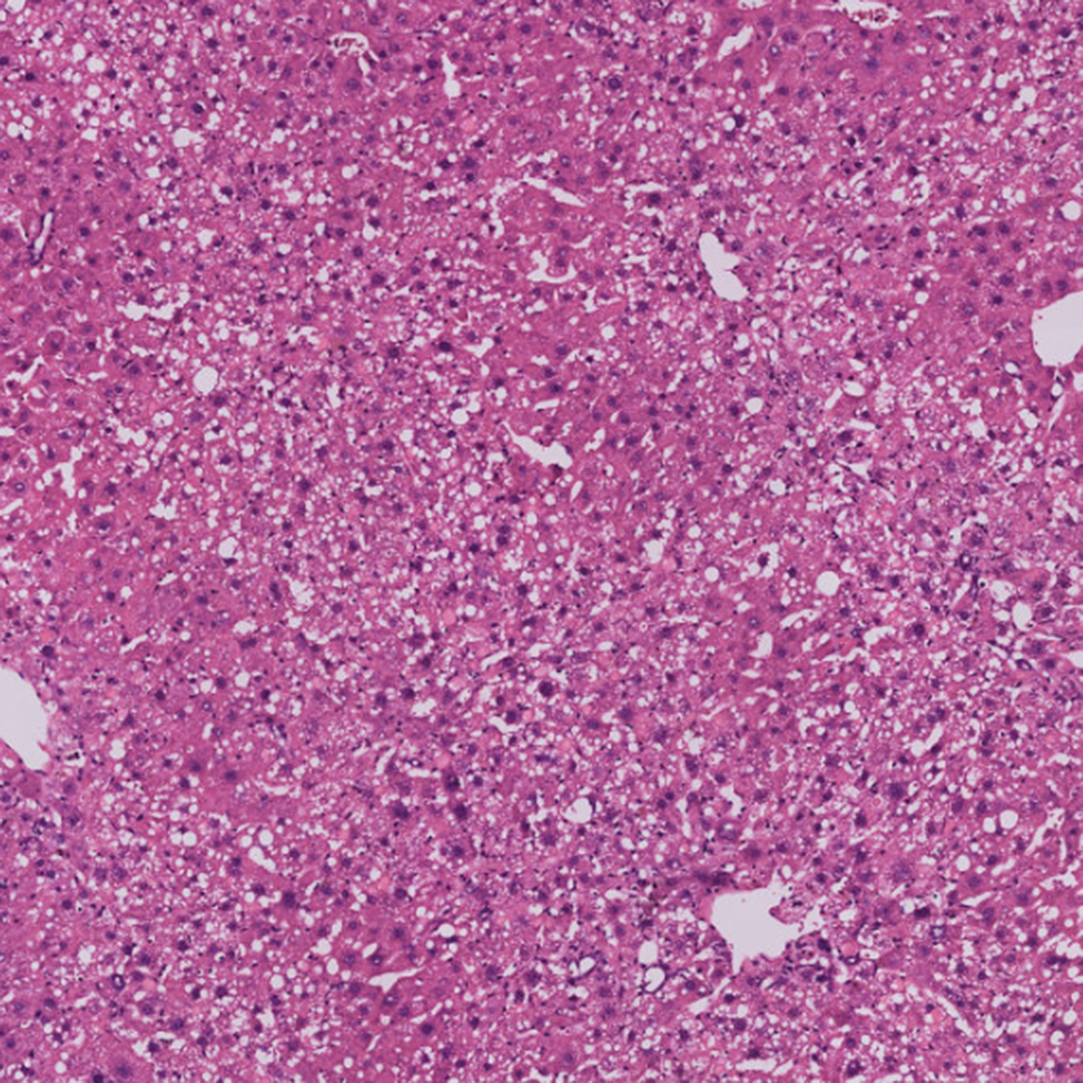

Supplement: Supplementary file 3 — Source Data Fig. 2 [file 44319_2024_92_MOESM3_ESM.zip › Figure 2H/HE/HE 1.tif]

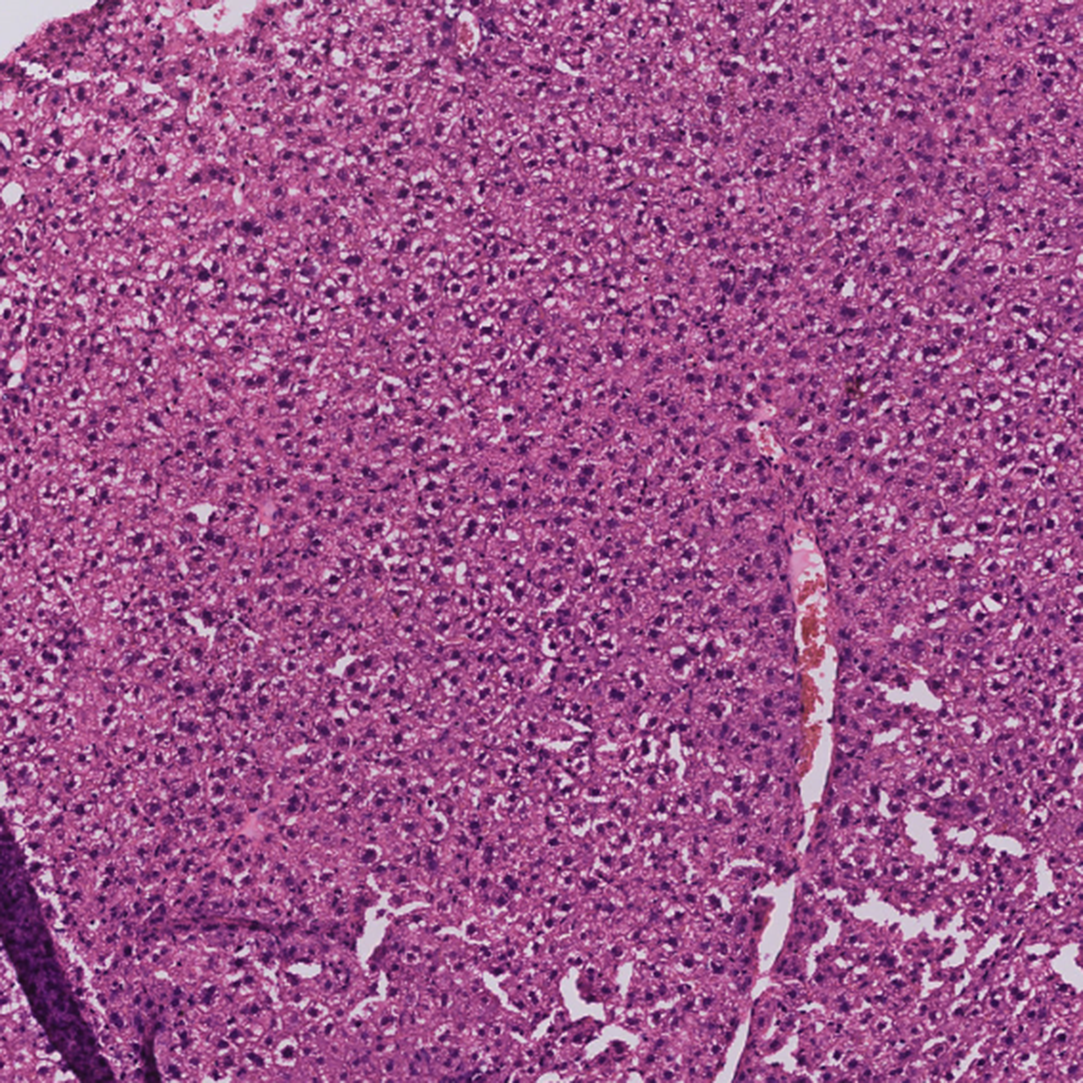

Supplement: Supplementary file 3 — Source Data Fig. 2 [file 44319_2024_92_MOESM3_ESM.zip › Figure 2H/HE/HE 2.tif]

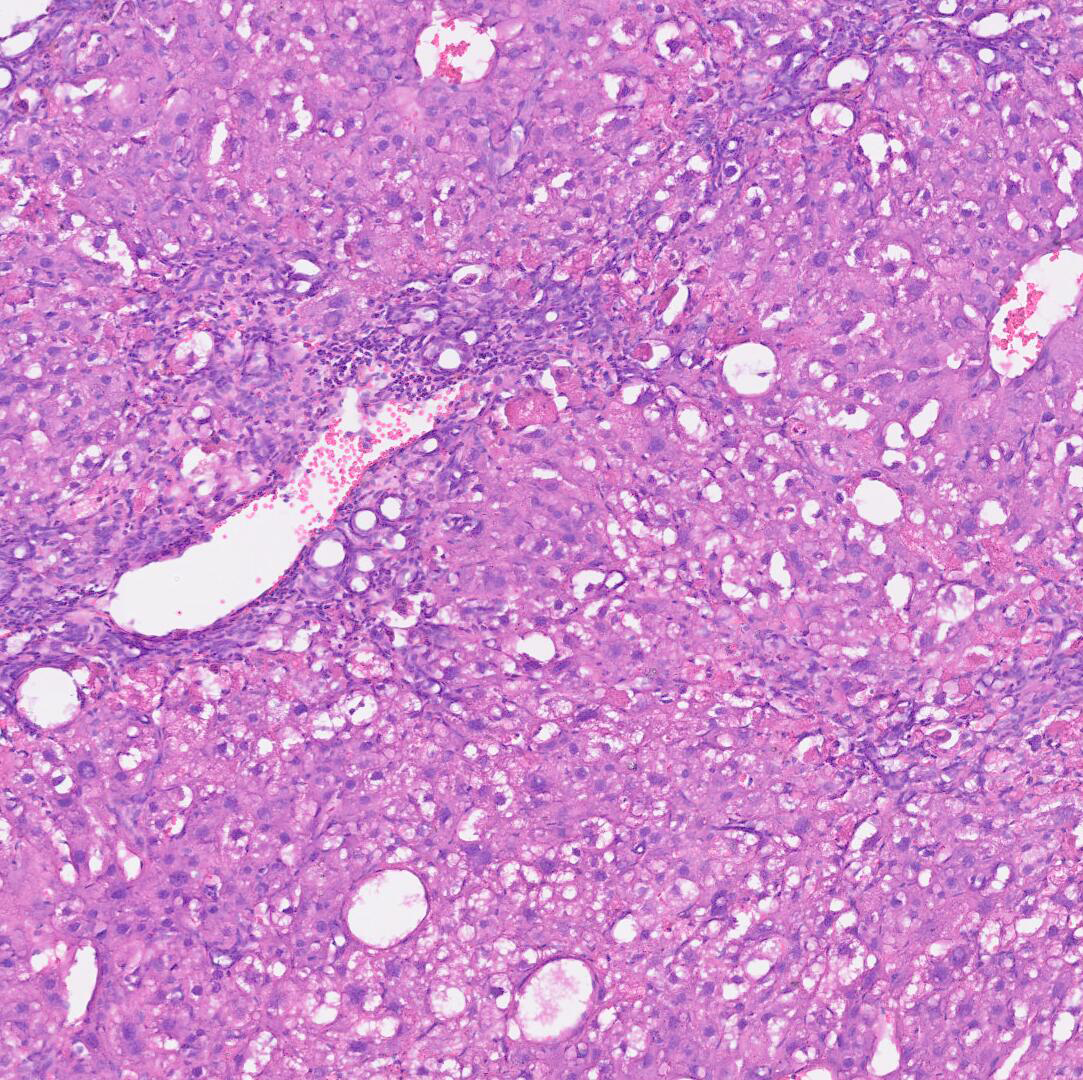

Supplement: Supplementary file 3 — Source Data Fig. 2 [file 44319_2024_92_MOESM3_ESM.zip › Figure 2H/HE/HE 3.tif]

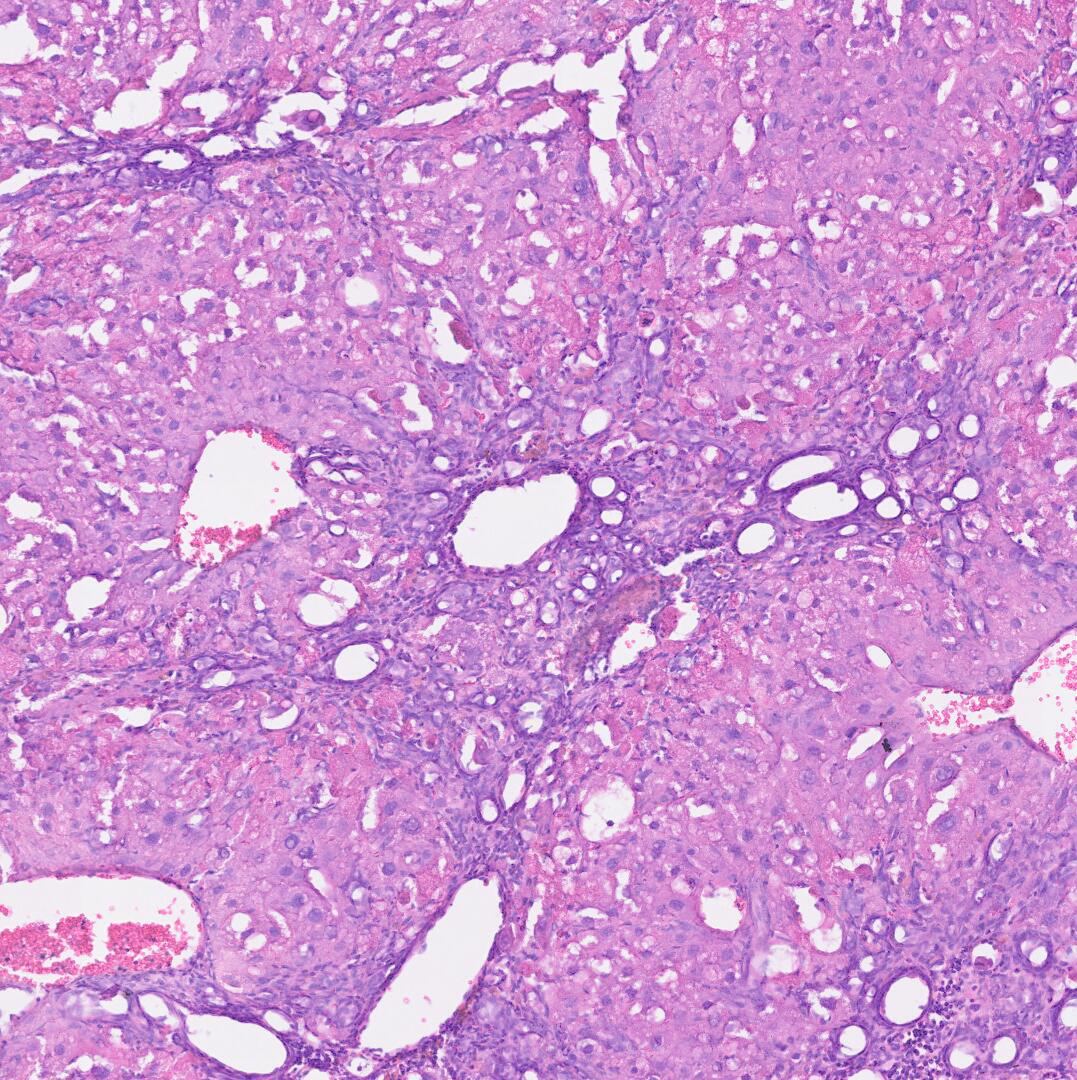

Supplement: Supplementary file 3 — Source Data Fig. 2 [file 44319_2024_92_MOESM3_ESM.zip › Figure 2H/HE/HE 4.tif]

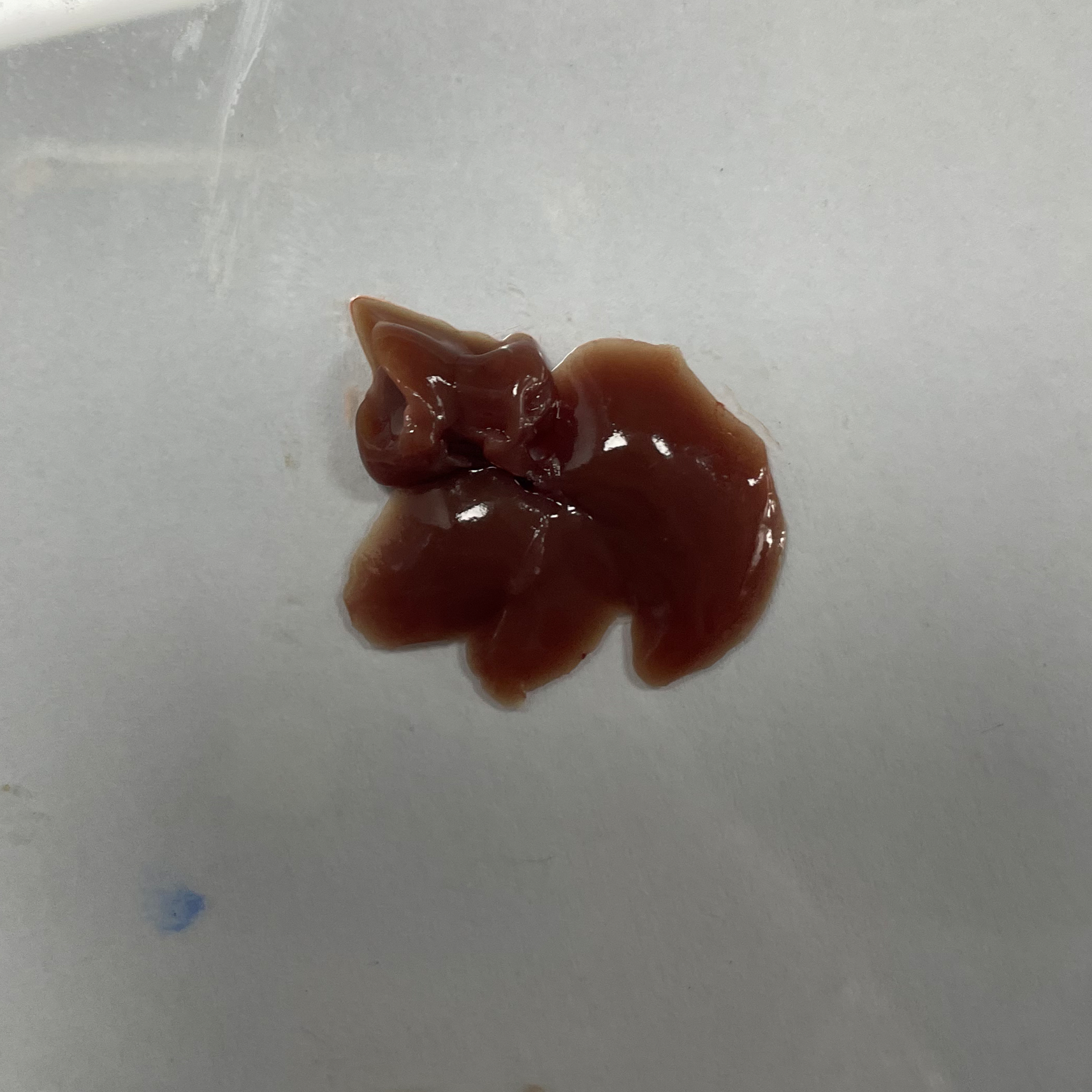

Supplement: Supplementary file 3 — Source Data Fig. 2 [file 44319_2024_92_MOESM3_ESM.zip › Figure 2H/liver/1.tif]

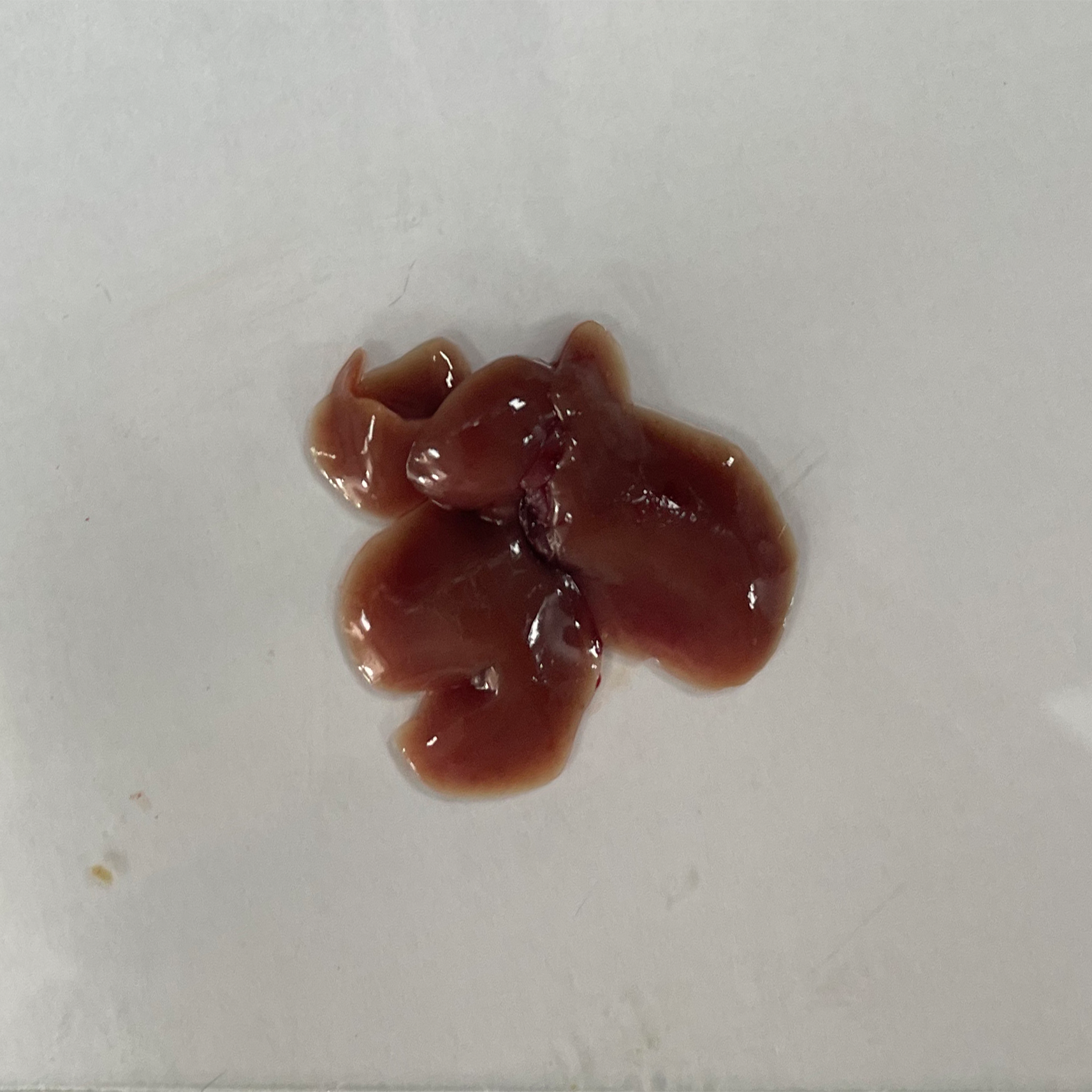

Supplement: Supplementary file 3 — Source Data Fig. 2 [file 44319_2024_92_MOESM3_ESM.zip › Figure 2H/liver/2.tif]

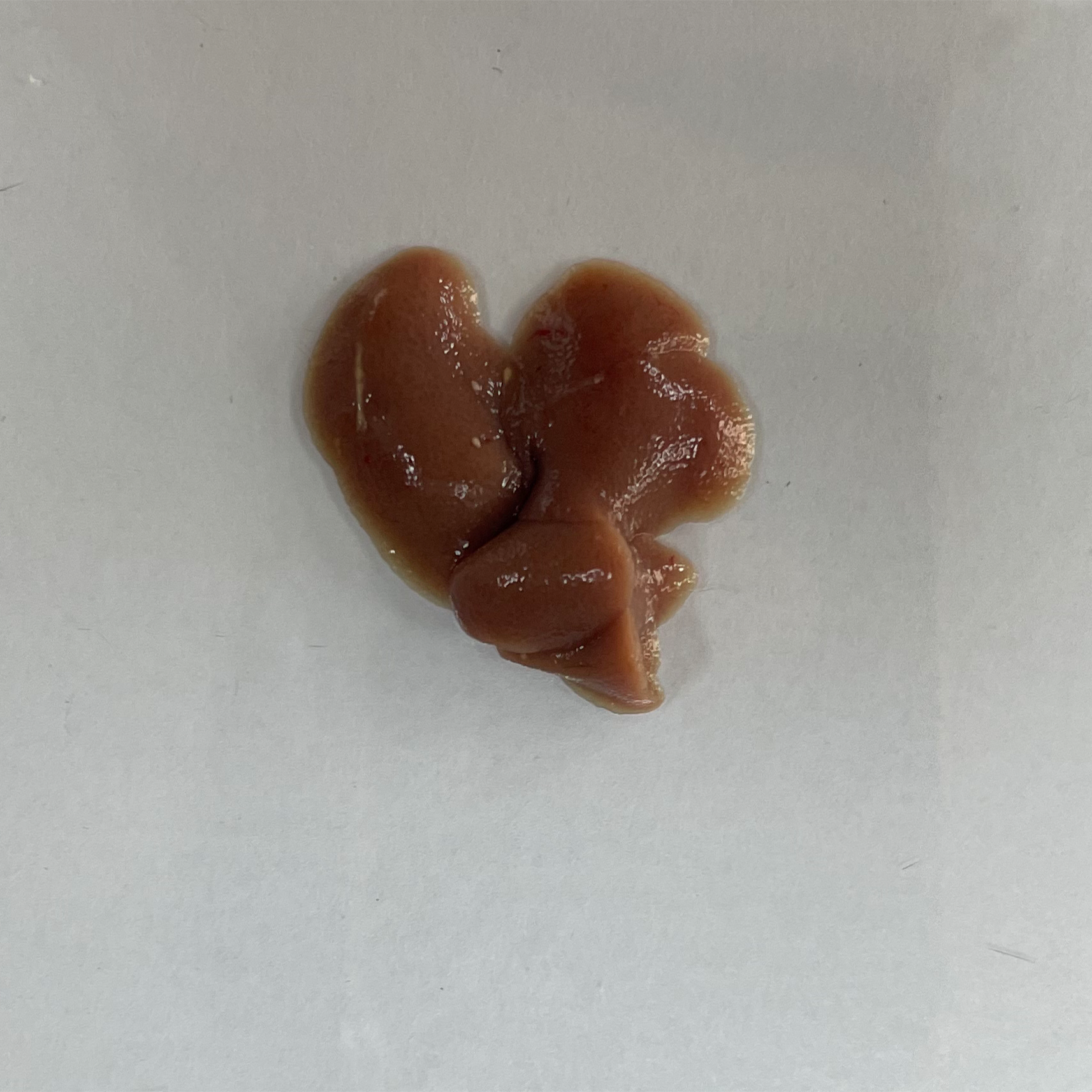

Supplement: Supplementary file 3 — Source Data Fig. 2 [file 44319_2024_92_MOESM3_ESM.zip › Figure 2H/liver/3.tif]

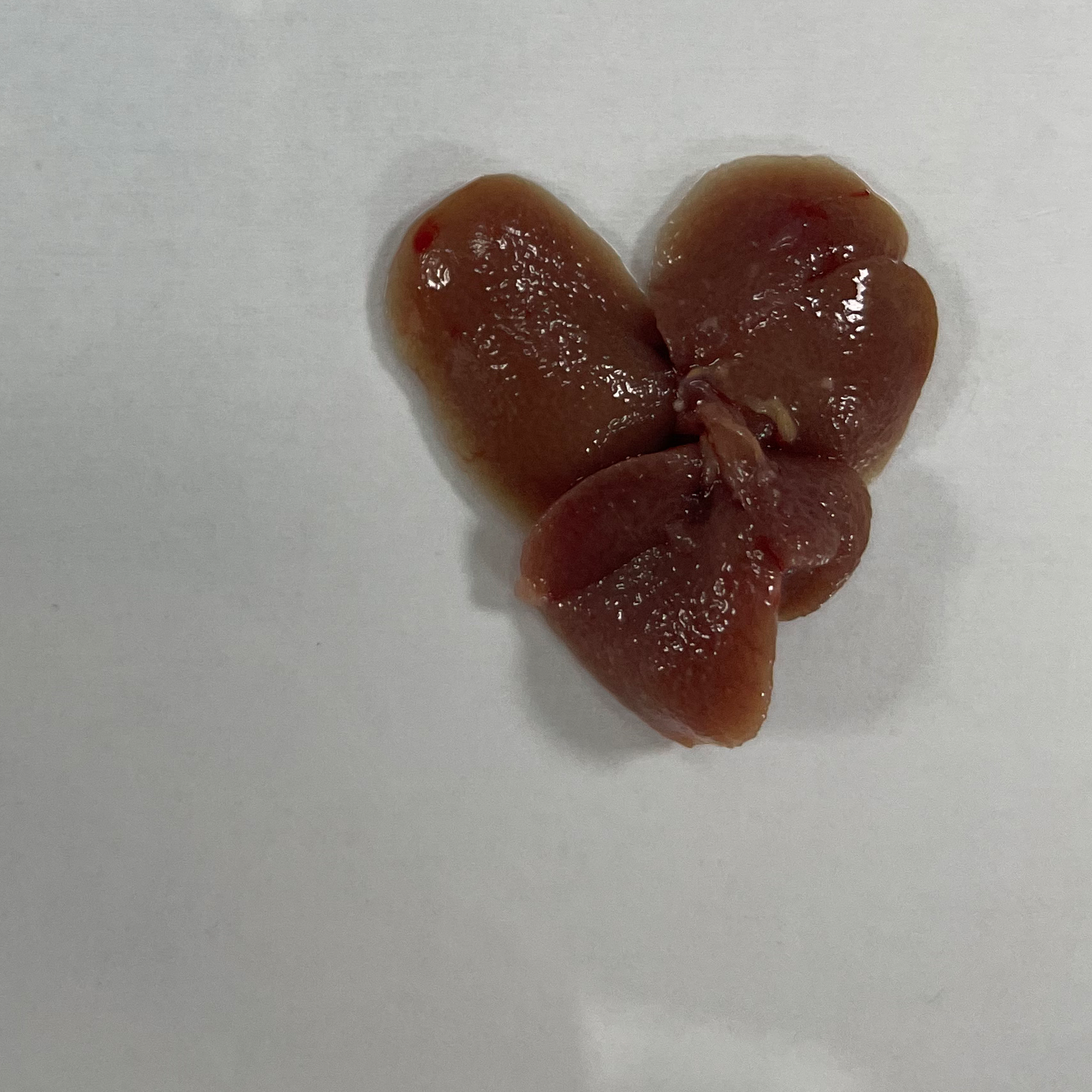

Supplement: Supplementary file 3 — Source Data Fig. 2 [file 44319_2024_92_MOESM3_ESM.zip › Figure 2H/liver/4.tif]

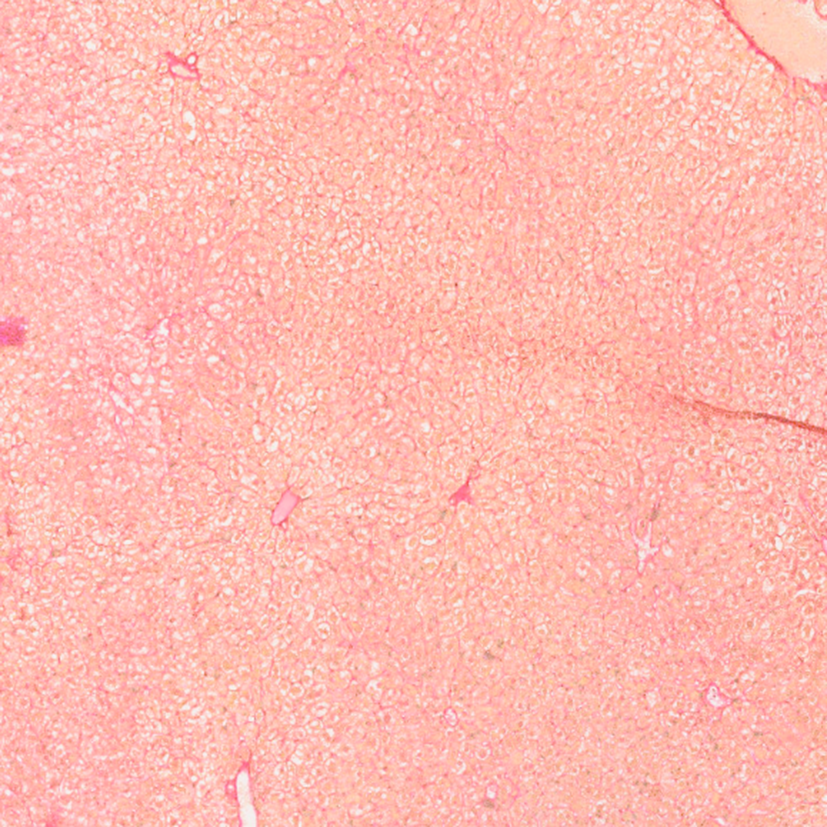

Supplement: Supplementary file 3 — Source Data Fig. 2 [file 44319_2024_92_MOESM3_ESM.zip › Figure 2H/Sirius red/Sirius red 1 .tif]

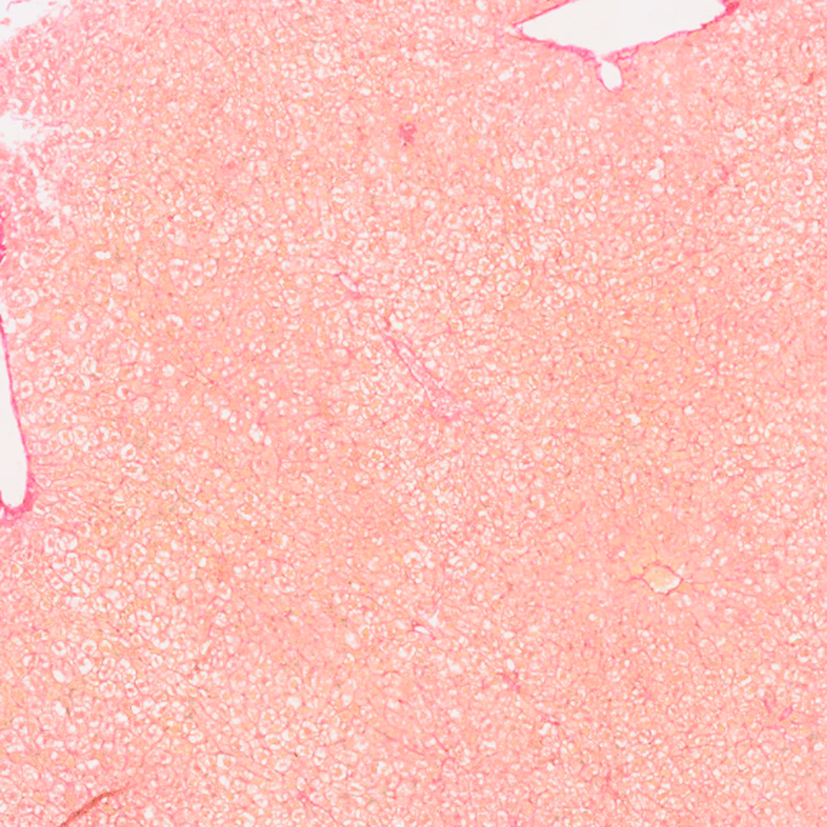

Supplement: Supplementary file 3 — Source Data Fig. 2 [file 44319_2024_92_MOESM3_ESM.zip › Figure 2H/Sirius red/Sirius red 2 .tif]

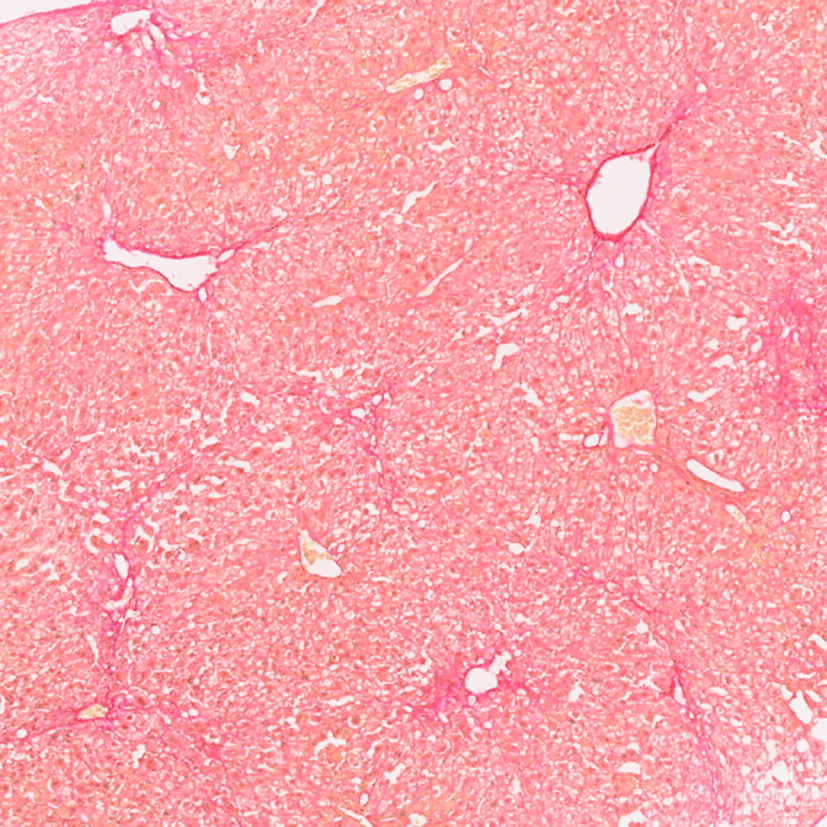

Supplement: Supplementary file 3 — Source Data Fig. 2 [file 44319_2024_92_MOESM3_ESM.zip › Figure 2H/Sirius red/Sirius red 3 .tif]

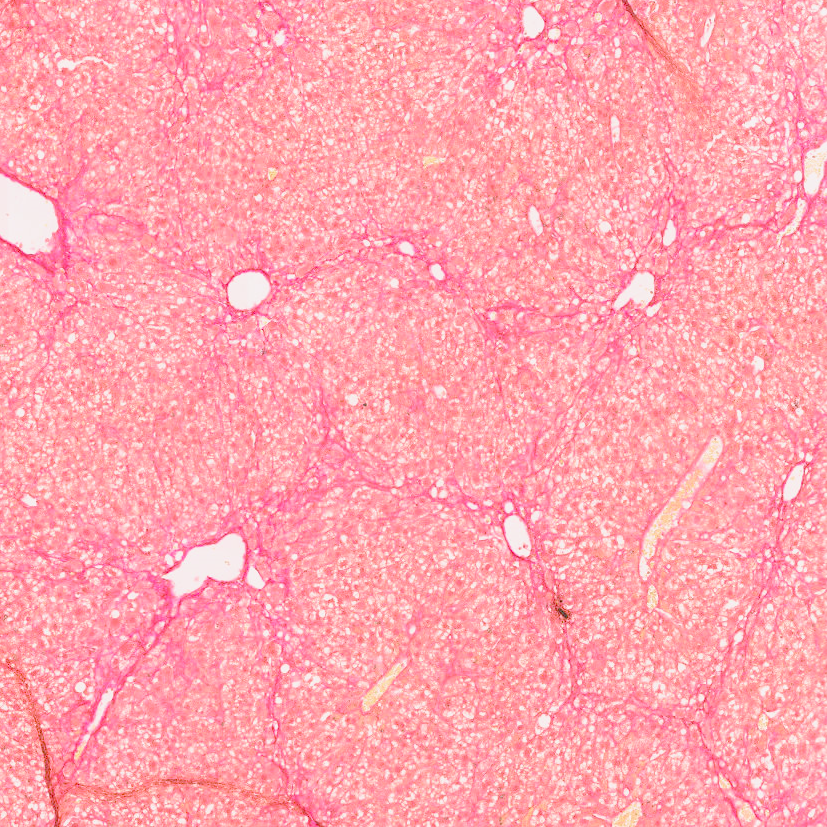

Supplement: Supplementary file 3 — Source Data Fig. 2 [file 44319_2024_92_MOESM3_ESM.zip › Figure 2H/Sirius red/Sirius red 4 .tif]

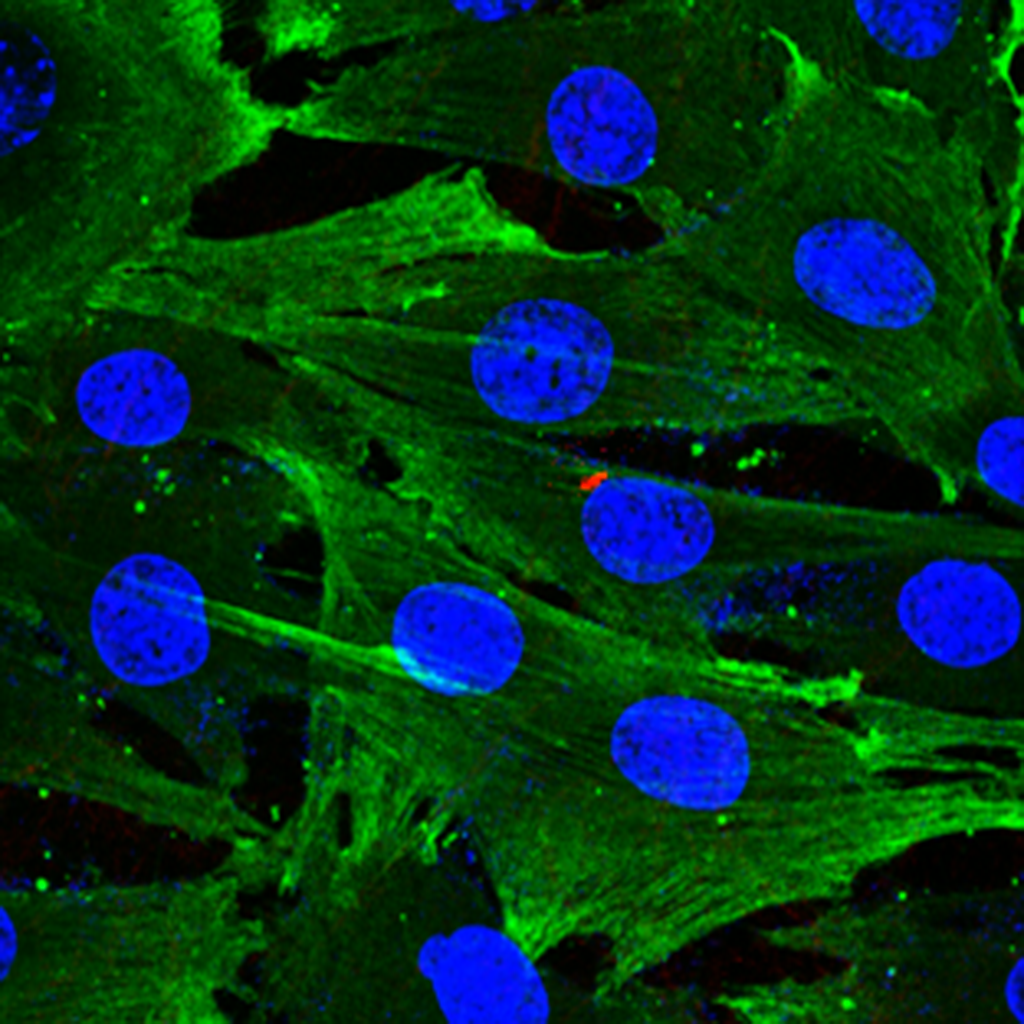

Supplement: Supplementary file 4 — Source Data Fig. 3 [file 44319_2024_92_MOESM4_ESM.zip › Figure 3A/TGFβ.tif]

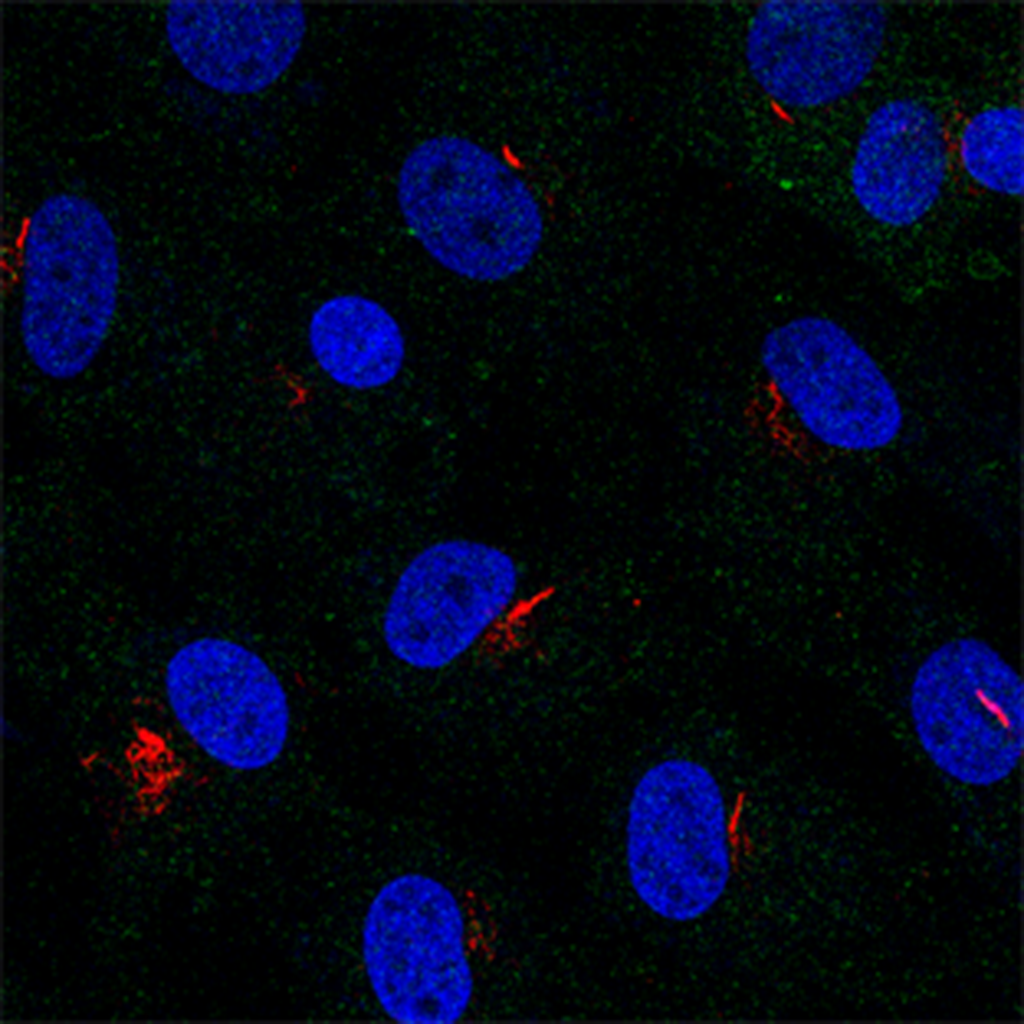

Supplement: Supplementary file 4 — Source Data Fig. 3 [file 44319_2024_92_MOESM4_ESM.zip › Figure 3A/Vehicle.tif]

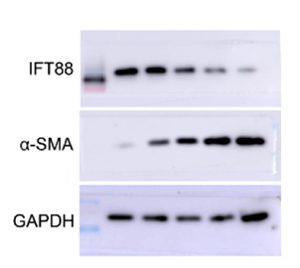

Supplement: Supplementary file 4 — Source Data Fig. 3 [file 44319_2024_92_MOESM4_ESM.zip › Figure 3D/Figure 3D.tif]

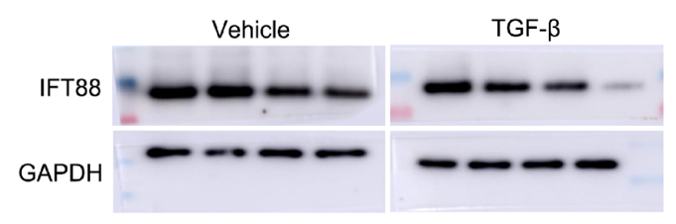

Supplement: Supplementary file 4 — Source Data Fig. 3 [file 44319_2024_92_MOESM4_ESM.zip › Figure 3G/Figure 3G.tif]

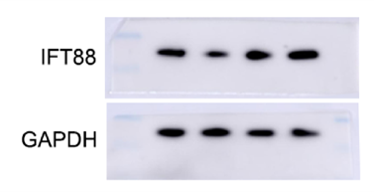

Supplement: Supplementary file 4 — Source Data Fig. 3 [file 44319_2024_92_MOESM4_ESM.zip › Figure 3I/Figure 3I.tif]

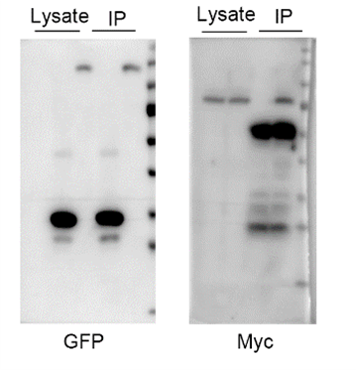

Supplement: Supplementary file 5 — Source Data Fig. 4 [file 44319_2024_92_MOESM5_ESM.zip › Figure 4A/Figure 4A.tif]

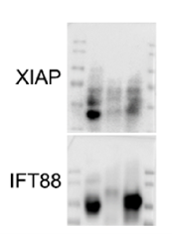

Supplement: Supplementary file 5 — Source Data Fig. 4 [file 44319_2024_92_MOESM5_ESM.zip › Figure 4B/Figure 4B.tif]

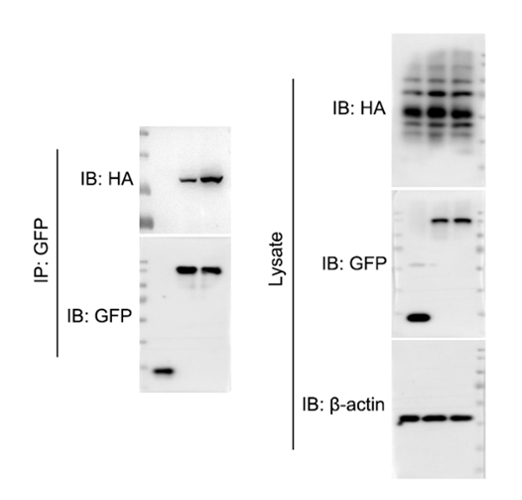

Supplement: Supplementary file 5 — Source Data Fig. 4 [file 44319_2024_92_MOESM5_ESM.zip › Figure 4C/Figure 4C.tif]

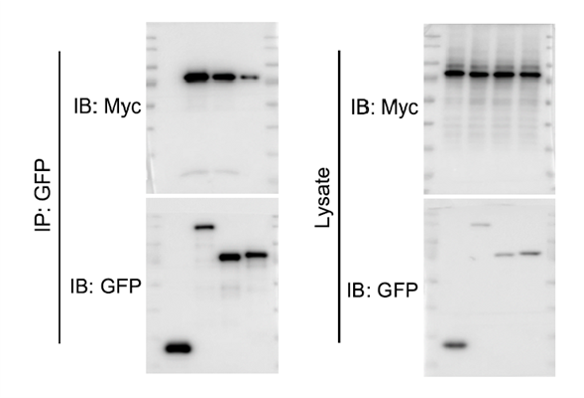

Supplement: Supplementary file 5 — Source Data Fig. 4 [file 44319_2024_92_MOESM5_ESM.zip › Figure 4E/Figure 4E.tif]

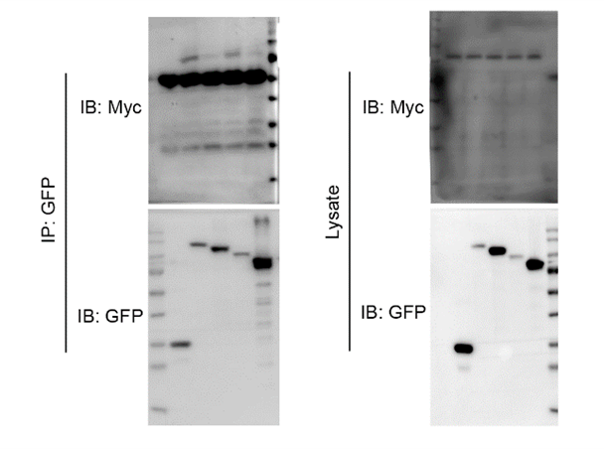

Supplement: Supplementary file 5 — Source Data Fig. 4 [file 44319_2024_92_MOESM5_ESM.zip › Figure 4F/Figure 4F.tif]

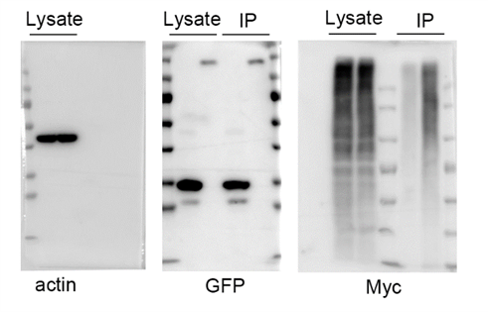

Supplement: Supplementary file 6 — Source Data Fig. 5 [file 44319_2024_92_MOESM6_ESM.zip › Figure 5A/Figure 5A.tif]

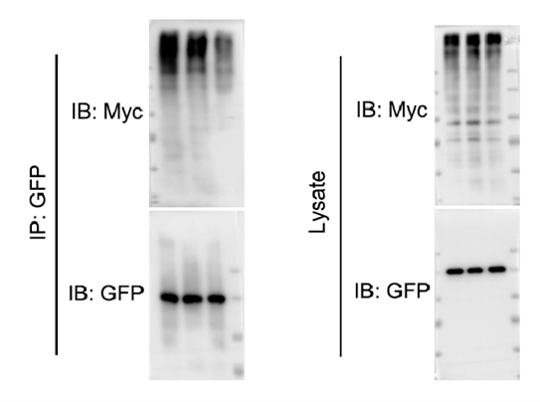

Supplement: Supplementary file 6 — Source Data Fig. 5 [file 44319_2024_92_MOESM6_ESM.zip › Figure 5B/Figure 5B.tif]

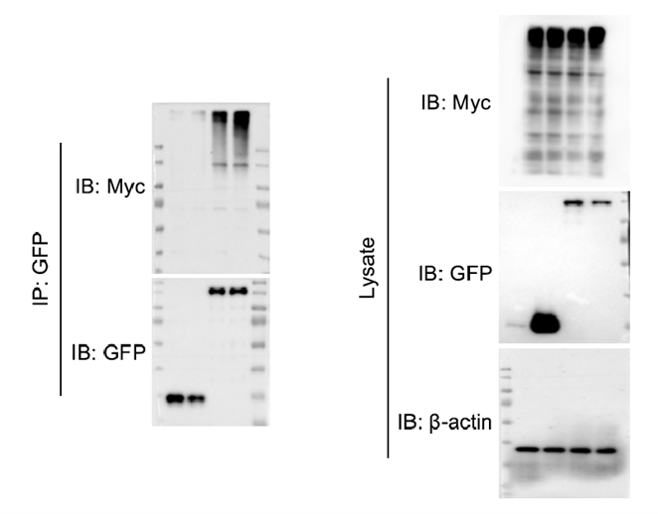

Supplement: Supplementary file 6 — Source Data Fig. 5 [file 44319_2024_92_MOESM6_ESM.zip › Figure 5C/Figure 5C.tif]

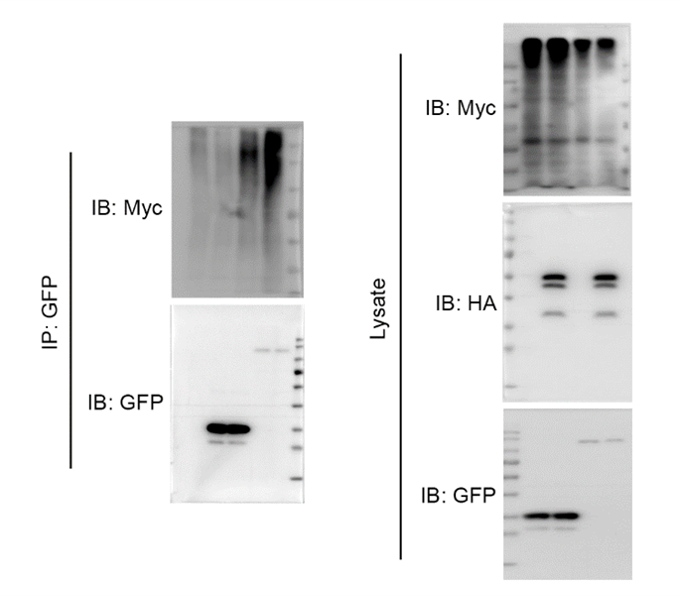

Supplement: Supplementary file 6 — Source Data Fig. 5 [file 44319_2024_92_MOESM6_ESM.zip › Figure 5D/Figure 5D.tif]

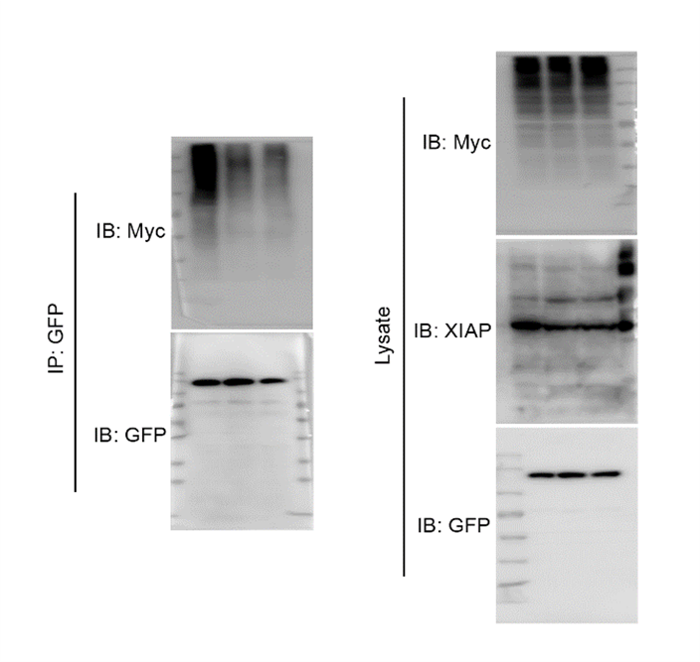

Supplement: Supplementary file 6 — Source Data Fig. 5 [file 44319_2024_92_MOESM6_ESM.zip › Figure 5E/Figure 5E.tif]

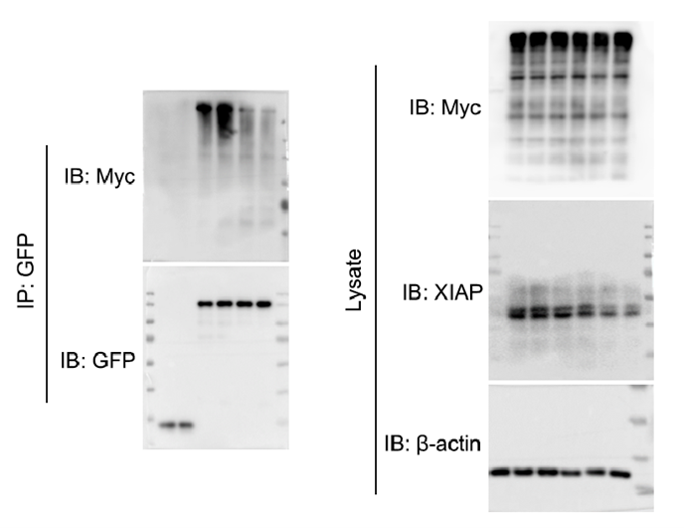

Supplement: Supplementary file 6 — Source Data Fig. 5 [file 44319_2024_92_MOESM6_ESM.zip › Figure 5F/Figure 5F.tif]

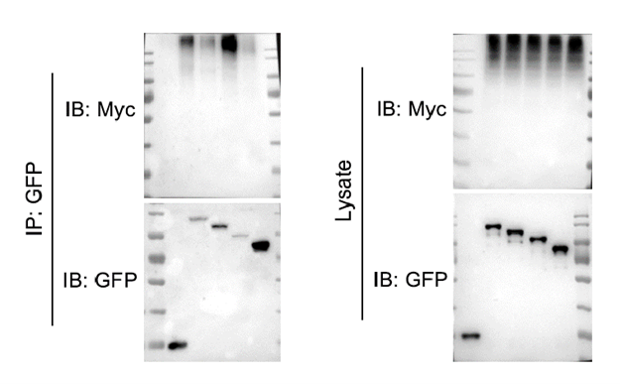

Supplement: Supplementary file 6 — Source Data Fig. 5 [file 44319_2024_92_MOESM6_ESM.zip › Figure 5G/Figure 5G.tif]

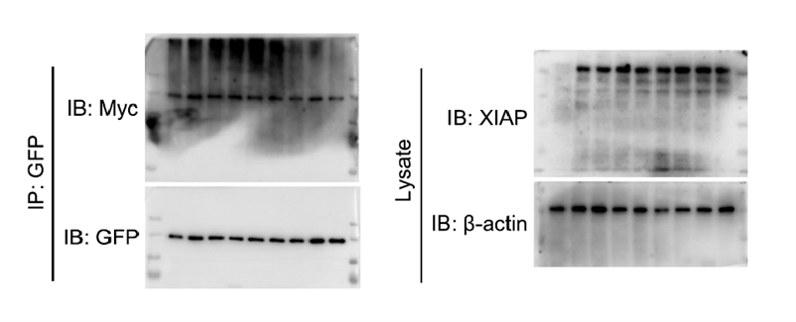

Supplement: Supplementary file 6 — Source Data Fig. 5 [file 44319_2024_92_MOESM6_ESM.zip › Figure 5I/Figure 5I.tif]

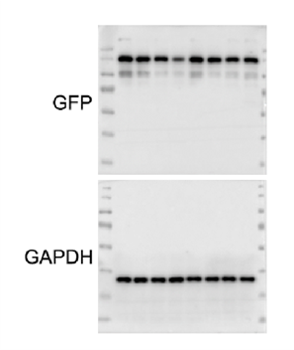

Supplement: Supplementary file 7 — Source Data Fig. 6 [file 44319_2024_92_MOESM7_ESM.zip › Figure 6A/Figure 6A.tif]

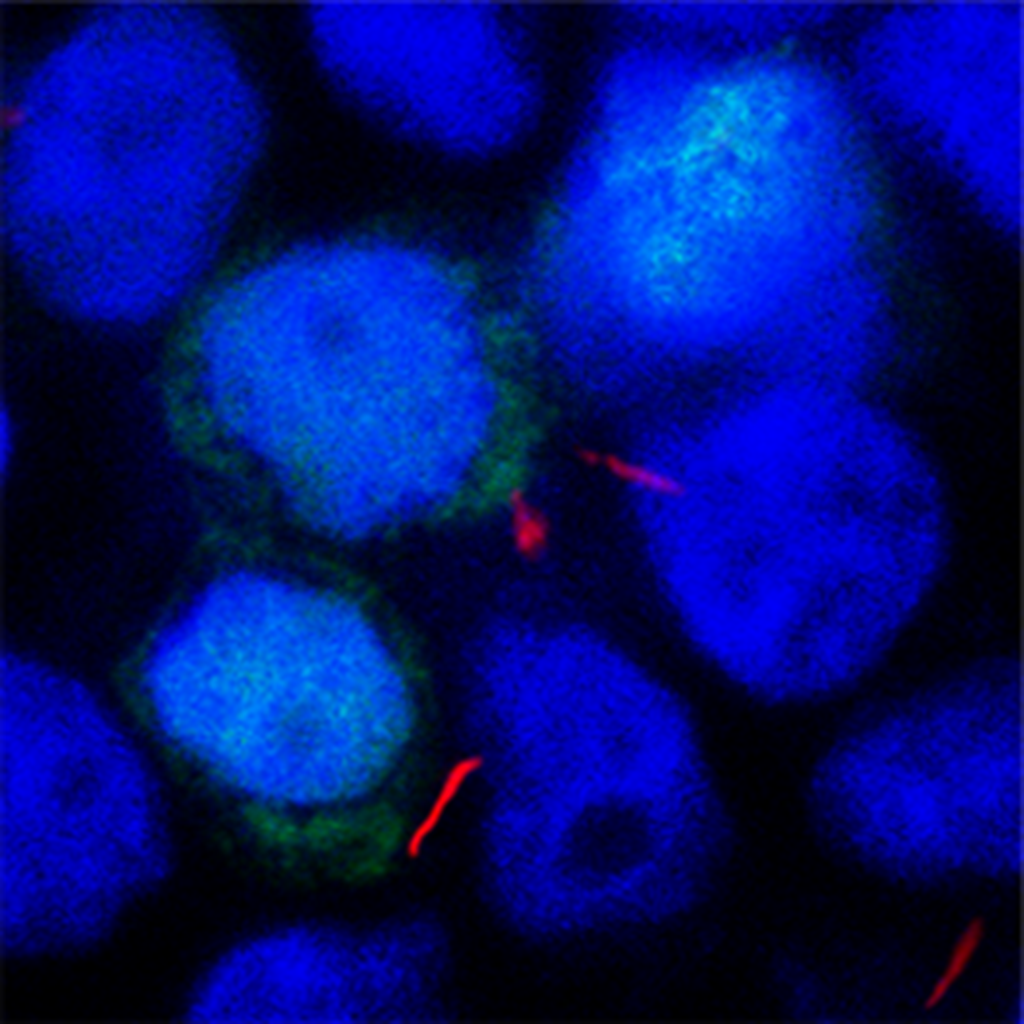

Supplement: Supplementary file 7 — Source Data Fig. 6 [file 44319_2024_92_MOESM7_ESM.zip › Figure 6C/1.tif]

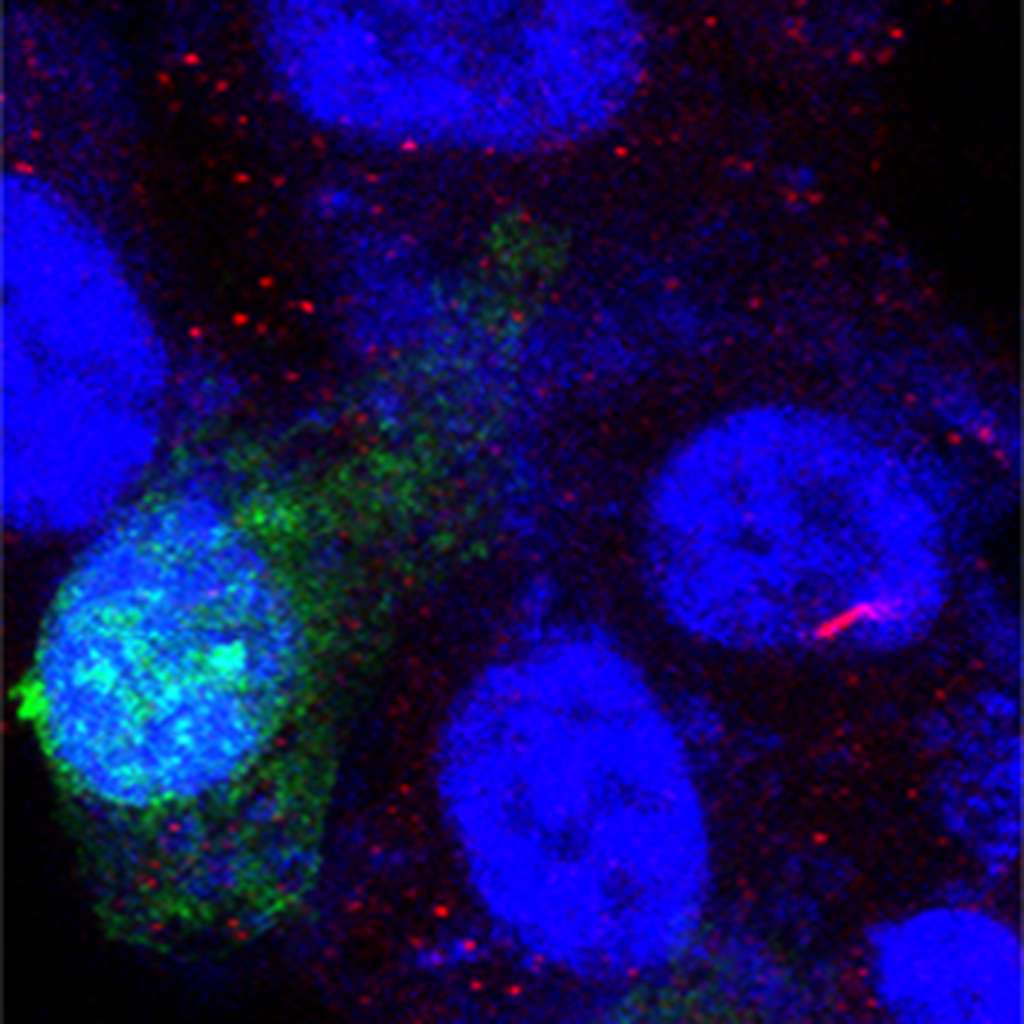

Supplement: Supplementary file 7 — Source Data Fig. 6 [file 44319_2024_92_MOESM7_ESM.zip › Figure 6C/2.tif]

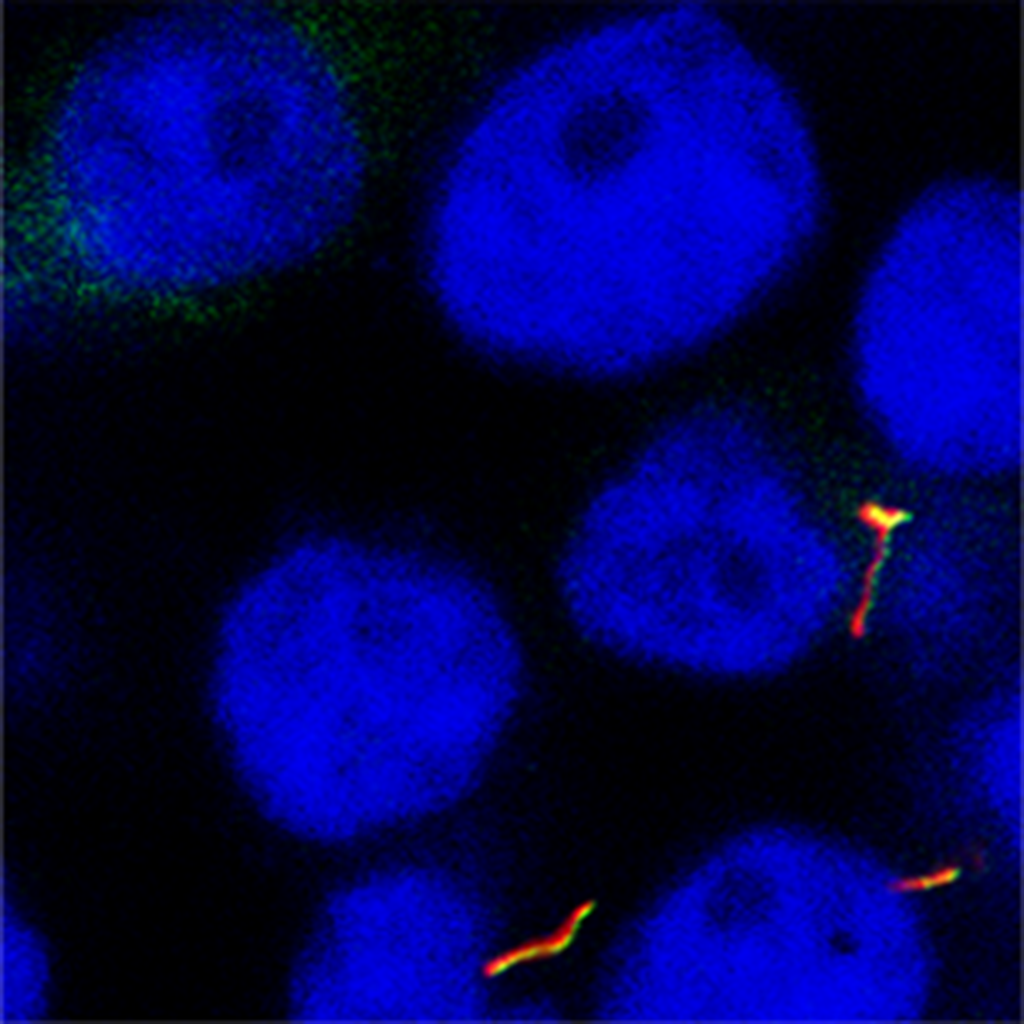

Supplement: Supplementary file 7 — Source Data Fig. 6 [file 44319_2024_92_MOESM7_ESM.zip › Figure 6C/3.tif]

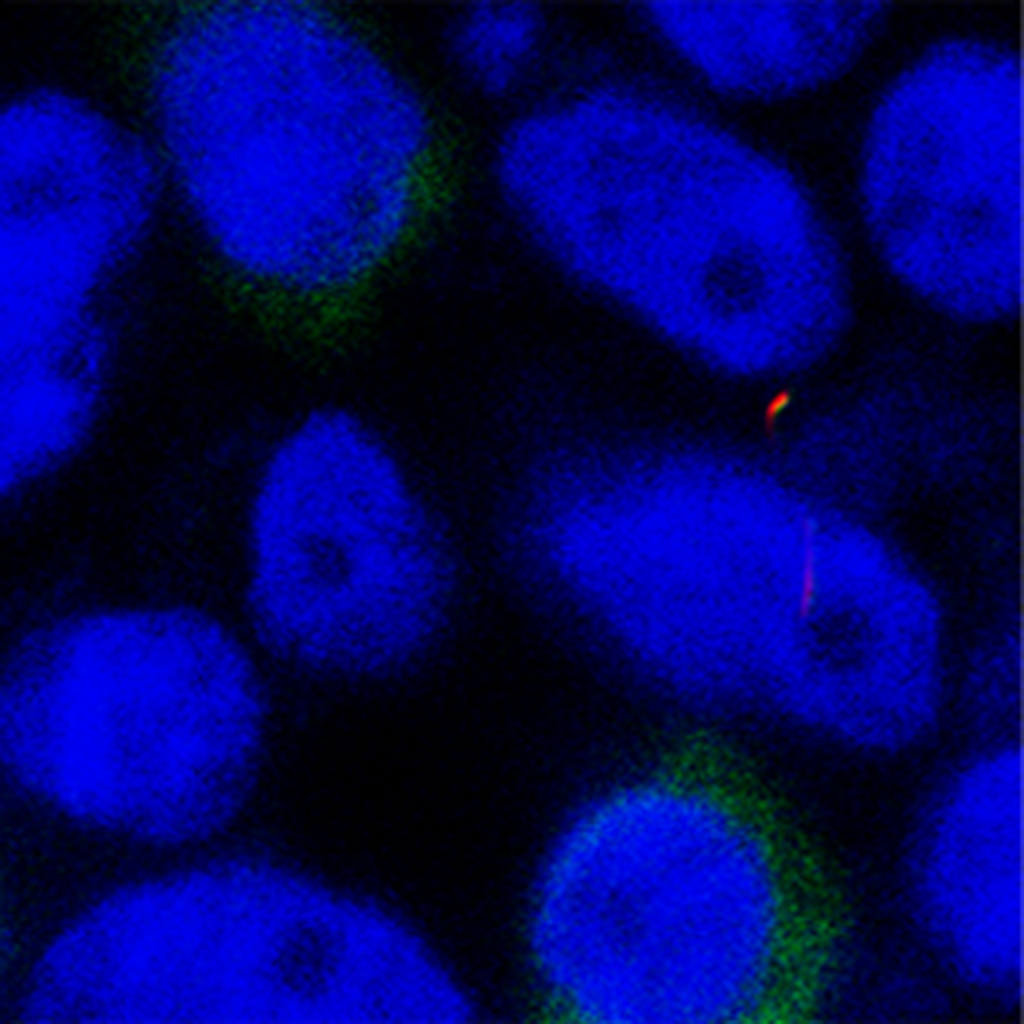

Supplement: Supplementary file 7 — Source Data Fig. 6 [file 44319_2024_92_MOESM7_ESM.zip › Figure 6C/4.tif]

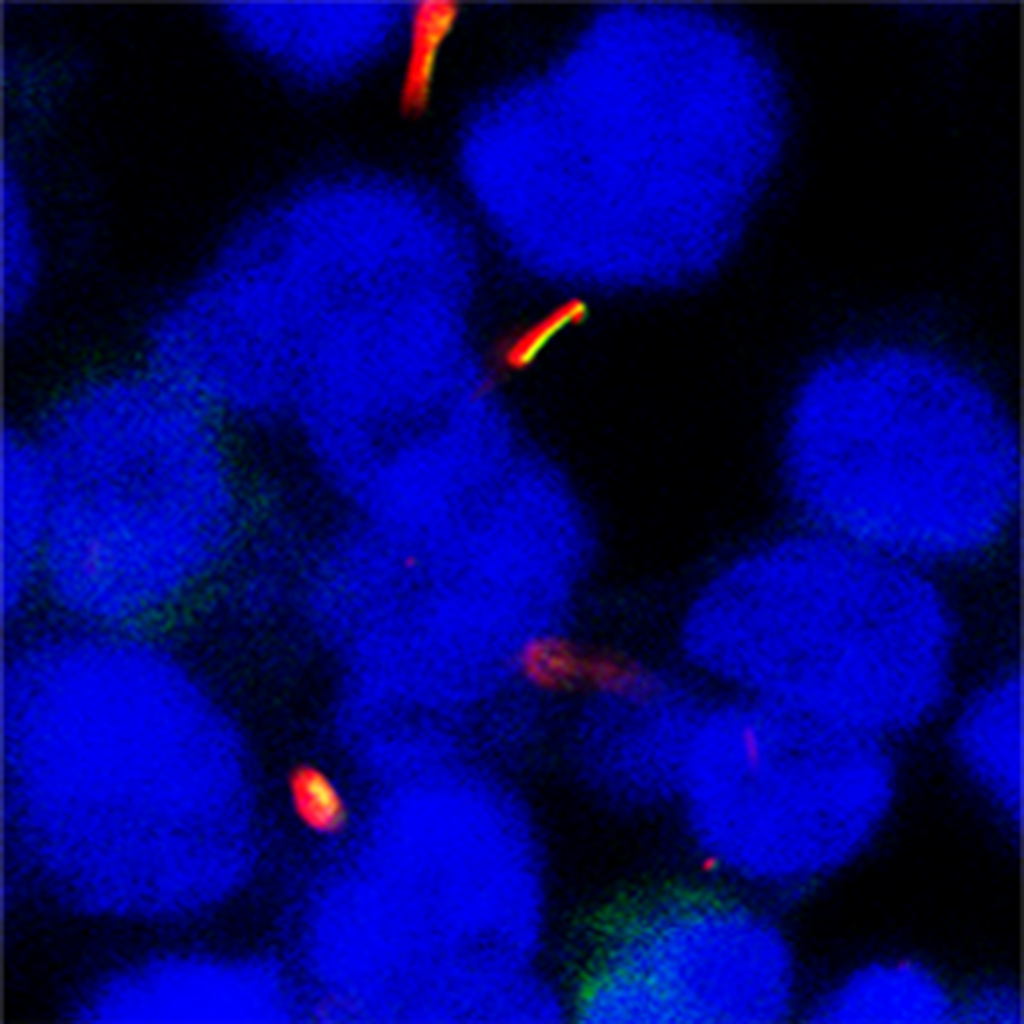

Supplement: Supplementary file 7 — Source Data Fig. 6 [file 44319_2024_92_MOESM7_ESM.zip › Figure 6C/5.tif]

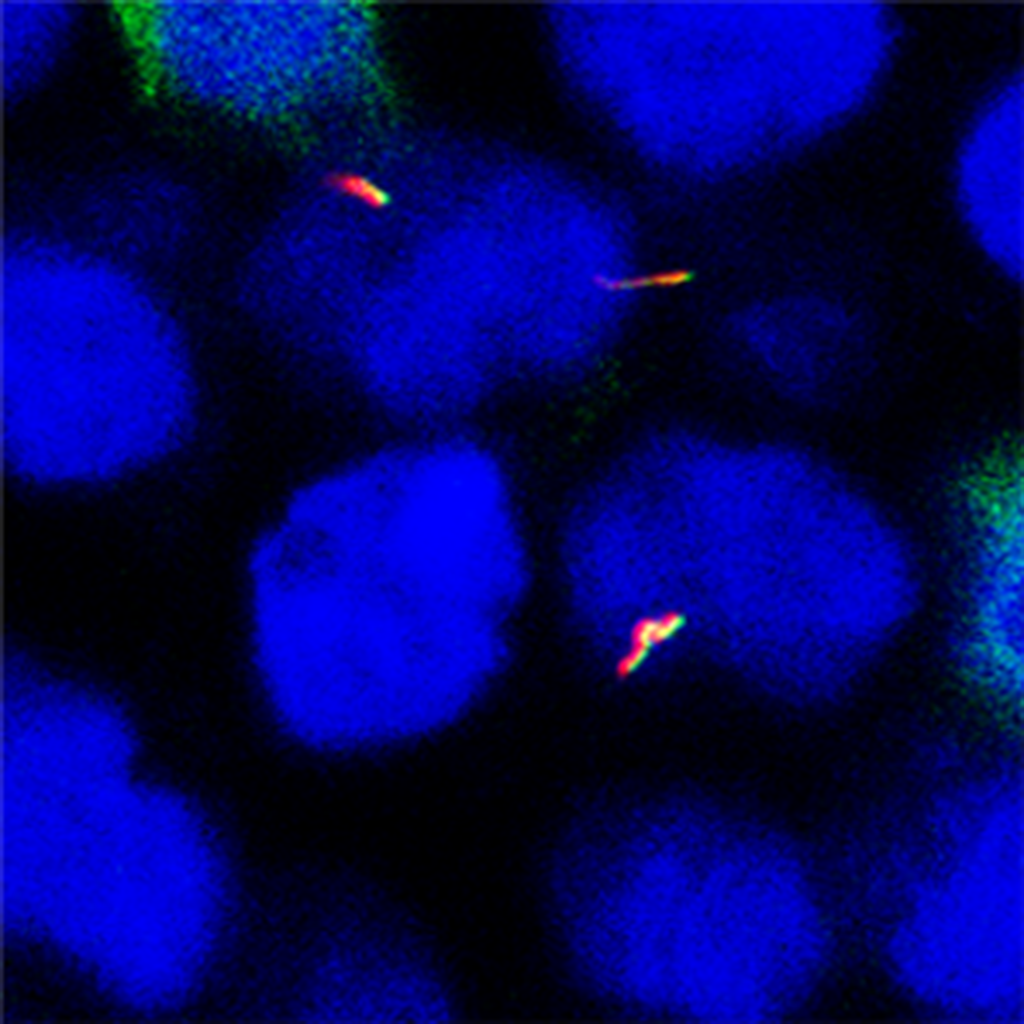

Supplement: Supplementary file 7 — Source Data Fig. 6 [file 44319_2024_92_MOESM7_ESM.zip › Figure 6C/6.tif]

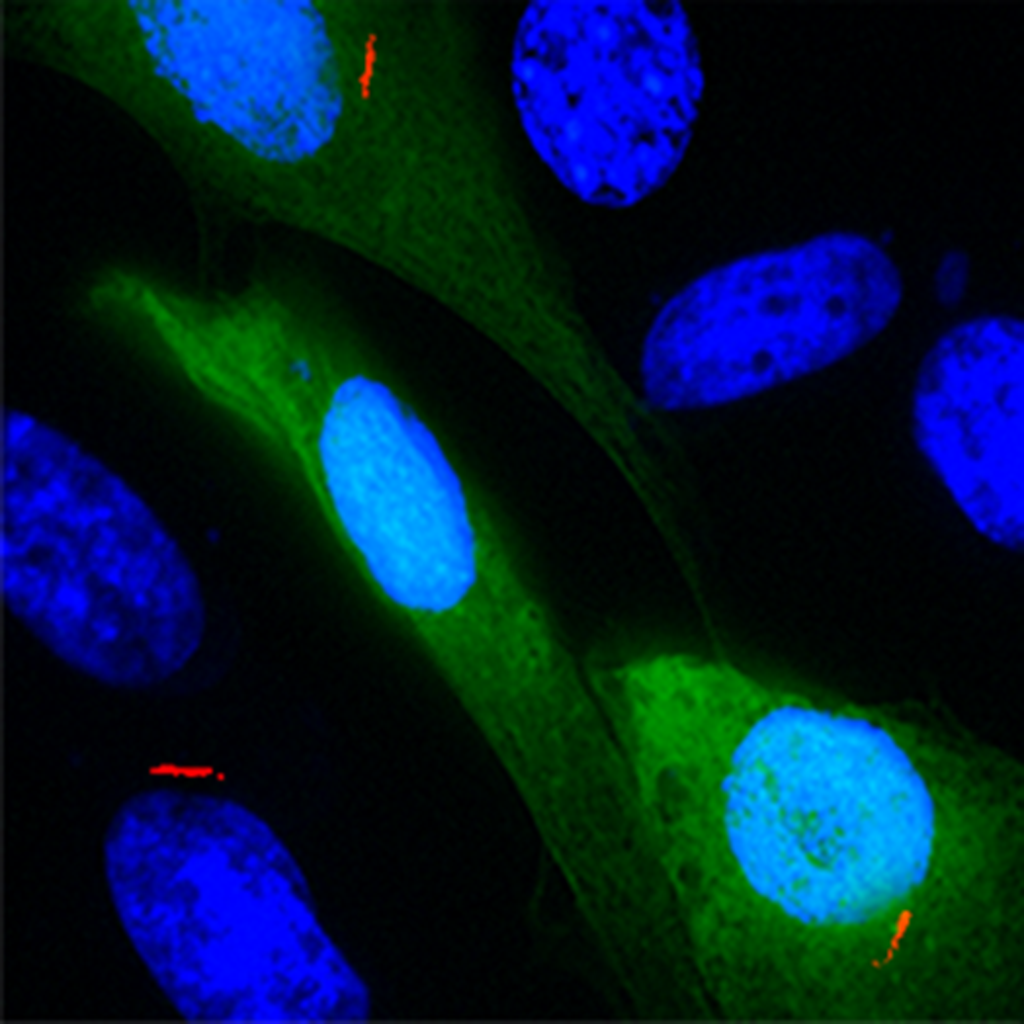

Supplement: Supplementary file 7 — Source Data Fig. 6 [file 44319_2024_92_MOESM7_ESM.zip › Figure 6E/1.tif]

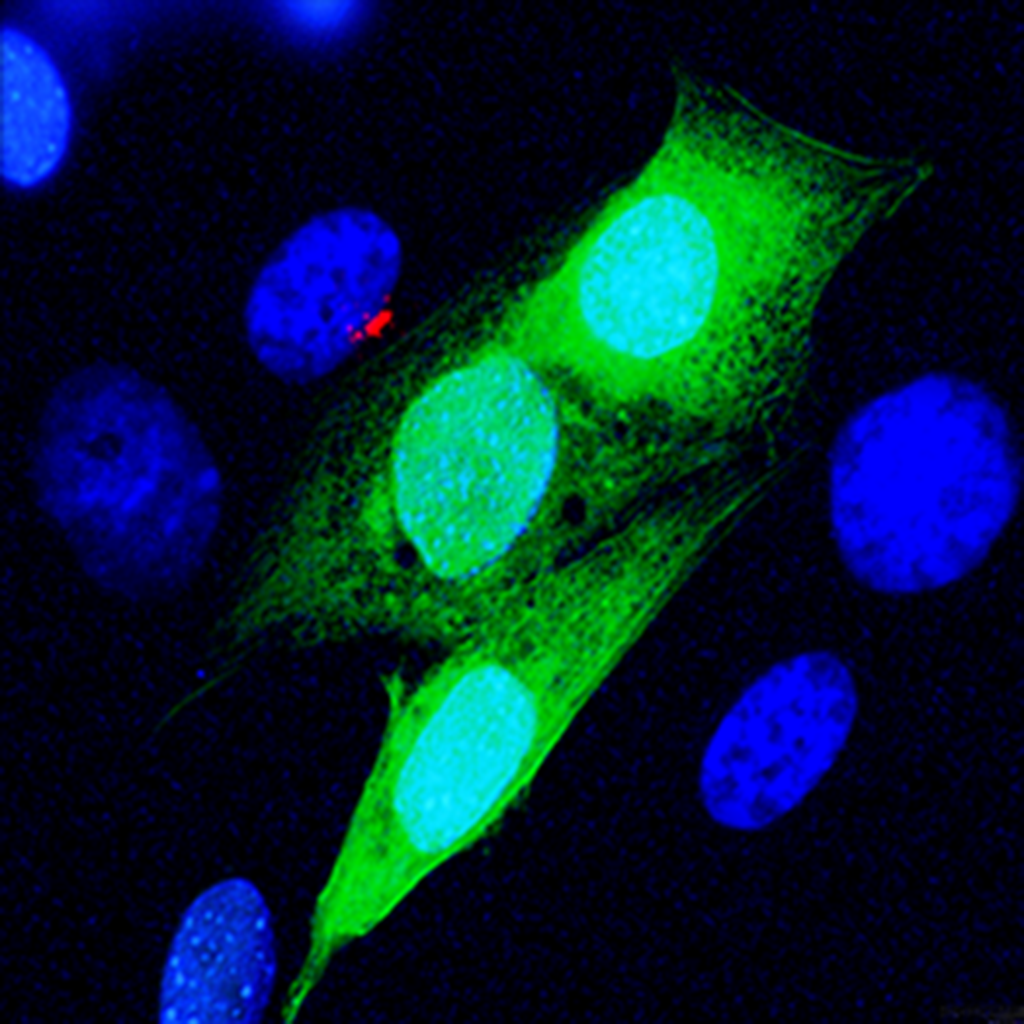

Supplement: Supplementary file 7 — Source Data Fig. 6 [file 44319_2024_92_MOESM7_ESM.zip › Figure 6E/2.tif]

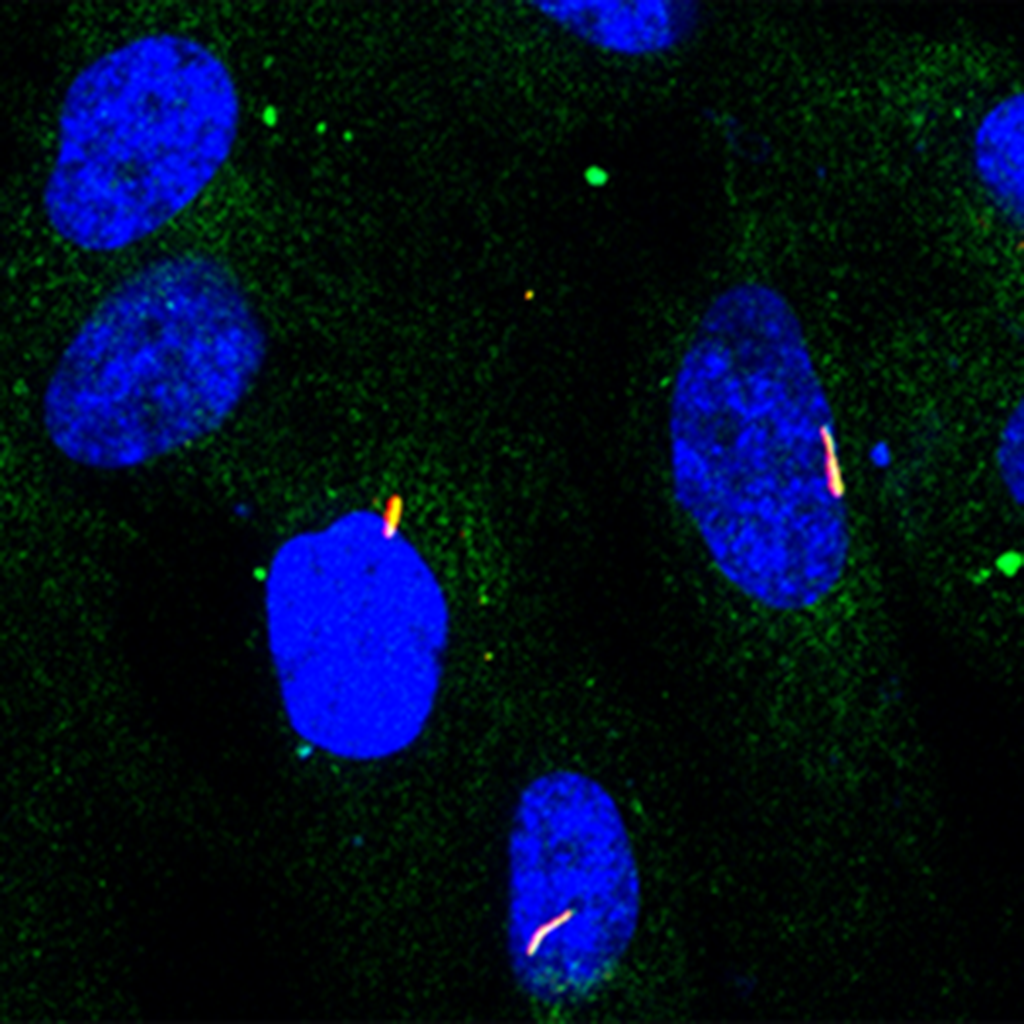

Supplement: Supplementary file 7 — Source Data Fig. 6 [file 44319_2024_92_MOESM7_ESM.zip › Figure 6E/3.tif]

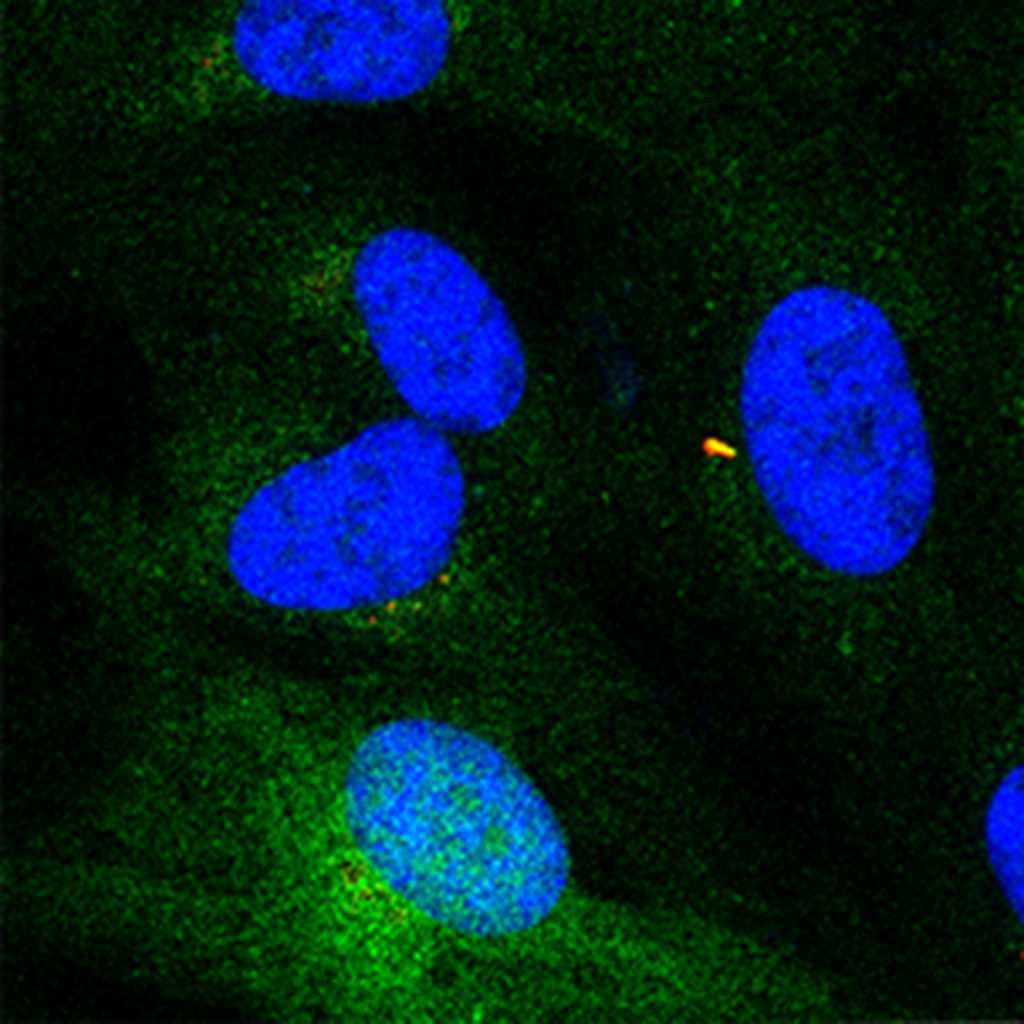

Supplement: Supplementary file 7 — Source Data Fig. 6 [file 44319_2024_92_MOESM7_ESM.zip › Figure 6E/4.tif]

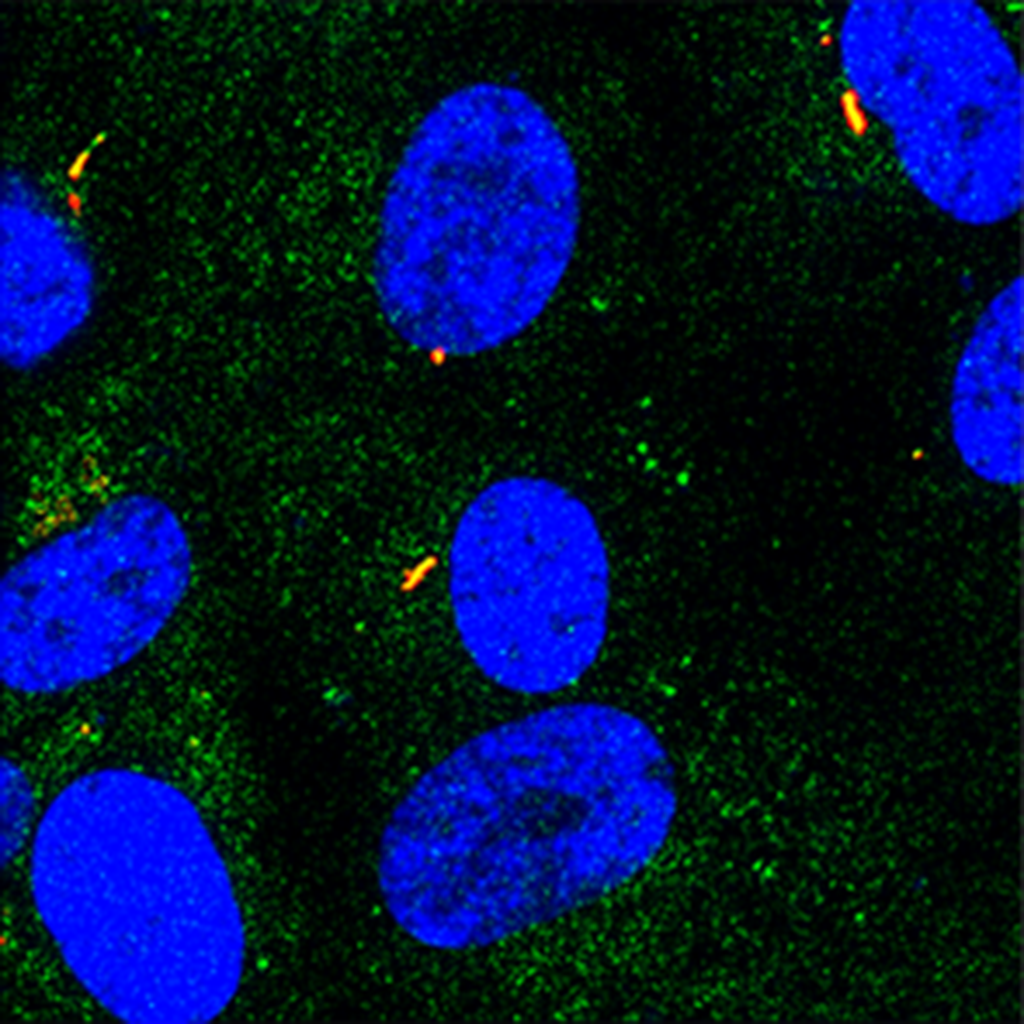

Supplement: Supplementary file 7 — Source Data Fig. 6 [file 44319_2024_92_MOESM7_ESM.zip › Figure 6E/5.tif]

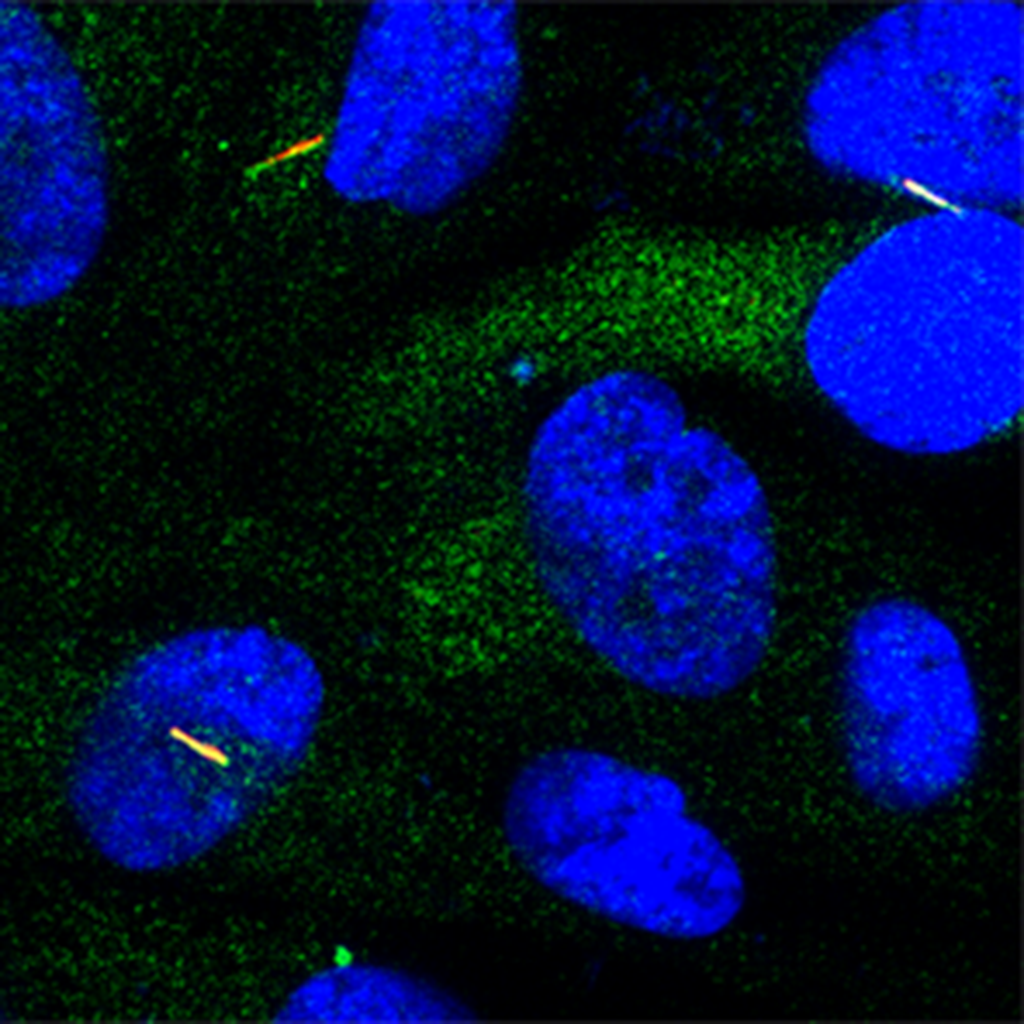

Supplement: Supplementary file 7 — Source Data Fig. 6 [file 44319_2024_92_MOESM7_ESM.zip › Figure 6E/6.tif]

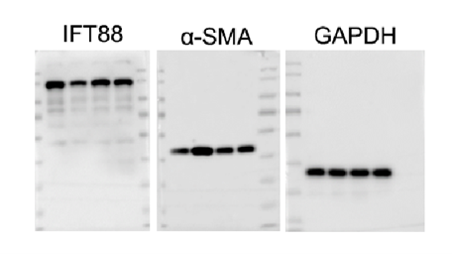

Supplement: Supplementary file 7 — Source Data Fig. 6 [file 44319_2024_92_MOESM7_ESM.zip › Figure 6G/Figure 6G.tif]

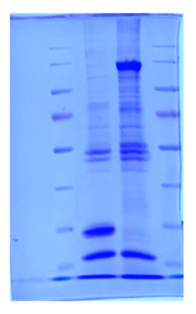

Supplement: Supplementary file 8 — Figure EV Source Data [file 44319_2024_92_MOESM8_ESM.zip › Appendix Figure S1/Appendix Figure S1.tif]

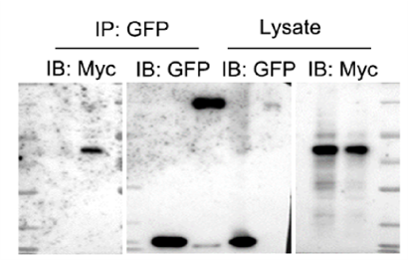

Supplement: Supplementary file 8 — Figure EV Source Data [file 44319_2024_92_MOESM8_ESM.zip › Appendix Figure S2/Appendix Figure S2A/Appendix Figure S2A.tif]

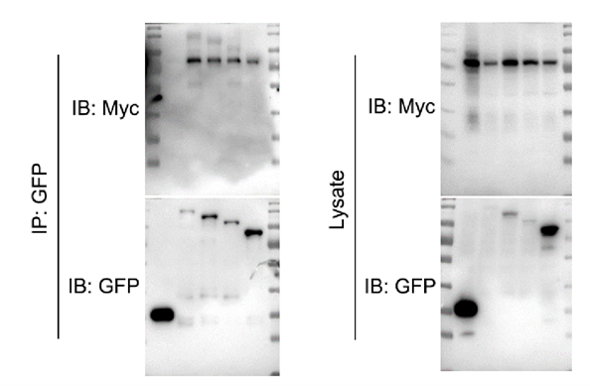

Supplement: Supplementary file 8 — Figure EV Source Data [file 44319_2024_92_MOESM8_ESM.zip › Appendix Figure S2/Appendix Figure S2B/Appendix Figure S2B.tif]

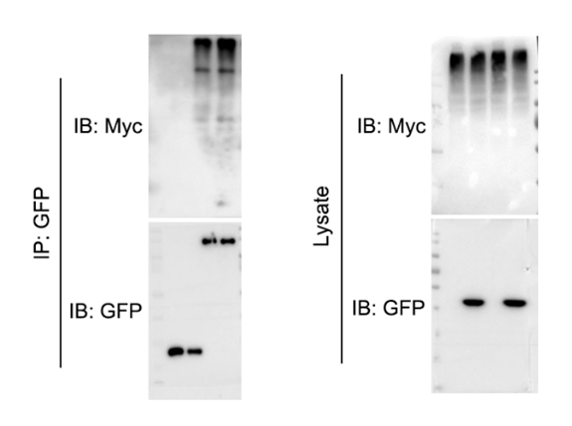

Supplement: Supplementary file 8 — Figure EV Source Data [file 44319_2024_92_MOESM8_ESM.zip › Appendix Figure S3/Appendix Figure S3A/Appendix Figure S3A.tif]

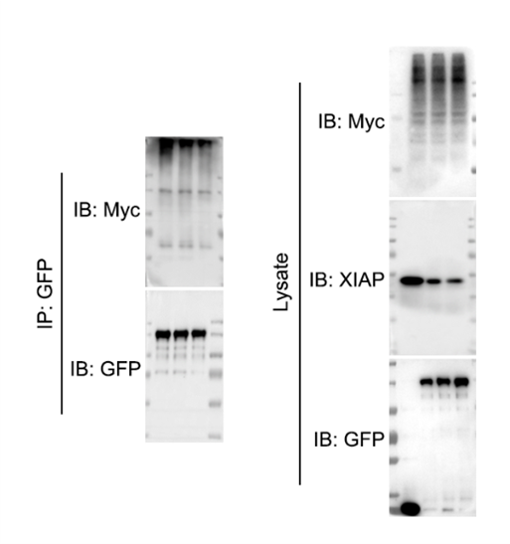

Supplement: Supplementary file 8 — Figure EV Source Data [file 44319_2024_92_MOESM8_ESM.zip › Appendix Figure S3/Appendix Figure S3B/Appendix Figure S3B.tif]

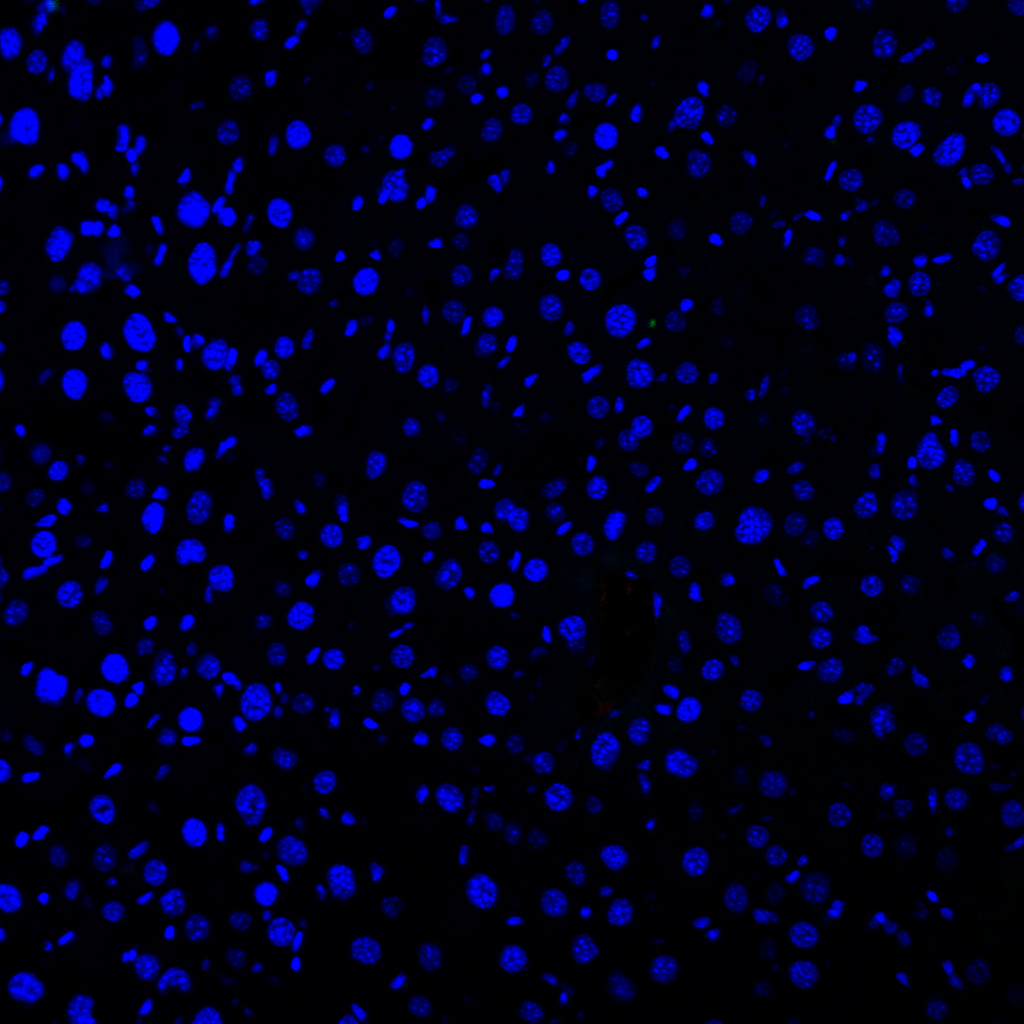

Supplement: Supplementary file 8 — Figure EV Source Data [file 44319_2024_92_MOESM8_ESM.zip › Figure EV1/Figure EV1A/CCl4 0M.tif]

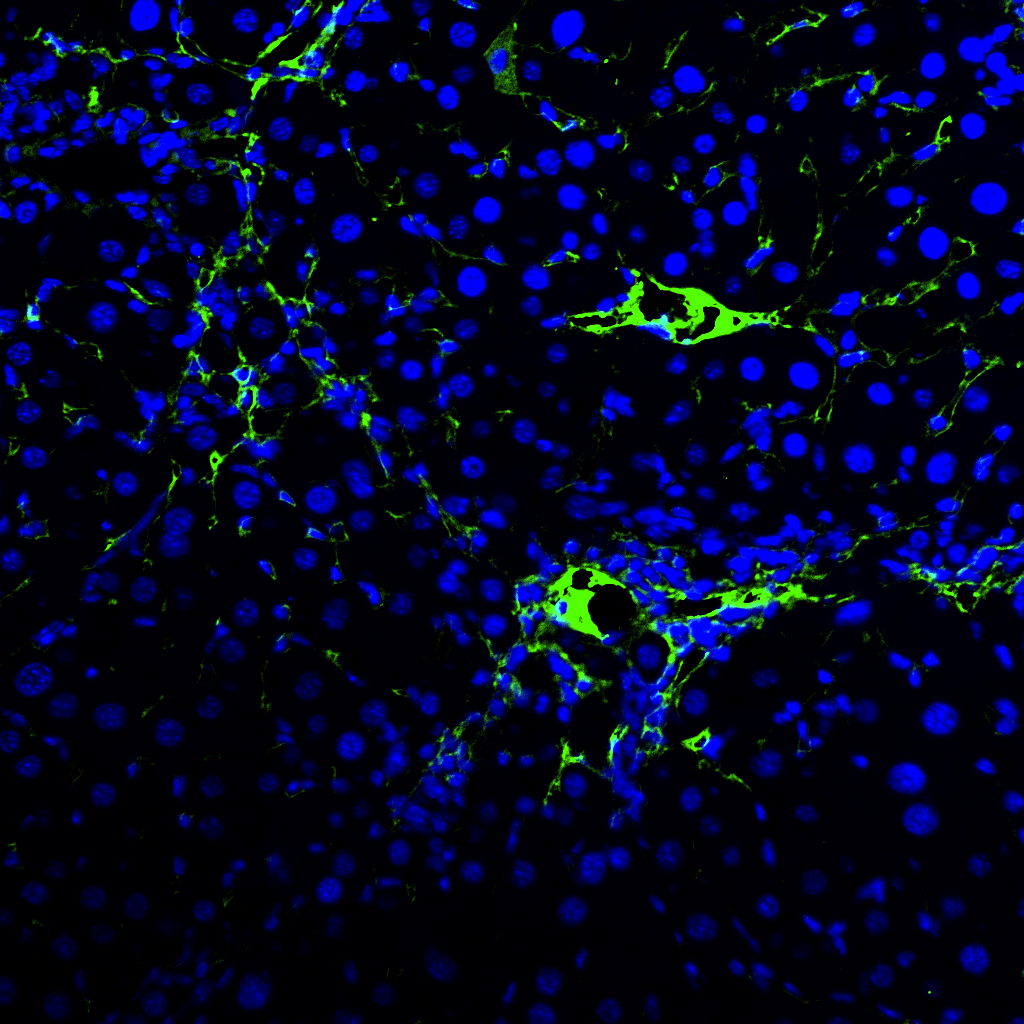

Supplement: Supplementary file 8 — Figure EV Source Data [file 44319_2024_92_MOESM8_ESM.zip › Figure EV1/Figure EV1A/CCl4 1M.tif]

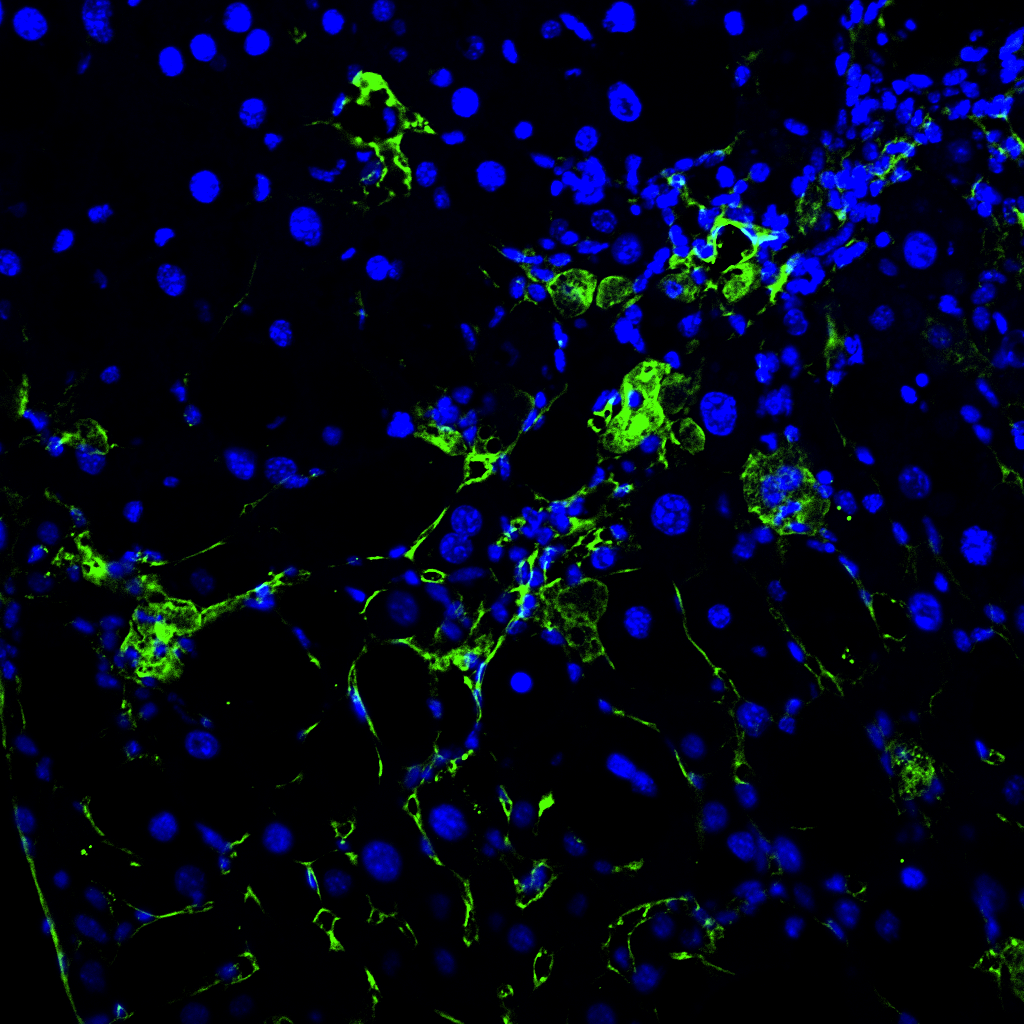

Supplement: Supplementary file 8 — Figure EV Source Data [file 44319_2024_92_MOESM8_ESM.zip › Figure EV1/Figure EV1A/CCl4 2M.tif]

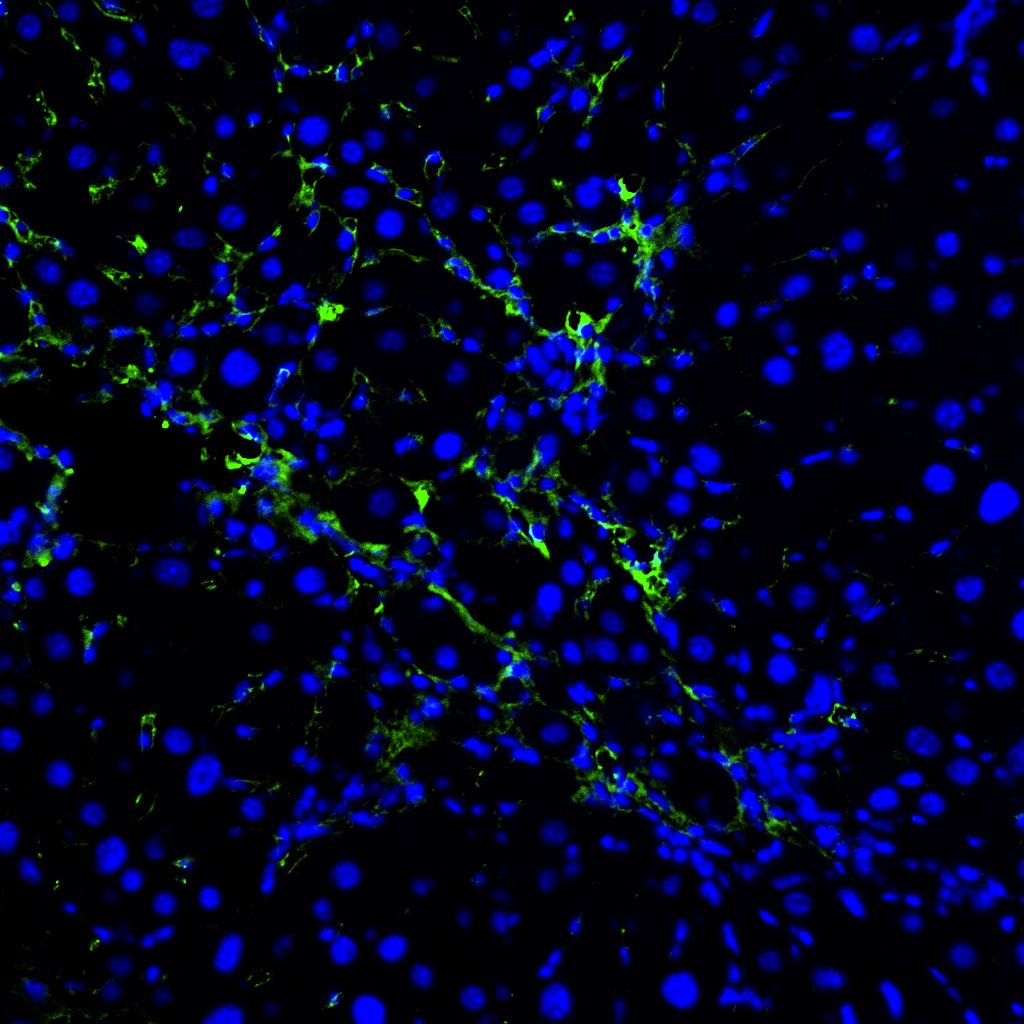

Supplement: Supplementary file 8 — Figure EV Source Data [file 44319_2024_92_MOESM8_ESM.zip › Figure EV1/Figure EV1A/CCl4 3M.tif]

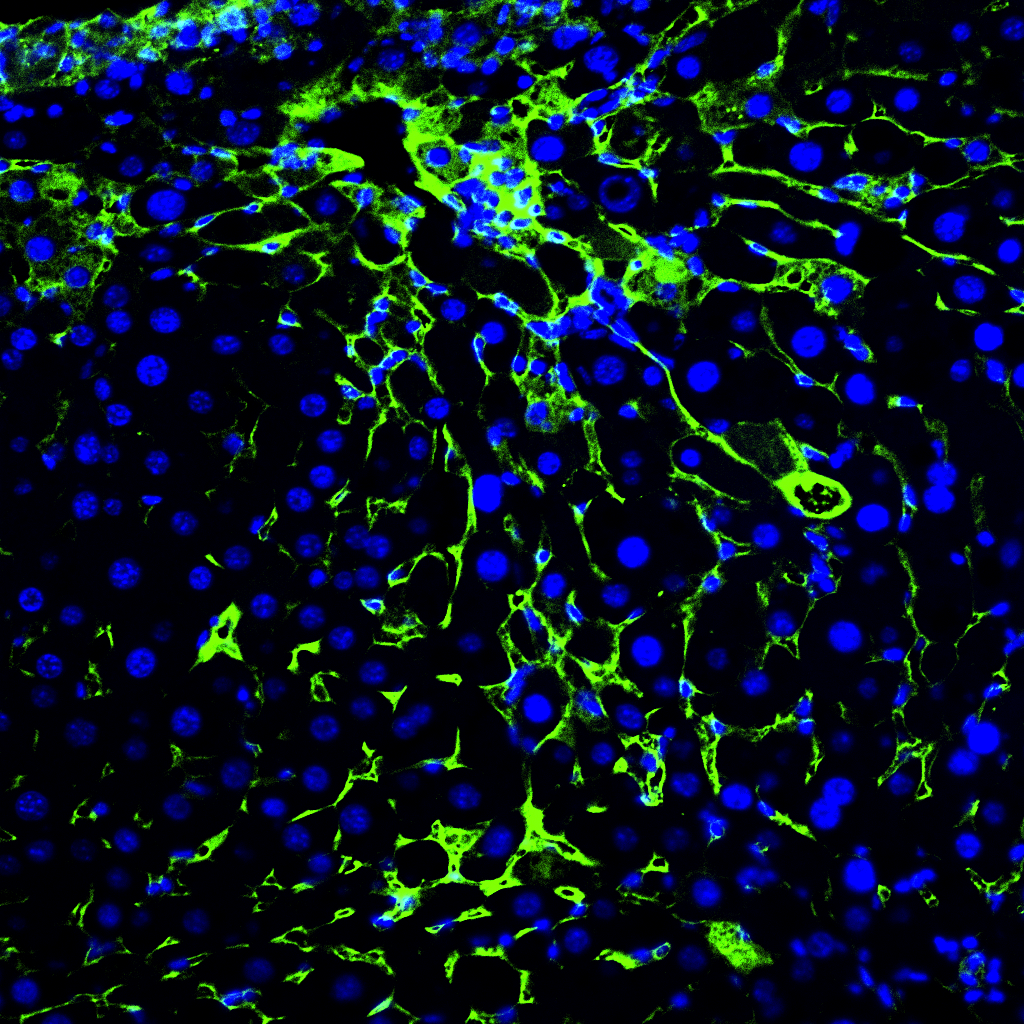

Supplement: Supplementary file 8 — Figure EV Source Data [file 44319_2024_92_MOESM8_ESM.zip › Figure EV1/Figure EV1A/CCl4 4M.tif]

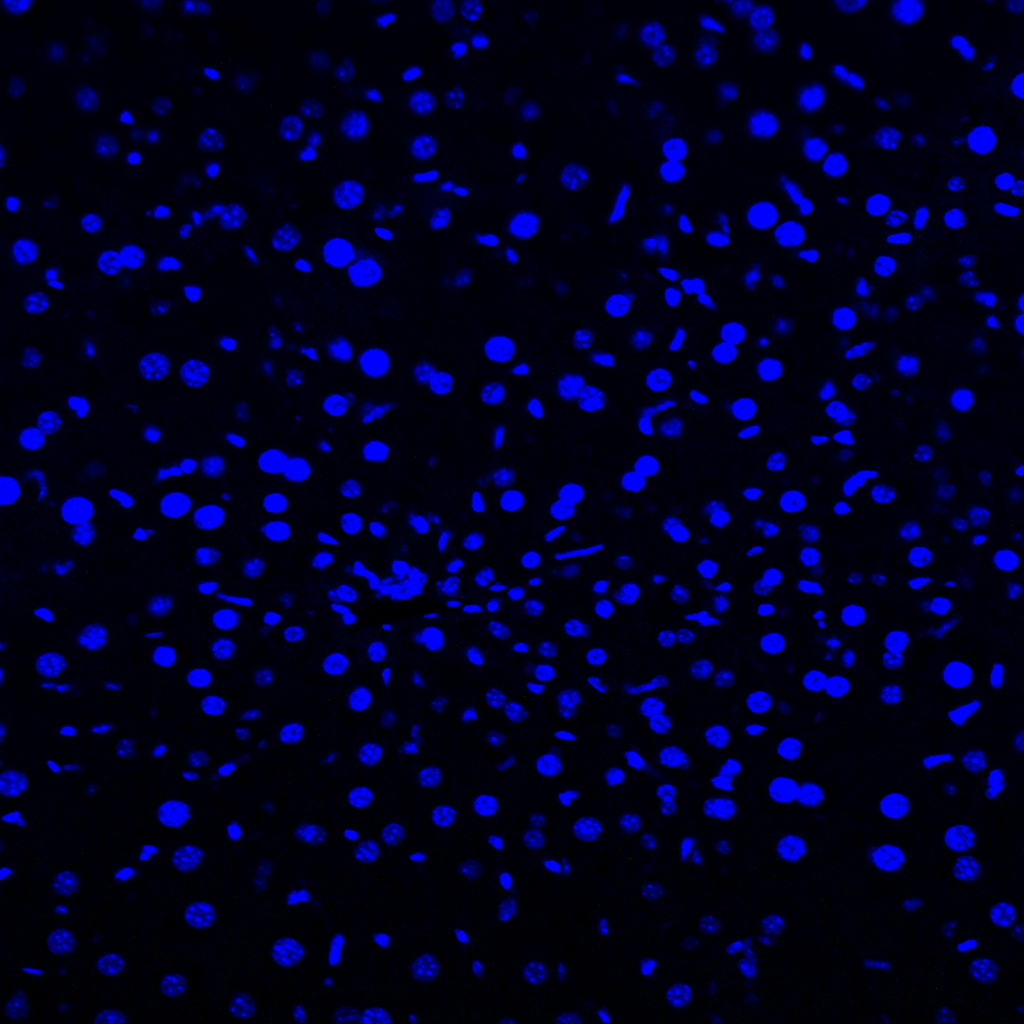

Supplement: Supplementary file 8 — Figure EV Source Data [file 44319_2024_92_MOESM8_ESM.zip › Figure EV1/Figure EV1A/Vehicle 0M.tif]

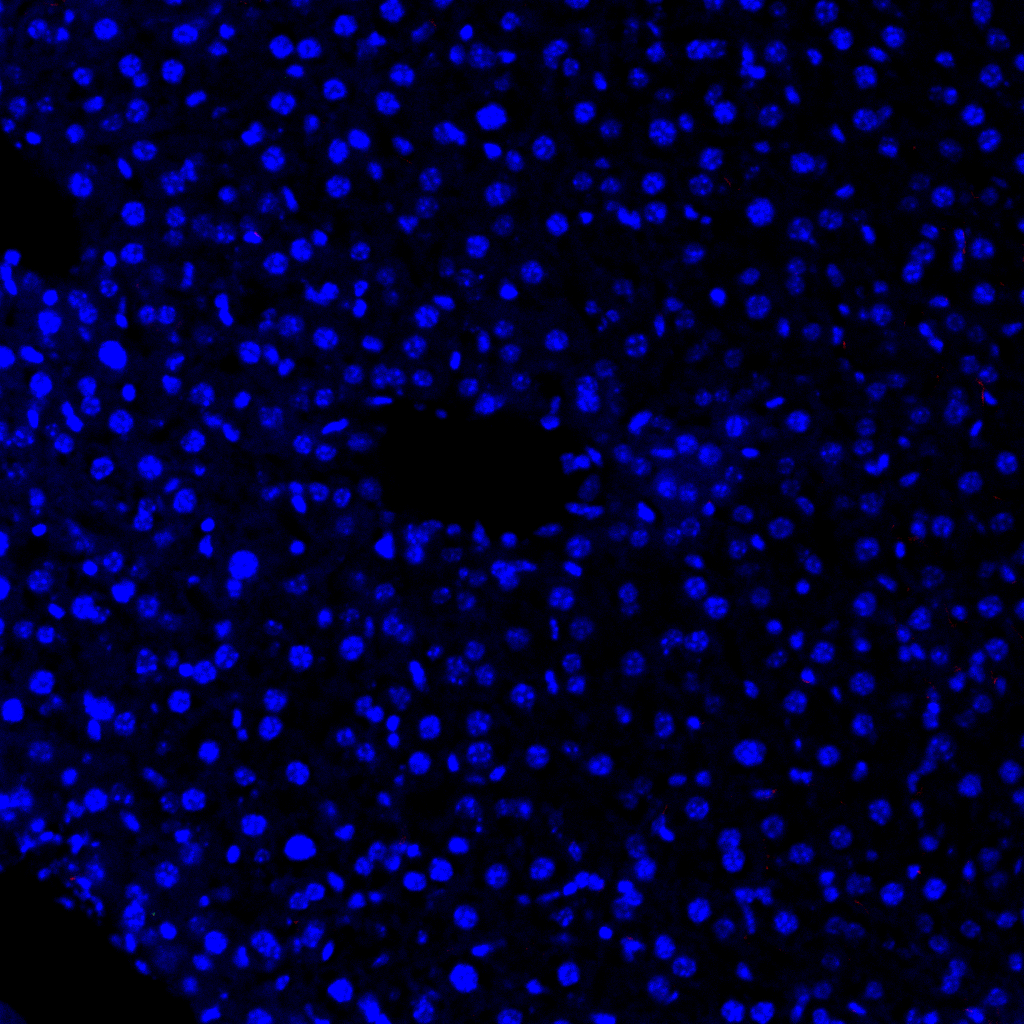

Supplement: Supplementary file 8 — Figure EV Source Data [file 44319_2024_92_MOESM8_ESM.zip › Figure EV1/Figure EV1A/Vehicle 1M.tif]

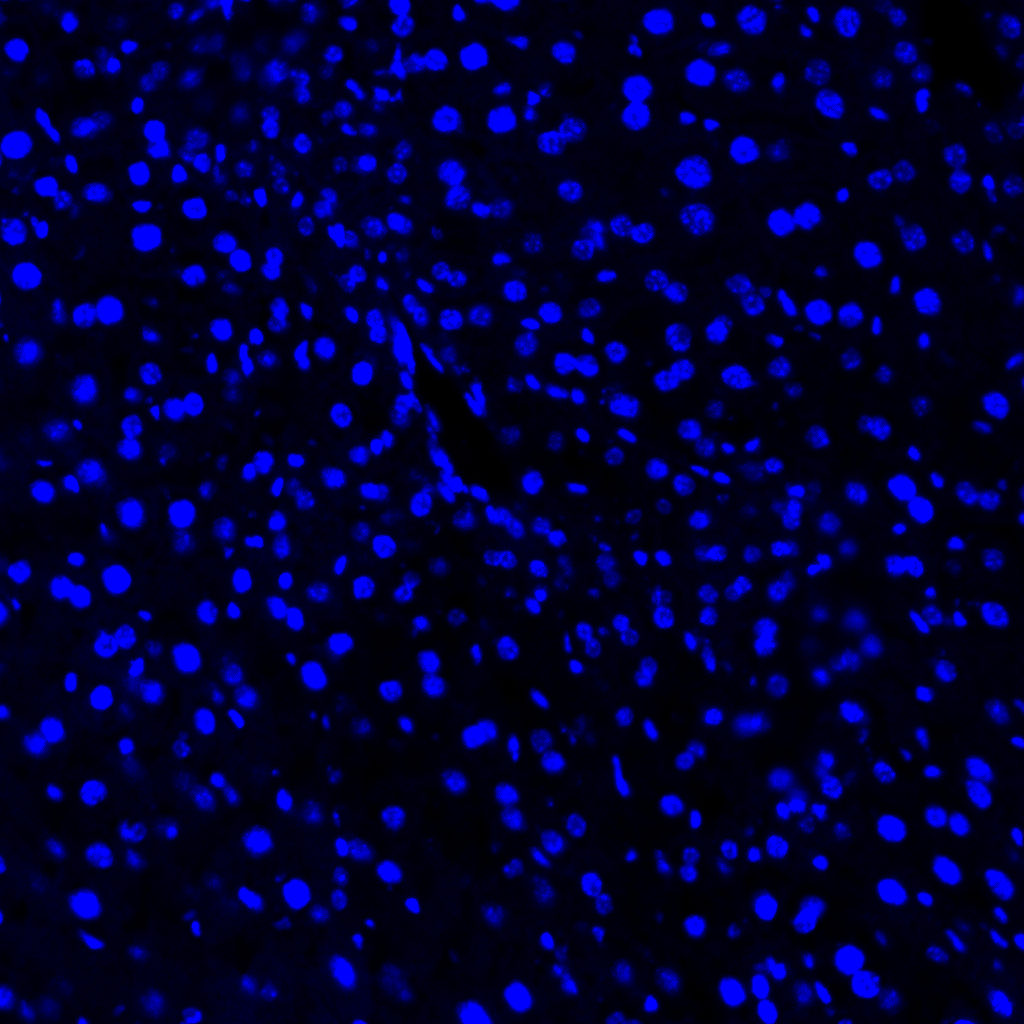

Supplement: Supplementary file 8 — Figure EV Source Data [file 44319_2024_92_MOESM8_ESM.zip › Figure EV1/Figure EV1A/Vehicle 2M.tif]

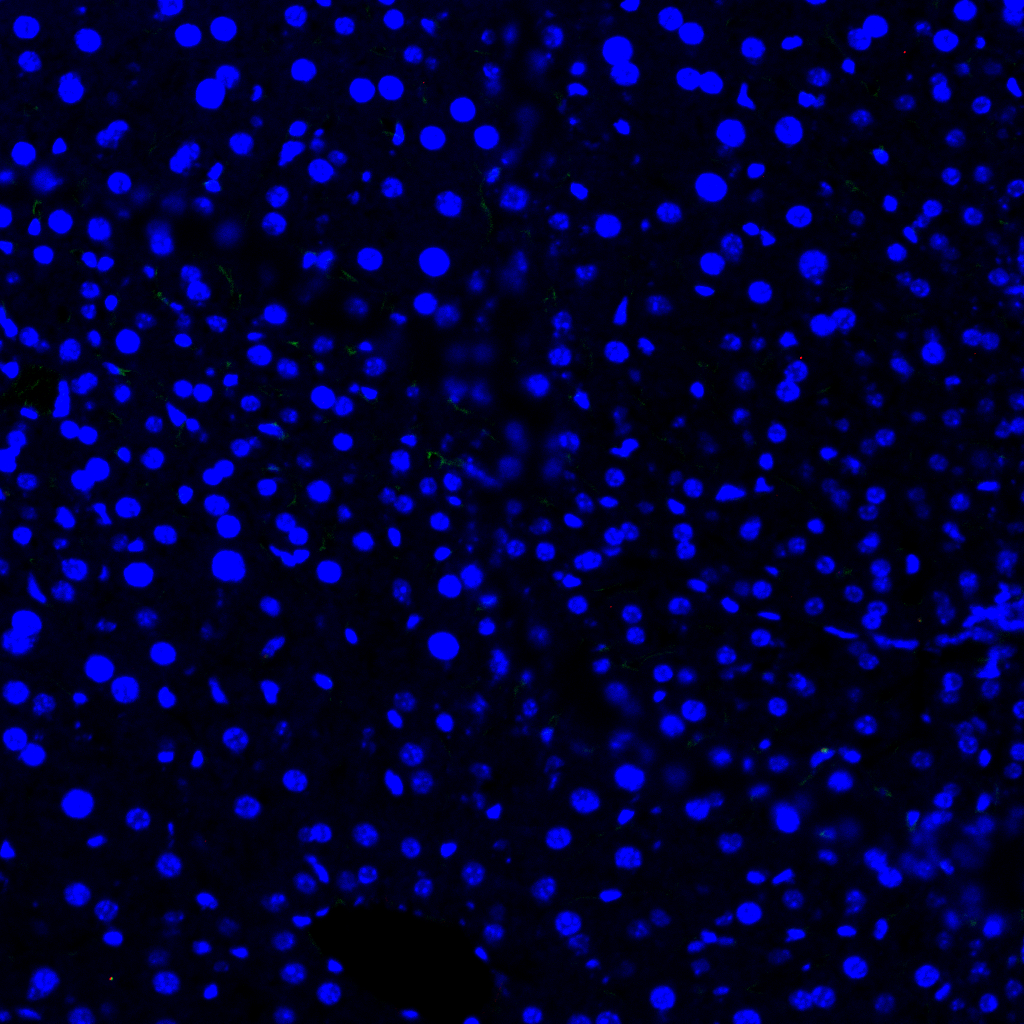

Supplement: Supplementary file 8 — Figure EV Source Data [file 44319_2024_92_MOESM8_ESM.zip › Figure EV1/Figure EV1A/Vehicle 3M.tif]

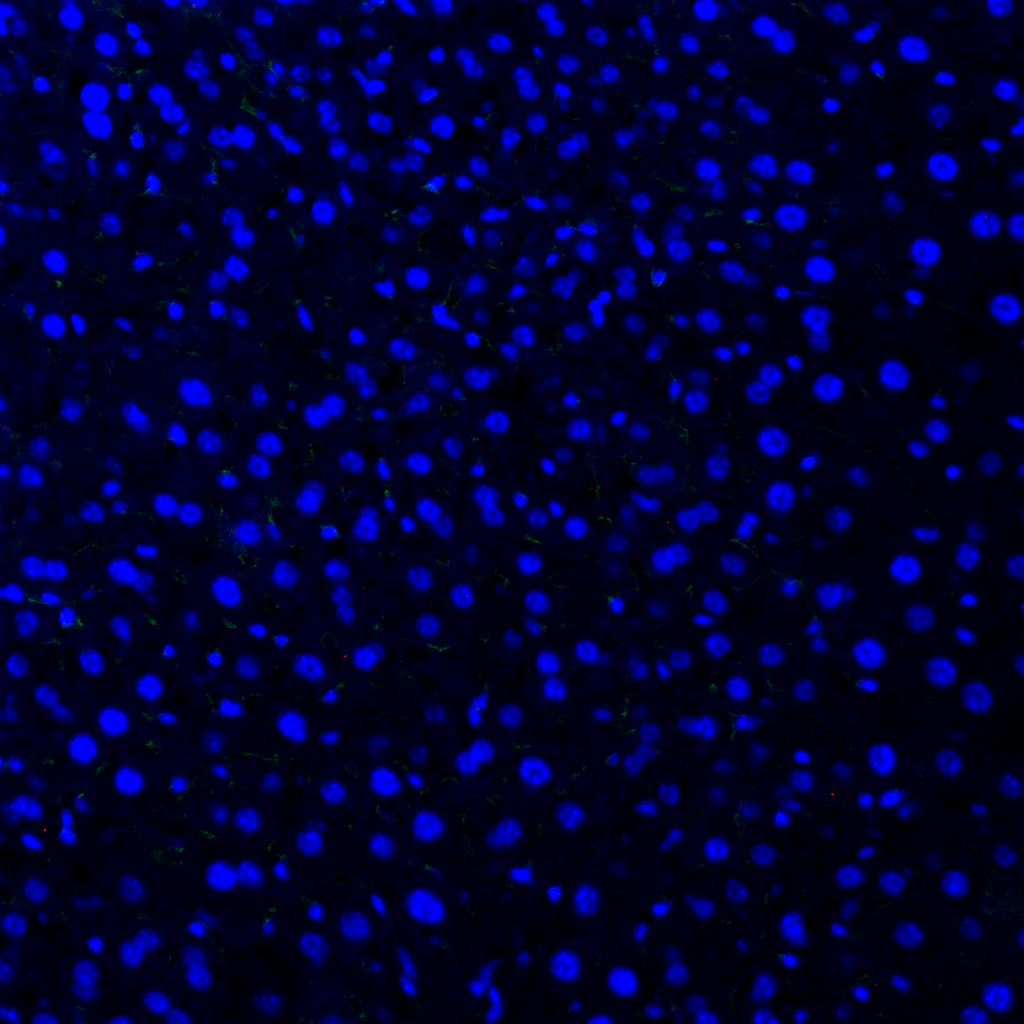

Supplement: Supplementary file 8 — Figure EV Source Data [file 44319_2024_92_MOESM8_ESM.zip › Figure EV1/Figure EV1A/Vehicle 4M.tif]

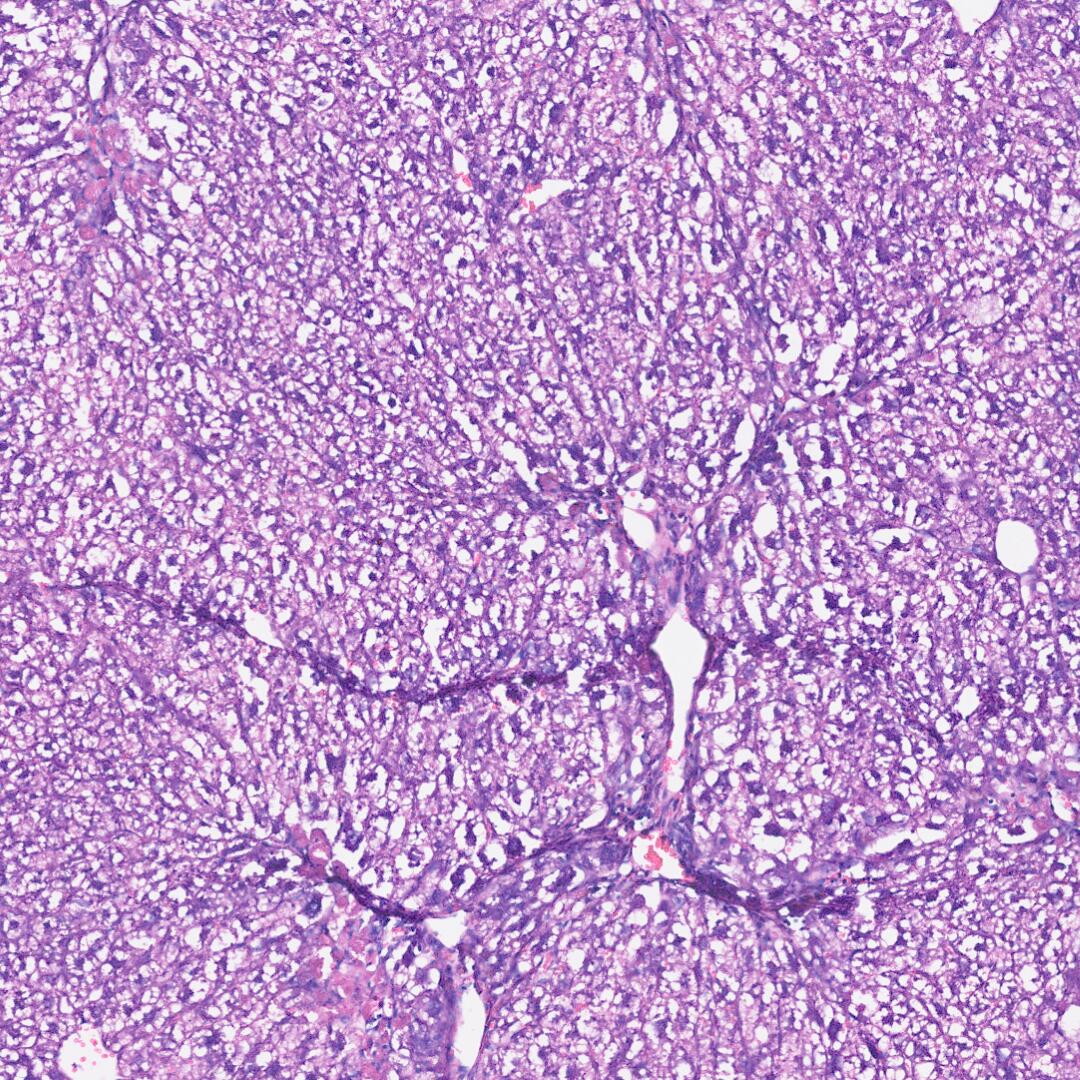

Supplement: Supplementary file 8 — Figure EV Source Data [file 44319_2024_92_MOESM8_ESM.zip › Figure EV1/Figure EV1C/HE CCl4.tif]

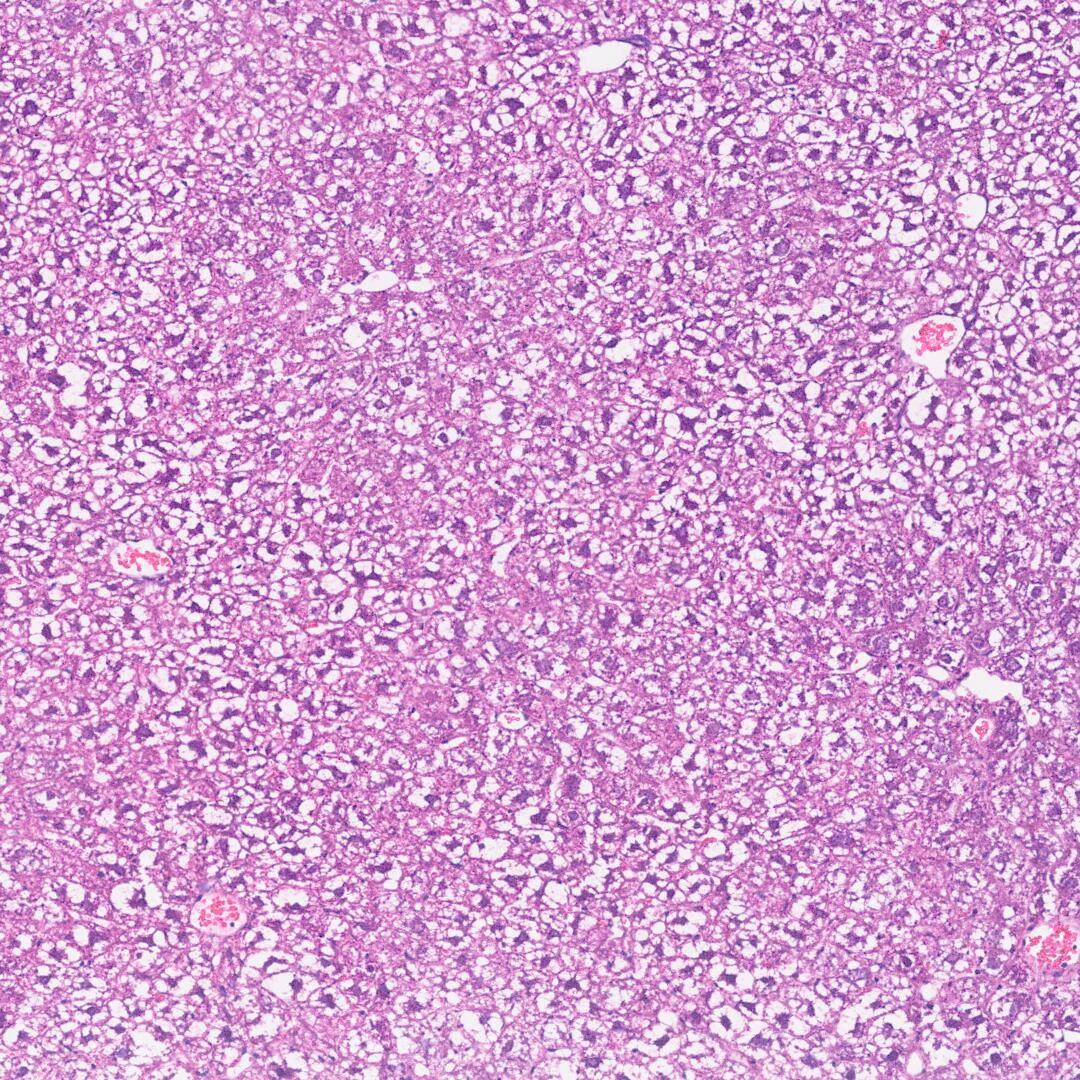

Supplement: Supplementary file 8 — Figure EV Source Data [file 44319_2024_92_MOESM8_ESM.zip › Figure EV1/Figure EV1C/HE Vehicle.tif]

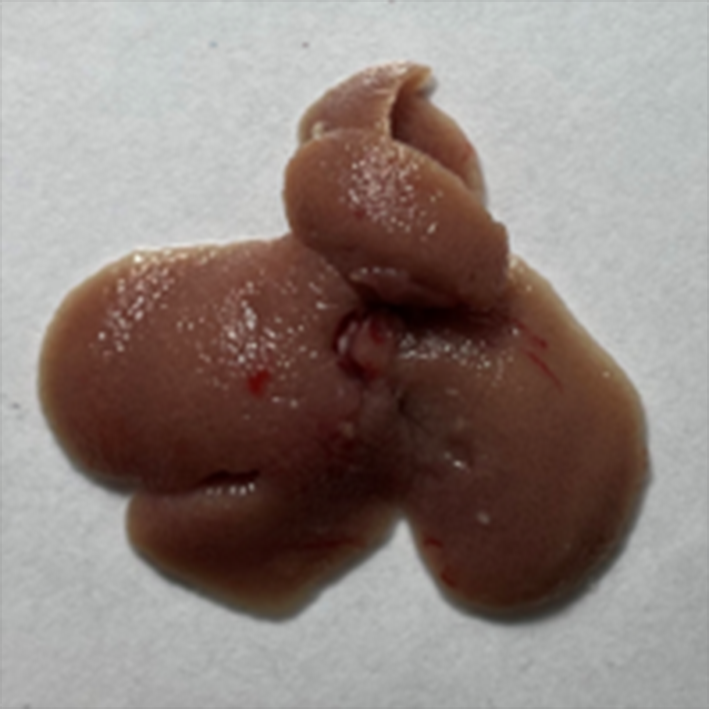

Supplement: Supplementary file 8 — Figure EV Source Data [file 44319_2024_92_MOESM8_ESM.zip › Figure EV1/Figure EV1C/liver CCl4.tif]

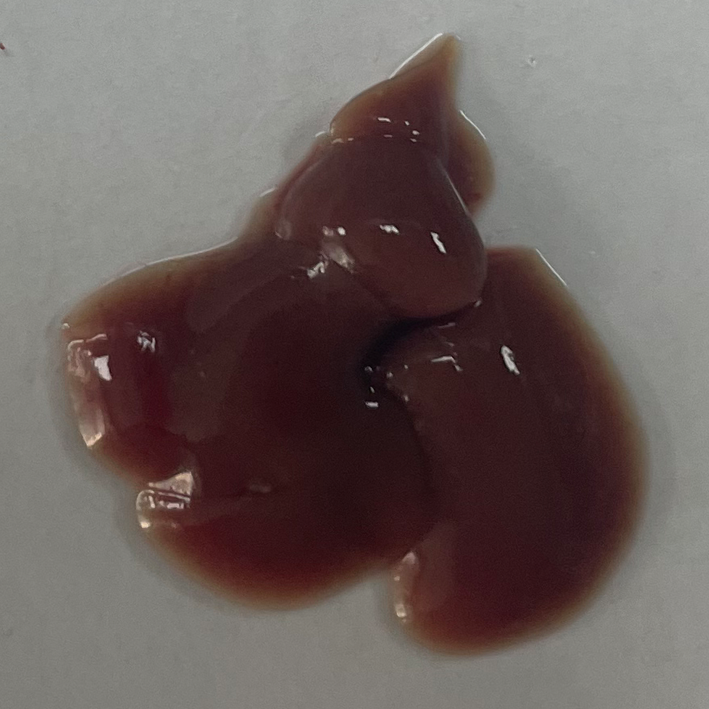

Supplement: Supplementary file 8 — Figure EV Source Data [file 44319_2024_92_MOESM8_ESM.zip › Figure EV1/Figure EV1C/liver Vehicle.tif]

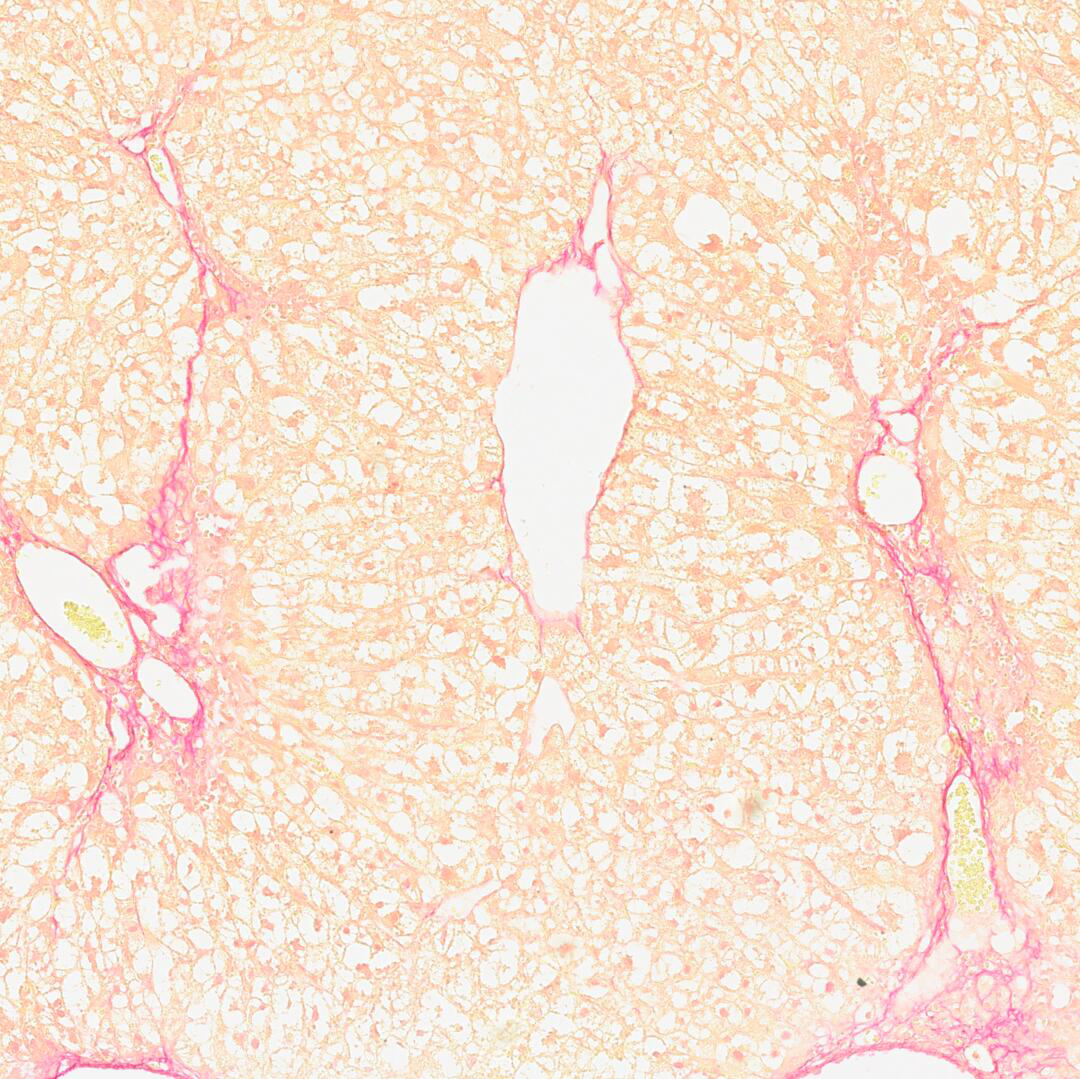

Supplement: Supplementary file 8 — Figure EV Source Data [file 44319_2024_92_MOESM8_ESM.zip › Figure EV1/Figure EV1C/Sirius red CCl4.tif]

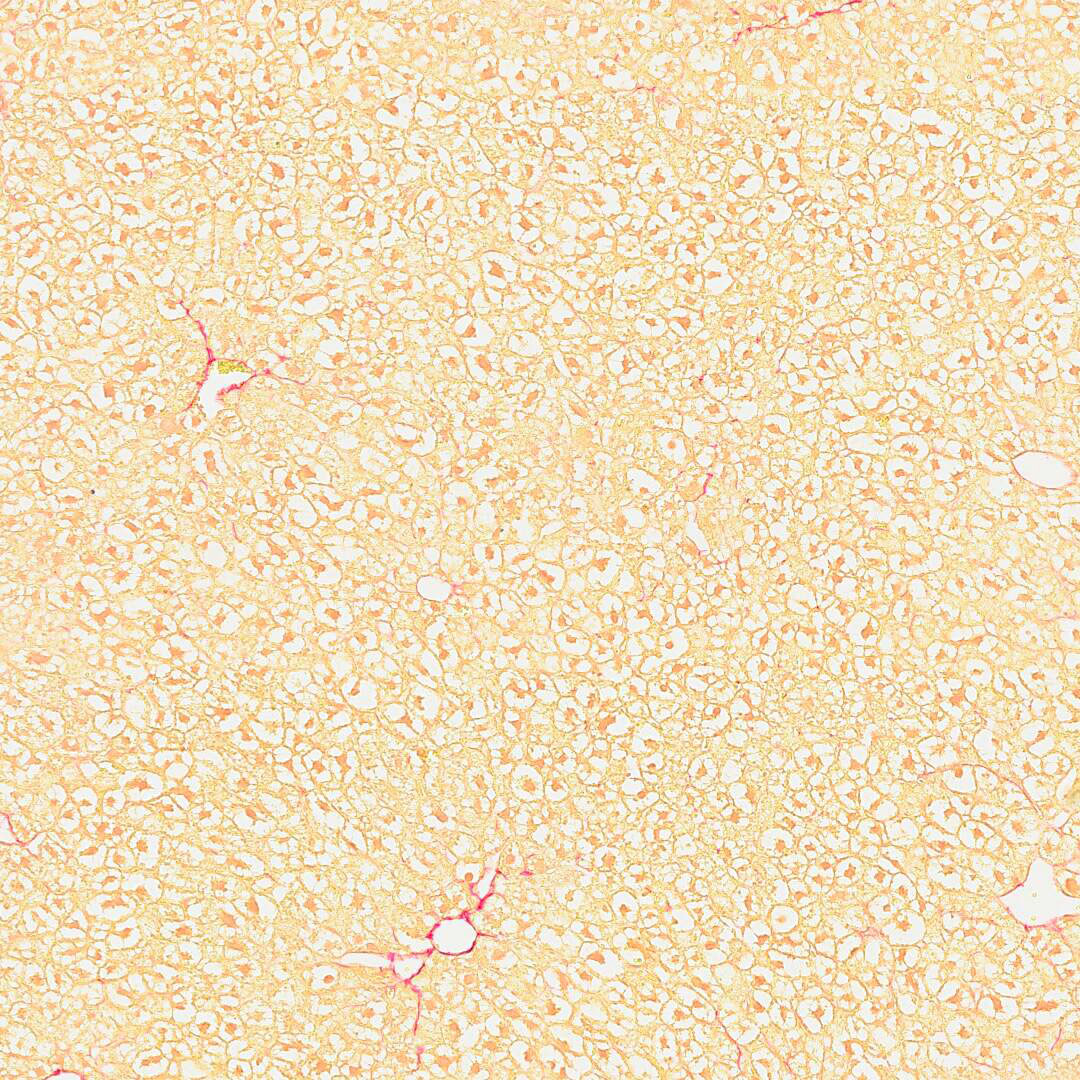

Supplement: Supplementary file 8 — Figure EV Source Data [file 44319_2024_92_MOESM8_ESM.zip › Figure EV1/Figure EV1C/Sirius red Vehicle.tif]

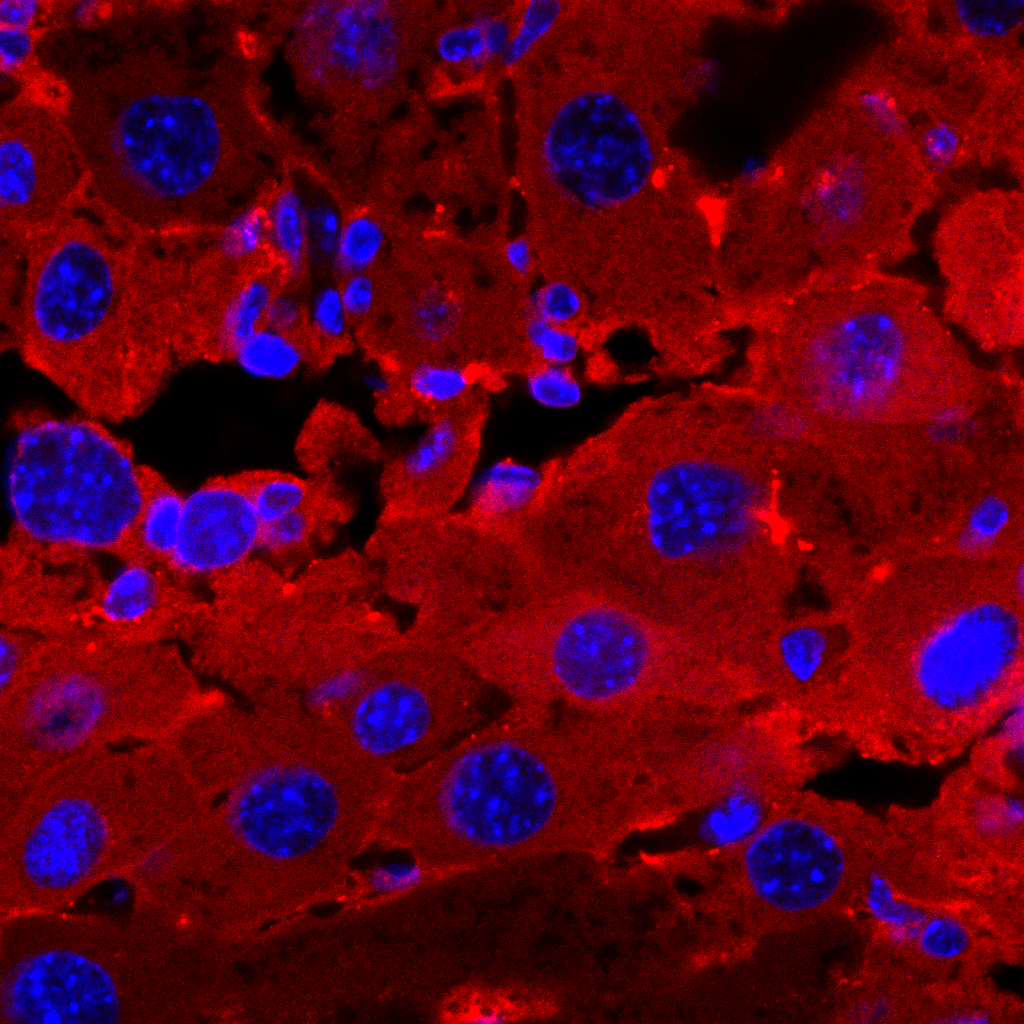

Supplement: Supplementary file 8 — Figure EV Source Data [file 44319_2024_92_MOESM8_ESM.zip › Figure EV2/Figure EV2A/0M.tif]

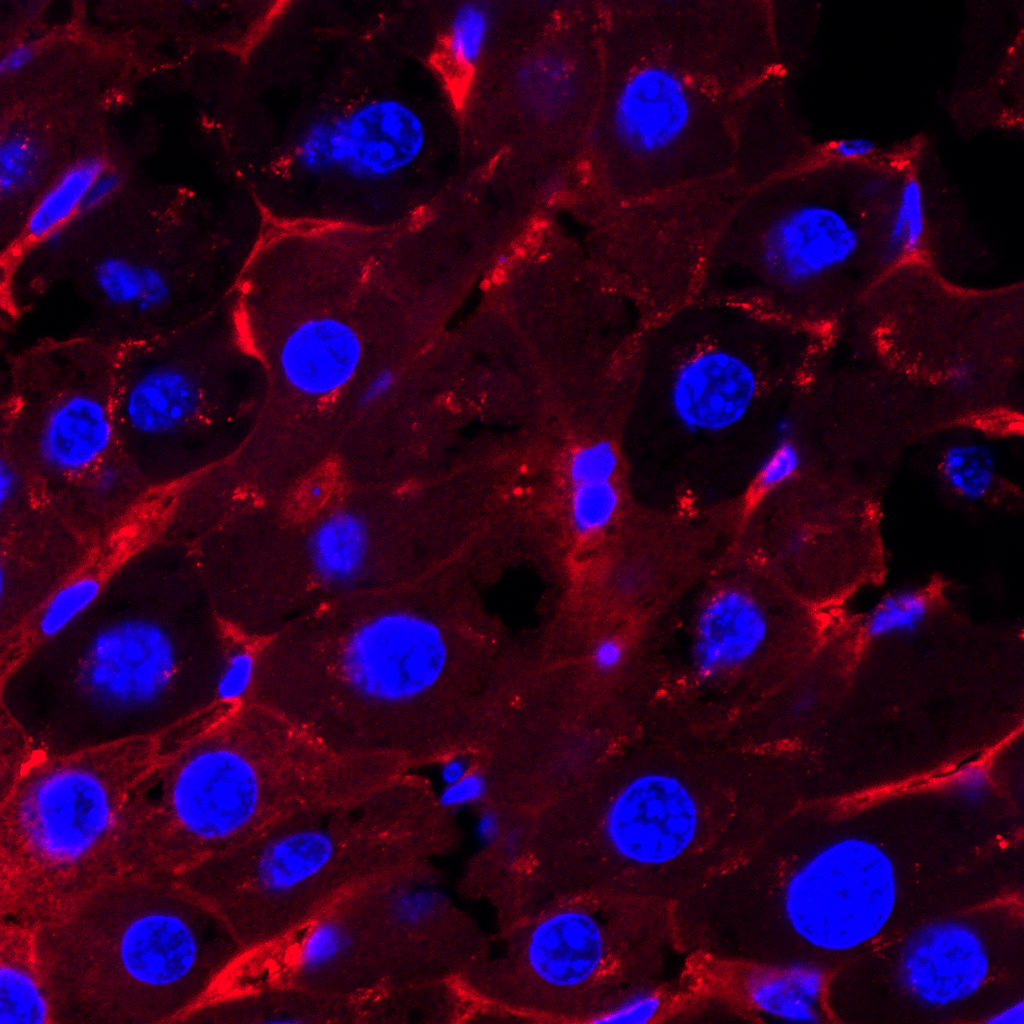

Supplement: Supplementary file 8 — Figure EV Source Data [file 44319_2024_92_MOESM8_ESM.zip › Figure EV2/Figure EV2A/1M.tif]

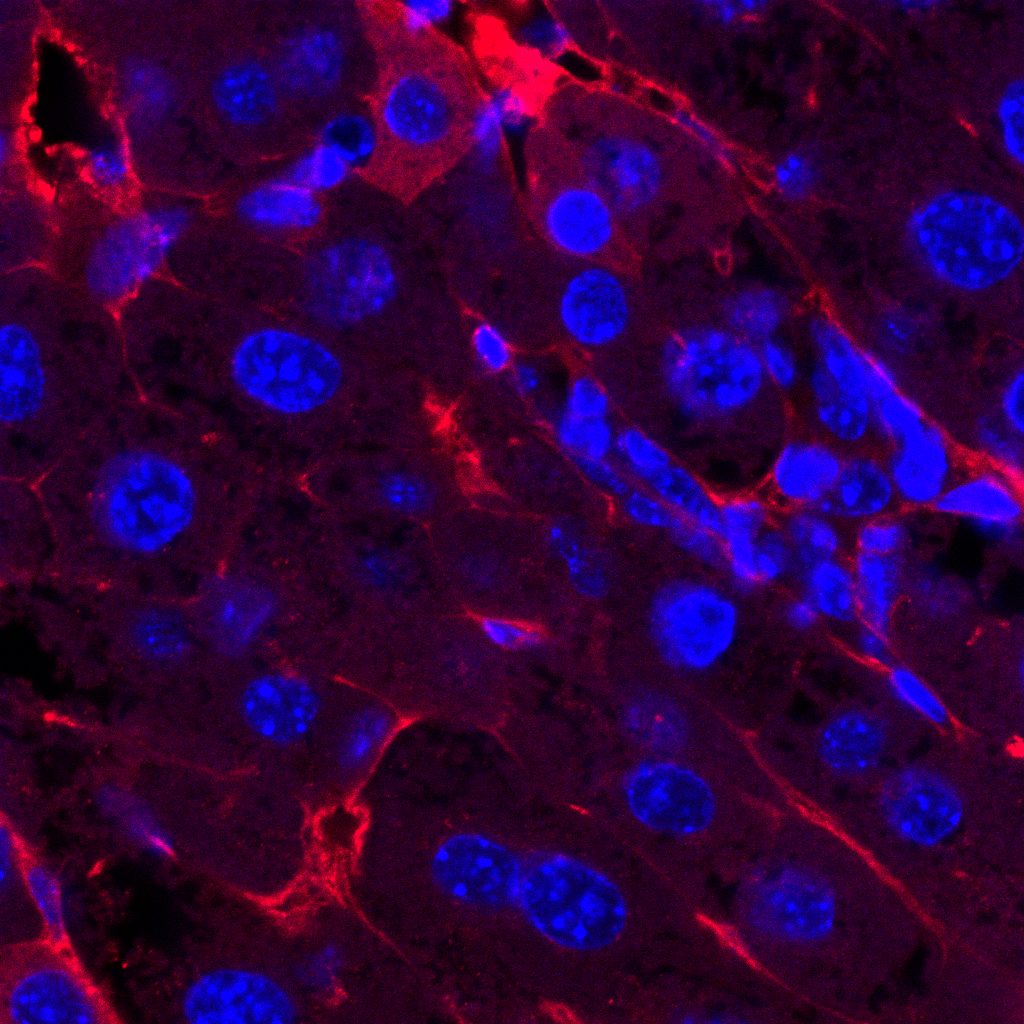

Supplement: Supplementary file 8 — Figure EV Source Data [file 44319_2024_92_MOESM8_ESM.zip › Figure EV2/Figure EV2A/2M.tif]

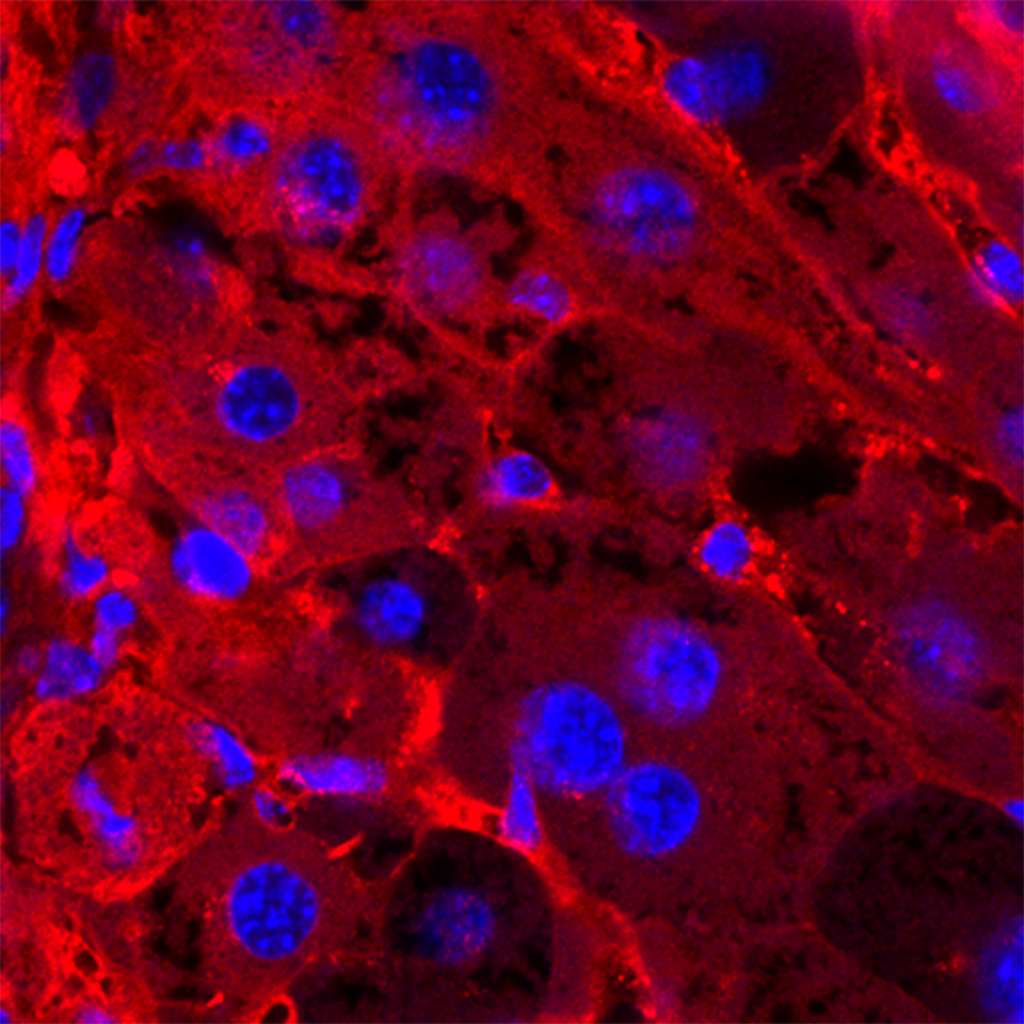

Supplement: Supplementary file 8 — Figure EV Source Data [file 44319_2024_92_MOESM8_ESM.zip › Figure EV2/Figure EV2A/3M.tif]

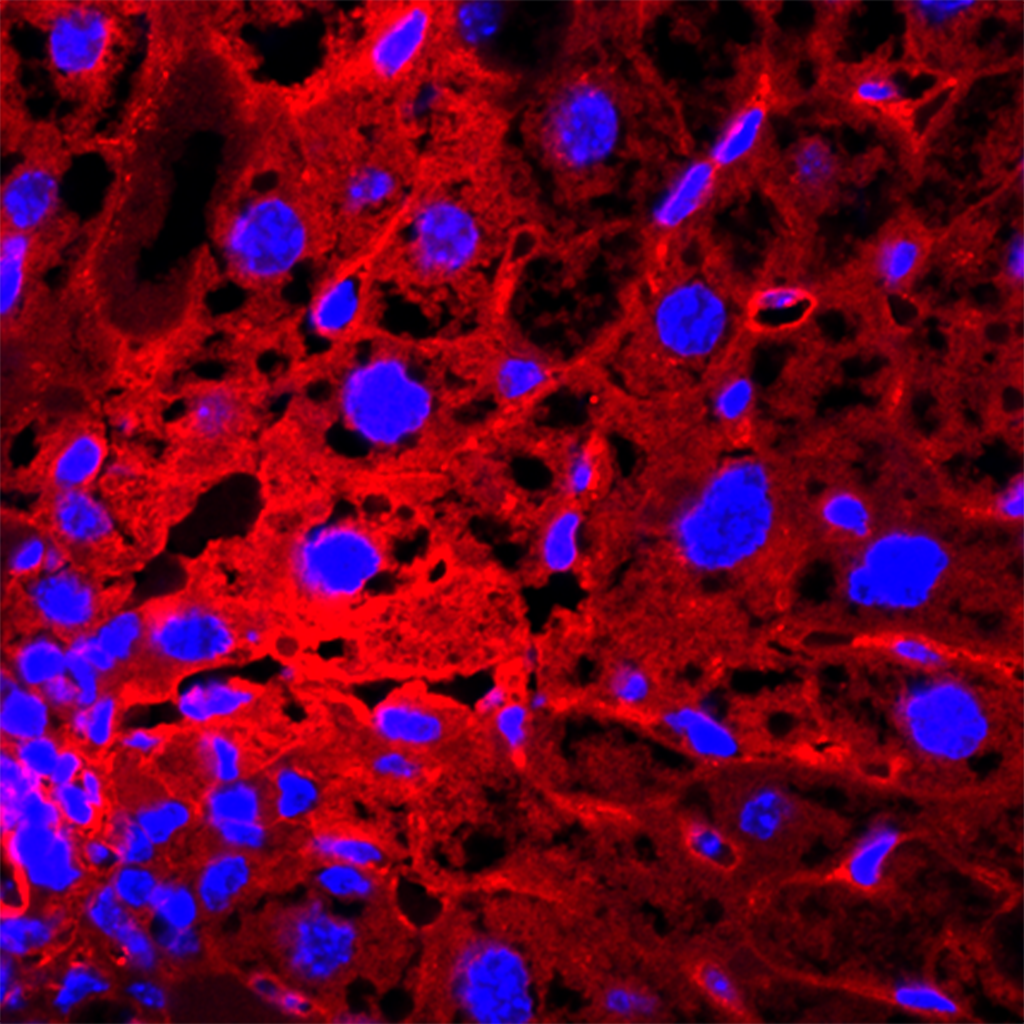

Supplement: Supplementary file 8 — Figure EV Source Data [file 44319_2024_92_MOESM8_ESM.zip › Figure EV2/Figure EV2A/4M.tif]

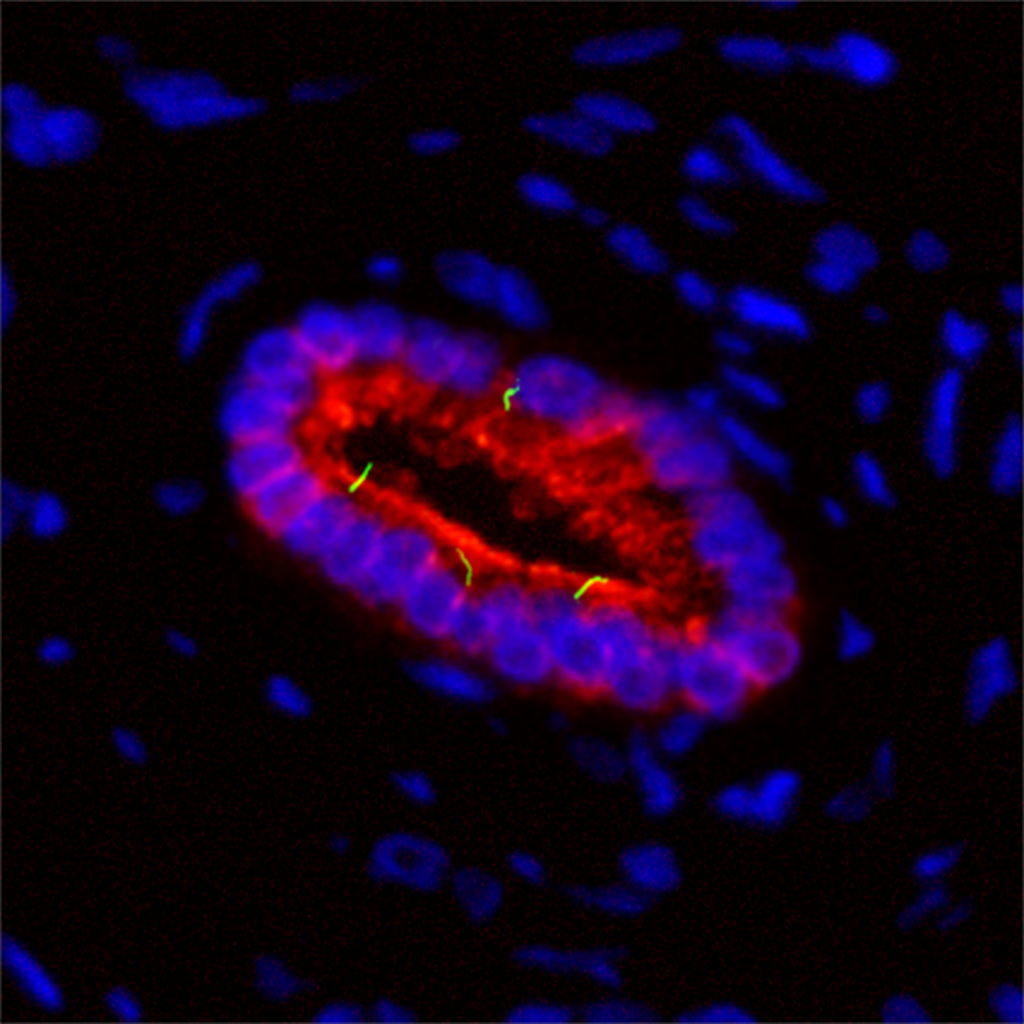

Supplement: Supplementary file 8 — Figure EV Source Data [file 44319_2024_92_MOESM8_ESM.zip › Figure EV2/Figure EV2B/0M .tif]

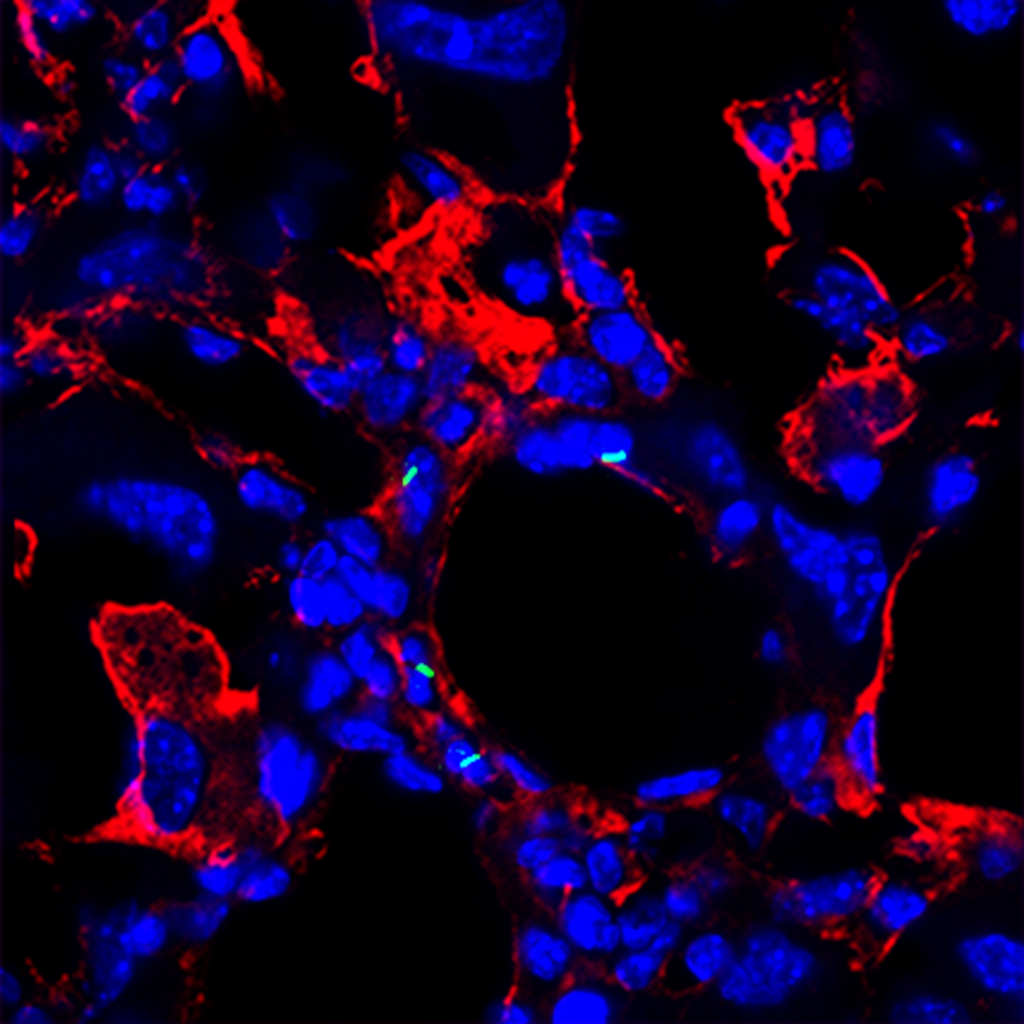

Supplement: Supplementary file 8 — Figure EV Source Data [file 44319_2024_92_MOESM8_ESM.zip › Figure EV2/Figure EV2B/1M.tif]

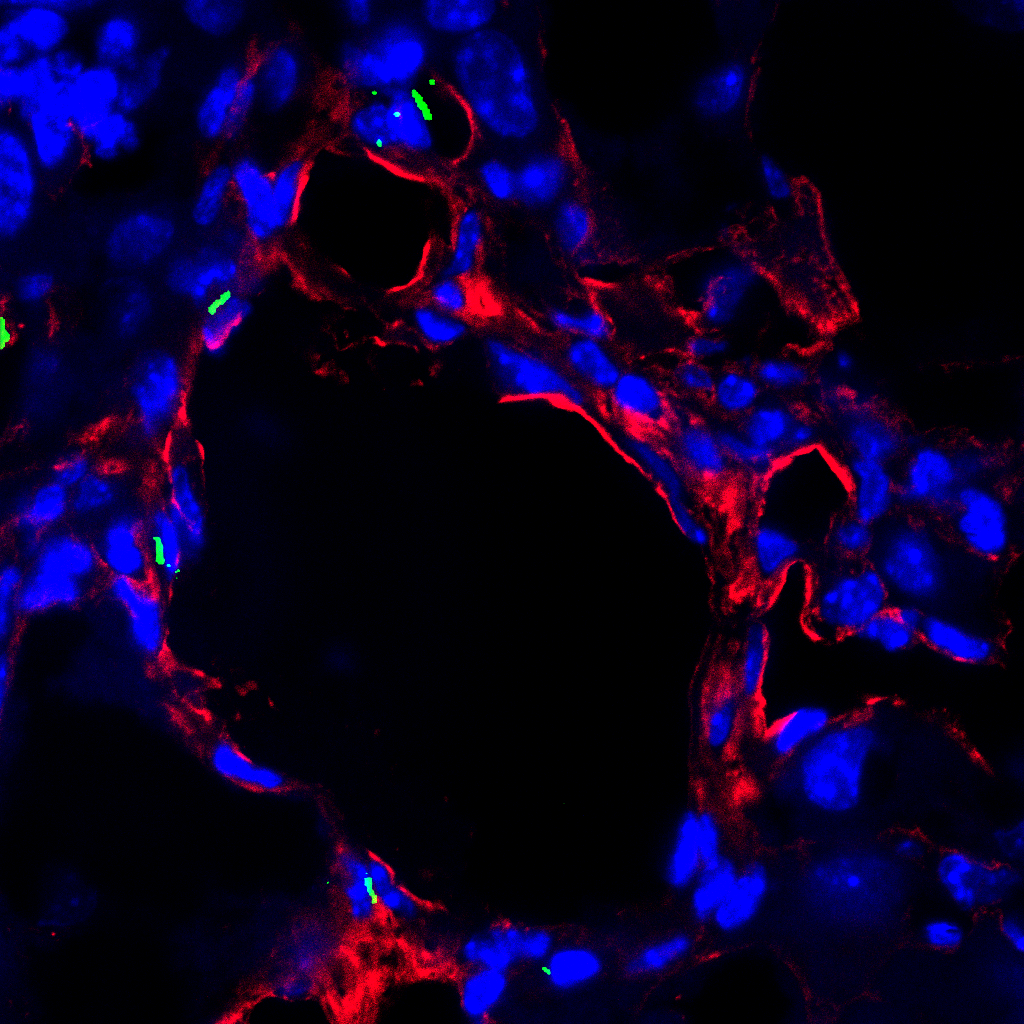

Supplement: Supplementary file 8 — Figure EV Source Data [file 44319_2024_92_MOESM8_ESM.zip › Figure EV2/Figure EV2B/2M.tif]

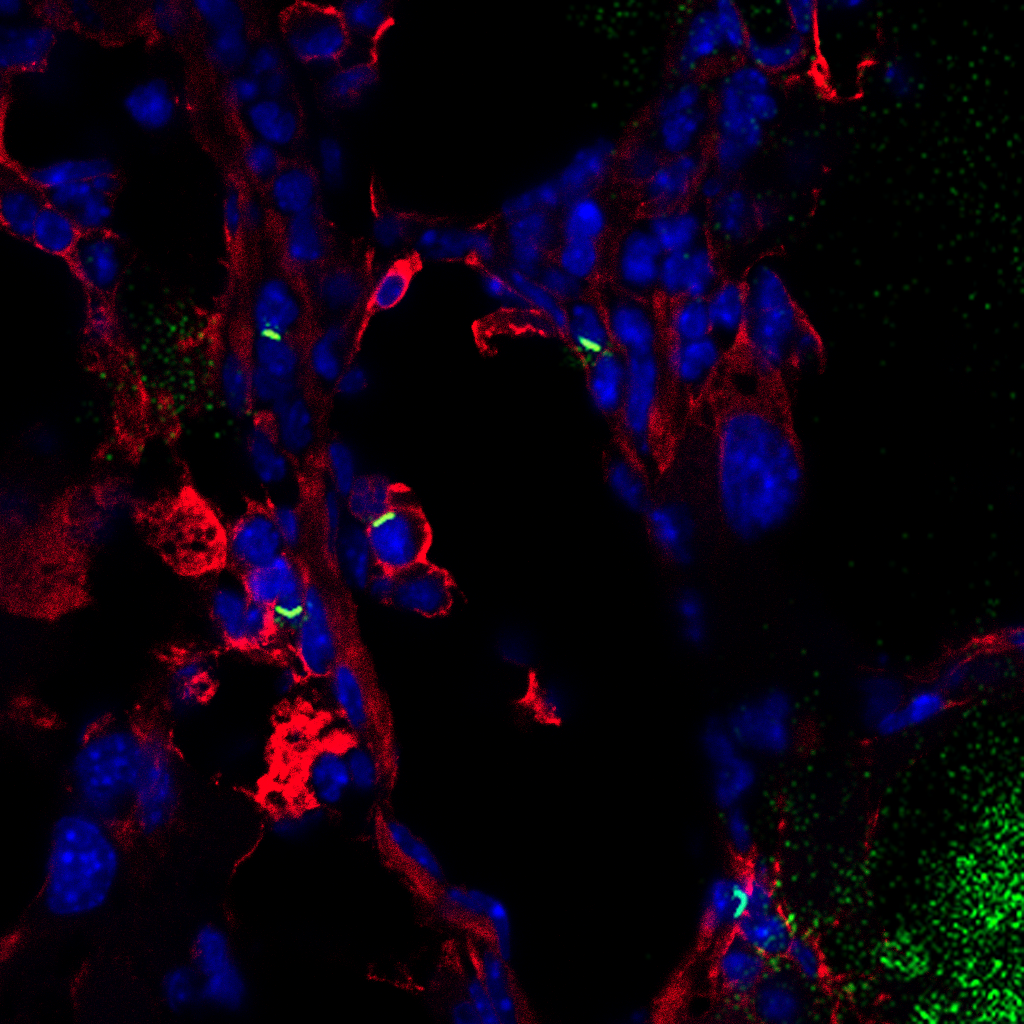

Supplement: Supplementary file 8 — Figure EV Source Data [file 44319_2024_92_MOESM8_ESM.zip › Figure EV2/Figure EV2B/3M.tif]

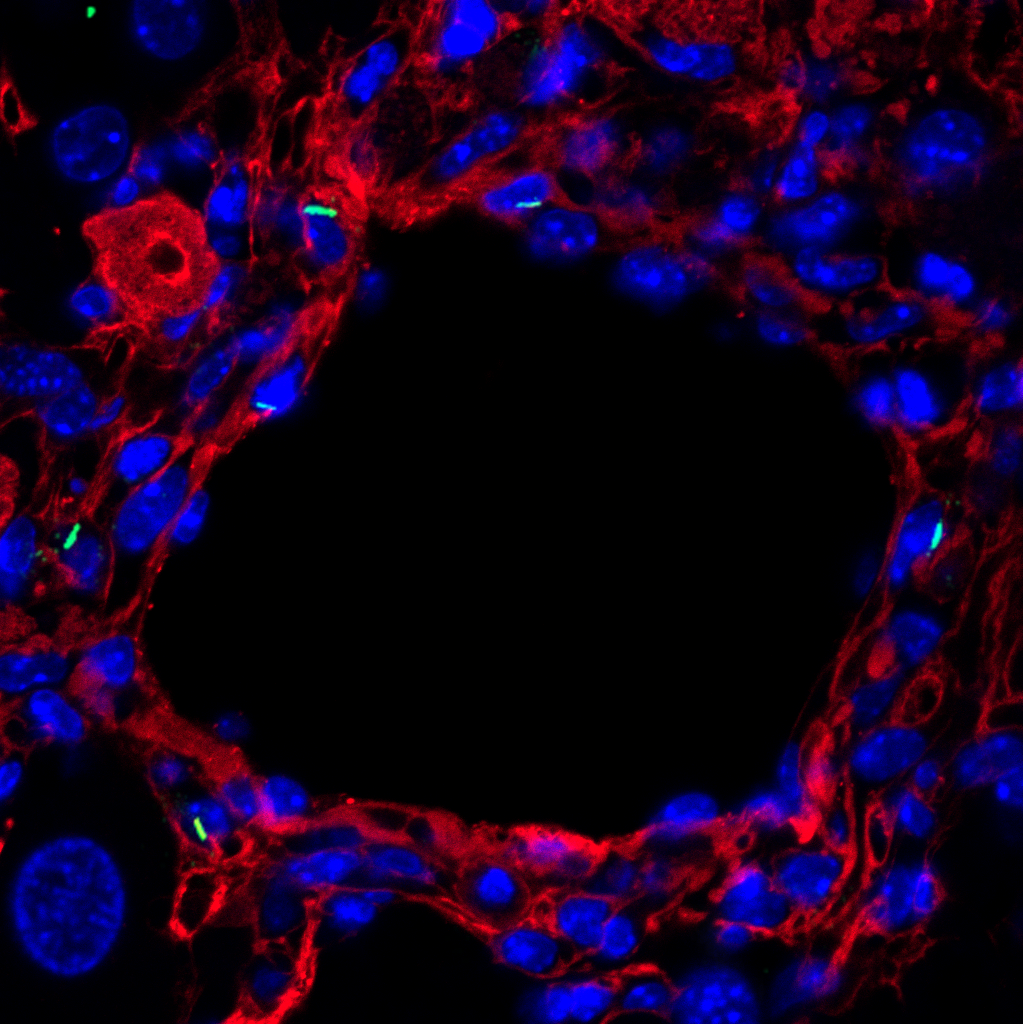

Supplement: Supplementary file 8 — Figure EV Source Data [file 44319_2024_92_MOESM8_ESM.zip › Figure EV2/Figure EV2B/4M.tif]

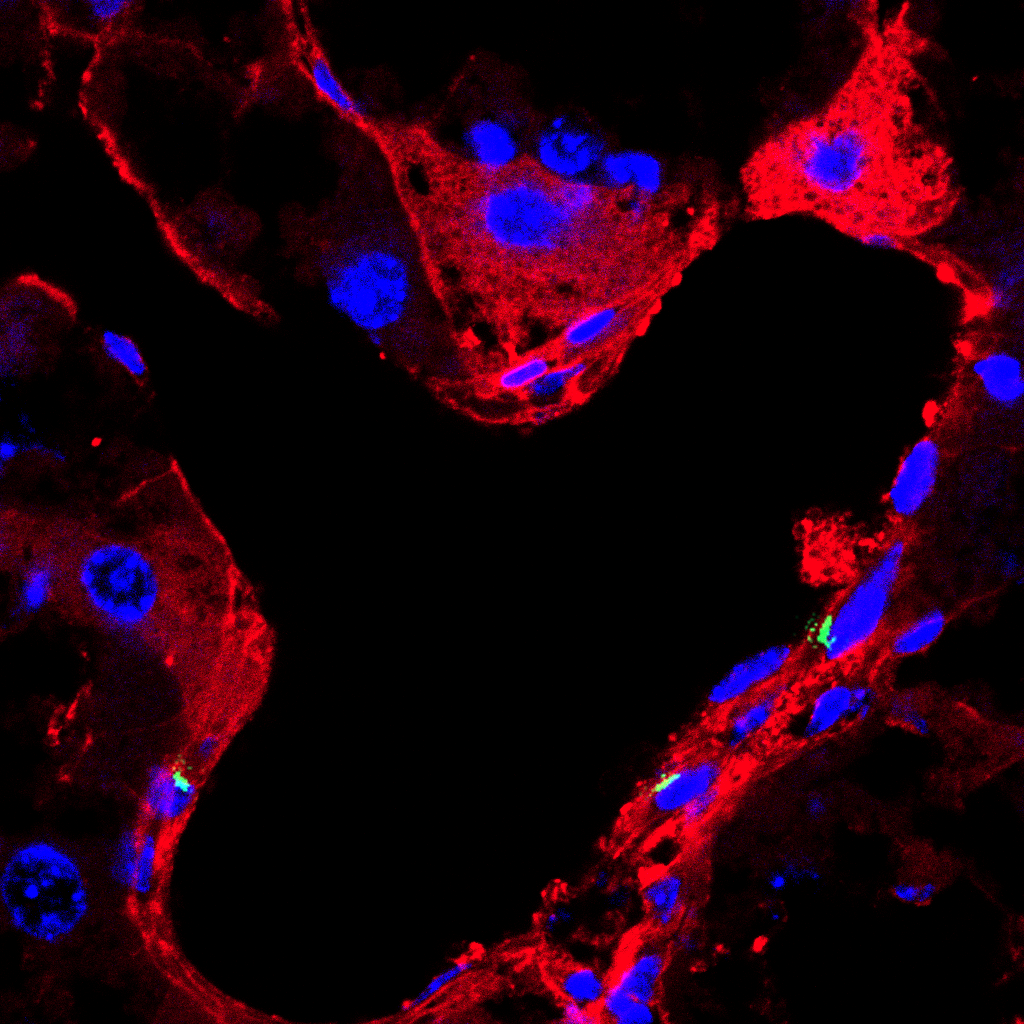

Supplement: Supplementary file 8 — Figure EV Source Data [file 44319_2024_92_MOESM8_ESM.zip › Figure EV2/Figure EV2C/0M.tif]

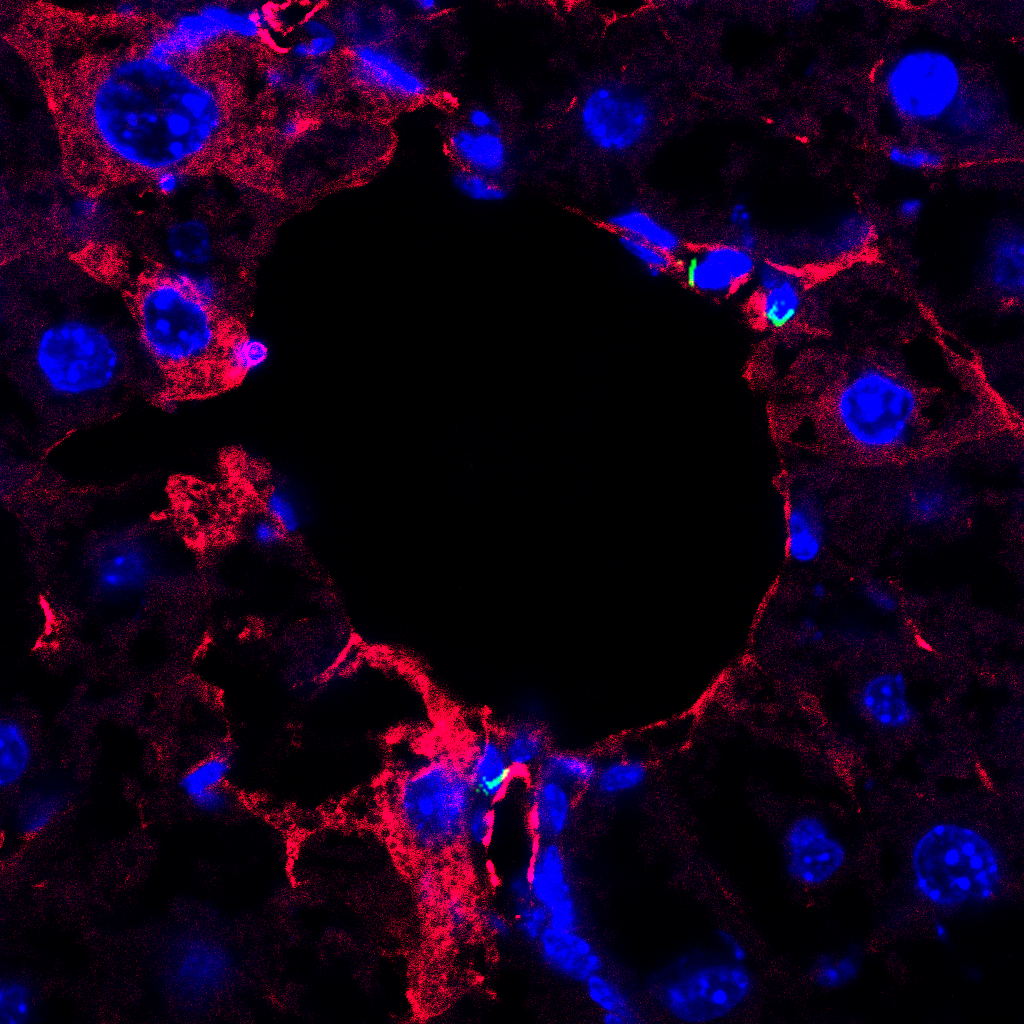

Supplement: Supplementary file 8 — Figure EV Source Data [file 44319_2024_92_MOESM8_ESM.zip › Figure EV2/Figure EV2C/1M.tif]

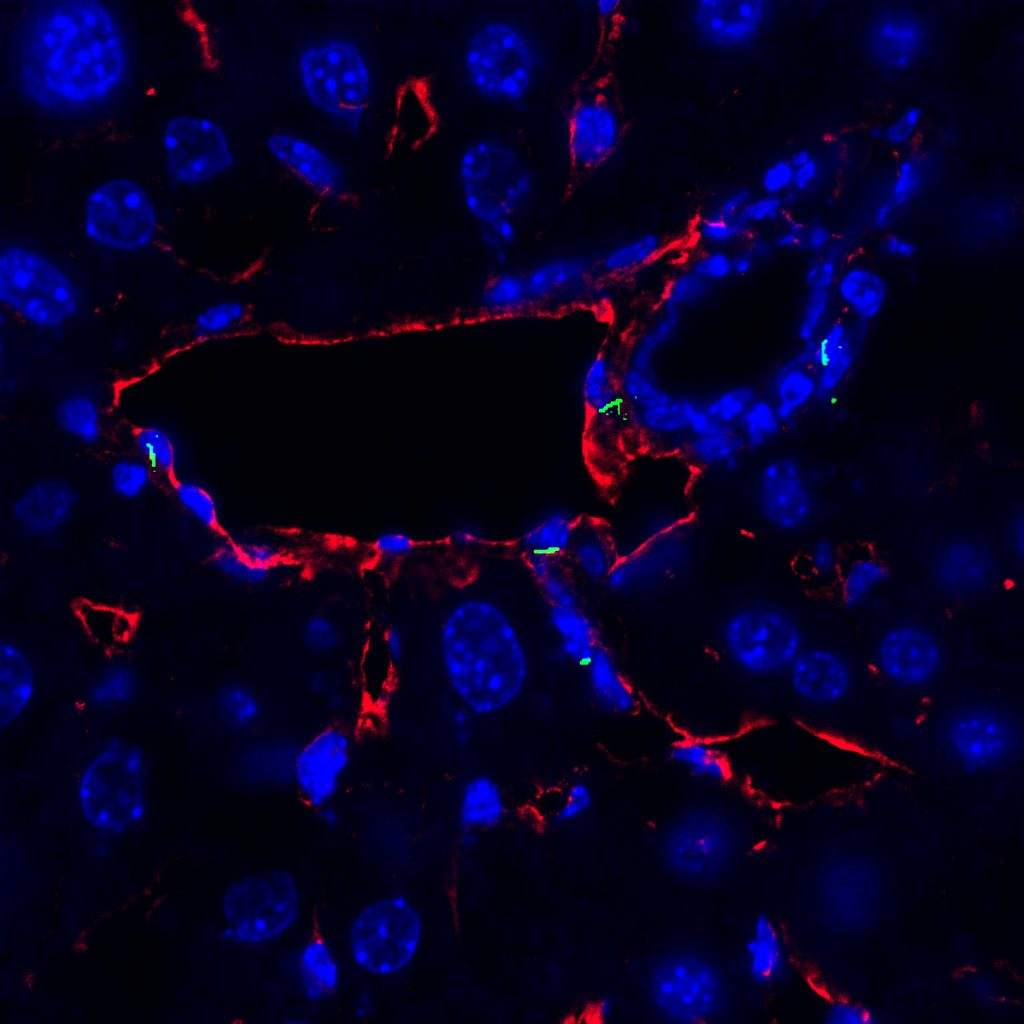

Supplement: Supplementary file 8 — Figure EV Source Data [file 44319_2024_92_MOESM8_ESM.zip › Figure EV2/Figure EV2C/2M.tif]

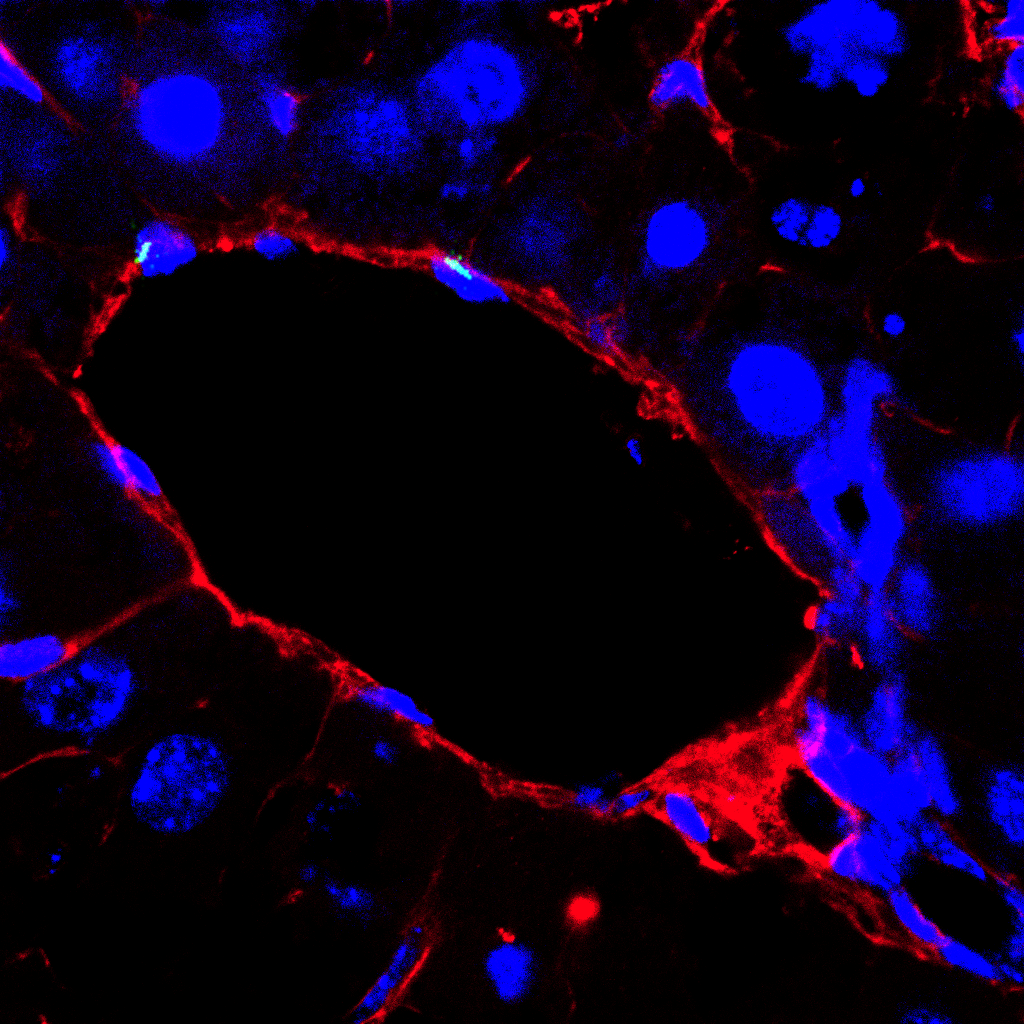

Supplement: Supplementary file 8 — Figure EV Source Data [file 44319_2024_92_MOESM8_ESM.zip › Figure EV2/Figure EV2C/3M.tif]

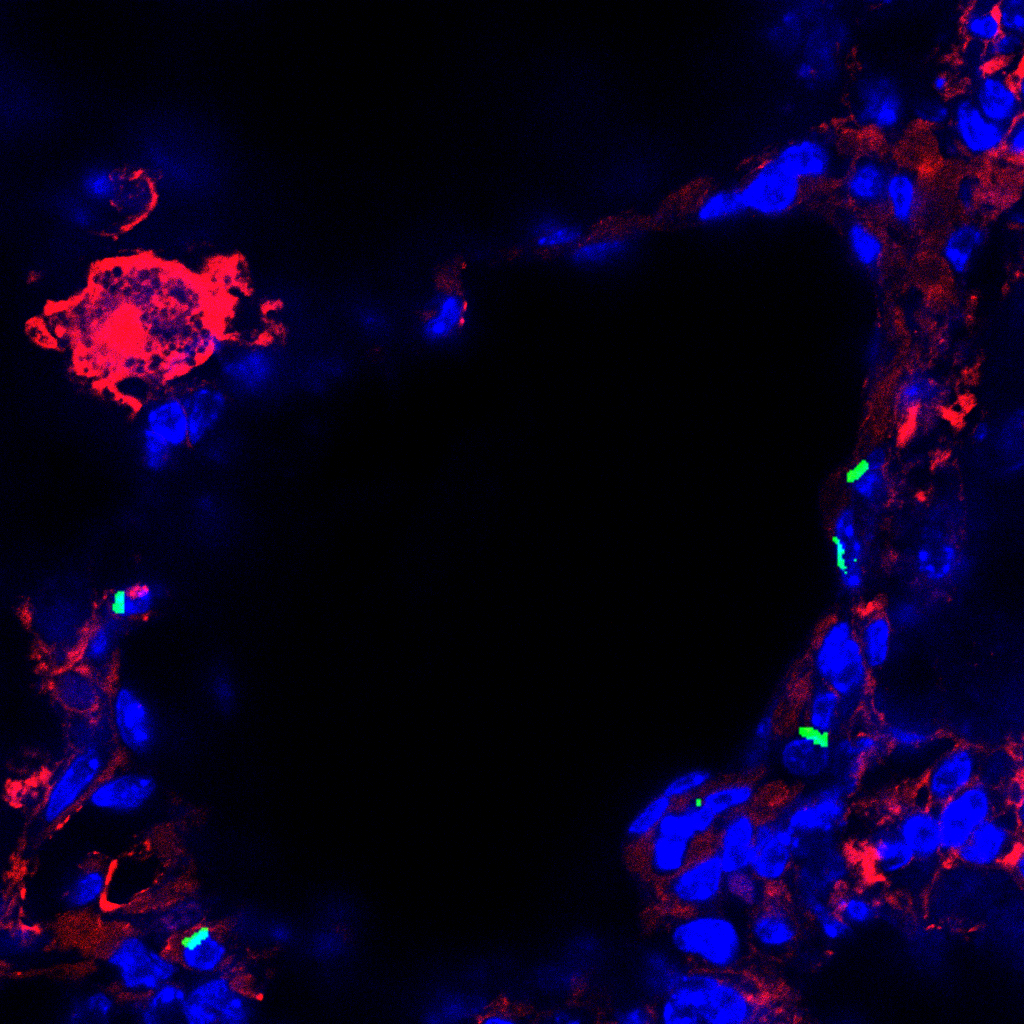

Supplement: Supplementary file 8 — Figure EV Source Data [file 44319_2024_92_MOESM8_ESM.zip › Figure EV2/Figure EV2C/4M.tif]

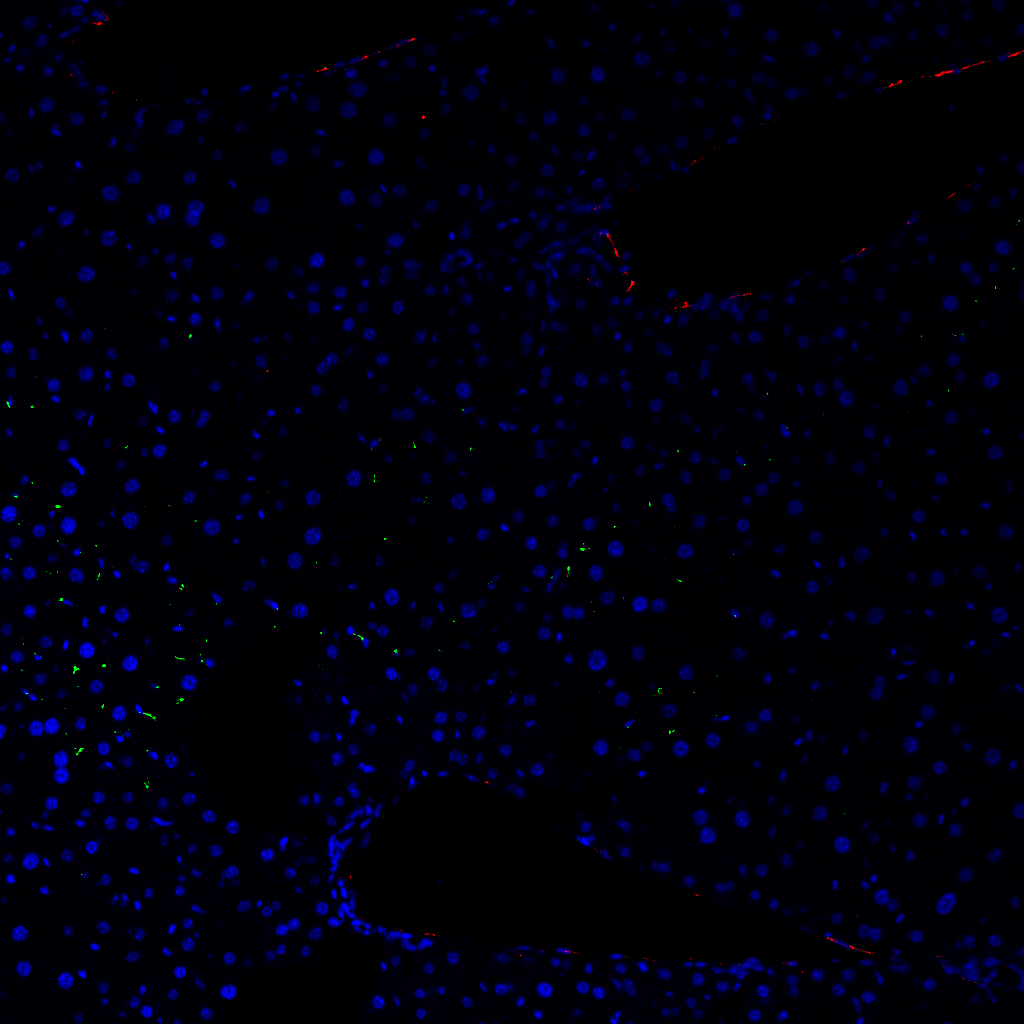

Supplement: Supplementary file 8 — Figure EV Source Data [file 44319_2024_92_MOESM8_ESM.zip › Figure EV3/Figure EV3A/1.tif]
