# Supplementary figures and images for: XIAP-mediated degradation of IFT88 disrupts HSC cilia to stimulate HSC activation and liver fibrosis (part 2 of 2)
Source: EMBO Rep. 2024 Feb 13;25(3):12. doi: 10.1038/s44319-024-00092-y (PMC10933415; doi:10.1038/s44319-024-00092-y)

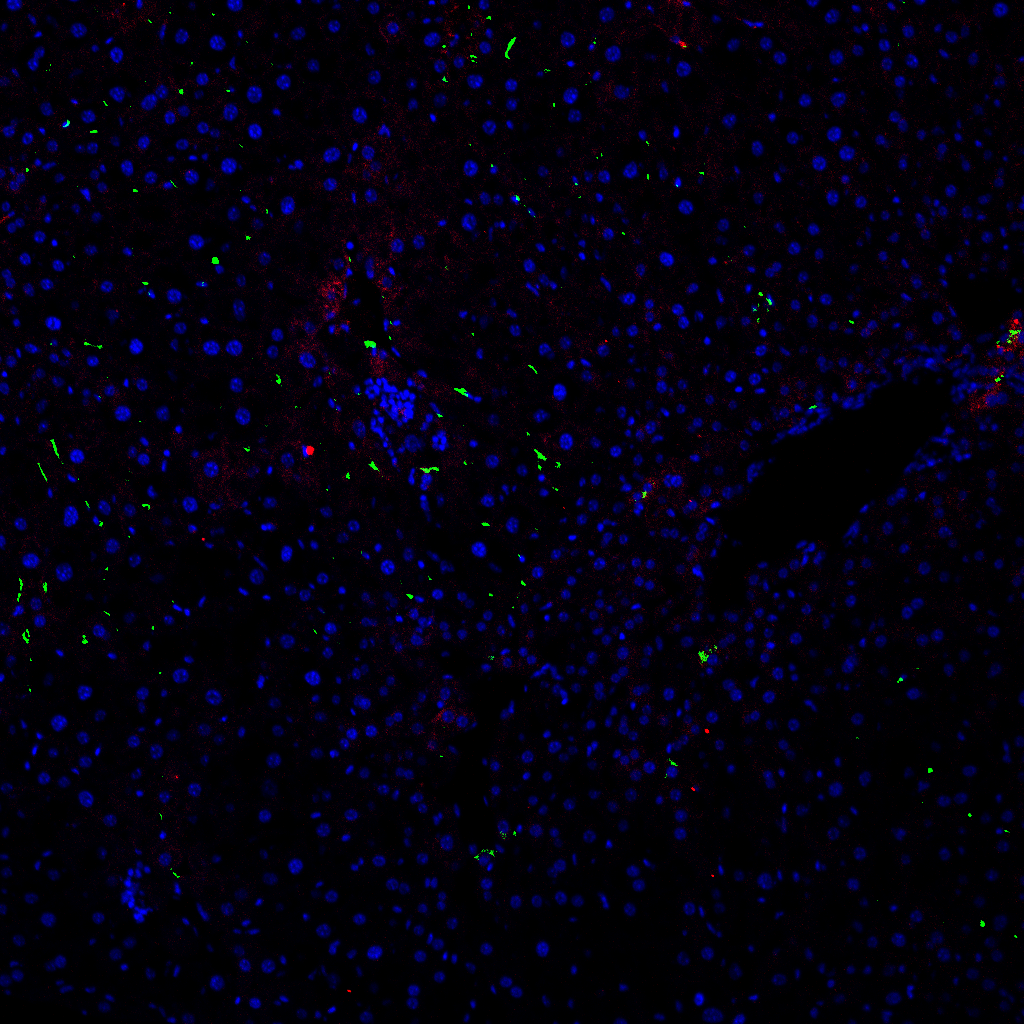

Supplement: Supplementary file 8 — Figure EV Source Data [file 44319_2024_92_MOESM8_ESM.zip › Figure EV3/Figure EV3A/2.tif]

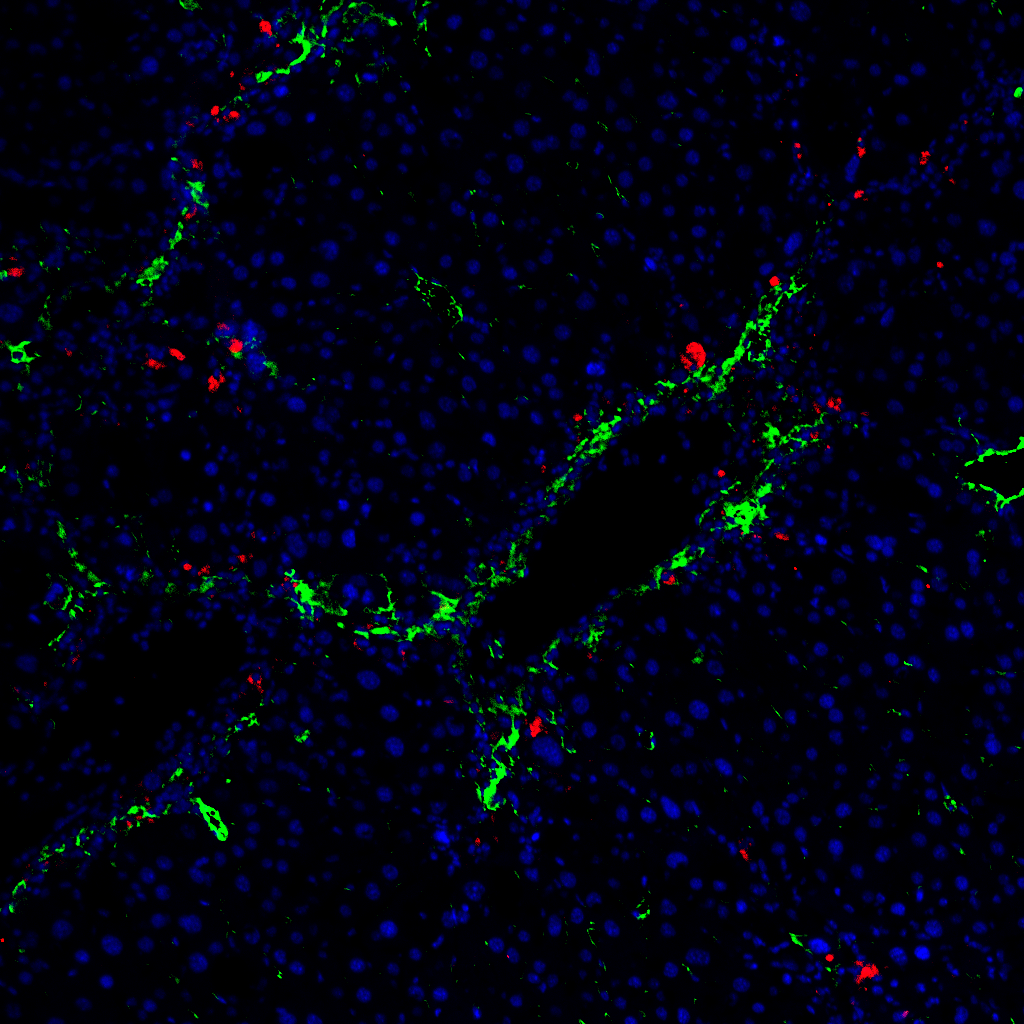

Supplement: Supplementary file 8 — Figure EV Source Data [file 44319_2024_92_MOESM8_ESM.zip › Figure EV3/Figure EV3A/3.tif]

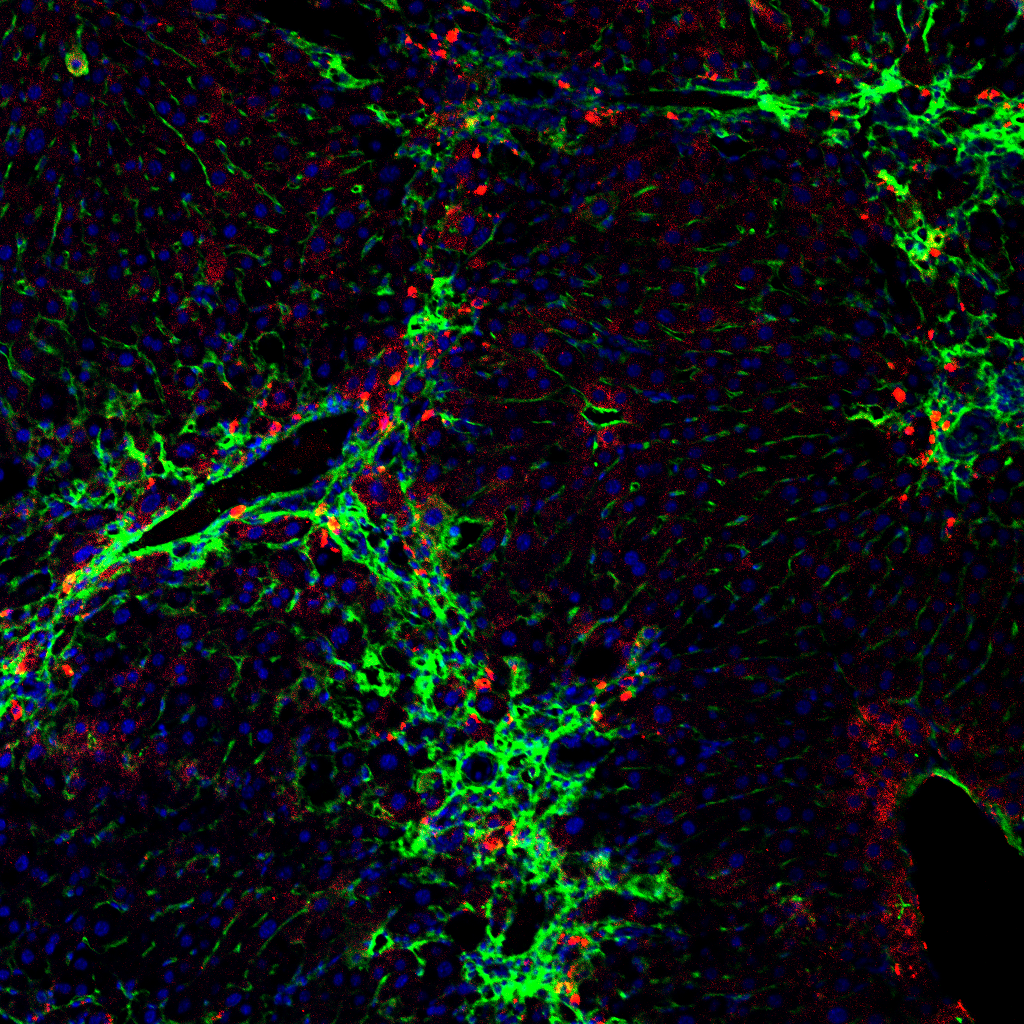

Supplement: Supplementary file 8 — Figure EV Source Data [file 44319_2024_92_MOESM8_ESM.zip › Figure EV3/Figure EV3A/4.tif]

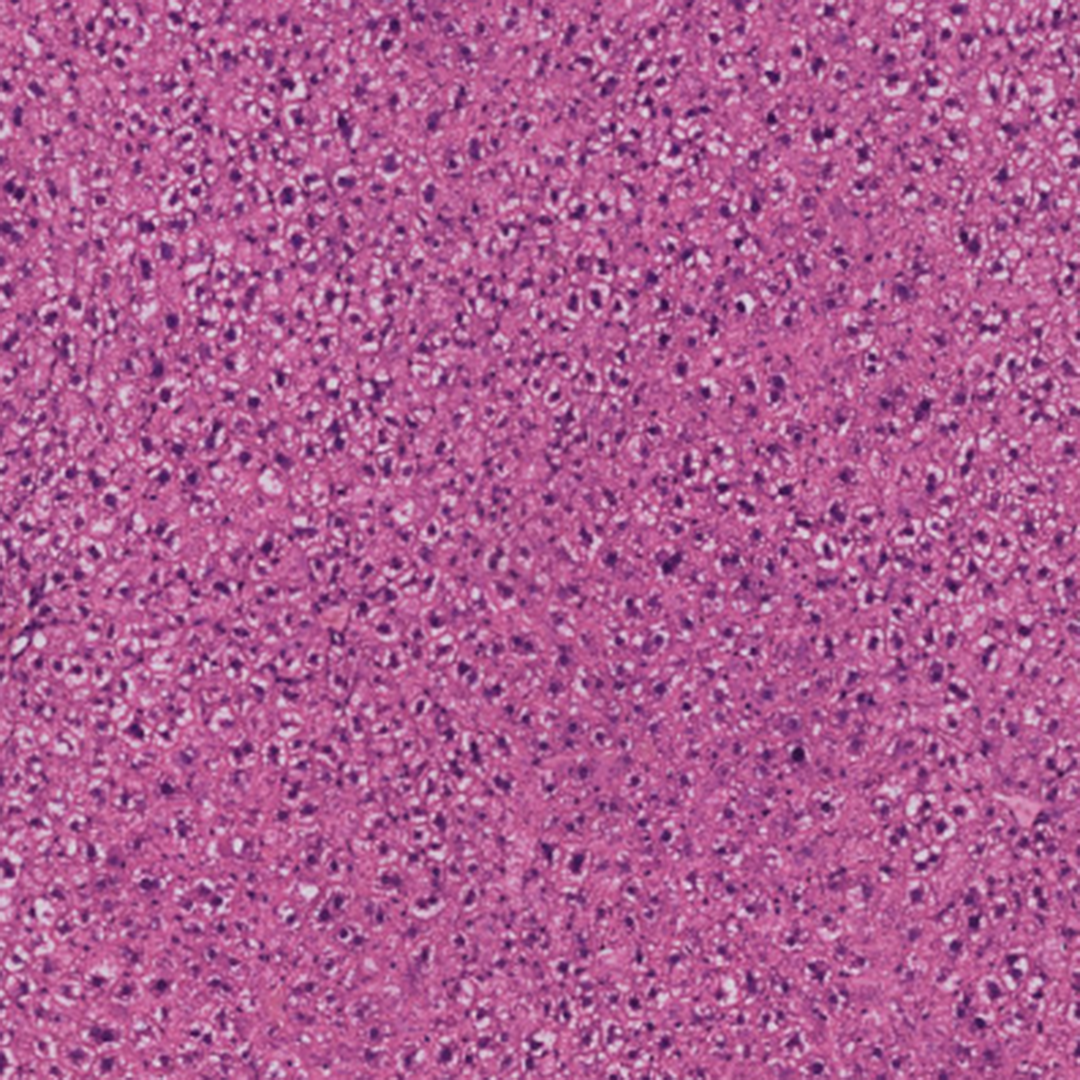

Supplement: Supplementary file 8 — Figure EV Source Data [file 44319_2024_92_MOESM8_ESM.zip › Figure EV3/Figure EV3D/HE/1.tif]

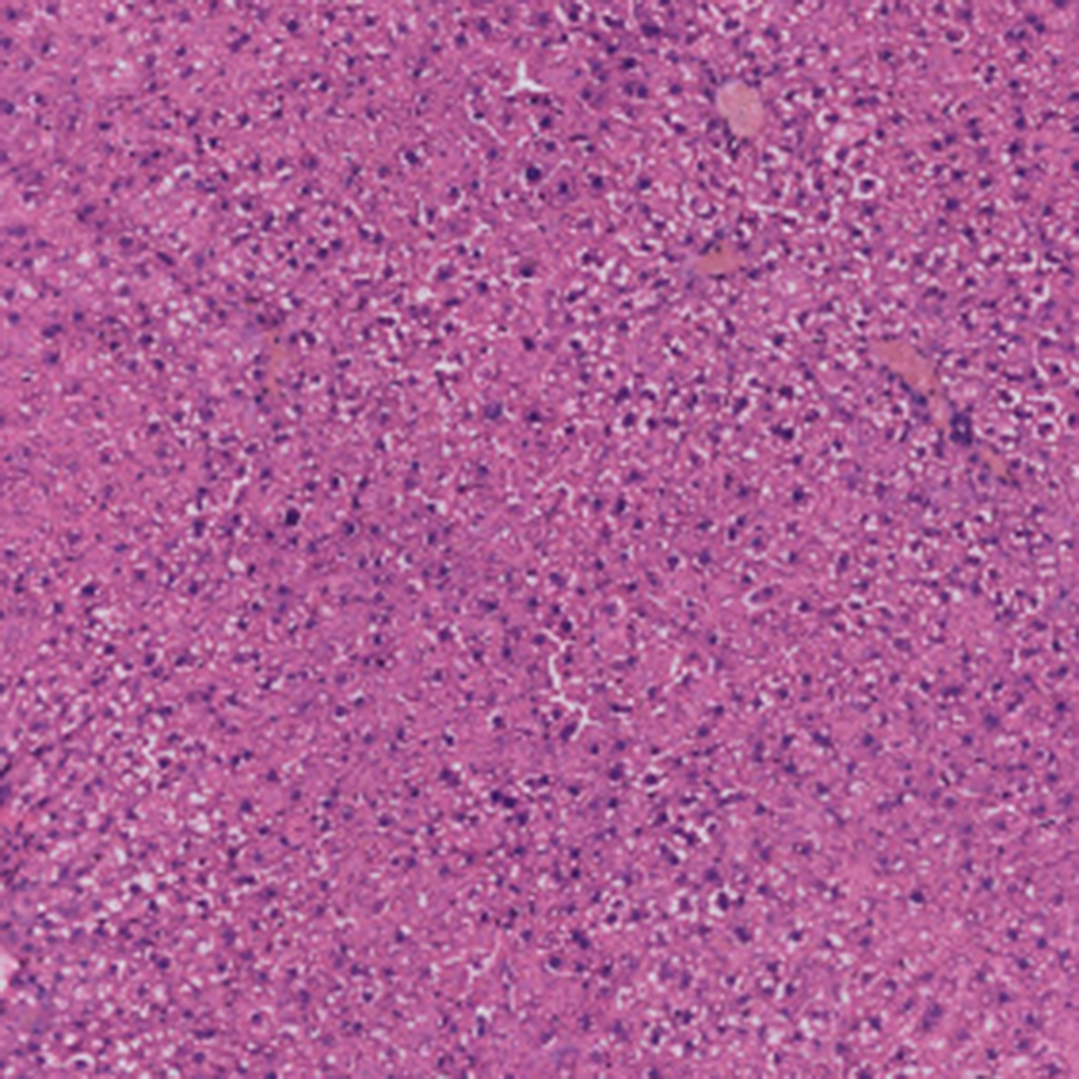

Supplement: Supplementary file 8 — Figure EV Source Data [file 44319_2024_92_MOESM8_ESM.zip › Figure EV3/Figure EV3D/HE/2.tif]

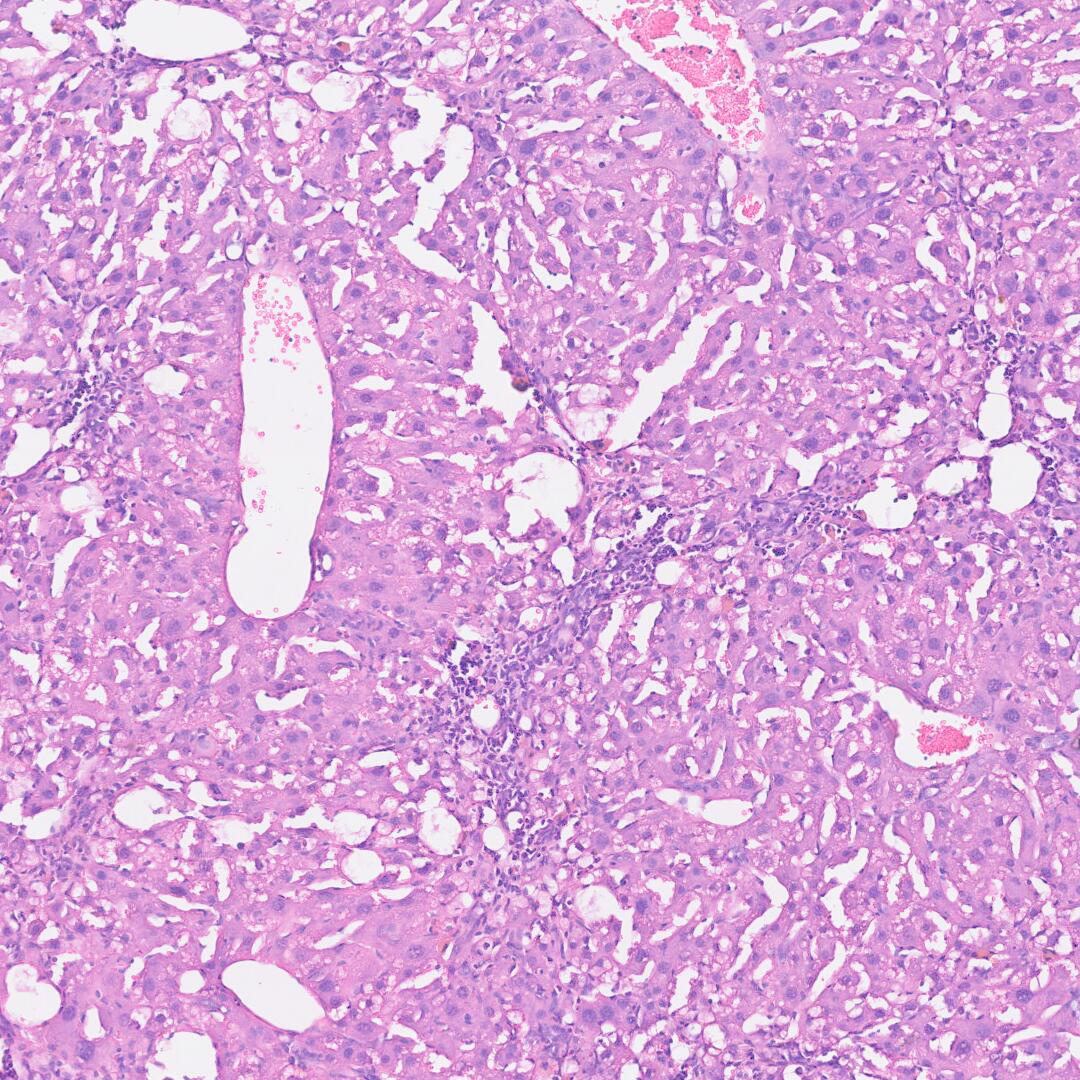

Supplement: Supplementary file 8 — Figure EV Source Data [file 44319_2024_92_MOESM8_ESM.zip › Figure EV3/Figure EV3D/HE/3.tif]

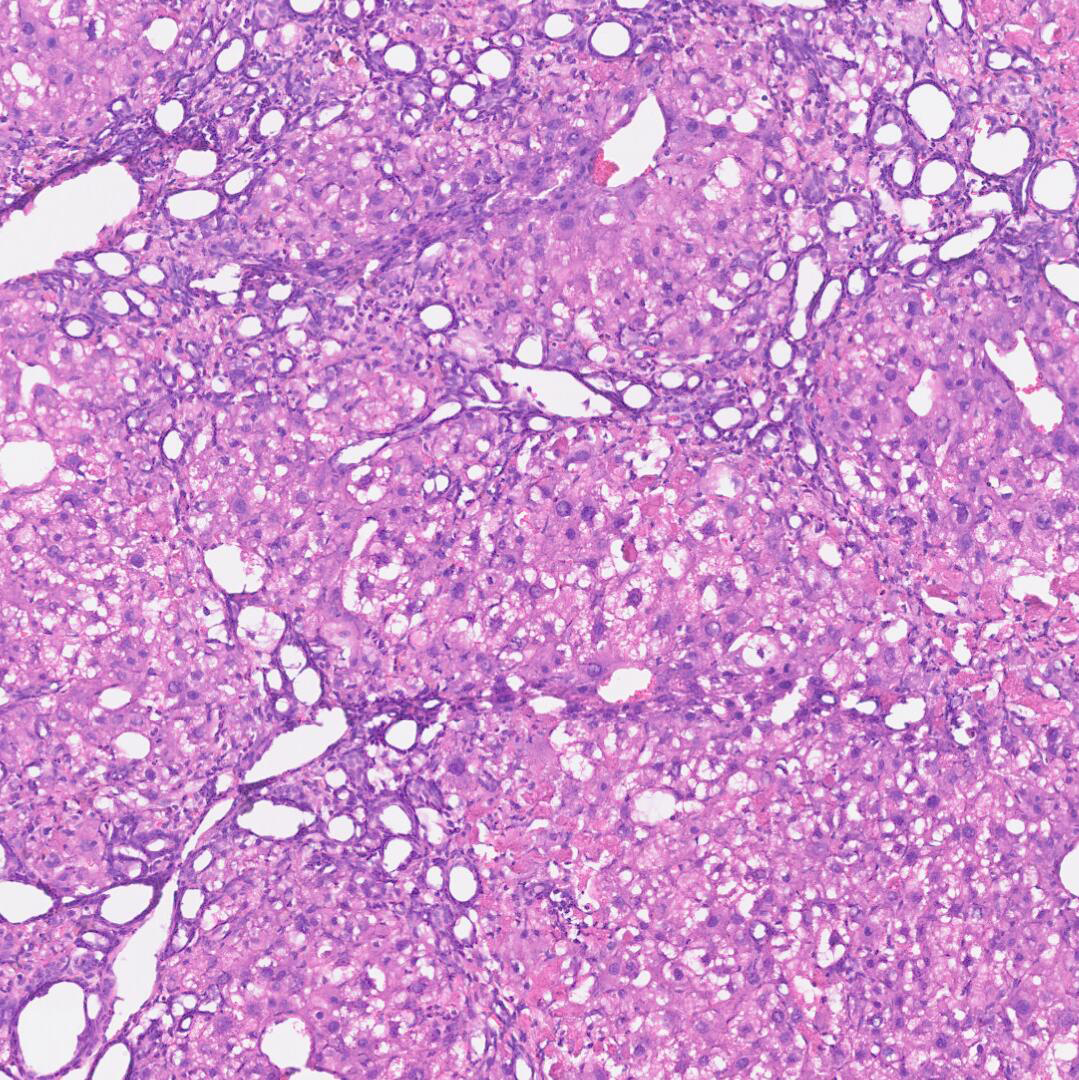

Supplement: Supplementary file 8 — Figure EV Source Data [file 44319_2024_92_MOESM8_ESM.zip › Figure EV3/Figure EV3D/HE/4.tif]

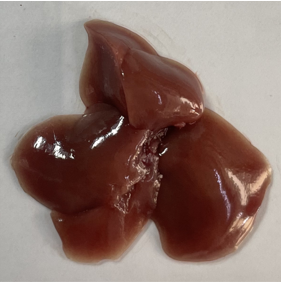

Supplement: Supplementary file 8 — Figure EV Source Data [file 44319_2024_92_MOESM8_ESM.zip › Figure EV3/Figure EV3D/liver/1.tif]

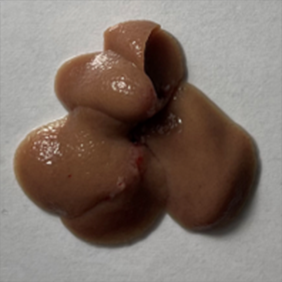

Supplement: Supplementary file 8 — Figure EV Source Data [file 44319_2024_92_MOESM8_ESM.zip › Figure EV3/Figure EV3D/liver/2.tif]

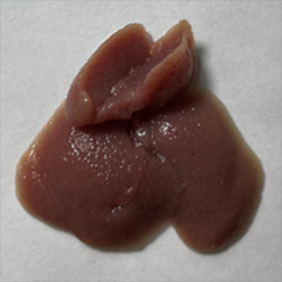

Supplement: Supplementary file 8 — Figure EV Source Data [file 44319_2024_92_MOESM8_ESM.zip › Figure EV3/Figure EV3D/liver/3.tif]

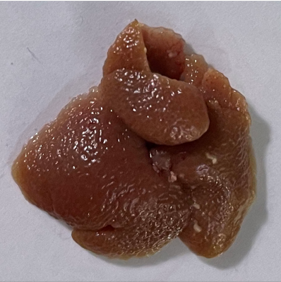

Supplement: Supplementary file 8 — Figure EV Source Data [file 44319_2024_92_MOESM8_ESM.zip › Figure EV3/Figure EV3D/liver/4.tif]

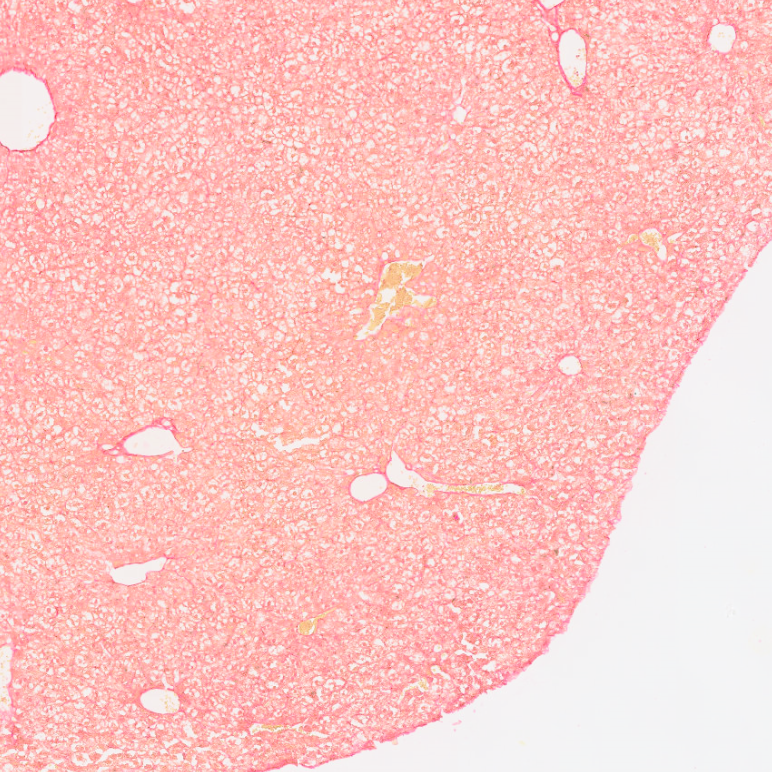

Supplement: Supplementary file 8 — Figure EV Source Data [file 44319_2024_92_MOESM8_ESM.zip › Figure EV3/Figure EV3D/Sirius red/Sirius red 1 .tif]

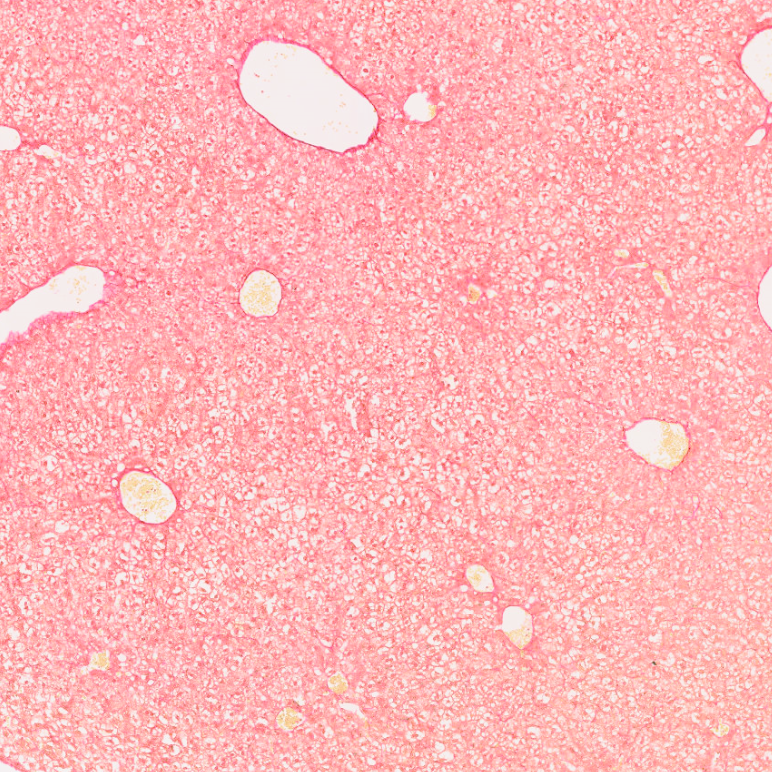

Supplement: Supplementary file 8 — Figure EV Source Data [file 44319_2024_92_MOESM8_ESM.zip › Figure EV3/Figure EV3D/Sirius red/Sirius red 2 .tif]

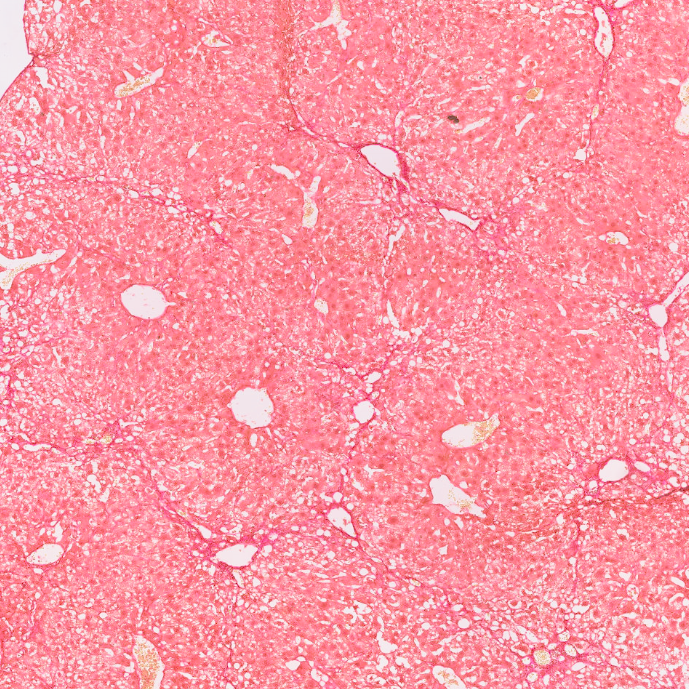

Supplement: Supplementary file 8 — Figure EV Source Data [file 44319_2024_92_MOESM8_ESM.zip › Figure EV3/Figure EV3D/Sirius red/Sirius red 3 .tif]

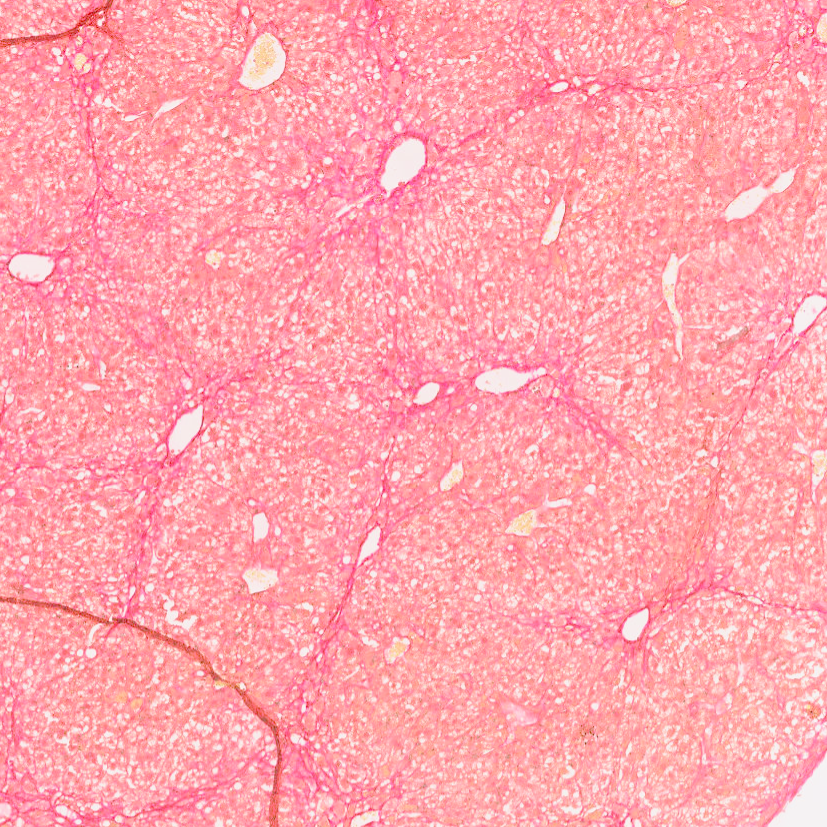

Supplement: Supplementary file 8 — Figure EV Source Data [file 44319_2024_92_MOESM8_ESM.zip › Figure EV3/Figure EV3D/Sirius red/Sirius red 4 .tif]

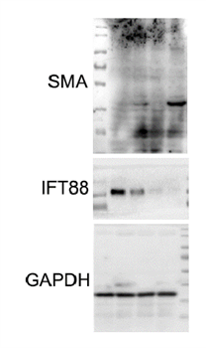

Supplement: Supplementary file 8 — Figure EV Source Data [file 44319_2024_92_MOESM8_ESM.zip › Figure EV3/Figure EV3M/Figure 3M.tif]

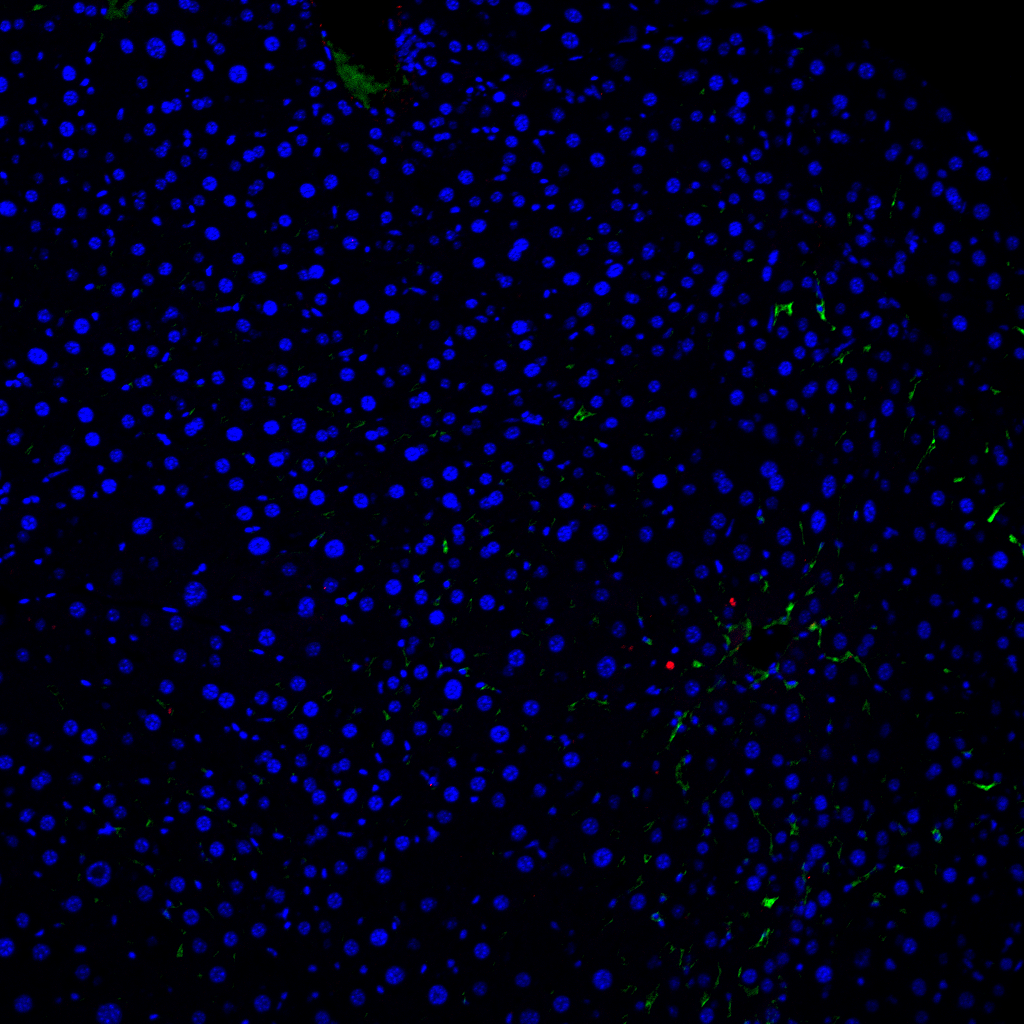

Supplement: Supplementary file 8 — Figure EV Source Data [file 44319_2024_92_MOESM8_ESM.zip › Figure EV4/Figure EV4A/1.tif]

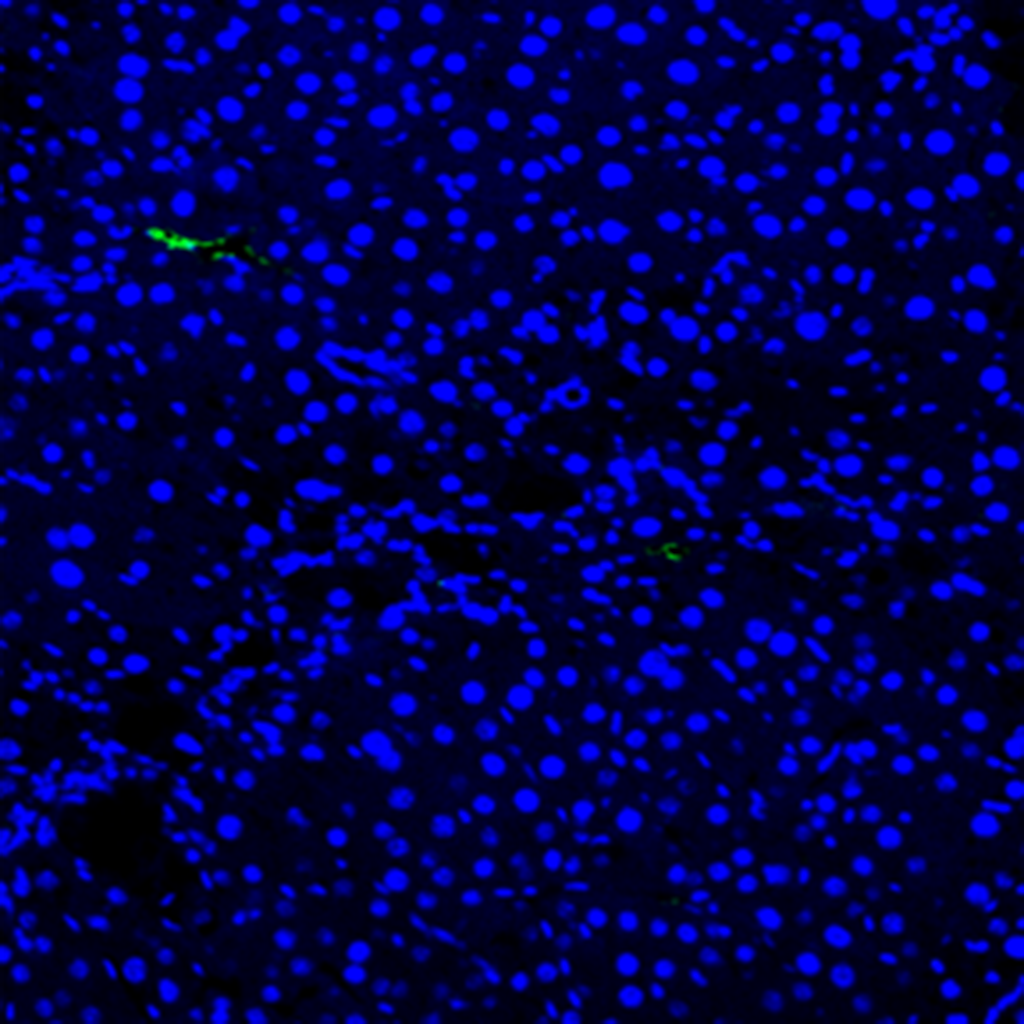

Supplement: Supplementary file 8 — Figure EV Source Data [file 44319_2024_92_MOESM8_ESM.zip › Figure EV4/Figure EV4A/2.tif]

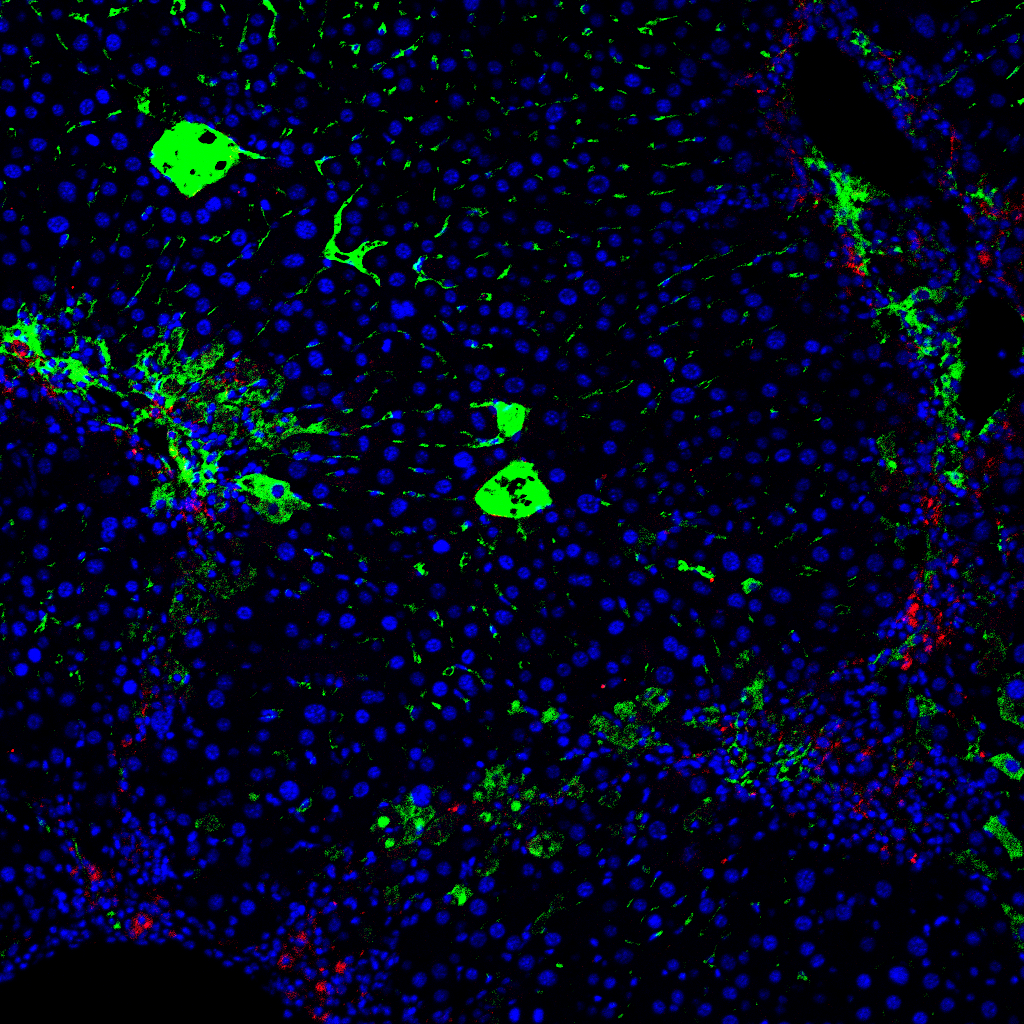

Supplement: Supplementary file 8 — Figure EV Source Data [file 44319_2024_92_MOESM8_ESM.zip › Figure EV4/Figure EV4A/3.tif]

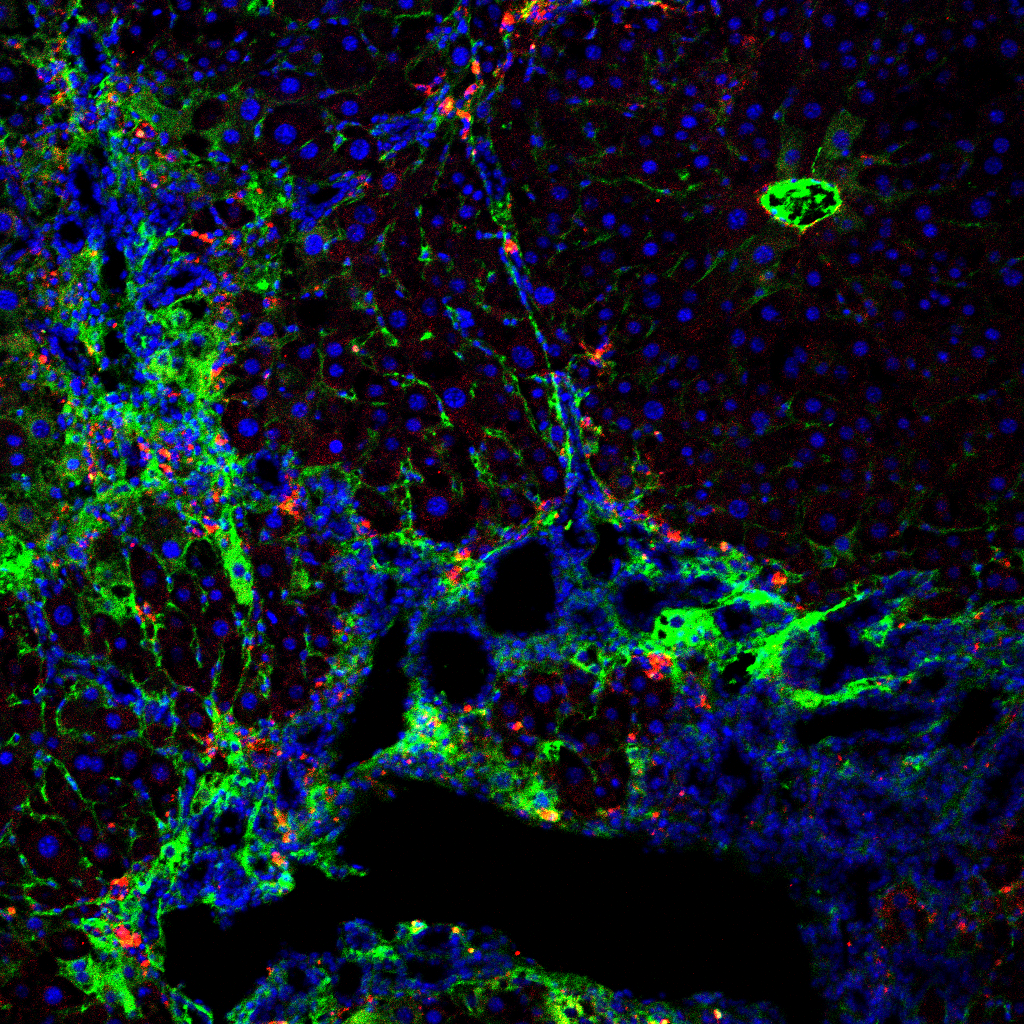

Supplement: Supplementary file 8 — Figure EV Source Data [file 44319_2024_92_MOESM8_ESM.zip › Figure EV4/Figure EV4A/4.tif]

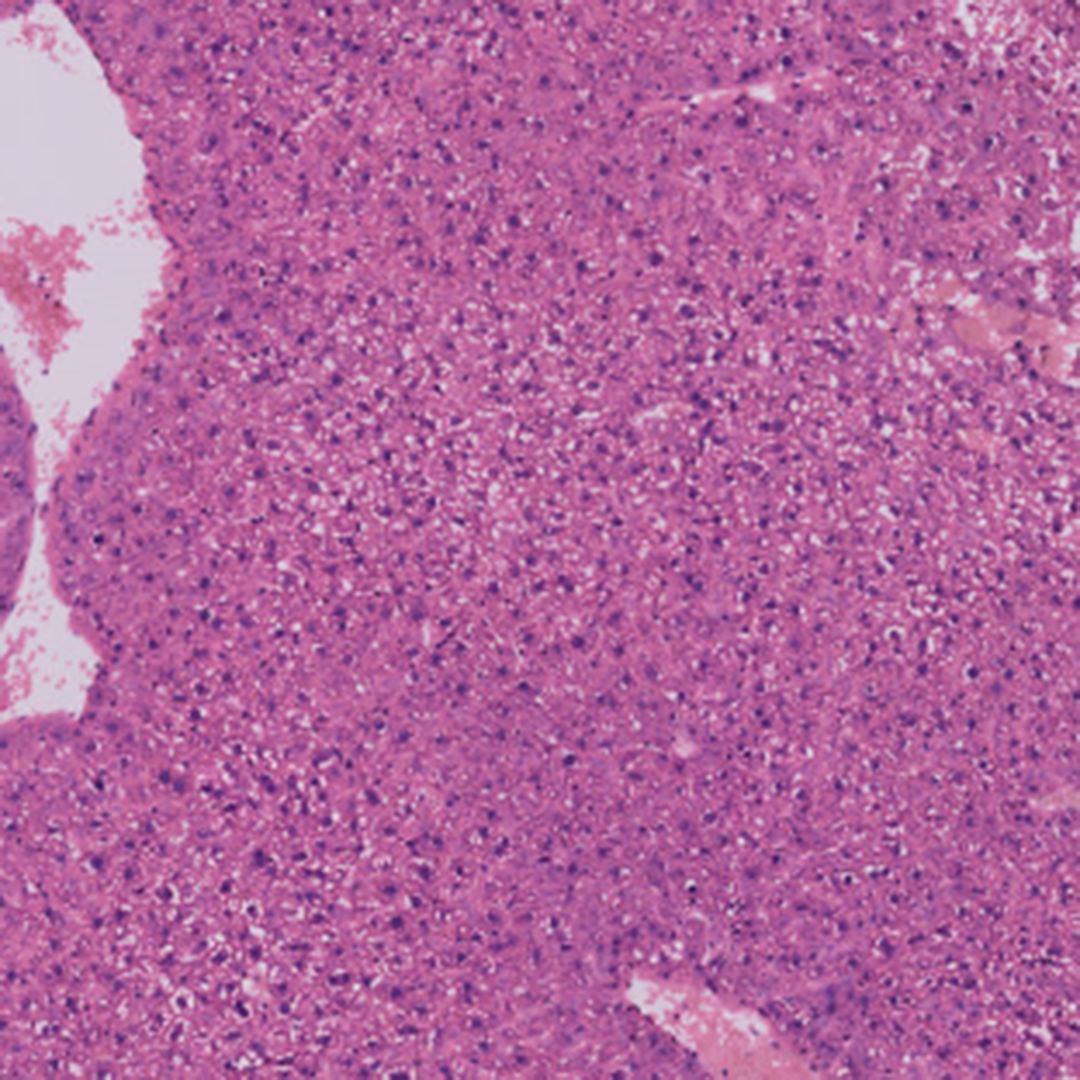

Supplement: Supplementary file 8 — Figure EV Source Data [file 44319_2024_92_MOESM8_ESM.zip › Figure EV4/Figure EV4D/HE/1.tif]

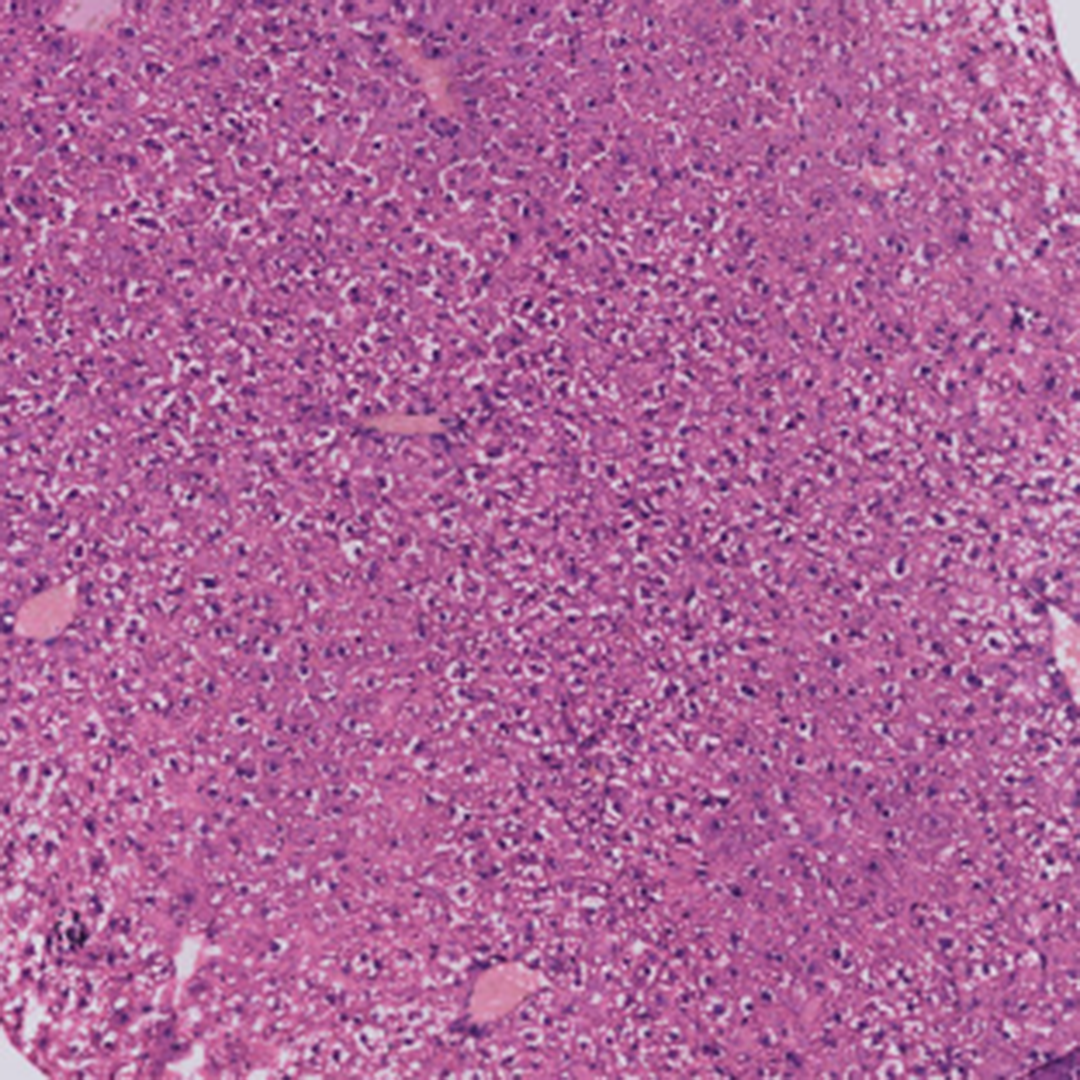

Supplement: Supplementary file 8 — Figure EV Source Data [file 44319_2024_92_MOESM8_ESM.zip › Figure EV4/Figure EV4D/HE/2.tif]

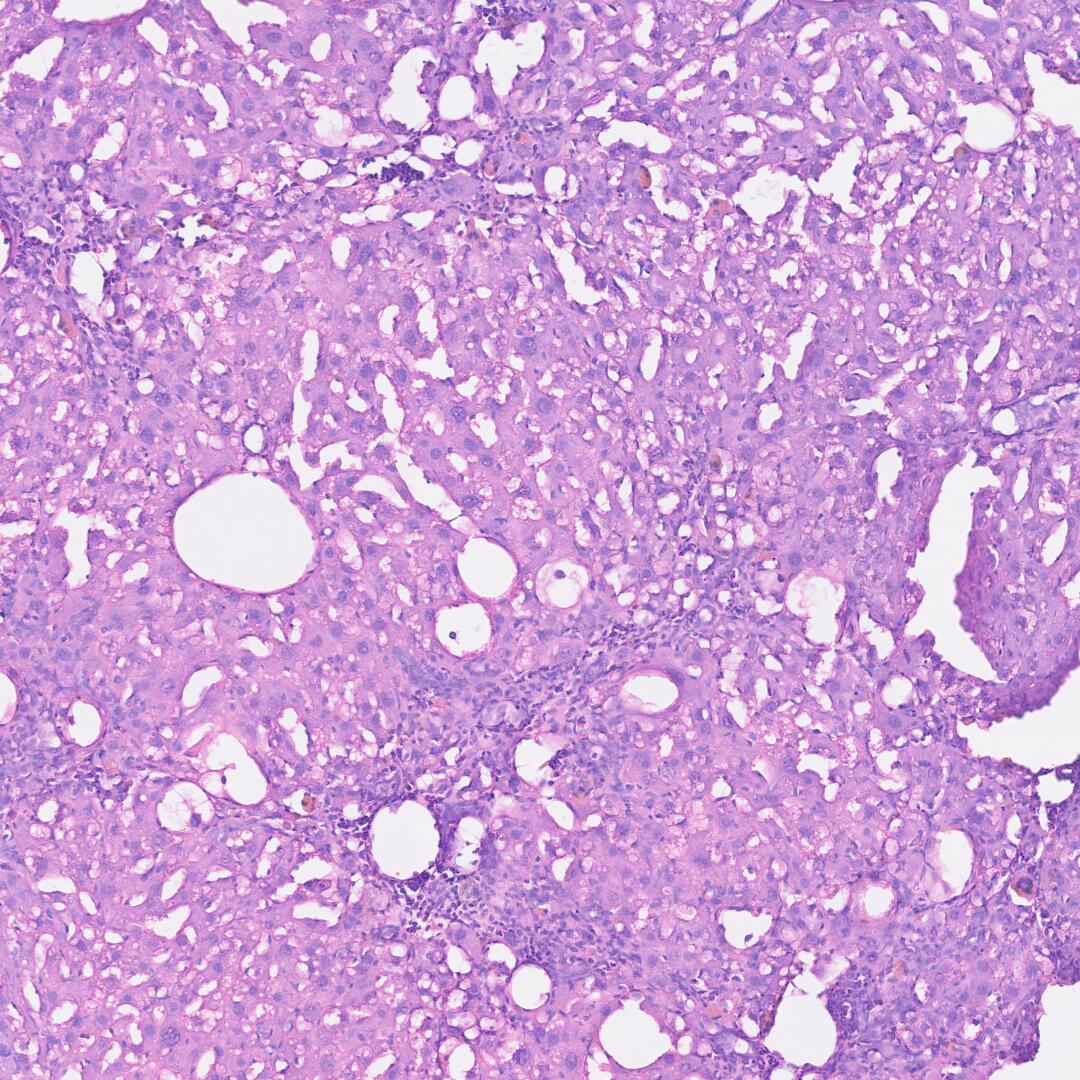

Supplement: Supplementary file 8 — Figure EV Source Data [file 44319_2024_92_MOESM8_ESM.zip › Figure EV4/Figure EV4D/HE/3.tif]

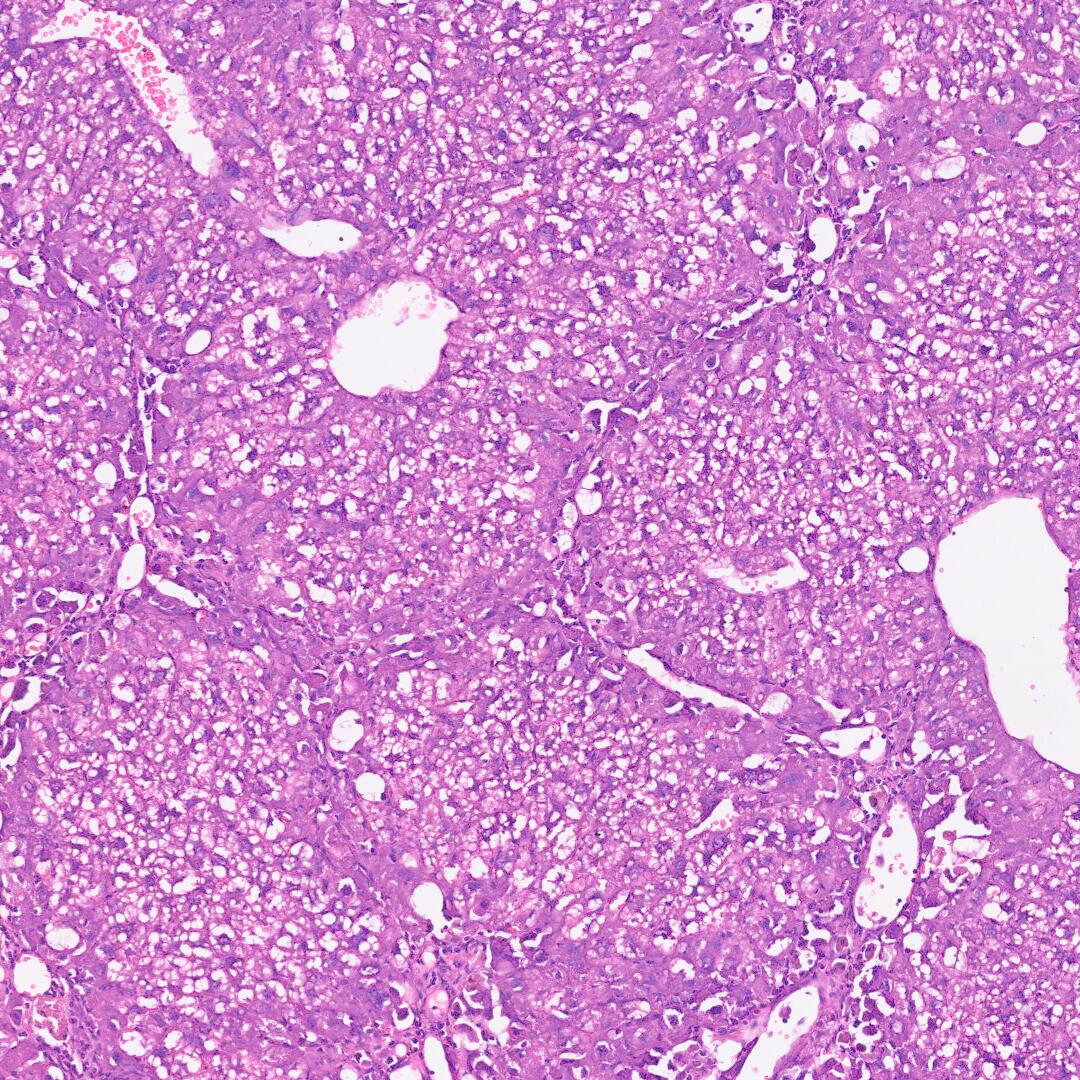

Supplement: Supplementary file 8 — Figure EV Source Data [file 44319_2024_92_MOESM8_ESM.zip › Figure EV4/Figure EV4D/HE/4.tif]

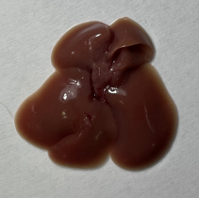

Supplement: Supplementary file 8 — Figure EV Source Data [file 44319_2024_92_MOESM8_ESM.zip › Figure EV4/Figure EV4D/liver/1.tif]

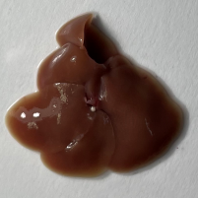

Supplement: Supplementary file 8 — Figure EV Source Data [file 44319_2024_92_MOESM8_ESM.zip › Figure EV4/Figure EV4D/liver/2.tif]

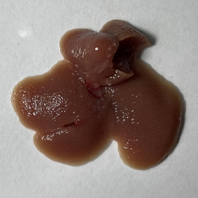

Supplement: Supplementary file 8 — Figure EV Source Data [file 44319_2024_92_MOESM8_ESM.zip › Figure EV4/Figure EV4D/liver/3.tif]

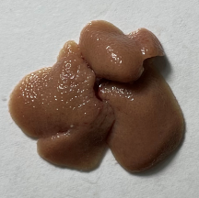

Supplement: Supplementary file 8 — Figure EV Source Data [file 44319_2024_92_MOESM8_ESM.zip › Figure EV4/Figure EV4D/liver/4.tif]

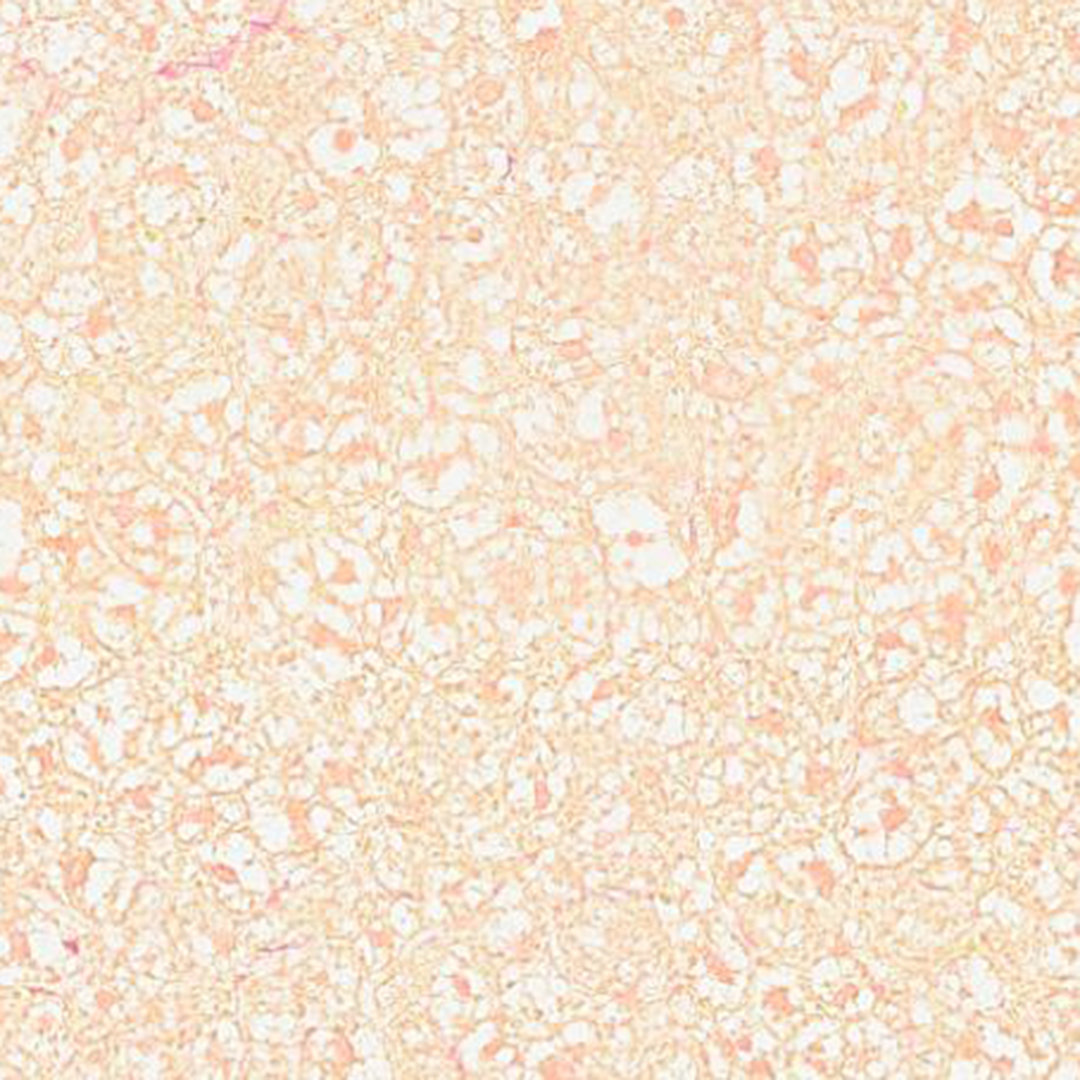

Supplement: Supplementary file 8 — Figure EV Source Data [file 44319_2024_92_MOESM8_ESM.zip › Figure EV4/Figure EV4D/Sirius red/1.tif]

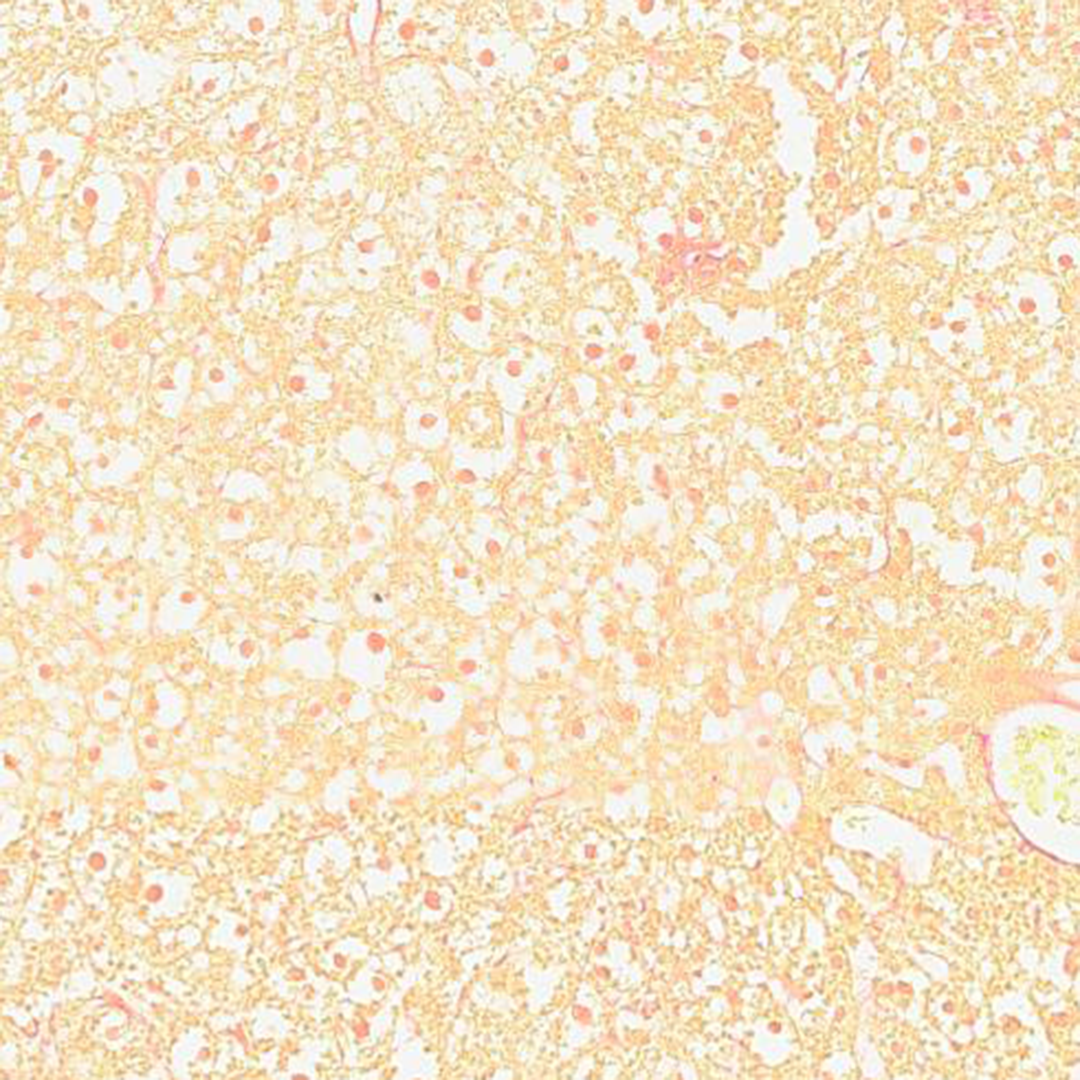

Supplement: Supplementary file 8 — Figure EV Source Data [file 44319_2024_92_MOESM8_ESM.zip › Figure EV4/Figure EV4D/Sirius red/2.tif]

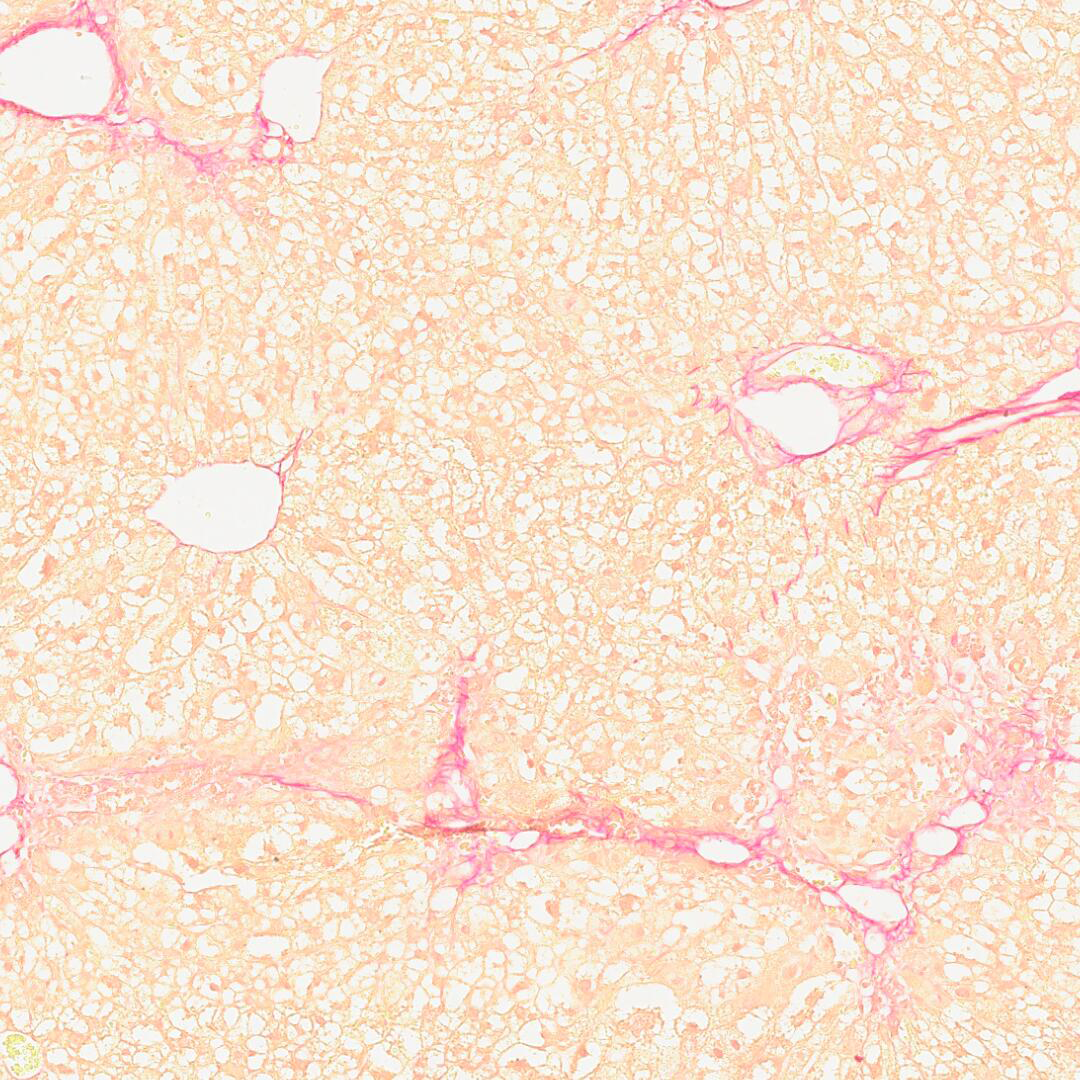

Supplement: Supplementary file 8 — Figure EV Source Data [file 44319_2024_92_MOESM8_ESM.zip › Figure EV4/Figure EV4D/Sirius red/3.tif]

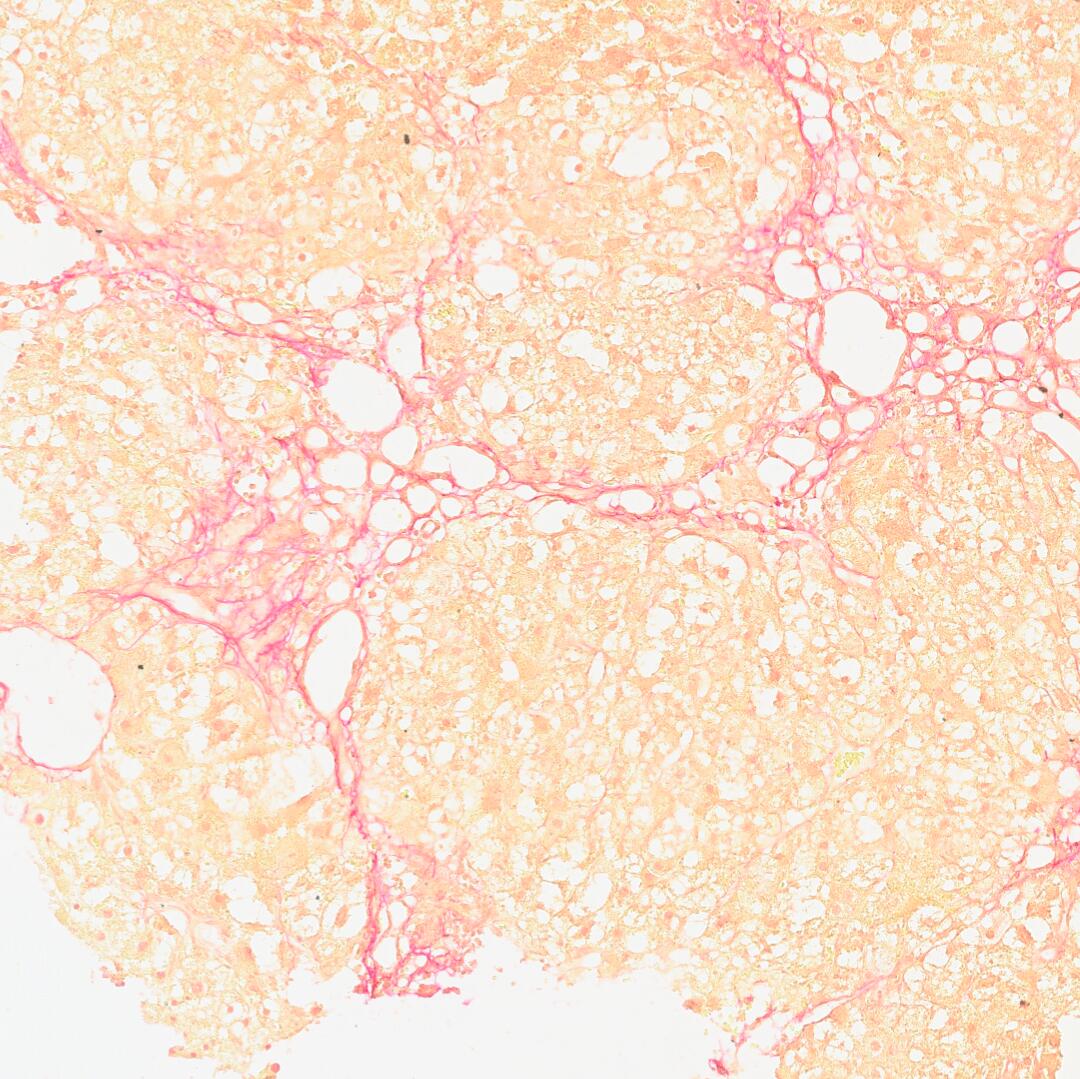

Supplement: Supplementary file 8 — Figure EV Source Data [file 44319_2024_92_MOESM8_ESM.zip › Figure EV4/Figure EV4D/Sirius red/4.tif]

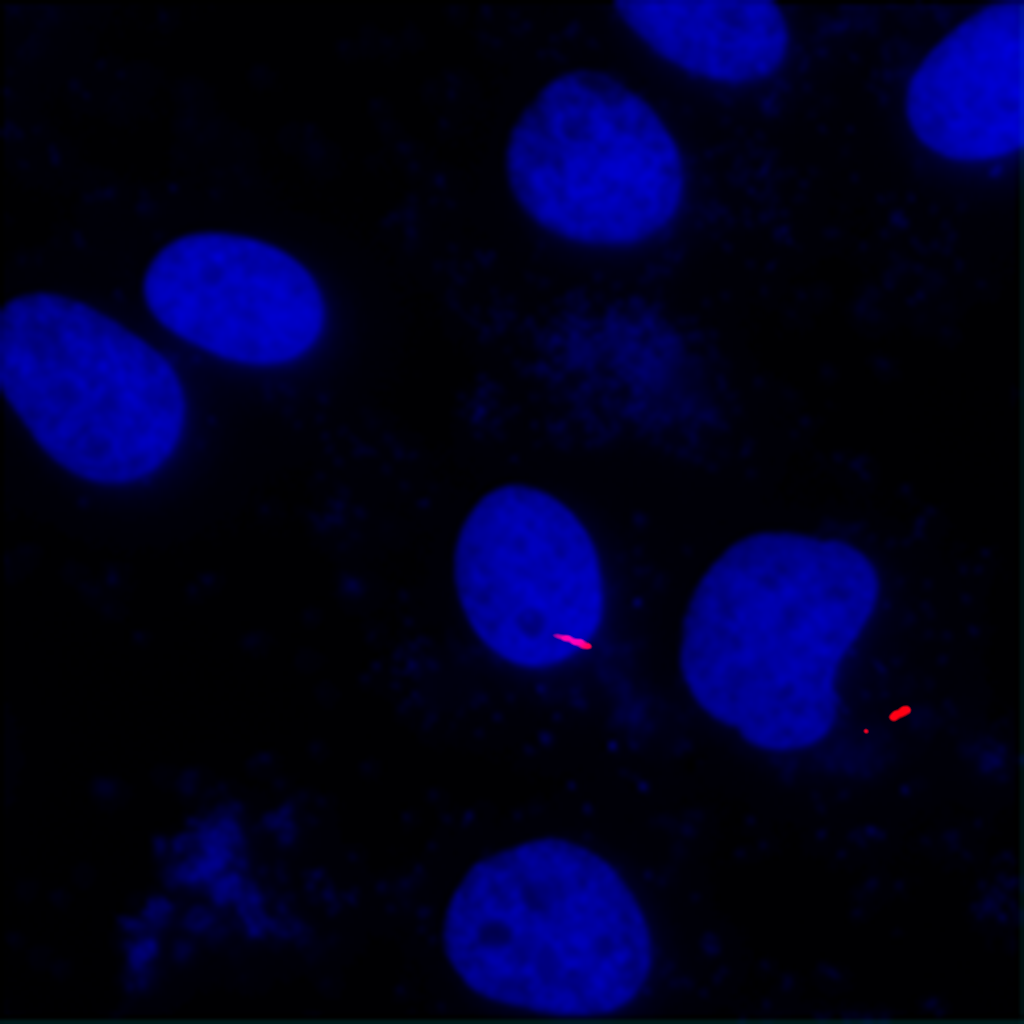

Supplement: Supplementary file 8 — Figure EV Source Data [file 44319_2024_92_MOESM8_ESM.zip › Figure EV5/Figure EV5A/TGF-β.tif]

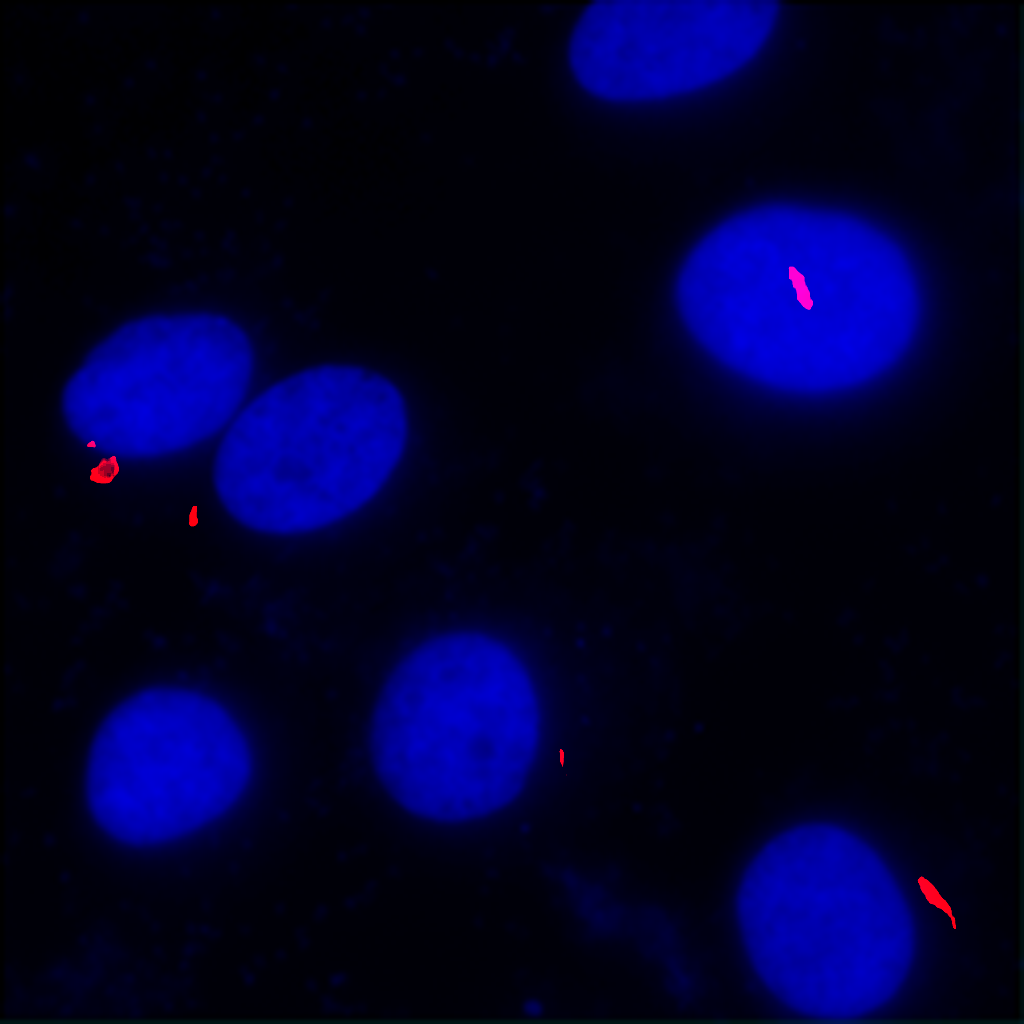

Supplement: Supplementary file 8 — Figure EV Source Data [file 44319_2024_92_MOESM8_ESM.zip › Figure EV5/Figure EV5A/Vehicle.tif]

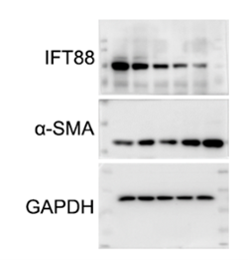

Supplement: Supplementary file 8 — Figure EV Source Data [file 44319_2024_92_MOESM8_ESM.zip › Figure EV5/Figure EV5D/Figure EV5D.tif]

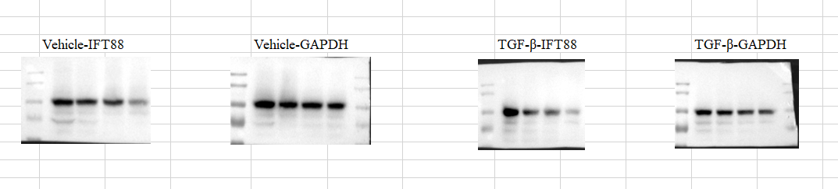

Supplement: Supplementary file 8 — Figure EV Source Data [file 44319_2024_92_MOESM8_ESM.zip › Figure EV5/Figure EV5G/Figure EV5G.tif]

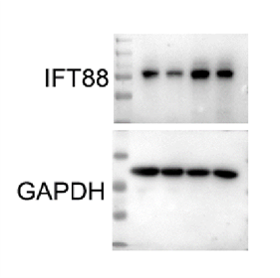

Supplement: Supplementary file 8 — Figure EV Source Data [file 44319_2024_92_MOESM8_ESM.zip › Figure EV5/Figure EV5I/Figure EV5I.tif]
