# Supplementary material for: XIAP-mediated degradation of IFT88 disrupts HSC cilia to stimulate HSC activation and liver fibrosis
Source: EMBO Rep. 2024 Feb 13;25(3):12. doi: 10.1038/s44319-024-00092-y (PMC10933415; doi:10.1038/s44319-024-00092-y)
Supplement: Supplementary file 9 — Table EV1 [file 44319_2024_92_MOESM9_ESM.docx]

**Table EV1.** Primers used for qRT-PCR.

| **Protein** | **Primer Sequence 5′-3′** |
| --- | --- |
| IFT88 (human) | Forward: TGAGGACGACCTTTACTCTGG |
|  | Reverse: GAAAACCCGTGTCATTCTCCAA |
| IFT88 (mouse) | Forward: GACACGGGTTTTCAGCAAGC |
|  | Reverse: TGTCATCGGTCTTCCCATTGAT |
| IL-6 | Forward: CTGCAAGAGACTTCCATCCAG |
|  | Reverse: AGTGGTATAGACAGGTCTGTTGG |
| COL1A1 | Forward: GTACATCAGCCCAAACCCCAAG |
|  | Reverse: CGGAACCTTCGCTTCCATACTC |
| IFNAR2 | Forward: TGTCTGCGAGCCTAGAGACTA |
|  | Reverse: AGCCGGGAATTTCGTATTGTTAT |
| TNF-α | Forward: CCTGTAGCCCACGTCGTAG |
|  | Reverse: GGGAGTAGACAAGGTACAACCC |
| α-SMA | Forward: GTGCTGTCCCTCTATGCCTCTGG |
|  | Reverse: GGCACGTTGTGAGTCACACCATC |
| GAPDH (human) | Forward: CTGGGCTACACTGAGCACC |
|  | Reverse: AAGTGGTCGTTGAGGGCAATG |
| GAPDH (mouse) | Forward: AGGTCGGTGTGAACGGATTTG |
|  | Reverse: TGTAGACCATGTAGTTGAGGTCA |
