# Supplementary material for: XIAP-mediated degradation of IFT88 disrupts HSC cilia to stimulate HSC activation and liver fibrosis
Source: EMBO Rep. 2024 Feb 13;25(3):12. doi: 10.1038/s44319-024-00092-y (PMC10933415; doi:10.1038/s44319-024-00092-y)
Supplement: Supplementary file 11 — Expanded View Figures [file 44319_2024_92_MOESM11_ESM.pdf]

Expanded View Figures

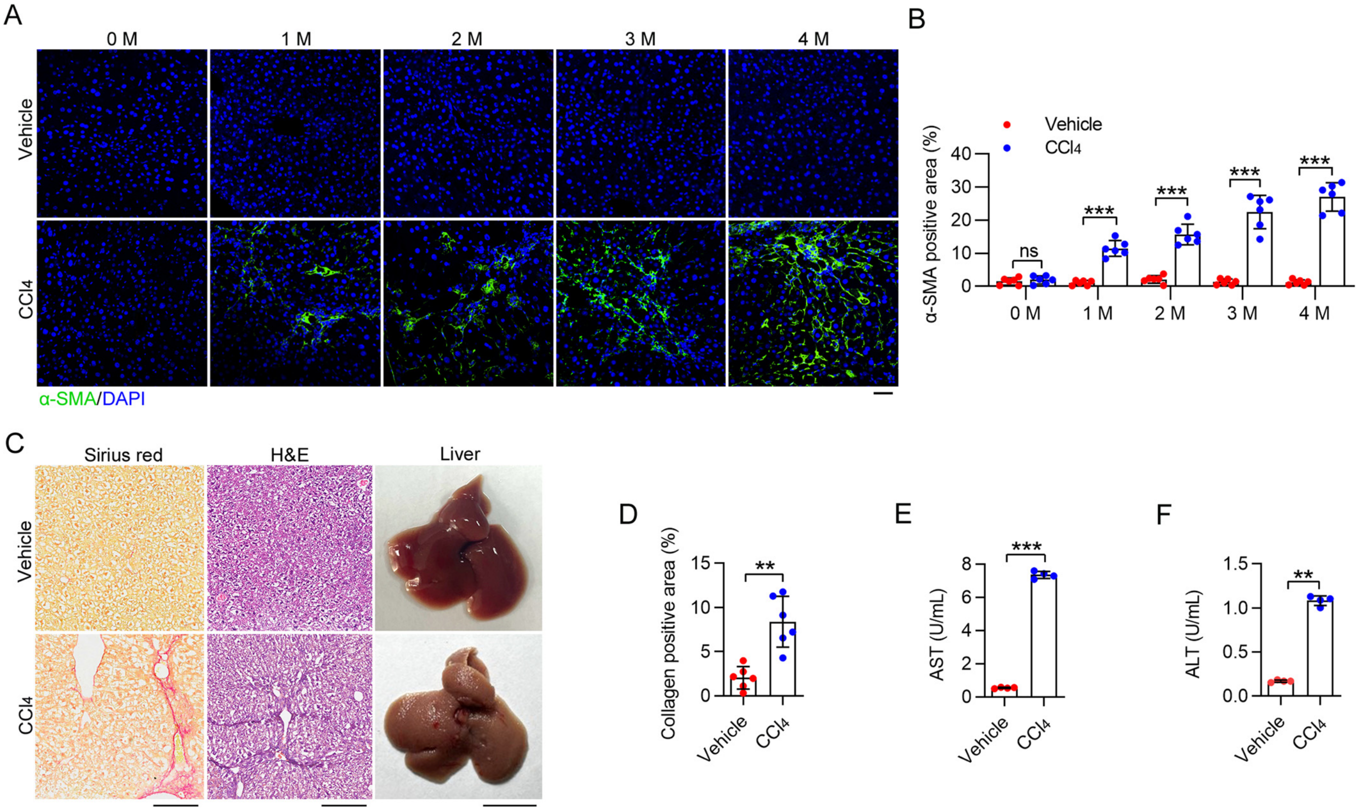

Figure EV1. The mouse model of CCl_4-induced liver fibrosis.

(**A**, **B**) Immunofluorescence images (**A**) and quantification (**B**) of the α-SMA positive areas in the liver of mice treated with CCl_4 for 0–4 months ($n = 6$ mice). Scale bar, 20 μm. (**C**, **D**) Representative images of Sirius red staining and H&E staining in the liver of C57BL6 mice treated with CCl_4 for 2 months (**C**). The Image J software was used to quantify the collagen-positive areas (**D**), $n = 6$ mice). Scale bars for Sirius red staining and H&E staining, 200 μm. Scale bar for liver, 1 cm. (**E**, **F**) The activities of AST (**E**) and ALT (**F**) in the serum were analyzed in CCl_4-treated mice for 2 months ($n = 4$ mice). Data information: Data are presented as mean ± SD. Statistical significance was determined by unpaired two-tailed Student's *t* test. ns not significant; $^{**}P < 0.01$, $^{***}P < 0.001$. Related to Fig. 1. Source data are available online for this figure.

 © The Author(s)

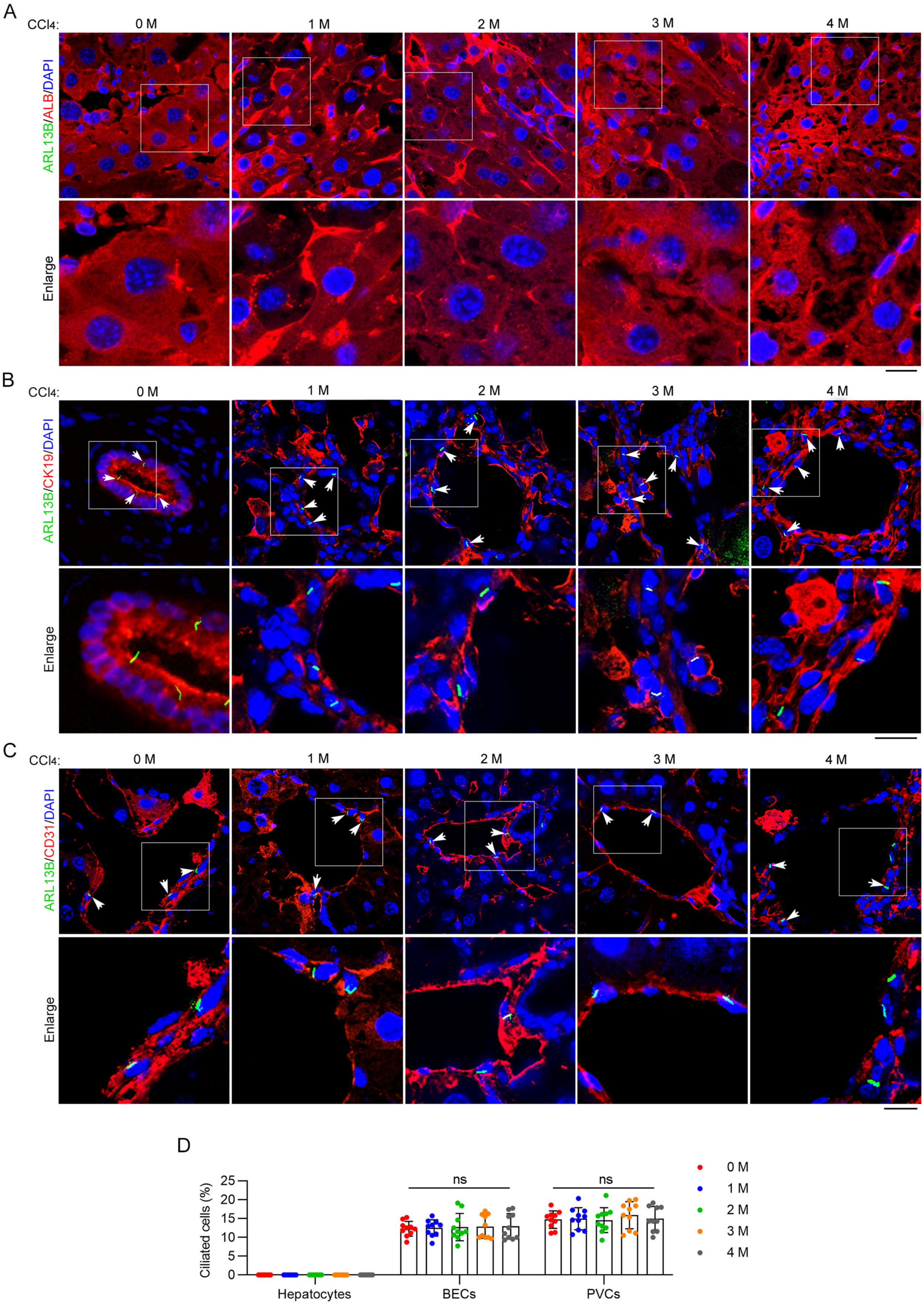
A
CCl4:
0 M
1 M
2 M
3 M
4 M
ARL13B/ALB/DAPI
Enlarge
B
CCl4:
0 M
1 M
2 M
3 M
4 M
ARL13B/CK19/DAPI
Enlarge
C
CCl4:
0 M
1 M
2 M
3 M
4 M
ARL13B/CD31/DAPI
Enlarge
D
Ciliated cells (%)
25
20
15
10
5
0
ns
ns
Hepatocytes
BECs
PVCs
0 M
1 M
2 M
3 M
4 M

Figure EV2. Persistent presence of primary cilia on BECs and PVCs during CCl_4-induced liver fibrosis.

(**A–C**) Immunofluorescence images of primary cilia on hepatocytes (A), BECs (**B**), and PVCs (**C**) from CCl_4-treated mice ($n = 10$ mice). Scale bars, 10 μm. (**D**) Quantification of the percentage of ciliated cells in hepatocytes, PVCs, and BECs from mice described in (**A–C**) ($n = 10$ mice). To quantify the percentage of ciliated cells (**D**), >200 cells from six fields were analyzed for each mouse. The same group of mice ($n = 10$ mice for each time point) were used for Fig. 1A–D and Fig. EV2. Data information: Data are presented as mean ± SD. Statistical significance was determined by one-way ANOVA. ns not significant. Related to Fig. 1. Source data are available online for this figure.

© The Author(s)

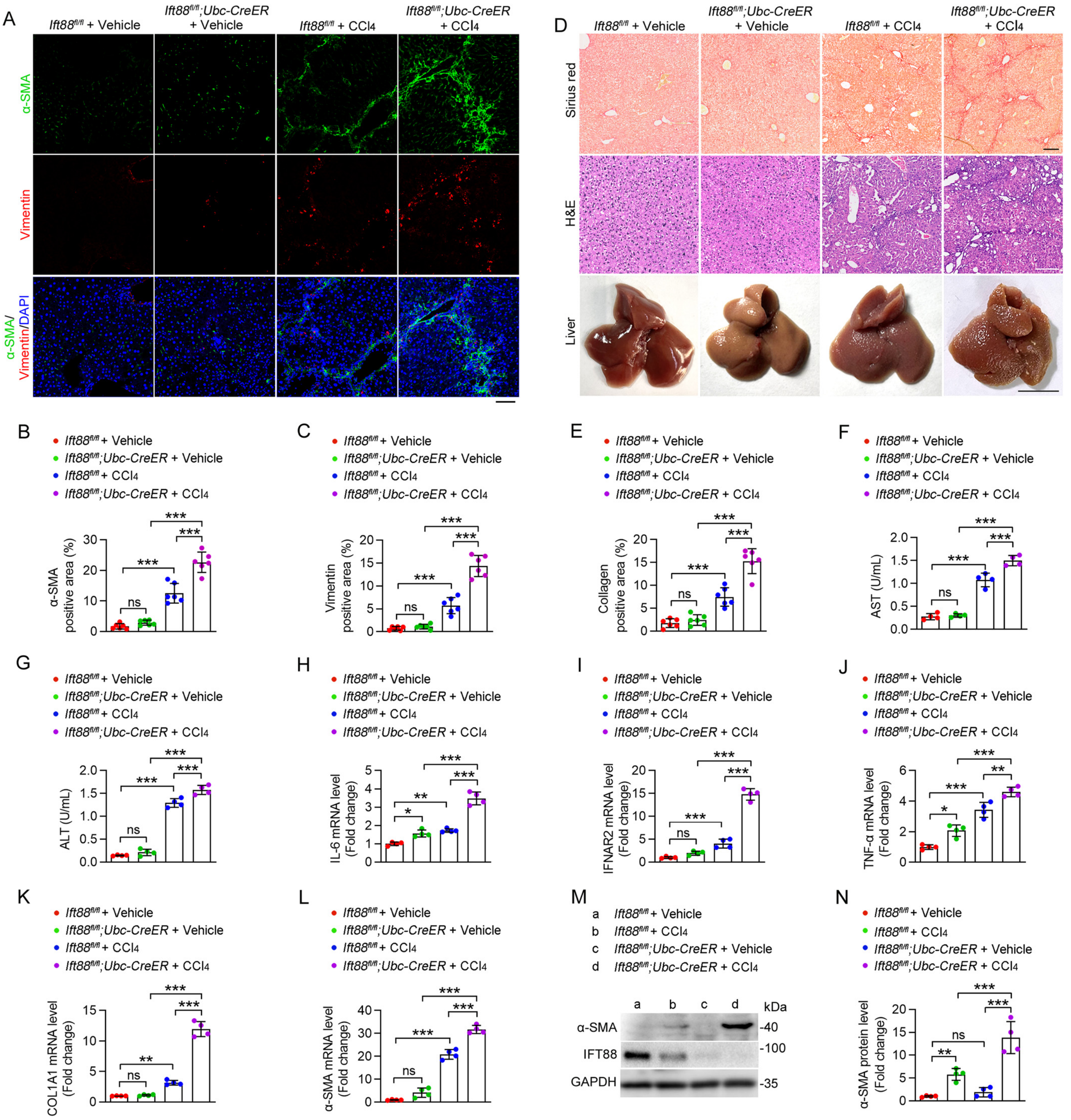

Figure EV3. Whole-body deficiency in IFT88 exacerbates CCl_4-induced liver fibrosis over a 2-month period.

(**A–C**) Immunofluorescence images (**A**) and quantification of the levels of α-SMA (**B**) and vimentin (**C**) in the liver of *Ift88*$^{fl/fl}$ and *Ift88*$^{fl/fl}$*;Ubc-CreER* mice treated with CCl_4 or corn oil (vehicle) for 2 months ($n = 6$ mice). Nuclei were stained with DAPI (blue). Scale bar, 50 μm. (**D, E**) CCl_4-induced liver fibrosis in *Ift88*$^{fl/fl}$ and *Ift88*$^{fl/fl}$*;Ubc-CreER* mice was examined with Sirius red staining and H&E staining (**D**), and the percentage of collagen-positive areas was quantified (**E**) ($n = 6$ mice). Scale bars for Sirius red staining and H&E staining, 200 μm. Scale bar for liver, 1 cm. (**F, G**) Examination of the activities of AST (**F**) and ALT (**G**) in the serum of *Ift88*$^{fl/fl}$ and *Ift88*$^{fl/fl}$*;Ubc-CreER* mice ($n = 4$ mice). (**H–L**) The mRNA levels of IL-6 (H), IFNAR2 (I), TNF-α (J), COL1A1 (K), and α-SMA (L) in liver tissues were measured by quantitative RT-PCR ($n = 4$ mice). (**M, N**) Immunoblotting (**M**) and quantification (**N**) of the levels of IFT88 and α-SMA in the liver of *Ift88*$^{fl/fl}$ and *Ift88*$^{fl/fl}$*;Ubc-CreER* mice treated with CCl_4 or vehicle ($n = 4$ mice). Data information: Data are presented as mean ± SD. Statistical significance was determined by two-way ANOVA with post hoc tests. ns not significant; *$P < 0.05$, **$P < 0.01$, ***$P < 0.001$. Related to Fig. 2. Source data are available online for this figure.

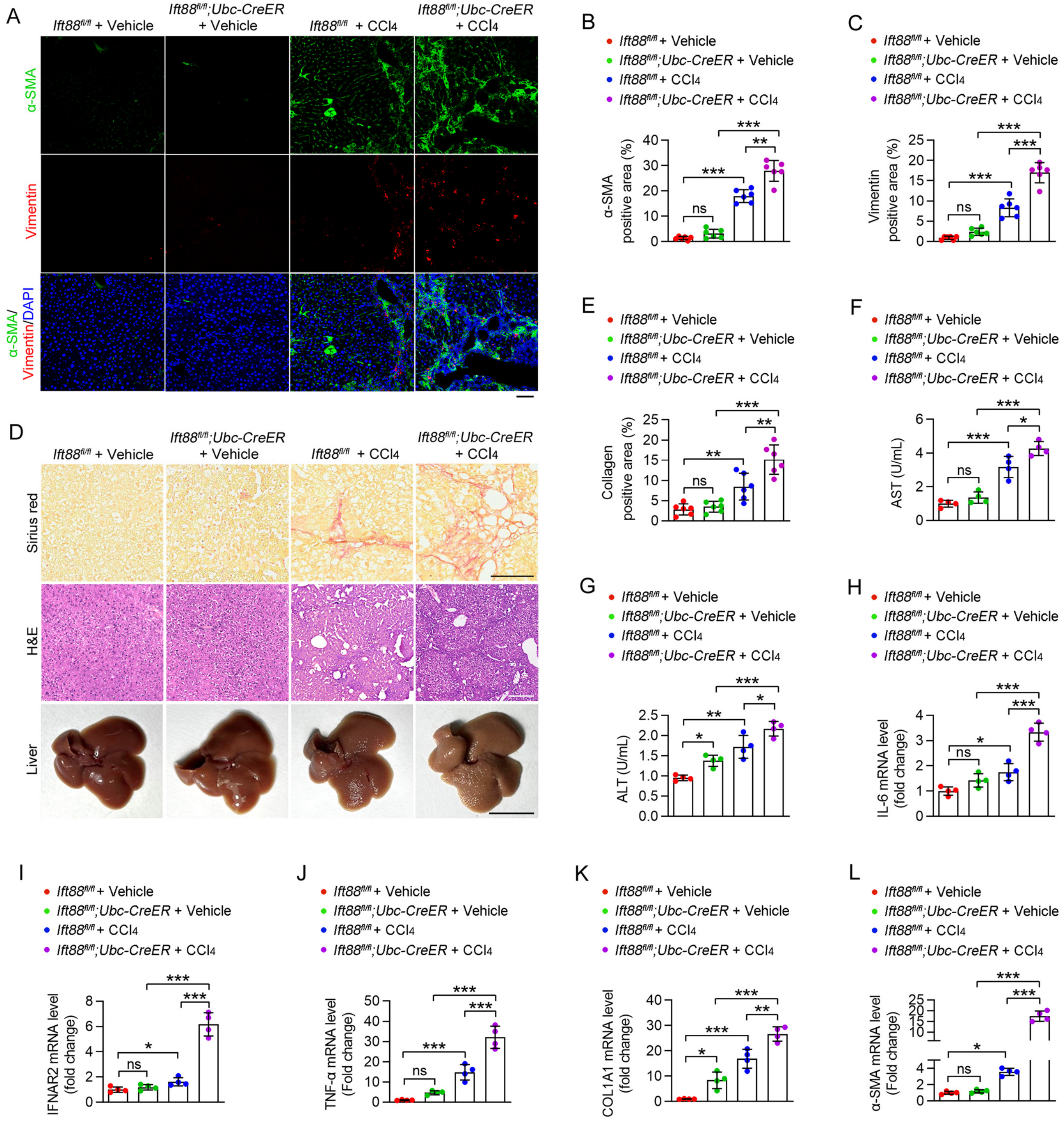

Figure EV4. Whole-body deficiency in IFT88 exacerbates CCl_4-induced liver fibrosis over a 4-month period.

(A–C) Immunofluorescence images **(A)** and quantification of α-SMA **(B)** and vimentin **(C)** in the liver of *Ift88$^{fl/fl}$* and *Ift88$^{fl/fl}$;Ubc-CreER* mice treated with CCl_4 or corn oil (vehicle) for 4 months ($n = 6$ mice). Scale bar, 50 µm. **(D, E)** CCl_4-induced liver fibrosis in *Ift88$^{fl/fl}$* and *Ift88$^{fl/fl}$;Ubc-CreER* mice for 4 months was evaluated with Sirius red staining and H&E staining **(D)**. The Image J software was used for the quantification of collagen-positive areas **(E)** ($n = 6$ mice). Scale bars for Sirius red staining and H&E staining, 200 µm. Scale bar for liver, 1 cm. **(F, G)** The activities of AST **(F)** and ALT **(G)** in the serum were analyzed in *Ift88$^{fl/fl}$* and *Ift88$^{fl/fl}$;Ubc-CreER* mice ($n = 4$ mice). **(H–L)** The mRNA levels of IL-6 **(H)**, IFNAR2 **(I)**, TNF-α **(J)**, COL1A1 **(K)**, and α-SMA **(L)** in liver tissues were measured by quantitative RT-PCR ($n = 4$ mice). Data information: Data are presented as mean ± SD. Statistical significance was determined by two-way ANOVA with post hoc tests. ns, not significant; $^{*}P < 0.05$, $^{**}P < 0.01$, $^{***}P < 0.001$. Related to Fig. 2. Source data are available online for this figure.

 © The Author(s)

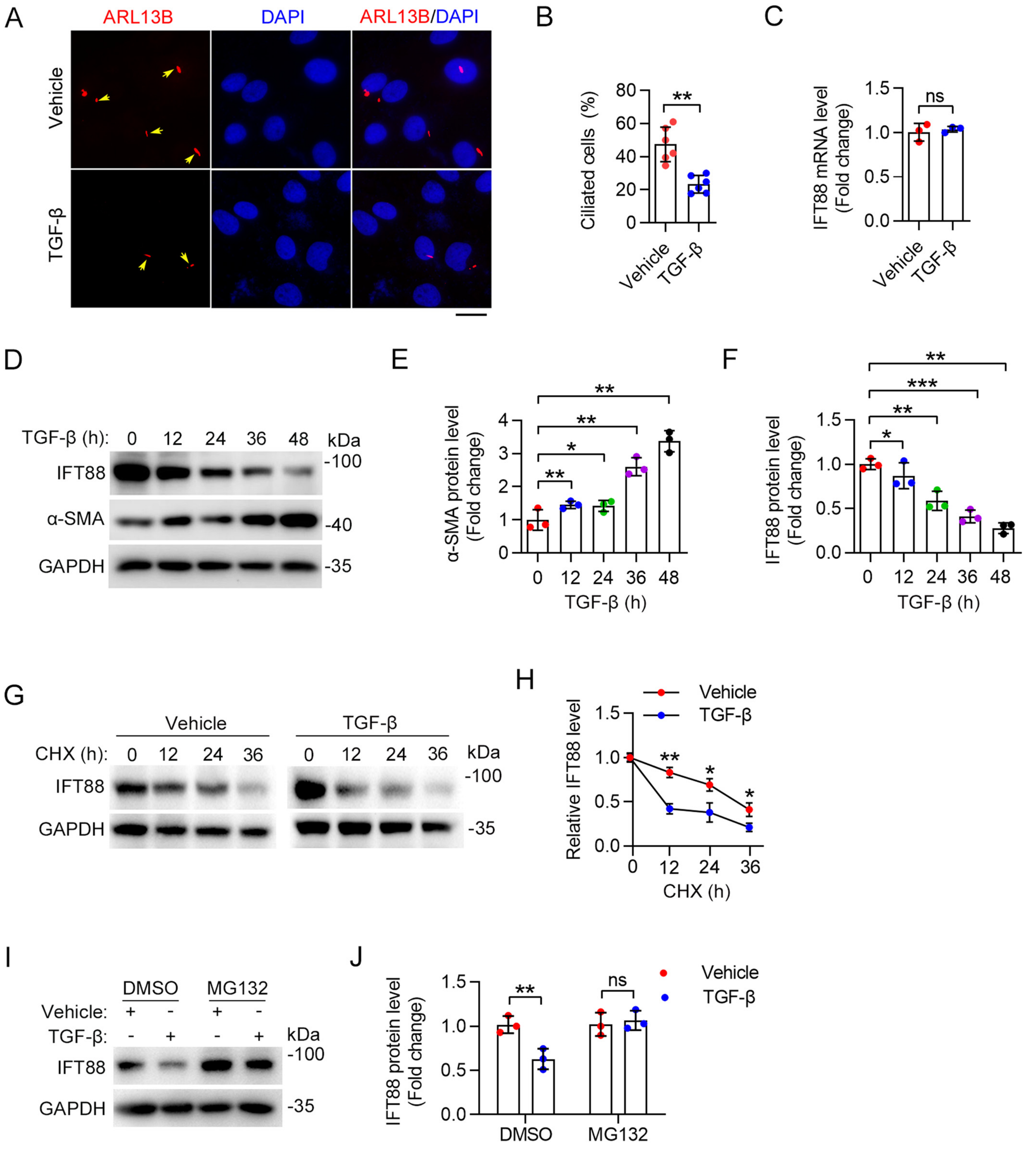
A
ARL13B
DAPI
ARL13B/DAPI
Vehicle
TGF-β
B
Ciliated cells (%)
Vehicle
TGF-β
C
IFT88 mRNA level (Fold change)
ns
D
TGF-β (h): 0 12 24 36 48
kDa
IFT88
α-SMA
GAPDH
-100
-40
-35
E
α-SMA protein level (Fold change)
TGF-β (h)
F
IFT88 protein level (Fold change)
TGF-β (h)
G
Vehicle
TGF-β
CHX (h): 0 12 24 36 0 12 24 36
H
Relative IFT88 level
CHX (h)
I
DMSO
MG132
Vehicle: + - + -
TGF-β: - + - +
J
IFT88 protein level (Fold change)
DMSO
MG132

Figure EV5. TGF-β promotes IFT88 degradation during LX-2 cell activation.

(**A**, **B**) Immunofluorescence images (**A**) and quantification of the density of cilia (**B**) in LX-2 cells treated with TGF-β for 24 h (n = 6 independent experiments). To quantify the percentage of ciliated cells (**B**), >140 cells were analyzed for each experiment. Scale bar, 10 μm. (**C**) IFT88 mRNA expression was measured by quantitative RT-PCR after TGF-β treatment for 24 h in LX-2 cells (n = 3 independent experiments). (**D–F**) Immunoblotting (**D**) of IFT88 and α-SMA in LX-2 cells treated with TGF-β, and the levels of IFT88 (**E**) and α-SMA (**F**) were quantified by densitometry (n = 3 independent experiments). (**G**, **H**) The effect of TGF-β on the half-life of IFT88 in LX-2 cells treated with CHX (20 mg/mL) was examined by immunoblotting (**G**), and the protein half-life curves were obtained (**H**) (n = 3 independent experiments). (**I**, **J**) LX-2 cells were treated with TGF-β for 24 h and then treated with MG132 (5 mM) for 12 h. The levels of IFT88 and GAPDH were examined by immunoblotting (**I**), and the level of IFT88 was determined by densitometry (**J**) (n = 3 independent experiments). Data information: Data are presented as mean ± SD. Statistical significance was determined by one-way ANOVA with post hoc tests (**E**, **F**) or unpaired two-tailed Student's t test (**B**, **C**, **H**, **J**). ns not significant; *P < 0.05, **P < 0.01, ***P < 0.001. Related to Fig. 3. Source data are available online for this figure.

© The Author(s)
