# Supplementary material for: XIAP-mediated degradation of IFT88 disrupts HSC cilia to stimulate HSC activation and liver fibrosis
Source: EMBO Rep. 2024 Feb 13;25(3):12. doi: 10.1038/s44319-024-00092-y (PMC10933415; doi:10.1038/s44319-024-00092-y)
Supplement: Supplementary file 10 — Peer Review File [file 44319_2024_92_MOESM10_ESM.pdf]

EMBO reports

XIAP-mediated degradation of IFT88 disrupts HSC cilia to stimulate HSC activation and liver fibrosis

Renjie Hong, Yanjie Tan, Xiaoyu Tian, Zhenzhou Huang, Jiaying Wang, Hua Ni, Jia Yang, Weiwen Bu, Song Yang, Te Li, Fan Yu, Weilong Zhong, Tao Sun, Xiaohong Wang, Dengwen Li, Min Liu, Yunfan Yang, and Jun Zhou
DOI: 10.15252/embr.202256647

Corresponding author(s): Jun Zhou (junzhou@nankai.edu.cn) , Yunfan Yang (yunfanyang@sdu.edu.cn)

Review Timeline:

Submission Date:	11th Dec 22
Editorial Decision:	25th Jan 23
Revision Received:	23rd Oct 23
Editorial Decision:	11th Dec 23
Revision Received:	15th Dec 23
Accepted:	25th Jan 24

Editor: Martina Rembold

Transaction Report: No Peer Review Process File is available with this article, as the authors have chosen not to make the review process public.
